# Perspectives of Rare Disease Experts on Newborn Genome Sequencing

**DOI:** 10.1001/jamanetworkopen.2023.12231

**Published:** 2023-05-08

**Authors:** Nina B. Gold, Sophia M. Adelson, Nidhi Shah, Shardae Williams, Sarah L. Bick, Emilie S. Zoltick, Jessica I. Gold, Alanna Strong, Rebecca Ganetzky, Amy E. Roberts, Melissa Walker, Alexander M. Holtz, Vijay G. Sankaran, Ottavia Delmonte, Weizhen Tan, Ingrid A. Holm, Jay R. Thiagarajah, Junne Kamihara, Jason Comander, Emily Place, Janey Wiggs, Robert C. Green

**Affiliations:** 1 Division of Medical Genetics and Metabolism, Massachusetts General Hospital for Children, Boston; 2 Department of Pediatrics, Harvard Medical School, Boston, Massachusetts; 3 Division of Genetics, Department of Medicine, Brigham and Women’s Hospital, Boston, Massachusetts; 4 Ariadne Labs, Boston, Massachusetts; 5 Dartmouth Hitchcock Medical Center, Lebanon, New Hampshire; 6 Geisel School of Medicine, Hanover, New Hampshire; 7 Division of Genetics and Genomics, Boston Children’s Hospital, Boston, Massachusetts; 8 Center for Healthcare Research in Pediatrics, Department of Population Medicine, Harvard Pilgrim Health Care Institute, Boston, Massachusetts; 9 Department of Population Medicine, Harvard Medical School, Boston, Massachusetts; 10 Division of Human Genetics, Department of Pediatrics, Children’s Hospital of Philadelphia, Philadelphia, Pennsylvania; 11 Perelman School of Medicine, University of Pennsylvania, Philadelphia; 12 Department of Cardiology and Division of Genetics and Genomics, Department of Pediatrics, Boston Children’s Hospital, Boston, Massachusetts; 13 Division of Pediatric Neurology, Massachusetts General Hospital for Children, Boston; 14 Department of Neurology, Harvard Medical School, Boston, Massachusetts; 15 Division of Hematology/Oncology, Boston Children’s Hospital, Boston, Massachusetts; 16 Department of Pediatric Oncology, Dana-Farber Cancer Institute, Harvard Medical School, Boston, Massachusetts; 17 Laboratory of Clinical Immunology and Microbiology, National Institute of Allergy and Infectious Diseases, National Institutes of Health, Bethesda, Maryland; 18 Division of Pediatric Nephrology, Massachusetts General Hospital for Children, Boston; 19 Manton Center for Orphan Diseases Research, Boston Children’s Hospital, Boston, Massachusetts; 20 Division of Gastroenterology, Hepatology and Nutrition, Boston Children’s Hospital, Boston, Massachusetts; 21 Department of Ophthalmology, Massachusetts Eye and Ear, Boston; 22 Department of Ophthalmology, Harvard Medical School, Boston, Massachusetts; 23 Department of Medicine, Harvard Medical School, Boston, Massachusetts; 24 Broad Institute, Boston, Massachusetts

## Abstract

**Importance:**

Newborn genome sequencing (NBSeq) can detect infants at risk for treatable disorders currently undetected by conventional newborn screening. Despite broad stakeholder support for NBSeq, the perspectives of rare disease experts regarding which diseases should be screened have not been ascertained.

**Objective:**

To query rare disease experts about their perspectives on NBSeq and which gene-disease pairs they consider appropriate to evaluate in apparently healthy newborns.

**Design, Setting, and Participants:**

This survey study, designed between November 2, 2021, and February 11, 2022, assessed experts’ perspectives on 6 statements related to NBSeq. Experts were also asked to indicate whether they would recommend including each of 649 gene-disease pairs associated with potentially treatable conditions in NBSeq. The survey was administered between February 11 and September 23, 2022, to 386 experts, including all 144 directors of accredited medical and laboratory genetics training programs in the US.

**Exposures:**

Expert perspectives on newborn screening using genome sequencing.

**Main Outcomes and Measures:**

The proportion of experts indicating agreement or disagreement with each survey statement and those who selected inclusion of each gene-disease pair were tabulated. Exploratory analyses of responses by gender and age were conducted using *t* and χ^2^ tests.

**Results:**

Of 386 experts invited, 238 (61.7%) responded (mean [SD] age, 52.6 [12.8] years [range 27-93 years]; 126 [52.9%] women and 112 [47.1%] men). Among the experts who responded, 161 (87.9%) agreed that NBSeq for monogenic treatable disorders should be made available to all newborns; 107 (58.5%) agreed that NBSeq should include genes associated with treatable disorders, even if those conditions were low penetrance; 68 (37.2%) agreed that actionable adult-onset conditions should be sequenced in newborns to facilitate cascade testing in parents, and 51 (27.9%) agreed that NBSeq should include screening for conditions with no established therapies or management guidelines. The following 25 genes were recommended by 85% or more of the experts: *OTC*, *G6PC*, *SLC37A4*, *CYP11B1*, *ARSB*, *F8*, *F9*, *SLC2A1*, *CYP17A1*, *RB1*, *IDS*, *GUSB*, *DMD*, *GLUD1*, *CYP11A1*, *GALNS*, *CPS1*, *PLPBP*, *ALDH7A1*, *SLC26A3*, *SLC25A15*, *SMPD1*, *GATM*, *SLC7A7*, and *NAGS*. Including these, 42 gene-disease pairs were endorsed by at least 80% of experts, and 432 genes were endorsed by at least 50% of experts.

**Conclusions and Relevance:**

In this survey study, rare disease experts broadly supported NBSeq for treatable conditions and demonstrated substantial concordance regarding the inclusion of a specific subset of genes in NBSeq.

## Key Points

**Question:**

Do rare disease experts endorse genome sequencing of newborns to screen for treatable genetic diseases, and do they agree on which genes to include?

**Findings:**

In this survey study of 238 rare disease experts, 87.9% agreed that genomic sequencing for monogenic treatable conditions should be available to all newborns. A total of 42 gene-disease pairs were endorsed by more than 80% of the experts.

**Meaning:**

In this study, rare disease experts broadly endorsed screening of newborns with genome sequencing, and there was substantial concordance on a limited number of specific gene-disease pairs for prioritization.

## Teaser

This survey study examines rare disease expert perspectives on newborn genome sequencing and which gene-disease pairs they consider appropriate for screening in newborns.

## Introduction

Newborn screening is a successful, state-mandated public health program that primarily uses mass spectrometry to identify and direct the initial treatment of infants at risk for rare, childhood-onset disorders that are amenable to early treatment.^1,2^ As sequencing technologies have advanced and their costs have dropped in recent decades, interest in expanding newborn screening through newborn genome sequencing (NBSeq) has grown.^3,4,5,6,7,8^ Many states use genetic testing as part of newborn screening for conditions without biochemical markers, such as spinal muscular atrophy, or as a second-tier test for infants with abnormal biochemical laboratory results.^9,10,11,12^ Newborn genome sequencing has the potential to simultaneously evaluate risk for thousands of genetic disorders not amenable to current laboratory assays. Lack of data regarding downstream medical, psychosocial, and economic effects of NBSeq, however, has contributed to concerns regarding its feasibility, cost, clinical utility, and associations with patient autonomy, privacy, and distress.^6,8,13,14,15,16,17,18,19,20,21^

Studies have indicated that a high proportion of individuals, particularly parents, are interested in expanding the number of disorders included in newborn screening,^22,23,24,25^ including through NBSeq.^26,27,28,29,30^ Surveys of pediatricians^31^ and genetic counselors^32^ have revealed more nuanced perspectives but still largely positive attitudes toward NBSeq. Discussions among laboratory directors, patient advocates, and pharmaceutical companies have suggested that systemic changes would be required to integrate genomic sequencing into newborn screening.^33,34^ To date, however, the opinions of medical geneticists and other rare disease experts, who likely would be responsible for implementing NBSeq and managing the care of children with positive findings, have not been systematically elicited.

Diverse approaches have been used to nominate gene and disease candidates for NBSeq. In 2017, the BabySeq Project team evaluated 1514 gene-disease pairs and deemed 954 to be well established, childhood onset, and highly penetrant.^35^ In 2019, the North Carolina Newborn Exome Sequencing for Universal Screening study classified 466 gene-disease pairs as having plausible early intervention and benefit.^36^ The Rx-Genes database, which became publicly available in 2021, delineated 633 genes associated with treatable disorders.^37^ In the context of indication-based diagnosis, but relevant to NBSeq, Owen et al^38^ described a system in which 5 clinical and biochemical geneticists curated interventions for 358 genes. Concurrently, several commercial laboratories have launched expanded newborn screening panels ranging from 109 to 275 genes without clear explanation of their rationale.^39^ This study aimed to assess the perspectives of medical geneticists and other rare disease experts on key questions about NBSeq and to measure concordance regarding specific gene-disease pair candidates for NBSeq.

## Methods

### Survey Design

This survey study was developed to assess the perspectives of rare disease experts on NBSeq, which included (1) 6 questions regarding characteristics of potential disorders for NBSeq, (2) a list of potential gene-disease pairs for NBSeq, and (3) demographic characteristics of respondents. The survey was designed between November 2, 2021, and February 11, 2022, and administered between February 11, 2022, and September 23, 2022. A preliminary version of the survey was developed by a subset of the investigators (N.B.G., S.M.A., N.S., S.W., S.B., and R.C.G.). A pilot survey was conducted with 8 medical geneticists for comprehension and revised to reflect their recommendations. The study was approved by the Mass General Brigham institutional review board. A recruitment email that contained the necessary components of consent was used in lieu of a formal informed consent process. Experts who completed the survey were offered a $50 gift card. The study followed the American Association for Public Opinion Research (AAPOR) reporting guideline^40^ (eTable 1 in Supplement 1).

### Survey Content

Six questions with responses measured on a 5-point Likert scale were used to elicit experts’ perspectives on NBSeq (eAppendix in Supplement 1). A list of gene-disease pairs was designed using data from multiple sources (eFigure in Supplement 1), including Rx-Genes^37^; Treatable ID^41,42^; and gene lists from publications describing commercial offerings of expanded genetic panels for childhood disorders,^39^ genetic disorders treatable by hematopoietic stem cell transplant,^43^ and a model for screening childhood cancer predisposition syndromes.^44^ From this aggregated list of 743 gene-disease pairs, we removed 92 pairs either associated with a core condition or designated as a secondary condition (ie, disorders that share biomarkers with core conditions and may be incidentally ascertained by newborn screening) on the US Department of Health and Human Services Recommended Uniform Screening Panel (RUSP).^45^ The remaining list of 651 genes was included in the final survey (eTable 2 in Supplement 1).

Gene-disease pairs were sorted into the following clinical areas: cardiovascular (17 genes), endocrinology (95 genes), gastroenterology (14 genes), hematology (90 genes), immunology (167 genes), metabolic (137 genes), nephrology (24 genes), neurology (83 genes), oncology (18 genes), ophthalmology (4 genes), and pulmonology (2 genes). For each gene, experts were asked whether they would recommend that pathogenic and likely pathogenic variants be screened in newborns. Experts were invited to assess all genes or to select the clinical area with which they were most familiar. They indicated their responses using radio buttons labeled yes, no, or unsure. Two genes (*SLC19A3* and *SLC35C1*) were paired on the survey with the incorrect disease and were subsequently deleted from analyses.

Experts were asked for their age, gender (female, male, nonbinary, or other), race, ethnicity, state of residence, years in practice, primary practice setting, and the patient population they serve. Options for race (American Indian or Alaska Native, Asian, Black or African American, Native Hawaiian or Pacific Islander, White, or other) and ethnicity (Hispanic, Latino, or Spanish origin) were self-selected from a list^46^ and included to investigate whether respondents were representative of the field of medical geneticists. Missing demographic information on nonresponding experts, specifically age and gender, was supplemented from publicly available resources.

### Enrollment of Rare Disease Experts

From February 11, 2022, through September 23, 2022, 386 experts were invited to participate. All 142 program directors of genetics and genomics programs accredited by the Accreditation Council for Graduate Medical Education were invited. Included in this group were directors of programs in molecular genetic pathology (n = 37), medical genetics and genomics (MGG) (n = 40), medical biochemical genetics (n = 7), clinical biochemical genetics (n = 11), internal medicine and MGG (n = 4), maternal fetal medicine and MGG (n = 7), laboratory genetics and genomics (n = 20), reproductive endocrinology and MGG (n = 3), and global molecular biology/genetics programs (n = 13).

Thirteen clinical champions, a group of individuals with expertise in each clinical area as demonstrated by recent scholarship and involvement in the care of patients with rare disease, were asked to complete the survey. This group included experts in pediatric cardiovascular disease (A.R.), endocrinology (I.H.), gastroenterology (J.R.T.), hematology (V.G.S.), immunology (O.D.), metabolism (R.G.), nephrology (W.T.), neurology (M.W.), oncology (J.K.), ophthalmology (J.C., E.P., and J.W.), and pulmonology (A.M.H.). They then provided the names of a total of 81 content area experts in their fields to participate in the survey. These content area experts were selected at the discretion of the clinical champions but broadly represented rare disease experts who were clinically active; had done scholarly and/or advocacy work related to newborn screening; and represented demographic, geographic, and gender diversity. An additional 150 individuals, including 138 clinicians and academicians and 12 employees of pharmaceutical companies, were identified as experts by the investigators based on their knowledge of an area of pediatric genetic disease and were invited to participate.

We emailed each prospective respondent a maximum of 7 times over the data collection period. Two weeks before closing the survey, a study team member called each individual who had not yet responded to the survey.

### Statistical Analysis

To explore whether there were patterns in the 649 gene-disease pairs selected, we created a table recording the inheritance pattern, prevalence, age of onset, disease symptoms, orthogonal tests (nonmolecular tests that can be used to confirm a diagnosis), intervention, age of intervention implementation, and specialist leading the intervention for each (eTable 3 in Supplement 1). The table, which was not shared with survey invitees, was reviewed and finalized by each of the 13 clinical champions.

Descriptive statistics, including means with SDs and counts with percentages, were reported for basic demographic characteristics. The age and gender distribution of respondents were compared with nonrespondents using a *t* test and χ^2^ test, respectively. The Likert scale responses to perspectives on NBSeq were dichotomized into agree (combining agree and somewhat agree) vs do not agree (combining all other responses). Multivariable logistic regression analyses were conducted to examine agreement with each perspective by age (reported per 10-year increase) and sex (female vs male), as well as by participant type (program director vs all other experts), reporting adjusted odds ratios (ORs) and 95% CIs. Responses regarding experts’ recommendation for each genetic disorder were tabulated, and rates of concordance were calculated and expressed as percentages. All statistical tests were 2-sided, with *P* < .05 considered statistically significant. Data were analyzed using SAS Studio, version 3.7 statistical software (SAS Institute Inc).

## Results

### Respondent Characteristics

Of 386 experts to whom the survey was sent, 238 (61.7%) responded (eTable 4 in Supplement 1). Respondents included 64 of 142 (45.1%) program directors, all 13 (100%) clinical champions, 50 of 81 (61.7%) content area experts, and 111 of 150 (74.0%) additional rare disease experts. There were no statistically significant differences between respondents and nonrespondents in age (mean [SD], 52.6 [12.8] vs 54.8 [9.5] years, respectively; *P* = .07) or gender (126 [52.9%] women and 112 [47.1%] men vs 65 [43.9%] women and 83 [56.1%] men, respectively; *P* = .09). Respondents’ race was self-reported as Asian (26 [10.9%]), Native Hawaiian or Pacific Islander (2 [0.8%]), White (141 [59.2%]), multiracial (4 [1.7%]), other (5 [2.1%]), and unknown (60 [25.2%]). Respondent ethnicity was self-reported as Hispanic (7 [2.9%]), non-Hispanic (169 [71.0%]), and unknown (62 [26.1%]).

### Perspectives on NBSeq

**Figure 1.**
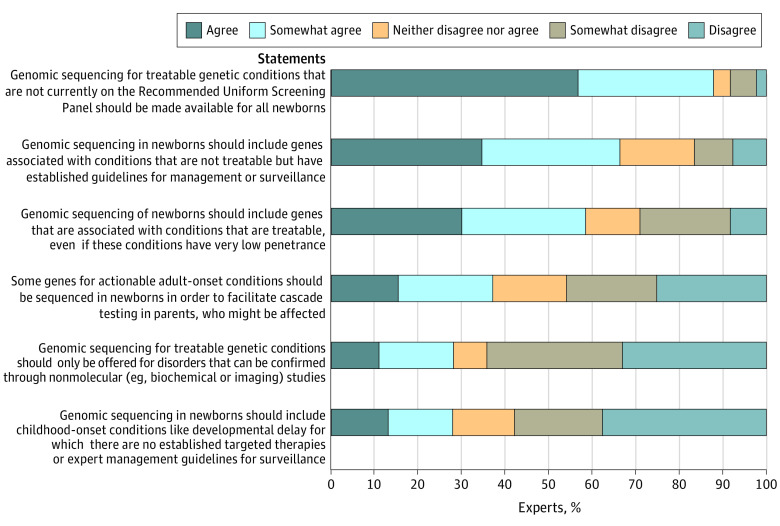
Experts’ Perspectives on the Scope of Disorders Included in Newborn Genome Sequencing Experts were asked to indicate whether they agreed with 6 statements regarding the types of disorders that should be included in newborn genome sequencing.

Figure 1 summarizes the experts’ perspectives regarding the types of disorders that should be included on NBSeq. Younger experts were significantly more likely to agree that genomic sequencing for treatable genetic conditions that are not currently on the RUSP should be made available for all newborns (OR, 0.67 per 10-year increase in age; 95% CI, 0.48-0.95; *P* = .02). Agreement with all other questions did not significantly differ by age or gender. Program directors had lower percentages of agreement with most of the questions compared with all other experts. However, after adjusting for age and gender, the only statistically significant finding was that program directors were less likely to agree with the statement that “genomic sequencing in newborns should include childhood-onset conditions like developmental delay for which there are no established targeted therapies or expert management guidelines for surveillance” compared with other experts (OR, 0.36; 95% CI, 0.15-0.89; *P* = .03).

### Free-Text Responses

Experts were invited to offer text responses throughout the survey, including suggestions for additional genes to be included in NBSeq (eTable 5 in Supplement 1) and to provide unstructured responses to the survey (Table 1). Several themes emerged in the text responses, including concern about the low prevalence of some of the disorders included in the survey; an emphasis on prioritizing treatable disorders; and mixed reactions to disorders that range in their age of onset, including those with attenuated adolescent or adult-onset forms.

**Table 1.** Themes From Free-Text Responses

Theme	Quotations
Emphasis on efficacy of treatments	“I think any diagnosis that can impact management is worth screening for.”
	“Probably obvious, but would select only treatable conditions–though this is likely to change as gene therapies advance.”
	“I don't think that we should be doing screening for conditions with dubious treatment.…It causes anxiety and fundamentally changes relationships.”
	“In principle, there is no reason to screen for any disorder that has no effective treatment.…Once an effective treatment becomes available and especially if treatment prior to symptoms is important to prevent irreversible damage, then screen.”
Concern about low prevalence of disorders	“Many [o]f these are super rare.”
	“I have put yes for many of these genes…that would benefit hugely from early identification and therefore treatment; however, their incidence is so low that universal screening would not likely be cost-effective.”
	“Others too uncommon should be in diagnostic panels.”
Mixed reactions to disorders with noninfantile age of onset	“I don’t think that you need to know [about some of these disorders] in the newborn period, and I don’t think that newborn screening should be used for identifying disease in parents.”
	“Newborn sequencing should focus on newborn diseases. For diseases with later manifestations, screening at age-appropriate times is more reasonable.”
	“For these, I’d be less concerned [with early identification through newborn screening] because of ages of onset and lack of presymptomatic opportunities to intervene.”
	“Despite some of these disorders being of later onset, I fully support early detection and appropriate intervention.”

### Expert Concordance for Gene-Disease Associations

**Figure 2.**
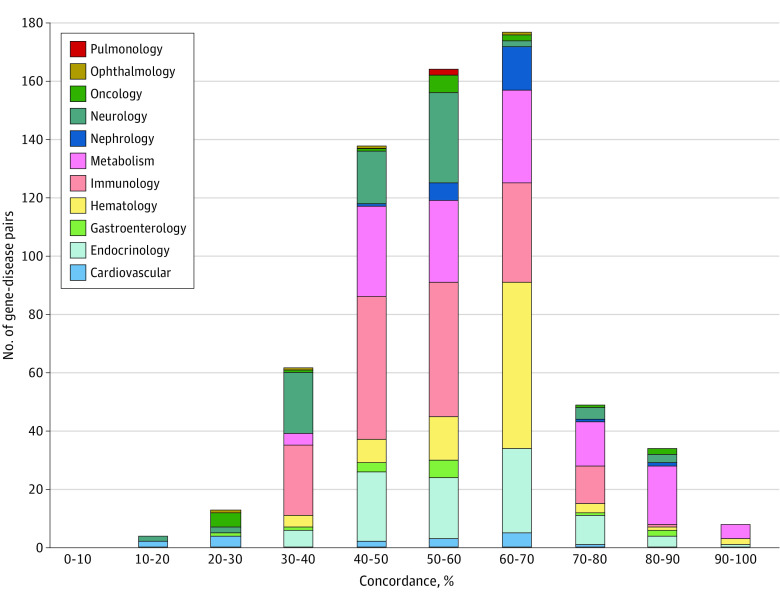
Distribution of Expert Concordance for the Inclusion of Gene-Disease Pairs in Newborn Genome Sequencing Among 649 gene-disease pairs in the survey, each was endorsed by at least 11.8% of experts. Overall, 25 gene-disease pairs were endorsed by 85% or more of the experts.

Among the experts who responded, 161 (87.9%) agreed that NBSeq for monogenic treatable disorders should be made available to all newborns, 107 (58.5%) agreed that NBSeq should include genes associated with treatable disorders even if those conditions were low penetrance, 68 (37.2%) agreed that actionable adult-onset conditions should be sequenced in newborns to facilitate cascade testing in parents, and 51 (27.9%) agreed that NBSeq should include screening for conditions with no established therapies or management guidelines. Among the 649 gene-disease pairs presented in the survey, each was endorsed by at least 11.8% of experts. Overall, 25 gene-disease pairs (*OTC*-ornithine transcarbamylase deficiency [OCT]; *G6PC*-glycogen storage disease Ia; *SLC37A4*-glycogen storage disease Ib; *CYP11B1*-congenital adrenal hyperplasia due to 11-β-hydroxylase deficiency; *ARSB*-mucopolysaccharidosis type VI; *F8*-hemophilia A; *F9*-hemophilia B; *SLC2A1*-GLUT1 deficiency syndrome 1; *CYP17A1*-17-α-hydroxylase/17,20-lyase deficiency; *RB1*-retinoblastoma [hereditary]; *IDS*-mucopolysaccharidosis II; *GUSB*-mucopolysaccharidosis type VII; *DMD*-Duchenne muscular dystrophy and other dystrophinopathies; *GLUD1*-hyperinsulinism-hyperammonemia syndrome; *CYP11A1*-adrenal insufficiency, congenital, with 46XY sex reversal, partial or complete; *GALNS*-mucopolysaccharidosis IVA; *CPS1*-carbamoyl phosphate synthetase I deficiency; *PLPBP*-vitamin B6–dependent epilepsy; *ALDH7A1*-pyridoxine-dependent epilepsy; *SLC26A3*-congenital secretory chloride diarrhea; *SLC25A15*-hyperornithinemia-hyperammonemia-homocitrullinuria syndrome; *SMPD1*-Niemann-Pick disease, type A and type B; *GATM*-cerebral creatine deficiency syndrome 3; *SLC7A7*-lysinuric protein intolerance; and *NAGS*-N-acetylglutamate synthase deficiency) were endorsed by 85% or more of the experts (Table 2). The first of these 8 gene-disease pairs were endorsed by at least 90% of experts (Figure 2). Among 42 gene-disease pairs with 80% or higher concordance, 25 (60%) were metabolic disorders, 5 (12%) were endocrinologic disorders, 3 (7%) were neurologic disorders, 3 (7%) were hematologic disorders, 2 (5%) were gastroenterologic disorders, 2 (5%) were hereditary cancer predisposition syndromes, 1 (2%) was a renal disorder, and 1 (2%) was an immunologic disorder. A total of 432 genes were endorsed by 50% or more experts.

**Table 2.** Gene-Disease Pairs With 85% or Higher Concordance Among Experts

Gene	Disease	Clinical area	No. (%)			Responses, No.	Prevalence of disease (per 100 000)	Age of onset	Orthogonal test for at-risk infants	Intervention
			Yes	No	Unsure					
*OTC*	Ornithine transcarbamylase deficiency	Metabolism	61 (98.4)	1 (1.6)	0	62	1.5	Infancy to adulthood	Orotic acid level, plasma amino acids	Protein restriction, citrulline, nitrogen scavengers, liver transplant
*G6PC*	Glycogen storage disease Ia	Metabolism	57 (93.4)	3 (4.9)	1 (1.6)	61	0.04	Infancy	No	Cornstarch, nighttime intragastric continuous glucose infusion, low-carbohydrate and high-protein diet
*SLC37A4*	Glycogen storage disease Ib	Metabolism	56 (93.3)	4 (6.7)	0	60	0.04	Infancy	No	Cornstarch, nighttime intragastric continuous glucose infusion, allopurinol, statin, granulocyte colony-stimulating factor, immunomodulators, low-carbohydrate and high-protein diet
*CYP11B1*	Congenital adrenal hyperplasia due to 11-β-hydroxylase deficiency	Endocrinology	35 (92.1)	2 (5.3)	1 (2.6)	38	0.8	Infancy to adolescence	Serum 11-deoxycortisol and 11-deoxycorticosterone levels	Hydrocortisone
*ARSB*	Mucopolysaccharidosis type VI	Metabolism	54 (91.5)	3 (5.1)	2 (3.4)	59	0.3	Childhood	Arylsulfatase B enzyme activity, urine glycosaminoglycans	Galsulfase enzyme replacement, HSCT
*F8*	Hemophilia A	Hematology	37 (90.2)	4 (9.8)	0	41	7.5	Infancy to adolescence	Factor VIII level	Factor VIII
*F9*	Hemophilia B	Hematology	37 (90.2)	4 (9.8)	0	41	1.3	Infancy to adolescence	Factor IX level	Factor IX
*SLC2A1*	GLUT1 deficiency syndrome 1	Metabolism	55 (90.2)	3 (4.9)	3 (4.9)	61	1.7	Infancy	Blood glucose, cerebrospinal fluid glucose	Ketogenic diet, carnitine supplementation, avoid barbiturates, methylxanthine, valproic acid
*CYP17A1*	17-α-Hydroxylase/17,20-lyase deficiency	Endocrinology	34 (89.5)	2 (5.3)	2 (5.3)	38	Unknown	Infancy	Potassium, cortisol, and corticotropin levels	Spironolactone, hydrocortisone
*RB1*	Retinoblastoma (hereditary)	Oncology	50 (89.3)	5 (8.9)	1 (1.8)	56	Unknown	Infancy to childhood	Eye examination	Surveillance
*IDS*	Mucopolysaccharidosis II	Metabolism	55 (88.7)	5 (8.1)	2 (3.2)	62	0.8	Early childhood	Iduronate 2-sulfatase enzyme activity, urine glucosaminoglycans	Idursulfase enzyme replacement, HSCT
*GUSB*	Mucopolysaccharidosis type VII	Metabolism	54 (88.5)	4 (6.6)	3 (4.9)	61	0.1	Neonatal to adolescence	Leukocyte β-glucuronidase enzyme activity	Vestronidase alfa enzyme replacement, HSCT
*DMD*	Duchenne muscular dystrophy and other dystrophinopathies	Neurology	44 (88.0)	2 (4.0)	4 (8.0)	50	Unknown	Early childhood	Serum creatine kinase level	Eteplirsen for exon 51 skipping, casimersen for exon 45 skipping, golodirsen for exon 53 skipping, vitolarsen for exon 53 skipping
*GLUD1*	Hyperinsulinism-hyperammonemia syndrome	Metabolism	54 (87.1)	4 (6.5)	4 (6.5)	62	2.4	Neonatal	Ammonia, glucose, insulin, free fatty acid levels	Diazoxide, somatostatin analogs, nifedipine, glucagon, insulinlike growth factor 1, glucocorticoids, growth hormone, pancreatic resection, mammalian target of rapamycin inhibitors, glucagon-like peptide 1 receptor antagonists, sirolimus
*CYP11A1*	Adrenal insufficiency, congenital, with 46XY sex reversal, partial or complete	Endocrinology	33 (86.8)	1 (2.6)	4 (10.5)	38	Unknown	Infancy to early childhood	Serum cortisol, aldosterone and corticotropin levels	Hydrocortisone, fludrohydrocortisone
*GALNS*	Mucopolysaccharidosis IVA	Metabolism	52 (86.7)	6 (10.0)	2 (3.3)	60	0.3	Early childhood	β-Glucuronidase enzyme activity	Vestronidase alfa enzyme replacement, HSCT
*CPS1*	Carbamoyl phosphate synthetase I deficiency	Metabolism	51 (86.4)	5 (8.5)	3 (5.1)	59	0.08	Neonatal	Ammonia, plasma amino acids	Protein restriction, citrulline, nitrogen scavengers, liver transplant, N-carbamylglutamate
*PLPBP*	Vitamin B6–dependent epilepsy	Neurology	43 (86.0)	3 (6.0)	4 (8.0)	50	2.6	Prenatal to neonatal	Intravenous pyridoxine trial on electroencephalogram	Pyridoxine, arginine supplementation, lysine restriction
*ALDH7A1*	Pyridoxine-dependent epilepsy	Neurology	42 (85.7)	4 (8.2)	3 (6.1)	49	2.6	Infancy to childhood	Urine organic acids	Pyridoxine, arginine supplementation, lysine restriction
*SLC26A3*	Congenital secretory chloride diarrhea	Gastroenterology	29 (85.3)	3 (8.8)	2 (5.9)	34	Unknown	Infancy	Fecal chloride content	Omeprazole, chloride, sodium, potassium
*SLC25A15*	Hyperornithinemia-hyperammonemia-homocitrullinuria syndrome	Metabolism	52 (85.2)	4 (6.6)	5 (8.2)	61	0.07	Neonatal to adulthood	Plasma ornithine, urinary homocitrulline	Protein-restricted diet, citrulline, nitrogen scavengers
*SMPD1*	Niemann-Pick disease, type A and type B	Metabolism	51 (85.0)	6 (10.0)	3 (5.0)	60	0.4	Infancy to adulthood	Oxysterol analysis, filippin staining	HSCT, recombinant human acid sphingomyelinase enzyme replacement therapy, liver transplant (late-onset only)
*GATM*	Cerebral creatine deficiency syndrome 3	Metabolism	51 (85.0)	6 (10.0)	3 (5.0)	60	0.01	Childhood	Guanidinoacetate, creatine, and creatinine levels in urine and plasma	Creatine monohydrate
*SLC7A7*	Lysinuric protein intolerance	Metabolism	51 (85.0)	5 (8.3)	4 (6.7)	60	Unknown	Infancy	24-h Urinary excretion of cationic amino acids	Protein restriction, carnitine, citrulline, lysine supplementation, nitrogen scavengers
*NAGS*	N-acetylglutamate synthase deficiency	Metabolism	51 (85.0)	5 (8.3)	4 (6.7)	60	0.05	Neonatal	Ammonia, peracetic acid	N-carbamyl glutamate, low-protein diet, nitrogen scavengers, liver transplant

Abbreviation: HSCT, hematopoietic stem cell transplant.

Because each clinical area included a different number of genes, we also tabulated the percentage of gene-disease pairs per area endorsed by experts. The highest percentage of genes that reached 80% or higher concordance were related to metabolic disorders (25 of 135 [18.5%]). Additionally, genes related to gastroenterology (2 of 14 [14.3%]), hereditary cancer syndromes (2 of 18 [11.1%]), endocrinology (5 of 95 [5.3%]), nephrology (1 of 24 [4.2%]), neurology (3 of 83 [3.6%]), hematology (3 of 90 [3.3%]), and immunology (1 of 167 [0.6%]) reached 80% or higher concordance. The gene-disease pair with the highest concordance was *OTC*, which is associated with OTC deficiency (62 of 63 [98.4%]). None of the cardiovascular, ophthalmology, or pulmonology genes reached 80% or higher concordance.

## Discussion

Newborn genome sequencing presents an opportunity to expand the reach of newborn screening by identifying more infants at risk for treatable genetic disorders, with the goal of improving childhood health and mortality. Here we present, to our knowledge, the first survey of rare disease experts on NBSeq, the results of which suggest that rare disease experts support the implementation of NBSeq with substantial agreement regarding which gene-disease pairs should be screened. In particular, we identified 25 gene-disease pairs with 85% or higher concordance that span several clinical areas and may be strong candidates for future inclusion in clinical and research NBSeq programs.

Many of the gene-disease pairs with high concordance are clinically similar to disorders currently included on the RUSP, but our results highlight the role of NBSeq as an adjunct screening modality. Concordance was highest among metabolic and endocrinologic disorders, clinical areas that are already well represented in current newborn screening. In particular, OTC deficiency, a condition with high morbidity and mortality in male infants, was recommended for inclusion in NBSeq by nearly all experts who evaluated it. Although some state programs currently screen for OTC deficiency using a glutamate/citrulline ratio, such biochemical measurements are sensitive to sample handling and may result in both false-positive and false-negative results,^47,48^ whereas NBSeq may more accurately identify children at risk for disease. Experts demonstrated high concordance regarding the inclusion of other treatable, infant-onset metabolic conditions that have no stable or pathognomonic biochemical screening biomarker and that could easily be assayed on a population level, such as glycogen storage diseases, types Ia and Ib; hyperinsulinism-hyperammonemia syndrome; and hereditary fructose intolerance. Additionally, 2 additional forms of congenital adrenal hyperplasia that are not ascertained by current newborn screening were highly endorsed. Our findings suggest that NBSeq could be used as a tool to further the long-standing goals of newborn screening by identifying infants at risk for additional severe, treatable, childhood-onset disorders in clinical areas that have already been deemed appropriate for screening but are not amenable to detection by current methodologies.

Experts also showed high concordance regarding the inclusion of disorders with newly developed and emerging pharmacologic therapies, such as Niemann-Pick disease, types A and B, for which enzyme replacement therapy (olipudase alfa) became clinically available in March 2022,^49,50,51^ and Duchenne muscular dystrophy, for which several exon-skipping therapies have emerged in addition to standard steroid therapy^52,53^ and for which trials of gene therapy are ongoing.^54,55^ Whereas current newborn screening programs often require the use of new biochemical methods to identify additional disorders, NBSeq provides a resource that can be repeatedly queried as treatments or clinical trials become available.

Our findings suggest that experts also support the inclusion of gene-disease pairs in clinical areas that have not previously been included in screening, such as childhood-onset cancer predisposition conditions and bleeding disorders. For example, *RB1*, which is associated with hereditary retinoblastoma, was endorsed for screening by 50 of 56 experts (89.3%). Early detection of retinoblastoma improves outcomes, affects ocular salvage, and leads to enhanced preservation of vision.^56^ Hemophilia A and B, which lead to symptoms ranging from severe intracranial bleeding in infancy to mild bleeding episodes in the setting of surgery or trauma, were also highly endorsed by experts. It has been previously suggested that screening *F8* variants may be of limited value because results would not be available until after the first 7 days of life, the period of greatest risk for intracranial hemorrhage. However, the turnaround time for diagnostic genomic testing has significantly shortened in some settings, often within 1 to 2 days, signaling that the technical capabilities for rapid turnaround times are not far off.^57,58,59,60^ Furthermore, ascertainment of individuals at risk for hemophilia may improve surveillance and management of future bleeding episodes in less severe forms of disease. Our survey findings highlight a growing shift away from the historical goals of newborn screening and toward a more expansive view of the uses of genomic information to include not only conditions that require imminent treatment but possibly also those that may prompt changes in long-term risk ascertainment and outcomes.^61,62^

Ethical, legal, and social implications scholars have highlighted concerns in applying NBSeq to apparently healthy infants, including the uncertainties of variant interpretation, variable expressivity of disease phenotypes, and our nascent knowledge of genomics.^14,20,63,64^ Our results suggest that rare disease experts are largely supportive of NBSeq as a means for detecting additional disorders in newborns. Of note, younger experts were significantly more likely than older experts to agree with the statement that NBSeq should be integrated into newborn screening, suggesting that clinical experts who trained more recently are more open to the use of molecular screening tools in apparently healthy newborns.

### Limitations

This study has several limitations. The experts were primarily US based and not necessarily representative of the rare disease field. There were at least 2 types of selection bias: experts invited by the research team may be biased in favor of promoting NBSeq, and those invited may have been more likely to respond to the survey if they were in favor of NBSeq. Nonresponder bias was not quantified. The experts did not interact or participate in a process that would constitute formal consensus building. Some experts may not have been familiar with diagnostic or therapeutic developments for all gene-disease pairs that they responded to, leading them to indicate responses of “unsure,” thereby lowering rates of concordance. Survey respondents were not asked about practical considerations that would be necessary to actually implement NBSeq, such as cost, consent, and the relative scarcity of medical geneticists and other rare disease experts.^65,66^ For infants with positive results on NBSeq, standard operating procedures would need to be developed to facilitate appropriate care coordination between general pediatricians and specialists. Future studies will be needed to determine whether NBSeq is cost-effective and positively contributes to short- and long-term outcomes.

## Conclusions

Our findings in this survey study suggest that rare disease experts support expanding the number of genetic disorders included in newborn screening through NBSeq. The greatest degree of consensus occurred within clinical areas that are already included on the RUSP, such as metabolic and endocrine disorders, for which experts support using NBSeq to screen for disorders that lack other accurate or efficient biomarkers. Our findings also indicate a growing awareness that other types of disorders could be screened with NBSeq in healthy newborns to facilitate early diagnosis and surveillance. The genes with the highest concordance in this study may be used in future genome-first studies to screen research participants or other apparently healthy individuals. Over time, the gene list may need to be revisited due to the increasing number of therapies available for genetic conditions. Eventually, with appropriate infrastructure, NBSeq may be an efficient modality to keep pace with the growing number of emerging pharmacologic and gene-based therapies for rare disorders by identifying infants who would benefit from presymptomatic and early treatments.

## Notes

Supplement 1. **eTable 1.** Response Rates Computed According to AAPOR Standard Definitions **eAppendix.** Complete PDF of Survey **eFigure.** Methods and Results of Survey **eTable 2.** All Genes Included in Survey, in Order of Concordance **eTable 3.** Description of Characteristics of Genes Included in Survey **eTable 4.** Demographic Information of the Respondents **eTable 5.** Additional Genes Suggested for Inclusion by Respondents

Supplement 2.
Data Sharing Statement
