# Supplementary material for: Perspectives of Rare Disease Experts on Newborn Genome Sequencing
Source: JAMA Netw Open. 2023 May 8;6(5):e2312231. doi: 10.1001/jamanetworkopen.2023.12231 (PMC10167563; doi:10.1001/jamanetworkopen.2023.12231)
Supplement: Supplement 1. — eTable 1. Response Rates Computed According to AAPOR Standard Definitions eAppendix. Complete PDF of Survey eFigure. Methods and Results of Survey eTable 2. All Genes Included in Survey, in Order of Concordance eTable 3. Description of Characteristics of Genes Included in Survey eTable 4. Demographic Information of the Respondents eTable 5. Additional Genes Suggested for Inclusion by Respondents [file jamanetwopen-e2312231-s001.pdf]

Supplemental Online Content

Gold NB, Adelson SM, Sha N, et al. Perspectives of rare disease experts on newborn genome sequencing. *JAMA Netw Open*. 2023;6(5):e2312231. doi:10.1001/jamanetworkopen.2023.12231

eTable 1. Response Rates Computed According to AAPOR Standard Definitions

eAppendix. Complete PDF of Survey

eFigure. Methods and Results of Survey

eTable 2. All Genes Included in Survey, in Order of Concordance

eTable 3. Description of Characteristics of Genes Included in Survey

eTable 4. Demographic Information of the Respondents

eTable 5. Additional Genes Suggested for Inclusion by Respondents

This supplemental material has been provided by the authors to give readers additional information about their work.

© 2023 Gold NB et al. *JAMA Network Open.*

eTable 1. Response Rates Computed According to AAPOR Standard Definitions

**Returned questionnaire:**	**N**	**Unknown eligibility, "non-interview"**	**N**
Complete	181	Nothing known about respondent or address	0
Partial or break-off with sufficient information	57	No invitation sent	0
**Eligible, "Non-Interview"**	**N**	Nothing ever returned	0
Refusal	0	Invitation returned undelivered	21
Explicit refusal	0	Invitation returned with forwarding information	0
Implicit refusal	0	Other **unable to access survey*	1
Logged on to survey, did not complete any items	0	Returned from a unsampled email address	0
Read receipt confirmation, refusal	0	**Not Eligible, Returned**	**N**
Break-off or partial with insufficient information	0	Selected respondent screened out of sample **reported unable to take the survey due to lack of familiarity with subject matter*	12
Non-Contact	148		
Respondent was unavailable during field period	0		
Completed questionnaire, but not returned during field period	0	Quota filled	0
Other	0	Duplicate listing	2
Language barrier	0	Other	0
**Total Return Questionnaire**	238	**Total Unknown Eligibility + Not Eligible**	36
**Total Eligible**	386		
**Response Rate**	61.66%		

© 2023 Gold NB et al. *JAMA Network Open.*

Treatable Newborn Gene List Survey

Many clinicians and researchers in our field are exploring the notion of expanded newborn screening, i.e. the addition of genomic sequencing to identify treatable conditions that are not currently included on the Recommended Uniform Screening Panel (RUSP). Regardless of logistical or cost hurdles, which could be significant, we are interested in which genes experts like you think might be medically appropriate to evaluate and return in healthy newborns.

Instructions:

Part 1: Individual Genes (10-20 minutes, depending upon how many genes you elect to evaluate)

The first section of this survey includes 651 unique genes to select from that are classified by system (e.g. immunology, metabolism, etc.). Genes associated with conditions already listed on the RUSP are excluded. The genes you will be evaluating are all associated with conditions that at least some experts consider treatable. With each gene, we ask that you independently evaluate whether you think that this gene should be screened for in newborns, using any criteria you personally think is of importance.

As this is screening and not diagnostic testing, please assume that only well-established pathogenic variants would be "agged for follow-up. When evaluating each gene, you might consider factors such as whether a diagnostic non-molecular test is available, a clinically available treatment could improve outcomes or slow progression, and if infancy is the appropriate time to screen for this condition. Please do as many sections as you can and even if you need to answer "unsure" please try to complete all of the genes within one section.

Part 2: Exploratory Questions (5 minutes)

The second section of this survey includes questions about your opinions on inclusion criteria for newborn sequencing.

Part 3: Demographics (3 minutes)

The final section will collect demographic information on you.

In order to go back to the instruction menu, click "previous page." If you need to leave your computer before you are finished with the survey, please hit "Save & Return Later" at the bottom of the page. You will be given a unique code to enter when you return to finish the survey. If you exit the survey without hitting "Save & Return Later," you will have to start the survey again from the beginning. Thank you very much for your time.

Part 1: Individual Genes

Please check the box of each system of genes you would like to evaluate.

All systems ***(651 genes)*** ☐

Cardiovascular ***(17 genes)*** ☐

Endocrinology ***(95 genes)*** ☐

Gastroenterology ***(14 genes)*** ☐

Hematology ***(90 genes)*** ☐

Immunology ***(167 genes)*** ☐

Metabolism ***(137 genes)*** ☐

Nephrology ***(24 genes)*** ☐

Neurology ***(83 genes)*** ☐

Oncology ***(18 genes)*** ☐

© 2023 Gold NB et al. *JAMA Network Open.*

Ophthalmology ***(4 genes)*** ☐

Pulmonology ***(2 genes)*** ☐

Cardiovascular **(17 genes)**

Would you recommend screening for the following genes in newborns?

(Please scroll to the bottom of this page to leave any comments, questions or concerns)

	Yes	No	Unsure
APOA5 Apolipoprotein A-V deficiency	○	○	○
APOC2 Apolipoprotein C-II (apoC-II) deficiency	○	○	○
APOE Apolipoprotein (apo) E	○	○	○
DBH Orthostatic hypotension 1	○	○	○
CYB561 Orthostatic hypotension 2	○	○	○
ENPP1 Generalized arterial calcification of infancy 1	○	○	○
ABCC6 Generalized arterial calcification of infancy 2	○	○	○
LDLR Familial hypercholesterolemia 1	○	○	○
APOB Hypobetalipoproteinemia/Familial hypercholesterolemia 2	○	○	○
PCSK9 Familial hypercholesterolemia 3	○	○	○
LDLRAP1 Familial hypercholesterolemia 4	○	○	○
LMF1 Lipase maturation factor 1 (LMF1) deficiency	○	○	○
LMNA Hutchinson-Gilford progeria syndrome	○	○	○
LPL Lipoprotein lipase deficiency	○	○	○
SMAD4 Myhre syndrome	○	○	○
TAZ Barth Syndrome	○	○	○

© 2023 Gold NB et al. *JAMA Network Open.*

TTR<br>Transthyretin associated hereditary amyloidosis	○	○	○

Are there any genes we didn't include here that you think should be screened for in healthy newborns?

Other Comments?

Endocrinology (95 genes)

Would you recommend screening for the following genes in newborns?

(Please scroll to the bottom of this page to leave any comments, questions or concerns)

	Yes	No	Unsure
PDX1<br>Maturity-onset diabetes of the young, type 4	○	○	○
NEUROD1<br>Maturity-onset diabetes of the young, type 6	○	○	○
KLF11<br>Maturity-onset diabetes of the young, type 7	○	○	○
CEL<br>Maturity-onset diabetes of the young, type 8	○	○	○
PAX4<br>Maturity-onset diabetes of the young, type 9	○	○	○
INS<br>Maturity-onset diabetes of the young, type 10	○	○	○
APPL1<br>Maturity-onset diabetes of the young, type 14	○	○	○
GATA4<br>GATA4 associated diabetes	○	○	○
HNF1B<br>Renal cysts and diabetes syndrome	○	○	○
SLC19A2<br>Thiamine-responsive megaloblastic anemia syndrome with diabetes mellitus and sensorineural deafness	○	○	○
MNX1<br>MNX1 associated neonatal diabetes mellitus	○	○	○
NEUROG3<br>NEUROG3 associated neonatal diabetes mellitus	○	○	○

© 2023 Gold NB et al. *JAMA Network Open.*

NKX2-2<br>NKX2-2 associated neonatal diabetes mellitus	○	○	○
ABCC8<br>Familial hyperinsulinemic hypoglycemia-1; ABCC8 associated permanent neonatal diabetes mellitus	○	○	○
KCNJ11<br>Familial hyperinsulinemic hypoglycemia-2; KCNJ11 associated permanent neonatal diabetes mellitus	○	○	○
GCK<br>Familial hyperinsulinemic hypoglycemia 3	○	○	○
SLC16A1<br>Familial hyperinsulinemic hypoglycemia 7	○	○	○
AKT2<br>Hypoinsulinemic hypoglycemia	○	○	○
FOXA2<br>FOXA2 associated hyperinsulinism	○	○	○
HK1<br>HK1 associated hyperinsulinism	○	○	○
HNF1A<br>HNF1A associated hyperinsulinism	○	○	○
HNF4A<br>HNF4A associated hyperinsulinism	○	○	○
UCP2<br>UCP2 associated hyperinsulinism	○	○	○
EIF2AK3<br>Wolcott-Rallison syndrome	○	○	○
RFX6<br>Mitchell-Riley syndrome	○	○	○
GATA6<br>Pancreatic agenesis and congenital heart defects	○	○	○
PTF1A<br>Pancreatic agenesis 2	○	○	○
AQP2<br>Nephrogenic diabetes insipidus	○	○	○
AVPR2<br>X-linked nephrogenic diabetes insipidus	○	○	○
AVP<br>Neurohypophyseal diabetes insipidus	○	○	○
AGPAT2<br>Congenital generalized lipodystrophy type 1	○	○	○

© 2023 Gold NB et al. *JAMA Network Open.*

BSCL2
Congenital generalized lipodystrophy type 2

CAV1
Congenital generalized lipodystrophy type 3

CAVIN1
Congenital generalized lipodystrophy type 4

ABCC9
ABCC9 associated hypertrichotic osteochondrodysplasia

KCNJ8
KCNJ8 associated hypertrichotic osteochondrodysplasia

CA2
Osteopetrosis with renal tubular acidosis

TCIRG1
Osteopetrosis type 1

TNFRSF11A
Osteopetrosis type 7

SNX10
Osteopetrosis type 8

FAM111A
Kenny-Ca#ey syndrome, type 2

CYP11B2
Aldosterone synthase deficiency

CACNA1D
Primary aldosteronism with seizures and neurologic abnormalities

CLCN2
Familial hyperaldosteronism, Type II

KCNJ5
Familial hyperaldosteronism, Type III

CACNA1H
Familial hyperaldosteronism, Type IV

WNK4
Pseudohypoaldosteronism, type IIB

WNK1
Pseudohypoaldosteronism, type IIC

KLHL3
Pseudohypoaldosteronism, type IID

CUL3
Pseudohypoaldosteronism, type IIE

© 2023 Gold NB et al. *JAMA Network Open.*

NR3C2
NR3C2 associated pseudohypoaldosteronism, type I

SCNN1A
SCNN1A associated pseudohypoaldosteronism, type I

SCNN1B
SCNN1B associated pseudohypoaldosteronism, type I

SCNN1G
SCNN1G associated pseudohypoaldosteronism, type I

CYP11A1
Adrenal insu$ciency, congenital, with 46XY sex reversal, partial or complete

POMC
Obesity, adrenal insu$ciency, and red hair due to POMC deficiency

CYP17A1
17-alpha-hydroxylase/17,20-lyase deficiency

CYP11B1
Congenital adrenal hyperplasia due to 11-beta-hydroxylase deficiency

HSD3B2
Adrenal hyperplasia, congenital, due to 3-beta-hydroxysteroid dehydrogenase 2 deficiency

STAR
Lipoid adrenal hyperplasia

NR5A1
NR5A1 associated adrenocortical insu$ciency

SAMD9
MIRAGE syndrome

MC2R
Glucocorticoid deficiency due to ACTH unresponsiveness

MRAP
Glucocorticoid deficiency 2

NNT
Glucocorticoid deficiency 4, with or without mineralocorticoid deficiency

HSD11B2
Apparent mineralocorticoid excess

TBX19
Adrenocorticotropic hormone deficiency

CYP27B1
Vitamin D-dependent rickets, type IA

© 2023 Gold NB et al. *JAMA Network Open.*

CYP2R1 Vitamin D-dependent rickets, type IB	○	○	○
VDR Vitamin D-dependent rickets, type 2A	○	○	○
PHEX X-linked dominant hypophosphatemic rickets	○	○	○
SLC34A3 Hypophosphatemic rickets with hypercalciuria	○	○	○
ALPL Hypophosphatasia	○	○	○
GH1 Isolated growth hormone deficiency type 1A, type 1B, type 2	○	○	○
GHRHR Isolated growth hormone deficiency type 4	○	○	○
RNPC3 RNPC3 associated growth hormone deficiency	○	○	○
GHR Growth hormone receptor deficiency	○	○	○
IGF1 Insulin-like growth factor I deficiency	○	○	○
GPR101 Growth hormone-secreting pituitary adenoma 2	○	○	○
IGFALS Acid-labile subunit deficiency	○	○	○
PAPPA2 PAPPA2 associated short stature	○	○	○
POU1F1 Combined pituitary hormone deficiency 1	○	○	○
PROP1 Combined pituitary hormone deficiency 2	○	○	○
LHX3 Combined pituitary hormone deficiency 3	○	○	○
LHX4 Combined pituitary hormone deficiency 4	○	○	○
HESX1 Combined pituitary hormone deficiency 5	○	○	○

© 2023 Gold NB et al. *JAMA Network Open.*

LEP
Leptin deficiency

LEPR
Leptin receptor deficiency

GNAS
GNAS associated Pseudohypoparathyroidism

FOXE1
Bamforth-Lazarus syndrome

SOX3
X-linked panhypopituitarism

WFS1
Wolfram syndrome 1

AAAS
Achalasia-addisonianism-alacrimia syndrome

AIRE
Autoimmune polyendocrinopathy syndrome, type I, with or without reversible metaphyseal dysplasia

PCSK1
Obesity with impaired prohormone processing

Are there any genes we didn't include here that you think should be screened for in healthy newborns?

Other Comments?

Gastroenterology (14 genes)

Would you recommend screening for the following genes in newborns?

(Please scroll to the bottom of this page to leave any comments, questions or concerns)

	Yes	No	Unsure
HSD3B7 Congenital bile acid synthesis defect type 1			
AKR1D1 Congenital bile acid synthesis defect type 2			
CYP7B1 Congenital bile acid synthesis defect type 3			
LARS1 LARS1 associated Infantile liver failure syndrome 1			
TRMU Transient infantile liver failure			

© 2023 Gold NB et al. *JAMA Network Open.*

MTTP
Abetalipoproteinemia

IL10RA
In%ammatory bowel disease 25

IL10RB
In%ammatory bowel disease 28

IL12RB1
In%ammatory bowel disease 25, early onset, autosomal recessive

SLC26A3
Congenital secretory chloride diarrhea

SLC9A3
Congenital secretory sodium diarrhea

DGAT1
Diarrhea 7, protein-losing enteropathy type

GPIHBP1
Glycosylphosphatidylinositol-anchored high-density lipoprotein-binding protein 1 (GPIHBP1) deficiency

SAR1B
Chylomicron retention disease

Are there any genes we didn't include here that you think should be screened for in healthy newborns?

Other Comments?

Hematology (90 genes)

Would you recommend screening for the following genes in newborns?

(Please scroll to the bottom of this page to leave any comments, questions or concerns)

	Yes	No	Unsure
ALAS2 X-linked erythropoietic protoporphyria			
UROD Porphyria cutanea tarda			
ALAD Aminolevulinic acid dehydratase deficiency porphyria			
CPOX Coproporphyria			
FECH Erythropoietic protoporphyria 1			

© 2023 Gold NB et al. *JAMA Network Open.*

HMBS<br>Acute intermittent porphyria	○	○	○
PPOX<br>Variegate porphyria	○	○	○
DNA JC21<br>Bone marrow failure syndrome 3	○	○	○
MYSM1<br>Bone marrow failure syndrome 4	○	○	○
GATA1<br>GATA1 associated X-Linked Cytopenia	○	○	○
SAMD9L<br>Ataxia-pancytopenia syndrome	○	○	○
LYST<br>Chediak-Higashi Syndrome	○	○	○
SBDS<br>Shwachman-Diamond syndrome	○	○	○
EFL1<br>Shwachman-Diamond syndrome 2	○	○	○
SRP54<br>SRP54 associated Shwachman-Diamond syndrome	○	○	○
FANCA<br>Fanconi anemia, complementation group A	○	○	○
FANCB<br>Fanconi anemia, complementation group B	○	○	○
FANCC<br>Fanconi anemia, complementation group C	○	○	○
BRCA2<br>Fanconi anemia, complementation group D1	○	○	○
FANCD2<br>Fanconi anemia, complementation group D2	○	○	○
FANCE<br>Fanconi anemia, complementation group E	○	○	○
FANCF<br>Fanconi anemia, complementation group F	○	○	○
FANCG<br>Fanconi anemia, complementation group G	○	○	○
FANCI<br>Fanconi anemia, complementation group I	○	○	○

© 2023 Gold NB et al. *JAMA Network Open.*

BRIP1
Fanconi anemia, complementation group J

FANCL
Fanconi anemia, complementation group L

PALB2
Fanconi anemia, complementation group N

RAD51C
Fanconi amenia, complementation group O

SLX4
Fanconi anemia, complementation group P

ERCC4
Fanconi anemia, complementation group Q

BRCA1
Fanconi anemia, complementation group S

UBE2T
Fanconi anemia, complementation group T

MAD2L2
Fanconi anemia, complementation group V

RFWD3
Fanconi anemia, complementation group W

RPS19
Diamond-Blackfan anemia 1

RPS24
Diamond-Blackfan anemia 3

RPS17
Diamond-Blackfan anemia 4

RPL35A
Diamond-Blackfan anemia 5

RPL5
Diamond-Blackfan anemia 6

RPL11
Diamond-Blackfan anemia 7

RPS7
Diamond-Blackfan anemia 8

RPS10
Diamond-Blackfan anemia 9

RPS26
Diamond-Blackfan anemia 10

© 2023 Gold NB et al. *JAMA Network Open.*

RPL26 Diamond-Blackfan anemia 11	○	○	○
RPL15 Diamond-Blackfan anemia 12	○	○	○
RPS29 Diamond-Blackfan anemia 13	○	○	○
TSR2 Diamond-Blackfan anemia 14 with mandibulofacial dysostosis	○	○	○
RPS28 Diamond Blackfan anemia 15 with mandibulofacial dysostosis	○	○	○
RPL27 Diamond-Blackfan anemia 16	○	○	○
RPS27 Diamond-Blackfan anemia 17	○	○	○
RPL18 Diamond-Blackfan anemia 18	○	○	○
RPL35 Diamond-Blackfan anemia 19	○	○	○
RPS15A Diamond-Blackfan anemia 20	○	○	○
RPL31 RPL31 associated Diamond-Blackfan anemia	○	○	○
G6PD Hemolytic anemia due to G6PD deficiency	○	○	○
SLC19A1 Folate dependent megaloblastic anemia	○	○	○
SLC46A1 Hereditary folate malabsorption	○	○	○
TF Atransferrinemia	○	○	○
SLC25A38 Pyridoxine-refractory sideroblastic anemia 2	○	○	○
NBN Nijmegen breakage syndrome	○	○	○
CBLIF Intrinsic factor deficiency	○	○	○
RTEL1 Dyskeratosis congenita	○	○	○
TERC Dyskeratosis congenita, autosomal dominant 1	○	○	○
TINF2 Dyskeratosis congenita, autosomal dominant 3	○	○	○

© 2023 Gold NB et al. *JAMA Network Open.*

DKC1
Dyskeratosis congenita, X-linked

ELANE
ELANE associated neutropenia 1

VPS45
Severe congenital neutropenia 5

F8
Hemophilia A

F9
Hemophilia B

F13A1
Factor XIIIA deficiency

F13B
Factor XIIIB deficiency

GGCX
Combined deficiency of vitamin K-dependent clotting factors 1

VKORC1
Combined deficiency of vitamin K-dependent clotting factors 2

FGA
FGA associated afibrinogenemia

FGB
FGB associate afibrinogenemia

FGG
FGG associated afibrinogenemia

HOXA11
Radioulnar synostosis with amegakaryocytic thrombocytopenia 1

MECOM
Radioulnar synostosis with amegakaryocytic thrombocytopenia 2

MPL
Congenital amegakaryocytic thrombocytopenia

WDR1
Periodic fever, immunodeficiency, and thrombocytopenia syndrome

ADAMTS13
Familial thrombotic thrombocytopenic purpura

AP3B1
Hermansky-Pudlak syndrome 2

HFE
Hemochromatosis type 1

HJV
Hemochromatosis, type 2A

© 2023 Gold NB et al. *JAMA Network Open.*

	Yes	No	Unsure
HAMP<br>Hemochromatosis, type 2B	○	○	○
TFR2<br>Hemochromatosis, type 3	○	○	○
SLC40A1<br>Hemochromatosis, type 4	○	○	○
HBA1<br>Alpha-thalassemia	○	○	○
HBA2<br>Alpha-thalassemia	○	○	○
PIK3CA<br>PIK3CA related overgrowth spectrum	○	○	○

Are there any genes we didn't include here that you think should be screened for in healthy newborns?

Other Comments?

Immunology (167 genes)

Would you recommend screening for the following genes in newborns?

(Please scroll to the bottom of this page to leave any comments, questions or concerns)

	Yes	No	Unsure
CORO1A<br>Immunodeficiency 8	○	○	○
ORAI1<br>Immunodeficiency 9	○	○	○
STIM1<br>Immunodeficiency 10	○	○	○
MALT1<br>Immunodeficiency 12	○	○	○
PIK3CD<br>Immunodeficiency 14	○	○	○
IKBKB<br>Immunodeficiency 15, 15B	○	○	○
CD3E<br>Immunodeficiency 18	○	○	○
CD3D<br>Immunodeficiency 19	○	○	○
GATA2<br>Immunodeficiency 21	○	○	○
LCK<br>Immunodeficiency 22	○	○	○
PGM3<br>Immunodeficiency 23	○	○	○

© 2023 Gold NB et al. *JAMA Network Open.*

CTPS1<br>Immunodeficiency 24	○	○	○
CD247<br>Immunodeficiency 25	○	○	○
PRKDC<br>Immunodeficiency 26	○	○	○
IFNGR2<br>Immunodeficiency 27A	○	○	○
IFNGR1<br>Immunodeficiency 27B	○	○	○
IL17RA<br>Immunodeficiency 30	○	○	○
STAT1<br>Immunodeficiency 31B	○	○	○
IRF8<br>Immunodeficiency 32B	○	○	○
TYK2<br>Immunodeficiency 35	○	○	○
IL2RA<br>Immunodeficiency 41 with lymphoproliferation and ○ autoimmunity	○	○	
ZAP70<br>Immunodeficiency 48	○	○	○
RELB<br>Immunodeficiency 53	○	○	○
MCM4<br>Immunodeficiency 54	○	○	○
IL21R<br>Immunodeficiency 56	○	○	○
IL2RB<br>Immunodeficiency 63 with lymphoproliferation and autoimmunity	○	○	○
RASGRP1<br>Immunodeficiency 64	○	○	○
CD40LG<br>X-linked immunodeficiency with hyper-IgM type 1	○	○	○
AICDA<br>Immunodeficiency with hyper-IgM, type 2	○	○	○
CD40<br>Immunodeficiency with hyper-IgM, type 3	○	○	○
UNG<br>Immunodeficiency with hyper IgM, type 5	○	○	○

© 2023 Gold NB et al. *JAMA Network Open.*

DNMT3B<br>Immunodeficiency-centromeric instability-facial anomalies syndrome 1	○	○	○
ZBTB24<br>Immunodeficiency-centromeric instability-facial anomalies syndrome 2	○	○	○
CDCA7<br>Immunodeficiency-centromeric instability-facial anomalies syndrome 3	○	○	○
HELLS<br>Immunodeficiency-centromeric instability-facial anomalies syndrome 4	○	○	○
DCLRE1C<br>Omenn syndrome/Severe combined immunodeficiency, Athabascan type	○	○	○
FOXN1<br>T-cell immunodeficiency with congenital alopecia and nail dystrophy	○	○	○
LAMTOR2<br>MAPBP-interacting protein associated immunodeficiency	○	○	○
LIG1<br>LIG1 associated immunodeficiency	○	○	○
MAGT1<br>X-linked Immunodeficiency with magnesium defect, Epstein-Barr virus infection and neoplasia	○	○	○
MAP3K14<br>MAP3K14 associated immunodeficiency	○	○	○
MTHFD1<br>Combined immunodeficiency and megaloblastic anemia with or without hyperhomocysteinemia	○	○	○
NFE2L2<br>NRF2 superactivity (immunodeficiency, developmental delay, and hypohomocysteinemia)	○	○	○
NFKBIA<br>Ectodermal dysplasia and immunodeficiency 2	○	○	○
RAG2<br>RAG2 associated T cell-negative, B cell-negative, severe combined immunodeficiency	○	○	○
SP110<br>Hepatic venoocclusive disease with immunodeficiency	○	○	○

© 2023 Gold NB et al. *JAMA Network Open.*

STAT5B Growth hormone insensitivity with immunodeficiency	○	○	○
STK4 STK4 associated T-cell immunodeficiency, recurrent infections, autoimmunity, and cardiac malformations	○	○	○
TRNT1 Sideroblastic anemia with B-cell immunodeficiency, periodic fevers, and developmental delay	○	○	○
TTC7A Gastrointestinal defects and immunodeficiency syndrome	○	○	○
CARD11 B-cell expansion with NKFB and T-cell anergy/Immunodeficiency 11B with atopic dermatitis	○	○	○
CUBN Imerslund-Grasbeck syndrome 1	○	○	○
AMN Imerslund-Grasbeck syndrome 2	○	○	○
IGHM Agammaglobulinemia 1	○	○	○
IGLL1 Agammaglobulinemia 2	○	○	○
CD79A Agammaglobulinemia 3	○	○	○
BLNK Agammaglobulinemia 4	○	○	○
CD79B Agammaglobulinemia 6	○	○	○
PIK3R1 Agammaglobulinemia 7	○	○	○
TCF3 Agammaglobulinemia 8	○	○	○
BTK X-linked agammaglobulinemia	○	○	○
C1QA C1QA associated C1q deficiency	○	○	○
C1QB C1QB associated C1q deficiency	○	○	○
C1QC C1QC associated C1q deficiency	○	○	○
C2 C2 deficiency	○	○	○
C3 C3 deficiency	○	○	○

© 2023 Gold NB et al. *JAMA Network Open.*

C5<br>C5 deficiency	○	○	○
C6<br>C6 deficiency	○	○	○
C7<br>C7 deficiency	○	○	○
C8A<br>C8 deficiency, type I	○	○	○
C8B<br>C8 deficiency, type II	○	○	○
C9<br>C9 deficiency	○	○	○
ICOS<br>Common variable immune deficiency 1	○	○	○
TNFRSF13B<br>Common variable immune deficiency 2	○	○	○
CD19<br>Common variable immune deficiency 3	○	○	○
TNFRSF13C<br>Common variable immune deficiency 4	○	○	○
MS4A1<br>Common variable immune deficiency 5	○	○	○
CD81<br>Common variable immune deficiency 6	○	○	○
CR2<br>Common variable immune deficiency 7	○	○	○
LRBA<br>Common variable immune deficiency 8	○	○	○
NFKB2<br>Common variable immune deficiency 10	○	○	○
IL21<br>Common variable immune deficiency 11	○	○	○
NFKB1<br>Common variable immune deficiency 12	○	○	○
IKZF1<br>Common variable immune deficiency 13	○	○	○

© 2023 Gold NB et al. *JAMA Network Open.*

IRF2BP2
Common variable immune deficiency 14

ITK
Lymphoproliferative syndrome 1

CD27
Lymphoproliferative syndrome 2

CD70
Lymphoproliferative syndrome 3

PRKCD
Autoimmune lymphoproliferative syndrome, type III

CTLA4
Autoimmune lymphoproliferative syndrome, type V

SH2D1A
X-linked lymphoproliferative syndrome 1

XIAP
X-linked lymphoproliferative syndrome 2

CFHR1
CFHR1 associated susceptibility to atypical hemolytic uremic syndrome

CD46
Susceptibility to atypical hemolytic uremic syndrome 2

THBD
Susceptibility to atypical hemolytic uremic syndrome 6

CFB
Complement factor B deficiency

CFD
Complement factor D deficiency

CFH
Complement factor H deficiency

CFI
Complement factor I deficiency

CIITA
Bare lymphocyte syndrome, type II, complementation group A

RFX5
Bare lymphocyte syndrome, type II, complementation group C and group E

RFXAP
Bare lymphocyte syndrome, type II, complementation group D

COL7A1
Epidermolysis bullosa

© 2023 Gold NB et al. *JAMA Network Open.*

KRT14
Epidermolysis bullosa

KRT5
Epidermolysis bullosa

GFI1
Severe congenital neutropenia 2

HAX1
Severe congenital neutropenia 3

G6PC3
Severe congenital neutropenia 4

JAGN1
Severe congenital neutropenia 6

CSF3R
Severe congenital neutropenia 7

CYBA
CYBA associated chronic granulomatous disease

CYBB
X-linked chronic granulomatous disease

CYBC1
CYBC1 associated chronic granulomatous disease

NCF1
NCF1 associated chronic granulomatous disease

NCF2
NCF2 associated chronic granulomatous disease

NCF4
NCF4 associated chronic granulomatous disease

DOCK2
DOCK2 defeciency

DOCK8
DOCK8 deficiency

ITGB2
Leukocyte adhesion deficiency, type I

FERMT3
Leukocyte adhesion deficiency, type III

IL10
Interleukin-10 deficiency

IL1RN
Interleukin 1 receptor antagonist deficiency

NLRC4
NLRC4 associated familial cold in%ammatory syndrome

© 2023 Gold NB et al. *JAMA Network Open.*

NLRP12
Familial cold autoin%ammatory syndrome 2

PRF1
Familial hemophagocytic lymphohistiocytosis 2

UNC13D
Familial hemophagocytic lymphohistiocytosis 3

STX11
Familial hemophagocytic lymphohistiocytosis 4

STXBP2
Familial hemophagocytic lymphohistiocytosis 5

MVK
Hyper-IgD syndrome / mevalonate kinase deficiency

STAT3
Hyper-IgE recurrent infection syndrome

PSTPIP1
PSTPIP1 associated in%ammatory disease

RAB27A
Griscelli syndrome, type 2

RFXANK
MHC class II deficiency, complementation group B

RMRP
Cartilage-hair hypoplasia

SMARCD2
Specific granule deficiency 2

TNFAIP3
TNFAIP3 associated autoin%ammatory syndrome

TNFRSF1A
Tumor necrosis factor receptor associated periodic syndrome

USP18
Pseudo-TORCH syndrome 2

WAS
WAS associated disorder

WIPF1
Wiskott-Aldrich syndrome 2

ADA2
Vasculitis, autoin%ammation, immunodeficiency, and hematologic defects syndrome

© 2023 Gold NB et al. *JAMA Network Open.*

AK2
Reticular dysgenesis

ACP5
Spondyloenchondrodysplasia with ACP5 immune dysregulation

ARPC1B
Platelet abnormalities with eosinophilia and immune-mediated in%ammatory disease

C1NH
Hereditary angioedema

CARD14
Pityriasis rubra pilaris

CARD9
Candidiasis, familial

CDKN1C
IMAGE syndrome

CFP
X-linked properdin deficiency

CXCR4
WHIM syndrome

FOXP3
X-linked immunodysregulation, polyendocrinopathy, and enteropathy

IL36RN
Pustular psoriasis 14

IRAK4
IRAK4 deficiency

KDSR
Erythrokeratodermia variabilis et progressiva 4

LIG4
LIG4 syndrome

LPIN2
Majeed syndrome

MARS1
MARS1 associated interstitial lung and liver disease

MEFV
Familial Mediterranean fever

MYD88
MYD88 deficiency

NIPAL4
Ichthyosis, congenital, autosomal recessive 6

NLRP3
Cryopyrin associated periodic fever syndrome

© 2023 Gold NB et al. *JAMA Network Open.*

NOD2
Blau syndrome

OTULIN
OTULIN deficiency

PARN
Dyskeratosis congenita, autosomal recessive 6

PAX1
Otofaciocervical syndrome 2

PLCG2
Autoin%ammation and PLCG2 associated antibody deficiency and immune dysregulation (APLAID)

PNP
Purine nucleoside phosphorylase deficiency

Are there any genes we didn't include here that you think should be screened for in healthy newborns?

Other Comments?

Metabolism (137 genes)

Would you recommend screening for the following genes in newborns?

(Please scroll to the bottom of this page to leave any comments, questions or concerns)

	Yes	No	Unsure
ABCG5 Sitosterolemia 1			
ABCG8 Sitosterolemia 2			
G6PC Glycogen storage disease Ia			
SLC37A4 Glycogen storage disease Ib			
AGL Glycogen storage disease III			
PHKA2 Glycogen storage disease, type IXa			
PHKB Glycogen storage disease, type IXb			
PHKG2 Glycogen storage disease, type IXc			
PHKA1 Glycogen storage disease, type IXd			
PYGL Glycogen storage disease VI			

© 2023 Gold NB et al. *JAMA Network Open.*

IDS
Mucopolysaccharidosis II

SGSH
Mucopolysaccharidosis type IIIA (Sanfilippo A)

NAGLU
Mucopolysaccharidosis type IIIB

HGSNAT
Mucopolysaccharidosis type IIIC (Sanfilippo C)

GALNS
Mucopolysaccharidosis IVA

ARSB
Mucopolysaccharidosis type VI

GUSB
Mucopolysaccharidosis type VII

GNPTA
I-Cell Disease

GALC
Krabbe disease

SMPD1
Niemann-Pick disease, type A and type B

NPC1
Niemann-Pick disease, type C, NPC1

NPC2
Niemann-Pick disease, type C, NPC2

HEXA
Tay-Sachs disease

HEXB
Sandho# disease, infantile, juvenile, and adult forms

FUCA1
Fucosidosis

GBA
Gaucher disease, type I

GLA
Fabry disease

PPT1
Ceroid lipofuscinosis, neuronal, 1

TPP1
Neuronal ceroid lipofuscinosis 2

MFSD8
Ceroid lipofuscinosis, neuronal, 7

COQ2
Primary coenzyme Q10 deficiency 1

© 2023 Gold NB et al. *JAMA Network Open.*

PDSS1<br>Primary coenzyme Q10 deficiency 2	○	○	○
PDSS2<br>Primary coenzyme Q10 deficiency 3	○	○	○
COQ8A<br>Primary coenzyme Q10 deficiency 4	○	○	○
COQ9<br>Primary coenzyme Q10 deficiency 5	○	○	○
COQ6<br>Primary coenzyme Q10 deficiency 6	○	○	○
COQ4<br>Primary coenzyme Q10 deficiency 7	○	○	○
COQ7<br>Primary coenzyme Q10 deficiency 8	○	○	○
COQ5<br>Coenzyme Q5 methyltransferase deficiency	○	○	○
MT-CO1<br>MELAS (Myopathy, Encephalopathy, Lactic Acidosis, and Stroke-like episodes)	○	○	○
MT-CO3<br>MELAS (Myopathy, Encephalopathy, Lactic Acidosis, and Stroke-like episodes)	○	○	○
MT-CPO2<br>MELAS (Myopathy, Encephalopathy, Lactic Acidosis, and Stroke-like episodes)	○	○	○
MT-ND1<br>MELAS (Myopathy, Encephalopathy, Lactic Acidosis, and Stroke-like episodes)	○	○	○
MT-ND4<br>MELAS (Myopathy, Encephalopathy, Lactic Acidosis, and Stroke-like episodes)	○	○	○
MT-ND5<br>MELAS (Myopathy, Encephalopathy, Lactic Acidosis, and Stroke-like episodes)	○	○	○
MT-ND6<br>MELAS (Myopathy, Encephalopathy, Lactic Acidosis, and Stroke-like episodes)	○	○	○
MT-TF<br>MELAS (Myopathy, Encephalopathy, Lactic Acidosis, and Stroke-like episodes)	○	○	○

© 2023 Gold NB et al. *JAMA Network Open.*

MT-TH
MELAS (Myopathy, Encephalopathy, Lactic Acidosis, and Stroke-like episodes)

MT-TL1
MELAS (Myopathy, Encephalopathy, Lactic Acidosis, and Stroke-like episodes)

MT-TQ
MELAS (Myopathy, Encephalopathy, Lactic Acidosis, and Stroke-like episodes)

MT-TS1
MELAS (Myopathy, Encephalopathy, Lactic Acidosis, and Stroke-like episodes)

MT-TS2
MELAS (Myopathy, Encephalopathy, Lactic Acidosis, and Stroke-like episodes)

MT-TW
MELAS (Myopathy, Encephalopathy, Lactic Acidosis, and Stroke-like episodes)

ACAD9
Mitochondrial complex I deficiency nuclear type 20

ACAT1
Mitochondrial acetoacetyl-CoA thiolase deficiency

ECHS1
Mitochondrial short-chain enoyl-CoA hydratase-1 deficiency

ETHE1
Mitochondrial sulfur dioxygenase deficiency

TK2
Thymidine kinase deficiency

DLAT
Pyruvate dehydrogenase deficiency

PDHA1
Pyruvate dehydrogenase deficiency

PDHB
Pyruvate dehydrogenase deficiency

PDHX
Pyruvate dehydrogenase deficiency

PDP1
Pyruvate dehydrogenase phosphatase deficiency

© 2023 Gold NB et al. *JAMA Network Open.*

PKLR
Pyruvate kinase deficiency

TRPM6
TRPM6 associated hypomagnesemia

FXYD2
Hypomagnesemia, type 2

MPI
Congenital disorder of glycosylation, type Ib

PGM1
Congenital disorder of glycosylation, type It

SLC35A2
Congenital disorder of glycosylation, type IIm

SLC39A8
Congenital disorder of glycosylation, type IIn

TMEM165
Congenital disorder of glycosylation, type IIk

PIGA
PIGA-CDG

PIGM
PIGM-CDG

PIGO
PIGO-CDG

AMT
Glycine encephalopathy due to aminomethyltransferase (AMT)

OAT
Ornithine aminotransferase deficiency

OTC
Ornithine transcarbamylase deficiency

GLUD1
Hyperinsulinism-hyperammonemia syndrome

UMPS
Orotic aciduria

SLC19A3
Hyperornithinemia-hyperammonemia-homocitrullinuria syndrome

SLC25A15
Hyperornithinemia-hyperammonemia-homocitrullinuria syndrome

© 2023 Gold NB et al. *JAMA Network Open.*

SLC25A19
Thiamine metabolism dysfunction syndrome 4

TPK1
Thiamine metabolism dysfunction syndrome 5

SLC2A1
GLUT1 deficiency syndrome 1

SLC35C1
GLUT1 deficiency syndrome 1

SLC6A8
Creatine transporter deficiency

GAMT
Cerebral creatine deficiency syndrome 2

GATM
Cerebral creatine deficiency syndrome 3

ALDH5A1
Succinic semialdehyde dehydrogenase deficiency

SLC30A10
Hypermagnesemia with dystonia 1

SLC39A14
Hypermagnesemia with dystonia 2

ALDOB
Hereditary fructose intolerance

FBP1
Fructose-1,6-bisphosphatase deficiency

GALM
Galactose mutarotase deficiency

SLC5A1
Glucose-galactose malabsorption

HIBCH
3-hydroxyisobutryl-CoA hydrolase deficiency

HMGCS2
3-hydroxy-3-methylglutaryl-CoA synthase deficiency

MTHFR
Methylenetetrahydrofolate reductase deficiency

MTHFS
5,10-Methenyltetrahydrofolate synthetase deficiency

© 2023 Gold NB et al. *JAMA Network Open.*

DDC
Aromatic amino acid decarboxylase deficiency

GLDC
Glycine decarboxylase (GLDC) deficiency

PHGDH
Phosphoglycerate dehydrogenase deficiency

MLYCD
Malonyl-CoA decarboxylase deficiency

SLC30A2
Transient neonatal zinc deficiency

SLC39A4
Acrodermatitis enteropathica

SLC7A7
Lysinuric protein intolerance

SORD
Sorbitol dehydrogenase deficiency with peripheral neuropathy

TCN2
Transcobalamin II deficiency

AGA
Aspartylglucosaminidase deficiency

AGXT
Primary hyperoxaluria type I

ALDH4A1
Hyperprolinemia, type II

APRT
Adenine phosphoribosyltransferase deficiency

ATP7A
Menkes disease

CP
Aceruloplasminemia

ATP7B
Wilson disease

BCKDK
Branched-chain ketoacid dehydrogenase kinase deficiency

CA5A
Carbonic anhydrase VA deficiency

CPS1
Carbamoyl phosphate synthetase I deficiency

CYP27A1
Cerebrotendinous xanthomatosis

© 2023 Gold NB et al. *JAMA Network Open.*

DHCR7<br>7-dehydrocholesterol reductase deficiency	○	○	○
DHFR<br>Dihydrofolate reductase deficiency	○	○	○
DLD<br>Dihydrolipoamide dehydrogenase deficiency	○	○	○
GLUL<br>Glutamine synthetase deficiency	○	○	○
GOT2<br>Glutamic-oxaloacetic transaminase 2 deficiency	○	○	○
IARS1<br>Isoleucyl-tRNA synthetase deficiency	○	○	○
LIPA<br>Lysosomal acid lipase deficiency	○	○	○
MAN2B1<br>Alpha-mannosidosis	○	○	○
MOCS1<br>Molybdenum cofactor deficiency A	○	○	○
NAGS<br>N-acetylglutamate synthase deficiency	○	○	○
NAXE<br>NAD(P)HX epimerase deficiency	○	○	○
OXCT1<br>Succinyl-CoA:3-ketoacid CoA transferase (SCOT) deficiency	○	○	○
PNPO<br>Pyridoxamine 5-prime-phosphate oxidase deficiency	○	○	○
POR<br>Cytochrome P450 oxidoreductase deficiency	○	○	○
PSAT1<br>Phosphoserine aminotransferase deficiency	○	○	○
PSPH<br>Phosphoserine phosphatase deficiency	○	○	○
SI<br>Congenital sucrase-isomaltase deficiency	○	○	○
AP1S1<br>MEDNIK syndrome	○	○	○

Are there any genes we didn't include here that you think should be screened for in healthy newborns? [ ]

© 2023 Gold NB et al. *JAMA Network Open.*

Other Comments?

Nephrology (24 genes)

Would you recommend screening for the following genes in newborns?

(Please scroll to the bottom of this page to leave any comments, questions or concerns)

	Yes	No	Unsure
ATP6V0A4 ATP6V0A4 associated distal renal tubular acidosis	◯	◯	◯
ATP6V1B1 ATP6V1B1 associated distal renal tubular acidosis	◯	◯	◯
FOXI1 FOXI1 associated distal renal tubular acidosis	◯	◯	◯
SLC4A1 SLC4A1 associated distal renal tubular acidosis	◯	◯	◯
WDR72 WDR72 associated distal renal tubular acidosis	◯	◯	◯
SLC4A4 SLC4A4 associated proximal renal tubular acidosis	◯	◯	◯
SLC12A1 Bartter syndrome, type 1	◯	◯	◯
KCNJ1 Bartter syndrome, type 2	◯	◯	◯
CLCNKB Bartter syndrome, type 3	◯	◯	◯
BSND Bartter syndrome, type 4a	◯	◯	◯
MAGED2 Bartter syndrome, type 5	◯	◯	◯
COL4A4 Alport syndrome 2	◯	◯	◯
COL4A3 Alport syndrome 3	◯	◯	◯
COL4A5 X-linked Alport syndrome 1	◯	◯	◯
COQ8B Nephrotic syndrome, type 9	◯	◯	◯

© 2023 Gold NB et al. *JAMA Network Open.*

SGPL1 Nephrotic syndrome, type 14	◯	◯	◯
GRHPR Primary hyperoxaluria type III	◯	◯	◯
HOGA1 Primary hyperoxaluria type III	◯	◯	◯
PKD1 Polycystic kidney disease 1	◯	◯	◯
PKD2 Polycystic kidney disease 2	◯	◯	◯
PMM2 Polycystic kidney disease with hyperinsulinemic hypoglycemia	◯	◯	◯
SLC12A3 Gitelman syndrome	◯	◯	◯
CA12 Isolated hyperchlorhidrosis	◯	◯	◯
CTNS Cystinosis	◯	◯	◯

Are there any genes we didn't include here that you think should be screened for in healthy newborns?

Other Comments?

Neurology (83 genes)

Would you recommend screening for the following genes in newborns?

(Please scroll to the bottom of this page to leave any comments, questions or concerns)

	Yes	No	Unsure
TREX1 Aicardi-Goutieres syndrome 1	◯	◯	◯
RNASEH2B Aicardi-Goutieres syndrome 2	◯	◯	◯
RNASEH2C Aicardi-Goutieres syndrome 3	◯	◯	◯
RNASEH2A Aicardi-Goutieres syndrome 4	◯	◯	◯
SAMHD1 Aicardi-Goutieres syndrome 5	◯	◯	◯
ADAR Aicardi-Goutieres syndrome 6	◯	◯	◯
IFIH1 Aicardi-Goutieres syndrome 7	◯	◯	◯
LSM11 Aicardi-Goutieres syndrome 8	◯	◯	◯

© 2023 Gold NB et al. *JAMA Network Open.*

RNU7-1<br>Aicardi-Goutieres syndrome 9	○	○	○
CHRNA1<br>Congenital myasthenic syndrome 1	○	○	○
CHRNB1<br>Congenital myasthenic syndrome 2	○	○	○
CHRND<br>Congenital myasthenic syndrome 3	○	○	○
CHRNE<br>Congenital myasthenic syndrome 4	○	○	○
COLQ<br>Congenital myasthenic syndrome 5	○	○	○
CHAT<br>Congenital myasthenic syndrome 6	○	○	○
SYT2<br>Congenital myasthenic syndrome 7	○	○	○
AGRN<br>Congenital myasthenic syndrome 8	○	○	○
MUSK<br>Congenital myasthenic syndrome 9	○	○	○
DOK7<br>Congenital myasthenic syndrome 10	○	○	○
RAPSN<br>Congenital myasthenic syndrome 11	○	○	○
GFPT1<br>Congenital myasthenic syndrome 12	○	○	○
DPAGT1<br>Congenital myasthenic syndrome 13	○	○	○
ALG2<br>Congenital myasthenic syndrome 14	○	○	○
ALG14<br>Congenital myasthenic syndrome 15	○	○	○
SCN4A<br>Congenital myasthenic syndrome 16	○	○	○
LRP4<br>Congenital myasthenic syndrome 17	○	○	○
SNAP25<br>Congenital myasthenic syndrome 18	○	○	○
COL13A1<br>Congenital myasthenic syndrome 19	○	○	○

© 2023 Gold NB et al. *JAMA Network Open.*

SLC5A7
Congenital myasthenic syndrome 20

SLC18A3
Congenital myasthenic syndrome 21

PREPL
Congenital myasthenic syndrome 22

SLC25A1
Congenital myasthenic syndrome 23

MYO9A
Congenital myasthenic syndrome 24

ALDH7A1
Pyridoxine-dependent epilepsy

PLPBP
Vitamin B6-dependent epilepsy

SCARB2
Progressive myoclonic epilepsy 4

SCN3A
Familial focal epilepsy with variable foci 4

KCNA1
Episodic ataxia/myokymia syndrome

CACNA1A
Episodic ataxia, type 2

SLC1A3
Episodic ataxia, type 6

ATM
Ataxia-telangiectasia

TTPA
Ataxia with vitamin E deficiency

SCN1A
Early infantile epileptic encephalopathy 6

KCNQ2
Early infantile epileptic encephalopathy 7

SCN2A
Early infantile epileptic encephalopathy 11

SCN8A
Early infantile epileptic encephalopathy 13

KCNT1
Early infantile epileptic encephalopathy 14

© 2023 Gold NB et al. *JAMA Network Open.*

SLC13A5
Early infantile epileptic encephalopathy 25

CAD
Early infantile epileptic encephalopathy 50

GLRA1
Hyperekplexia 1

GLRB
Hyperekplexia 2

SLC6A5
Hyperekplexia 3

GRIN1
Ionotropic glutamate receptor NMDA type subunit 1 dysregulation

GRIN2A
Ionotropic glutamate receptor NMDA type subunit 2A dysregulation

GRIN2B
Ionotropic glutamate receptor NMDA type subunit 2B dysregulation

GRIN2D
Ionotropic glutamate receptor NMDA type subunit 2D superactivity

SLC25A12
Mitochondrial aspartate-glutamate carrier isoform 1 deficiency (aralar deficiency)

SLC18A2
Infantile parkinsonism-dystonia 2

SLC52A3
Brown-Vialetto-Van Laere syndrome 1

SLC52A2
Brown-Vialetto-Van Laere syndrome 2

SPR
Dopa-responsive dystonia due to sepiapterin reductase deficiency

TH
Dopa-responsive dystonia due to tyrosine hydroxylase deficiency

TMLHE
Epsilon-N-trimethyllysine hydroxylase deficiency

SPTLC1
Hereditary sensory neuropathy type IA

© 2023 Gold NB et al. *JAMA Network Open.*

SPTLC2<br>Hereditary sensory neuropathy type IC	○	○	○
FLAD1<br>Lipid storage myopathy due to %avin adenine dinucleotide synthetase deficiency	○	○	○
GNE<br>GNE myopathy	○	○	○
TSC1<br>Tuberous sclerosis 1	○	○	○
TSC2<br>Tuberous sclerosis 2	○	○	○
ARSA<br>Metachromatic leukodystrophy	○	○	○
CACNA1S<br>Hypokalemic periodic paralysis type 1	○	○	○
CHD7<br>CHARGE syndrome	○	○	○
CLCN1<br>Myotonia congenita	○	○	○
CLCN7<br>Osteopetrosis type 4	○	○	○
DMD<br>Duchenne muscular dystrophy and other dystrophinopathies	○	○	○
FARSB<br>Autosomal recessive aminoacyl transfer RNA (tRNA) synthetase (ARS) deficiencies	○	○	○
FOLR1<br>Cerebral folate transport deficiency	○	○	○
NF1<br>Neurofibromatosis type 1	○	○	○
PDGFRB<br>PDGFRB activating spectrum disorder	○	○	○
PRPS1<br>Arts syndrome	○	○	○
PRRT2<br>Episodic kinesigenic dyskinesia 1	○	○	○
SLC5A6<br>Infantile-onset, biotin-responsive neurodegeneration	○	○	○
SARS1<br>SARS1 associated neurodevelopmental disorder with microcephaly, ataxia, and seizures	○	○	○

© 2023 Gold NB et al. *JAMA Network Open.*

Are there any genes we didn't include here that you think should be screened for in healthy newborns?

Other Comments?

Oncology (18 genes)

Would you recommend screening for the following genes in newborns?

(Please scroll to the bottom of this page to leave any comments, questions or concerns)

	Yes	No	Unsure
MSH2 Hereditary nonpolyposis colorectal cancer 1	○	○	○
MLH1 Hereditary nonpolyposis colorectal cancer 2	○	○	○
PMS2 Hereditary nonpolyposis colorectal cancer 4	○	○	○
MSH6 Hereditary nonpolyposis colorectal cancer 5	○	○	○
EPCAM Hereditary nonpolyposis colorectal cancer 8	○	○	○
APC Familial adenomatous polyposis 1/Hepatoblastoma	○	○	○
MUTYH Familial adenomatous polyposis 2	○	○	○
BMPR1A BMPR1A associated juvenile polyposis syndrome	○	○	○
ALK Neuroblastoma	○	○	○
PHOX2B Neuroblastoma	○	○	○
DICER1 Pleuropulmonary blastoma	○	○	○
PTCH1 Medulloblastoma	○	○	○
SUFU Medulloblastoma	○	○	○
RET Multiple endocrine neoplasia II/Medullary thyroid carcinoma	○	○	○

© 2023 Gold NB et al. *JAMA Network Open.*

	Yes	No	Unsure
TP53 Adrenocortical carcinoma	○	○	○
RB1 Retinoblastoma (hereditary)	○	○	○
SMARCB1 Rhabdoid tumors	○	○	○
WT1 Wilms tumor	○	○	○

Are there any genes we didn't include here that you think should be screened for in healthy newborns?

Other Comments?

Ophthalmology (4 genes)

Would you recommend screening for the following genes in newborns?

(Please scroll to the bottom of this page to leave any comments, questions or concerns)

	Yes	No	Unsure
PLG Plasminogen deficiency, type I	○	○	○
RPE65 RPE65 associated Leber congenital amaurosis, early-onset severe retinal dystrophy	○	○	○
SLC6A6 Taurine transporter deficiency	○	○	○
VAMP1 Congenital myasthenic syndrome 25	○	○	○

Are there any genes we didn't include here that you think should be screened for in healthy newborns?

Other Comments?

Pulmonology (2 genes)

Would you recommend screening for the following genes in newborns?

(Please scroll to the bottom of this page to leave any comments, questions or concerns)

	Yes	No	Unsure
SERPINA1 Alpha-1-antitrypsin deficiency	○	○	○
SFTPC			

© 2023 Gold NB et al. *JAMA Network Open.*

Pulmonary surfactant metabolism dysfunction 2

Are there any genes that you would add to a newborn sequencing panel for pulmonology that we didn't include here?

Other Comments?

Part 2: Exploratory Questions

Please indicate your level of agreement with the following statements.

	Disagree	Somewhat disagree	Neither disagree nor agree	Somewhat agree	Agree
Genomic sequencing for treatable genetic conditions that are not currently on the Recommended Uniform Screening Panel should be made available for all newborns.	○	○	○	○	○
Some genes for actionable adult-onset conditions should be sequenced in newborns in order to facilitate cascade testing in parents, who might be a#ected.	○	○	○	○	○
Genomic sequencing of newborns should include genes that are associated with conditions that are treatable, even if these conditions have very low penetrance.	○	○	○	○	○
Genomic sequencing for treatable genetic conditions should only be o#ered for disorders that can be confirmed through non-molecular (e.g. biochemical or imaging) studies.	○	○	○	○	○
Genomic sequencing in newborns should include genes associated with conditions that are not treatable, but have established guidelines for management or surveillance.	○	○	○	○	○
Genomic sequencing in newborns should include childhood onset conditions like developmental delay for which there are no established targeted therapies or expert management guidelines for surveillance.	○	○	○	○	○

© 2023 Gold NB et al. *JAMA Network Open.*

Comments?

Part 3: Demographics

Lastly, we would like to ask some basic questions about you.

What is your age?

What gender do you identify as?
- Female
- Male
- Non-binary
- Other

Are you of Hispanic, Latino, or Spanish origin?
- Yes
- No

What is your race? *(Please check all that apply.)*
- American Indian or Alaska Native
- Asian
- Black or African American
- Native Hawaiian or Pacific Islander
- White
- Other

In which state do you currently reside?

How many years have you been in practice?

Are you currently involved in newborn screening?
- Yes
- No

What type of practice setting do you work in? *(Please check all that apply.)*
- Academic hospital
- Community hospital
- Clinical laboratory
- Newborn screening laboratory
- Commercial laboratory
- Other

What type of patients do you see? *(Please check all that apply.)*
- General Genetics
- Metabolism
- Cardiac Genetics
- Cancer Genetics
- Prenatal Genetics
- Neurogenetics
- Specialized clinic for a single genetic condition
- Other type of clinic

© 2023 Gold NB et al. *JAMA Network Open.*

eFigure. Methods and results of survey

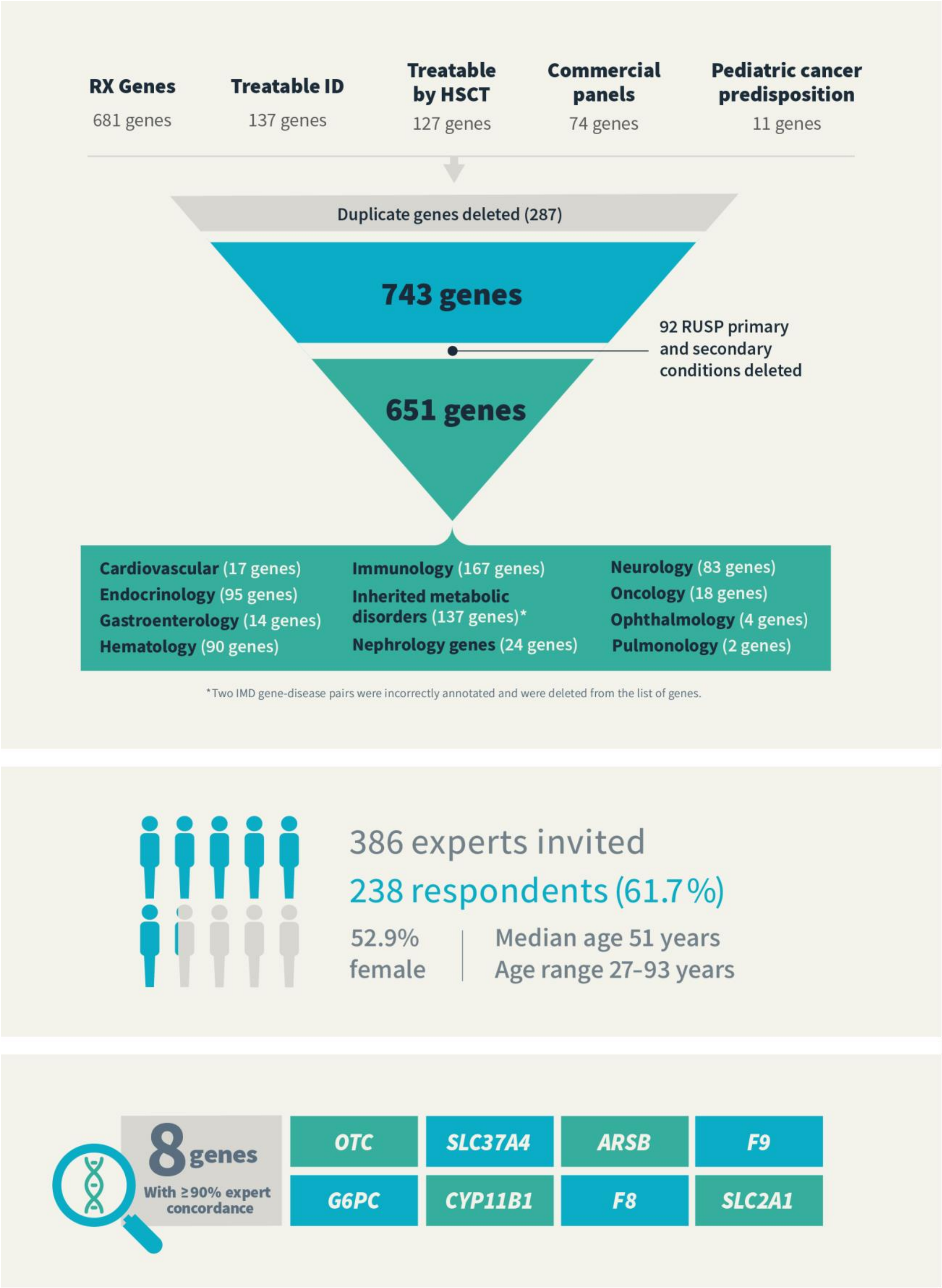

© 2023 Gold NB et al. *JAMA Network Open.*

eTable 2. All Genes Included in Survey, in Order of Concordance

Clinical Category	Gene-disease pairs	Yes (%)	No (%)	Unsure (%)	n	Yes (n)	No (n)	Unsure (n)
Metabolism	*OTC*<br>Ornithine transcarbamylase deficiency	98.4%	1.6%	0.0%	62	61	1	0
Metabolism	*G6PC*<br>Glycogen storage disease Ia	93.4%	4.9%	1.6%	61	57	3	1
Metabolism	*SLC37A4*<br>Glycogen storage disease Ib	93.3%	6.7%	0.0%	60	56	4	0
Endocrinology	*CYP11B1*<br>Congenital adrenal hyperplasia due to 11-beta-hydroxylase deficiency	92.1%	5.3%	2.6%	38	35	2	1
Metabolism	*ARSB*<br>Mucopolysaccharidosis type VI	91.5%	5.1%	3.4%	59	54	3	2
Hematology	*F8*<br>Hemophilia A	90.2%	9.8%	0.0%	41	37	4	0
Hematology	*F9*<br>Hemophilia B	90.2%	9.8%	0.0%	41	37	4	0
Metabolism	*SLC2A1*<br>GLUT1 deficiency syndrome 1	90.2%	4.9%	4.9%	61	55	3	3
Endocrinology	*CYP17A1*<br>17-alpha-hydroxylase/17,20-lyase deficiency	89.5%	5.3%	5.3%	38	34	2	2
Oncology	*RB1*<br>Retinoblastoma (hereditary)	89.3%	8.9%	1.8%	56	50	5	1
Metabolism	*IDS*<br>Mucopolysaccharidosis II	88.7%	8.1%	3.2%	62	55	5	2
Metabolism	*GUSB*<br>Mucopolysaccharidosis type VII	88.5%	6.6%	4.9%	61	54	4	3
Neurology	*DMD*<br>Duchenne muscular dystrophy and other	88.0%	4.0%	8.0%	50	44	2	4
Metabolism	*GLUD1*<br>Hyperinsulinism-hyperammonemia syndrome	87.1%	6.5%	6.5%	62	54	4	4

© 2023 Gold NB et al. *JAMA Network Open.*

Clinical Category	Gene-disease pairs	Yes (%)	No (%)	Unsure (%)	n	Yes (n)	No (n)	Unsure (n)
Endocrinology	*CYP11A1*<br>Adrenal insufficiency, congenital, with 46XY sex reversal, partial or complete	86.8%	2.6%	10.5%	38	33	1	4
Metabolism	*GALNS*<br>Mucopolysaccharidosis IVA	86.7%	10.0%	3.3%	60	52	6	2
Metabolism	*CPS1*<br>Carbamoyl phosphate synthetase I	86.4%	8.5%	5.1%	59	51	5	3
Neurology	*PLPBP*<br>Vitamin B6-dependent epilepsy	86.0%	6.0%	8.0%	50	43	3	4
Neurology	*ALDH7A1*<br>Pyridoxine-dependent epilepsy	85.7%	8.2%	6.1%	49	42	4	3
Gastroenterology	*SLC26A3*<br>Congenital secretory chloride diarrhea	85.3%	8.8%	5.9%	34	29	3	2
Metabolism	*SLC25A15*<br>Hyperornithinemia-hyperammonemia-homocitrullinuria syndrome	85.2%	6.6%	8.2%	61	52	4	5
Metabolism	*SMPD1*<br>Niemann-Pick disease, type A and type B	85.0%	10.0%	5.0%	60	51	6	3
Metabolism	*GATM*<br>Cerebral creatine deficiency syndrome 3	85.0%	10.0%	5.0%	60	51	6	3
Metabolism	*SLC7A7*<br>Lysinuric protein intolerance	85.0%	8.3%	6.7%	60	51	5	4
Metabolism	*NAGS*<br>N-acetylglutamate synthase deficiency	85.0%	8.3%	6.7%	60	51	5	4
Metabolism	*AGL*<br>Glycogen storage disease III	84.7%	10.2%	5.1%	59	50	6	3
Endocrinology	*HSD3B2*<br>Adrenal hyperplasia, congenital, due to 3-beta-hydroxysteroid dehydrogenase 2 deficiency	84.2%	7.9%	7.9%	38	32	3	3
Metabolism	*ATP7A*<br>Menkes disease	84.1%	6.3%	9.5%	63	53	4	6

© 2023 Gold NB et al. *JAMA Network Open.*

Clinical Category	Gene-disease pairs	Yes (%)	No (%)	Unsure (%)	n	Yes (n)	No (n)	Unsure (n)
Metabolism	*ALDOB*<br>Hereditary fructose intolerance	83.9%	9.7%	6.5%	62	52	6	4
Metabolism	*SLC6A8*<br>Creatine transporter deficiency	83.6%	11.5%	4.9%	61	51	7	3
Metabolism	*GAMT*<br>Cerebral creatine deficiency syndrome 2	83.6%	11.5%	4.9%	61	51	7	3
Metabolism	*GBA*<br>Gaucher disease, type I	83.3%	8.3%	8.3%	60	50	5	5
Metabolism	*GLA*<br>Fabry disease	83.3%	8.3%	8.3%	60	50	5	5
Metabolism	*PYGL*<br>Glycogen storage disease VI	82.5%	10.5%	7.0%	57	47	6	4
Endocrinology	*ABCC8*<br>Familial hyperinsulinemic hypoglycemia-1; ABCC8 associated permanent neonatal diabetes mellitus	81.0%	9.5%	9.5%	42	34	4	4
Immunology	*BTK*<br>X-linked agammaglobulinemia	80.6%	9.7%	9.7%	31	25	3	3
Metabolism	*ATP7B*<br>Wilson disease	80.6%	11.3%	8.1%	62	50	7	5
Oncology	*RET*<br>Multiple endocrine neoplasia II/Medullary thyroid carcinoma	80.4%	8.9%	10.7%	56	45	5	6
Gastroenterology	*SLC9A3*<br>Congenital secretory sodium diarrhea	80.0%	14.3%	5.7%	35	28	5	2
Hematology	*G6PD*<br>Hemolytic anemia due to G6PD deficiency	80.0%	12.5%	7.5%	40	32	5	3
Metabolism	*LIPA*<br>Lysosomal acid lipase deficiency	80.0%	6.7%	13.3%	60	48	4	8
Nephrology	*CTNS*<br>Cystinosis	80.0%	13.3%	6.7%	30	24	4	2

© 2023 Gold NB et al. *JAMA Network Open.*

Clinical Category	Gene-disease pairs	Yes (%)	No (%)	Unsure (%)	n	Yes (n)	No (n)	Unsure (n)
Metabolism	*FBP1*<br>Fructose-1,6-bisphosphatase deficiency	79.7%	13.6%	6.8%	59	47	8	4
Gastroenterology	*DGAT1*<br>Diarrhea 7, protein-losing enteropathy type	79.4%	8.8%	11.8%	34	27	3	4
Endocrinology	*POMC*<br>Obesity, adrenal insufficiency, and red hair due to POMC deficiency	78.9%	13.2%	7.9%	38	30	5	3
Endocrinology	*MC2R*<br>Glucocorticoid deficiency due to ACTH unresponsiveness	78.9%	10.5%	10.5%	38	30	4	4
Metabolism	*BCKDK*<br>Branched-chain ketoacid dehydrogenase kinase deficiency	78.9%	12.3%	8.8%	57	45	7	5
Metabolism	*PHKA2*<br>Glycogen storage disease, type IXa	78.3%	13.3%	8.3%	60	47	8	5
Metabolism	*MPI*<br>Congenital disorder of glycosylation, type Ib	78.0%	11.9%	10.2%	59	46	7	6
Cardiovascular	*TAZ*<br>Barth Syndrome	77.8%	12.7%	9.5%	63	49	8	6
Metabolism	*PHKB*<br>Glycogen storage disease, type IXb	76.3%	13.6%	10.2%	59	45	8	6
Metabolism	*PHKA1*<br>Glycogen storage disease, type IXd	76.3%	13.6%	10.2%	59	45	8	6
Endocrinology	*KCNJ11*<br>Familial hyperinsulinemic hypoglycemia-2; KCNJ11 associated permanent neonatal diabetes mellitus	76.2%	9.5%	14.3%	42	32	4	6
Oncology	*WT1*<br>Wilms tumor	75.9%	18.5%	5.6%	54	41	10	3

© 2023 Gold NB et al. *JAMA Network Open.*

Clinical Category	Gene-disease pairs	Yes (%)	No (%)	Unsure (%)	n	Yes (n)	No (n)	Unsure (n)
Metabolism	*PHKG2*<br>Glycogen storage disease, type IXc	75.9%	13.8%	10.3%	58	44	8	6
Metabolism	*MOCS1*<br>Molybdenum cofactor deficiency A	74.6%	13.6%	11.9%	59	44	8	7
Immunology	*CD40LG*<br>X-linked immunodeficiency with hyper-IgM type 1	74.2%	9.7%	16.1%	31	23	3	5
Immunology	*CYBA*<br>CYBA associated chronic granulomatous disease	74.2%	12.9%	12.9%	31	23	4	4
Immunology	*CYBB*<br>X-linked chronic granulomatous disease	74.2%	12.9%	12.9%	31	23	4	4
Immunology	*CYBC1*<br>CYBC1 associated chronic granulomatous disease	74.2%	12.9%	12.9%	31	23	4	4
Neurology	*SLC5A6*<br>Infantile-onset, biotin-responsive neurodegeneration	74.0%	6.0%	20.0%	50	37	3	10
Endocrinology	*PHEX*<br>X-linked dominant hypophosphatemic rickets	73.7%	13.2%	13.2%	38	28	5	5
Endocrinology	*GH1*<br>Isolated growth hormone deficiency type 1A, type 1B, type 2	73.7%	18.4%	7.9%	38	28	7	3
Endocrinology	*GHRHR*<br>Isolated growth hormone deficiency type 4	73.7%	18.4%	7.9%	38	28	7	3
Metabolism	*OXCT1*<br>Succinyl-CoA:3-ketoacid CoA transferase (SCOT) deficiency	73.7%	12.3%	14.0%	57	42	7	8
Endocrinology	*STAR*<br>Lipoid adrenal hyperplasia	73.0%	10.8%	16.2%	37	27	4	6

© 2023 Gold NB et al. *JAMA Network Open.*

Clinical Category	Gene-disease pairs	Yes (%)	No (%)	Unsure (%)	n	Yes (n)	No (n)	Unsure (n)
Hematology	*F13A1*<br>Factor XIIIA deficiency	73.0%	18.9%	8.1%	37	27	7	3
Hematology	*F13B*<br>Factor XIIIB deficiency	73.0%	18.9%	8.1%	37	27	7	3
Metabolism	*PDHA1*<br>Pyruvate dehydrogenase deficiency	72.9%	15.3%	11.9%	59	43	9	7
Metabolism	*PDHB*<br>Pyruvate dehydrogenase deficiency	72.9%	16.9%	10.2%	59	43	10	6
Metabolism	*TCN2*<br>Transcobalamin II deficiency	72.1%	8.2%	19.7%	61	44	5	12
Immunology	*TTC7A*<br>Gastrointestinal defects and immunodeficiency syndrome	71.9%	12.5%	15.6%	32	23	4	5
Metabolism	*OAT*<br>Ornithine aminotransferase deficiency	71.7%	11.7%	16.7%	60	43	7	10
Metabolism	*DDC*<br>Aromatic amino acid decarboxylase	71.7%	11.7%	16.7%	60	43	7	10
Endocrinology	*CYP2R1*<br>Vitamin D-dependent rickets, type IB	71.1%	18.4%	10.5%	38	27	7	4
Endocrinology	*SLC34A3*<br>Hypophosphatemic rickets with hypercalciuria	71.1%	18.4%	10.5%	38	27	7	4
Endocrinology	*ALPL*<br>Hypophosphatasia	71.1%	13.2%	15.8%	38	27	5	6
Immunology	*ZAP70*<br>Immunodeficiency 48	71.0%	6.5%	22.6%	31	22	2	7
Immunology	*DCLRE1C*<br>Omenn syndrome/Severe combined immunodeficiency, Athabascan type	71.0%	6.5%	22.6%	31	22	2	7

© 2023 Gold NB et al. *JAMA Network Open.*

Clinical Category	Gene-disease pairs	Yes (%)	No (%)	Unsure (%)	n	Yes (n)	No (n)	Unsure (n)
Immunology	*RAG2*<br>RAG2 associated T cell-negative, B cell-negative, severe combined immunodeficiency	71.0%	6.5%	22.6%	31	22	2	7
Immunology	*NCF1*<br>NCF1 associated chronic granulomatous disease	71.0%	12.9%	16.1%	31	22	4	5
Immunology	*NCF4*<br>NCF4 associated chronic granulomatous disease	71.0%	12.9%	16.1%	31	22	4	5
Immunology	*WIPF1*<br>Wiskott-Aldrich syndrome 2	71.0%	16.1%	12.9%	31	22	5	4
Neurology	*FOLR1*<br>Cerebral folate transport deficiency	70.8%	6.3%	22.9%	48	34	3	11
Hematology	*SBDS*<br>Shwachman-Diamond syndrome	70.7%	14.6%	14.6%	41	29	6	6
Immunology	*MAGT1*<br>X-linked Immunodeficiency with magnesium defect, Epstein-Barr virus infection and neoplasia	70.0%	10.0%	20.0%	30	21	3	6
Immunology	*NCF2*<br>NCF2 associated chronic granulomatous disease	70.0%	13.3%	16.7%	30	21	4	5
Metabolism	*NAGLU*<br>Mucopolysaccharidosis type IIIB	70.0%	20.0%	10.0%	60	42	12	6
Nephrology	*COL4A5*<br>X-linked Alport syndrome 1	70.0%	20.0%	10.0%	30	21	6	3
Neurology	*SPR*<br>Dopa-responsive dystonia due to sepiapterin reductase deficiency	70.0%	10.0%	20.0%	50	35	5	10

© 2023 Gold NB et al. *JAMA Network Open.*

Clinical Category	Gene-disease pairs	Yes (%)	No (%)	Unsure (%)	n	Yes (n)	No (n)	Unsure (n)
Neurology	*TH*<br>Dopa-responsive dystonia due to tyrosine hydroxylase deficiency	70.0%	12.0%	18.0%	50	35	6	9
Cardiovascular	*LDLR*<br>Familial hypercholesterolemia 1	69.8%	25.4%	4.8%	63	44	16	3
Metabolism	*PDHX*<br>Pyruvate dehydrogenase deficiency	69.5%	16.9%	13.6%	59	41	10	8
Endocrinology	*GHR*<br>Growth hormone receptor deficiency	69.2%	20.5%	10.3%	39	27	8	4
Hematology	*LYST*<br>Chediak-Higashi Syndrome	69.2%	15.4%	15.4%	39	27	6	6
Hematology	*EFL1*<br>Shwachman-Diamond syndrome 2	69.2%	15.4%	15.4%	39	27	6	6
Hematology	*RPS19*<br>Diamond-Blackfan anemia 1	69.2%	17.9%	12.8%	39	27	7	5
Hematology	*RPL5*<br>Diamond-Blackfan anemia 6	69.2%	17.9%	12.8%	39	27	7	5
Hematology	*RPL11*<br>Diamond-Blackfan anemia 7	69.2%	17.9%	12.8%	39	27	7	5
Oncology	*TP53*<br>Adrenocortical carcinoma	69.1%	23.6%	7.3%	55	38	13	4
Endocrinology	*SLC19A2*<br>Thiamine-responsive megaloblastic anemia syndrome with diabetes mellitus and sensorineural deafness	69.0%	16.7%	14.3%	42	29	7	6
Metabolism	*NPC1*<br>Niemann-Pick disease, type C, NPC1	69.0%	22.4%	8.6%	58	40	13	5
Metabolism	*NPC2*<br>Niemann-Pick disease, type C, NPC2	69.0%	22.4%	8.6%	58	40	13	5
Nephrology	*SLC12A1*<br>Bartter syndrome, type 1	69.0%	20.7%	10.3%	29	20	6	3

© 2023 Gold NB et al. *JAMA Network Open.*

Clinical Category	Gene-disease pairs	Yes (%)	No (%)	Unsure (%)	n	Yes (n)	No (n)	Unsure (n)
Nephrology	*KCNJ1*<br>Bartter syndrome, type 2	69.0%	20.7%	10.3%	29	20	6	3
Nephrology	*MAGED2*<br>Bartter syndrome, type 5	69.0%	20.7%	10.3%	29	20	6	3
Cardiovascular	*APOB*<br>Hypobetalipoproteinemia/Familial hypercholesterolemia 2	68.8%	26.6%	4.7%	64	44	17	3
Endocrinology	*CYP27B1*<br>Vitamin D-dependent rickets, type IA	68.4%	21.1%	10.5%	38	26	8	4
Endocrinology	*VDR*<br>Vitamin D-dependent rickets, type 2A	68.4%	21.1%	10.5%	38	26	8	4
Hematology	*RPS24*<br>Diamond-Blackfan anemia 3	68.4%	18.4%	13.2%	38	26	7	5
Hematology	*RPS17*<br>Diamond-Blackfan anemia 4	68.4%	18.4%	13.2%	38	26	7	5
Hematology	*RPL35A*<br>Diamond-Blackfan anemia 5	68.4%	18.4%	13.2%	38	26	7	5
Hematology	*ADAMTS13*<br>Familial thrombotic thrombocytopenic purpura	68.4%	10.5%	21.1%	38	26	4	8
Metabolism	*SGSH*<br>Mucopolysaccharidosis type IIIA	68.3%	20.0%	11.7%	60	41	12	7
Metabolism	*COQ2*<br>Primary coenzyme Q10 deficiency 1	68.3%	10.0%	21.7%	60	41	6	13
Nephrology	*CLCNKB*<br>Bartter syndrome, type 3	67.9%	21.4%	10.7%	28	19	6	3
Nephrology	*BSND*<br>Bartter syndrome, type 4a	67.9%	21.4%	10.7%	28	19	6	3
Metabolism	*DLAT*<br>Pyruvate dehydrogenase deficiency	67.8%	20.3%	11.9%	59	40	12	7

© 2023 Gold NB et al. *JAMA Network Open.*

Clinical Category	Gene-disease pairs	Yes (%)	No (%)	Unsure (%)	n	Yes (n)	No (n)	Unsure (n)
Metabolism	*PNPO*<br>Pyridoxamine 5-prime-phosphate oxidase deficiency	67.8%	8.5%	23.7%	59	40	5	14
Cardiovascular	*LPL*<br>Lipoprotein lipase deficiency	67.7%	21.0%	11.3%	62	42	13	7
Immunology	*LRBA*<br>Common variable immune deficiency 8	67.7%	16.1%	16.1%	31	21	5	5
Immunology	*STAT3*<br>Hyper-IgE recurrent infection syndrome	67.7%	16.1%	16.1%	31	21	5	5
Immunology	*WAS*<br>WAS associated disorder	67.7%	9.7%	22.6%	31	21	3	7
Ophthalmology	*RPE65*<br>RPE65 associated Leber congenital amaurosis, early-onset severe retinal dystrophy	67.6%	8.8%	23.5%	34	23	3	8
Hematology	*VPS45*<br>Severe congenital neutropenia 5	67.6%	13.5%	18.9%	37	25	5	7
Endocrinology	*AIRE*<br>Autoimmune polyendocrinopathy syndrome, type I, with or without reversible metaphyseal dysplasia	67.5%	20.0%	12.5%	40	27	8	5
Hematology	*FANCA*<br>Fanconi anemia, complementation group A	67.5%	15.0%	17.5%	40	27	6	7
Metabolism	*COQ6*<br>Primary coenzyme Q10 deficiency 6	67.2%	10.3%	22.4%	58	39	6	13
Metabolism	*SLC25A19*<br>Thiamine metabolism dysfunction syndrome 4	67.2%	10.3%	22.4%	58	39	6	13
Metabolism	*TPK1*<br>Thiamine metabolism dysfunction syndrome 5	67.2%	10.3%	22.4%	58	39	6	13

© 2023 Gold NB et al. *JAMA Network Open.*

Clinical Category	Gene-disease pairs	Yes (%)	No (%)	Unsure (%)	n	Yes (n)	No (n)	Unsure (n)
Endocrinology	*GCK*<br>Familial hyperinsulinemic hypoglycemia 3	66.7%	14.3%	19.0%	42	28	6	8
Hematology	*SRP54*<br>SRP54 associated Shwachman-Diamond syndrome	66.7%	15.4%	17.9%	39	26	6	7
Hematology	*FANCB*<br>Fanconi anemia, complementation group B	66.7%	15.4%	17.9%	39	26	6	7
Hematology	*BRCA2*<br>Fanconi anemia, complementation group D1	66.7%	20.5%	12.8%	39	26	8	5
Hematology	*RPL31*<br>RPL31 associated Diamond-Blackfan anemia	66.7%	16.7%	16.7%	36	24	6	6
Hematology	*HBA1*<br>Alpha-thalassemia	66.7%	10.3%	23.1%	39	26	4	9
Metabolism	*PDSS2*<br>Primary coenzyme Q10 deficiency 3	66.1%	11.9%	22.0%	59	39	7	13
Metabolism	*COQ8A*<br>Primary coenzyme Q10 deficiency 4	66.1%	11.9%	22.0%	59	39	7	13
Metabolism	*COQ4*<br>Primary coenzyme Q10 deficiency 7	66.1%	11.9%	22.0%	59	39	7	13
Metabolism	*HMGCS2*<br>3-hydroxy-3-methylglutaryl-CoA synthase deficiency	66.1%	13.6%	20.3%	59	39	8	12
Oncology	*APC*<br>Familial adenomatous polyposis 1/Hepatoblastoma	66.1%	23.2%	10.7%	56	37	13	6
Endocrinology	*NR5A1*<br>NR5A1 associated adrenocortical insufficiency	65.8%	15.8%	18.4%	38	25	6	7

© 2023 Gold NB et al. *JAMA Network Open.*

Clinical Category	Gene-disease pairs	Yes (%)	No (%)	Unsure (%)	n	Yes (n)	No (n)	Unsure (n)
Endocrinology	*MRAP*<br>Glucocorticoid deficiency 2	65.8%	13.2%	21.1%	38	25	5	8
Endocrinology	*NNT*<br>Glucocorticoid deficiency 4, with or without mineralocorticoid deficiency	65.8%	13.2%	21.1%	38	25	5	8
Endocrinology	*TBX19*<br>Adrenocorticotropic hormone deficiency	65.8%	10.5%	23.7%	38	25	4	9
Endocrinology	*IGF1*<br>Insulin-like growth factor I deficiency	65.8%	18.4%	15.8%	38	25	7	6
Endocrinology	*POU1F1*<br>Combined pituitary hormone deficiency 1	65.8%	21.1%	13.2%	38	25	8	5
Endocrinology	*PROP1*<br>Combined pituitary hormone deficiency 2	65.8%	18.4%	15.8%	38	25	7	6
Endocrinology	*LHX3*<br>Combined pituitary hormone deficiency 3	65.8%	18.4%	15.8%	38	25	7	6
Endocrinology	*LHX4*<br>Combined pituitary hormone deficiency 4	65.8%	18.4%	15.8%	38	25	7	6
Endocrinology	*HESX1*<br>Combined pituitary hormone deficiency 5	65.8%	18.4%	15.8%	38	25	7	6
Endocrinology	*WFS1*<br>Wolfram syndrome 1	65.8%	21.1%	13.2%	38	25	8	5
Hematology	*RPS7*<br>Diamond-Blackfan anemia 8	65.8%	18.4%	15.8%	38	25	7	6
Hematology	*RPS10*<br>Diamond-Blackfan anemia 9	65.8%	18.4%	15.8%	38	25	7	6
Hematology	*RPS26*<br>Diamond-Blackfan anemia 10	65.8%	18.4%	15.8%	38	25	7	6
Hematology	*RPL26*<br>Diamond-Blackfan anemia 11	65.8%	18.4%	15.8%	38	25	7	6
Hematology	*RPL15*<br>Diamond-Blackfan anemia 12	65.8%	18.4%	15.8%	38	25	7	6

© 2023 Gold NB et al. *JAMA Network Open.*

Clinical Category	Gene-disease pairs	Yes (%)	No (%)	Unsure (%)	n	Yes (n)	No (n)	Unsure (n)
Hematology	*RPS29*<br>Diamond-Blackfan anemia 13	65.8%	18.4%	15.8%	38	25	7	6
Hematology	*RPL27*<br>Diamond-Blackfan anemia 16	65.8%	18.4%	15.8%	38	25	7	6
Hematology	*RPS27*<br>Diamond-Blackfan anemia 17	65.8%	18.4%	15.8%	38	25	7	6
Hematology	*RPL18*<br>Diamond-Blackfan anemia 18	65.8%	18.4%	15.8%	38	25	7	6
Hematology	*RPL35*<br>Diamond-Blackfan anemia 19	65.8%	18.4%	15.8%	38	25	7	6
Hematology	*RPS15A*<br>Diamond-Blackfan anemia 20	65.8%	18.4%	15.8%	38	25	7	6
Hematology	*HBA2*<br>Alpha-thalassemia	65.8%	10.5%	23.7%	38	25	4	9
Cardiovascular	*PCSK9*<br>Familial hypercholesterolemia 3	65.6%	29.7%	4.7%	64	42	19	3
Metabolism	*COQ7*<br>Primary coenzyme Q10 deficiency 8	65.5%	12.1%	22.4%	58	38	7	13
Nephrology	*ATP6V0A4*<br>ATP6V0A4 associated distal renal tubular acidosis	65.5%	13.8%	20.7%	29	19	4	6
Nephrology	*ATP6V1B1*<br>ATP6V1B1 associated distal renal tubular acidosis	65.5%	13.8%	20.7%	29	19	4	6
Nephrology	*SLC4A1*<br>SLC4A1 associated distal renal tubular acidosis	65.5%	13.8%	20.7%	29	19	4	6
Nephrology	*COQ8B*<br>Nephrotic syndrome, type 9	65.5%	17.2%	17.2%	29	19	5	5
Nephrology	*GRHPR*<br>Primary hyperoxaluria type III	65.5%	17.2%	17.2%	29	19	5	5

© 2023 Gold NB et al. *JAMA Network Open.*

Clinical Category	Gene-disease pairs	Yes (%)	No (%)	Unsure (%)	n	Yes (n)	No (n)	Unsure (n)
Nephrology	*HOGA1*<br>Primary hyperoxaluria type III	65.5%	17.2%	17.2%	29	19	5	5
Nephrology	*SLC12A3*<br>Gitelman syndrome	65.5%	24.1%	10.3%	29	19	7	3
Neurology	*TTPA*<br>Ataxia with vitamin E deficiency	65.3%	10.2%	24.5%	49	32	5	12
Endocrinology	*AVP*<br>Neurohypophyseal diabetes insipidus	65.0%	20.0%	15.0%	40	26	8	6
Hematology	*FANCC*<br>Fanconi anemia, complementation group C	65.0%	17.5%	17.5%	40	26	7	7
Hematology	*TERC*<br>Dyskeratosis congenita, autosomal dominant 1	65.0%	15.0%	20.0%	40	26	6	8
Hematology	*DKC1*<br>Dyskeratosis congenita, X-linked	65.0%	15.0%	20.0%	40	26	6	8
Hematology	*GATA1*<br>GATA1 associated X-Linked Cytopenia	64.9%	16.2%	18.9%	37	24	6	7
Hematology	*TSR2*<br>Diamond-Blackfan anemia 14 with mandibulofacial dysostosis	64.9%	18.9%	16.2%	37	24	7	6
Hematology	*RPS28*<br>Diamond Blackfan anemia 15 with mandibulofacial dysostosis	64.9%	18.9%	16.2%	37	24	7	6
Cardiovascular	*LDLRAP1*<br>Familial hypercholesterolemia 4	64.5%	27.4%	8.1%	62	40	17	5
Immunology	*AICDA*<br>Immunodeficiency with hyper-IgM, type 2	64.5%	16.1%	19.4%	31	20	5	6
Immunology	*FOXN1*<br>T-cell immunodeficiency with congenital alopecia and nail dystrophy	64.5%	12.9%	22.6%	31	20	4	7

© 2023 Gold NB et al. *JAMA Network Open.*

Clinical Category	Gene-disease pairs	Yes (%)	No (%)	Unsure (%)	n	Yes (n)	No (n)	Unsure (n)
Immunology	*CD79A*<br>Agammaglobulinemia 3	64.5%	9.7%	25.8%	31	20	3	8
Immunology	*BLNK*<br>Agammaglobulinemia 4	64.5%	9.7%	25.8%	31	20	3	8
Immunology	*CD79B*<br>Agammaglobulinemia 6	64.5%	9.7%	25.8%	31	20	3	8
Immunology	*NFKB1*<br>Common variable immune deficiency 12	64.5%	22.6%	12.9%	31	20	7	4
Immunology	*IKZF1*<br>Common variable immune deficiency 13	64.5%	16.1%	19.4%	31	20	5	6
Immunology	*ADA2*<br>Vasculitis, autoinflammation, immunodeficiency, and hematologic defects syndrome	64.5%	12.9%	22.6%	31	20	4	7
Immunology	*AK2*<br>Reticular dysgenesis	64.5%	9.7%	25.8%	31	20	3	8
Immunology	*FOXP3*<br>X-linked immunodysregulation, polyendocrinopathy, and enteropathy	64.5%	12.9%	22.6%	31	20	4	7
Metabolism	*PDSS1*<br>Primary coenzyme Q10 deficiency 2	64.4%	11.9%	23.7%	59	38	7	14
Metabolism	*COQ9*<br>Primary coenzyme Q10 deficiency 5	64.4%	11.9%	23.7%	59	38	7	14
Metabolism	*TK2*<br>Thymidine kinase deficiency	64.4%	15.3%	20.3%	59	38	9	12
Metabolism	*PGM1*<br>Congenital disorder of glycosylation, type It	64.4%	20.3%	15.3%	59	38	12	9
Metabolism	*CYP27A1*<br>Cerebrotendinous xanthomatosis	64.4%	15.3%	20.3%	59	38	9	12

© 2023 Gold NB et al. *JAMA Network Open.*

Clinical Category	Gene-disease pairs	Yes (%)	No (%)	Unsure (%)	n	Yes (n)	No (n)	Unsure (n)
Hematology	*FANCD2*<br>Fanconi anemia, complementation group D2	64.1%	17.9%	17.9%	39	25	7	7
Hematology	*FANCE*<br>Fanconi anemia, complementation group E	64.1%	17.9%	17.9%	39	25	7	7
Hematology	*FANCF*<br>Fanconi anemia, complementation group F	64.1%	17.9%	17.9%	39	25	7	7
Hematology	*FANCG*<br>Fanconi anemia, complementation group G	64.1%	17.9%	17.9%	39	25	7	7
Hematology	*FANCI*<br>Fanconi anemia, complementation group I	64.1%	17.9%	17.9%	39	25	7	7
Hematology	*PALB2*<br>Fanconi anemia, complementation group N	64.1%	20.5%	15.4%	39	25	8	6
Hematology	*TINF2*<br>Dyskeratosis congenita, autosomal dominant 3	64.1%	15.4%	20.5%	39	25	6	8
Hematology	*ELANE*<br>ELANE associated neutropenia 1	64.1%	17.9%	17.9%	39	25	7	7
Metabolism	*HGSNAT*<br>Mucopolysaccharidosis type IIIC (Sanfilippo C)	63.9%	23.0%	13.1%	61	39	14	8
Metabolism	*HEXA*<br>Tay-Sachs disease	63.3%	26.7%	10.0%	60	38	16	6
Hematology	*SAMD9L*<br>Ataxia-pancytopenia syndrome	63.2%	15.8%	21.1%	38	24	6	8
Hematology	*BRIP1*<br>Fanconi anemia, complementation group J	63.2%	21.1%	15.8%	38	24	8	6

© 2023 Gold NB et al. *JAMA Network Open.*

Clinical Category	Gene-disease pairs	Yes (%)	No (%)	Unsure (%)	n	Yes (n)	No (n)	Unsure (n)
Hematology	*FANCL*<br>Fanconi anemia, complementation group L	63.2%	18.4%	18.4%	38	24	7	7
Metabolism	*MLYCD*<br>Malonyl-CoA decarboxylase deficiency	63.2%	14.0%	22.8%	57	36	8	13
Metabolism	*DLD*<br>Dihydrolipoamide dehydrogenase deficiency	63.2%	12.3%	24.6%	57	36	7	14
Endocrinology	*NEUROG3*<br>NEUROG3 associated neonatal diabetes mellitus	62.8%	18.6%	18.6%	43	27	8	8
Endocrinology	*NKX2-2*<br>NKX2-2 associated neonatal diabetes mellitus	62.8%	18.6%	18.6%	43	27	8	8
Endocrinology	*AQP2*<br>Nephrogenic diabetes insipidus	62.5%	22.5%	15.0%	40	25	9	6
Hematology	*BRCA1*<br>Fanconi anemia, complementation group S	62.5%	22.5%	15.0%	40	25	9	6
Endocrinology	*SAMD9*<br>MIRAGE syndrome	62.2%	13.5%	24.3%	37	23	5	9
Endocrinology	*GNAS*<br>GNAS associated Pseudohypoparathyroidism	62.2%	24.3%	13.5%	37	23	9	5
Hematology	*SLC46A1*<br>Hereditary folate malabsorption	62.2%	16.2%	21.6%	37	23	6	8
Hematology	*VKORC1*<br>Combined deficiency of vitamin K-dependent clotting factors 2	62.2%	18.9%	18.9%	37	23	7	7
Hematology	*WDR1*<br>Periodic fever, immunodeficiency, and thrombocytopenia syndrome	62.2%	18.9%	18.9%	37	23	7	7

© 2023 Gold NB et al. *JAMA Network Open.*

Clinical Category	Gene-disease pairs	Yes (%)	No (%)	Unsure (%)	n	Yes (n)	No (n)	Unsure (n)
Immunology	*STAT5B*<br>Growth hormone insensitivity with immunodeficiency	62.1%	17.2%	20.7%	29	18	5	6
Metabolism	*TPP1*<br>Neuronal ceroid lipofuscinosis 2	62.1%	19.0%	19.0%	58	36	11	11
Nephrology	*WDR72*<br>WDR72 associated distal renal tubular acidosis	62.1%	17.2%	20.7%	29	18	5	6
Metabolism	*CA5A*<br>Carbonic anhydrase VA deficiency	61.8%	10.9%	27.3%	55	34	6	15
Metabolism	*MAN2B1*<br>Alpha-mannosidosis	61.7%	21.7%	16.7%	60	37	13	10
Hematology	*RAD51C*<br>Fanconi amenia, complementation group O	61.5%	20.5%	17.9%	39	24	8	7
Metabolism	*AGXT*<br>Primary hyperoxaluria type I	61.4%	17.5%	21.1%	57	35	10	12
Metabolism	*DHFR*<br>Dihydrofolate reductase deficiency	61.4%	12.3%	26.3%	57	35	7	15
Immunology	*NFKBIA*<br>Ectodermal dysplasia and immunodeficiency 2	61.3%	16.1%	22.6%	31	19	5	7
Immunology	*CARD11*<br>B-cell expansion with NKFB and T-cell anergy/Immunodeficiency 11B with atopic dermatitis	61.3%	19.4%	19.4%	31	19	6	6
Immunology	*IGHM*<br>Agammaglobulinemia 1	61.3%	9.7%	29.0%	31	19	3	9
Immunology	*IGLL1*<br>Agammaglobulinemia 2	61.3%	9.7%	29.0%	31	19	3	9
Immunology	*CD19*<br>Common variable immune deficiency 3	61.3%	19.4%	19.4%	31	19	6	6

© 2023 Gold NB et al. *JAMA Network Open.*

Clinical Category	Gene-disease pairs	Yes (%)	No (%)	Unsure (%)	n	Yes (n)	No (n)	Unsure (n)
Immunology	*NFKB2*<br>Common variable immune deficiency 10	61.3%	22.6%	16.1%	31	19	7	5
Immunology	*IL21*<br>Common variable immune deficiency 11	61.3%	25.8%	12.9%	31	19	8	4
Immunology	*CTLA4*<br>Autoimmune lymphoproliferative syndrome, type V	61.3%	12.9%	25.8%	31	19	4	8
Immunology	*XIAP*<br>X-linked lymphoproliferative syndrome 2	61.3%	12.9%	25.8%	31	19	4	8
Immunology	*G6PC3*<br>Severe congenital neutropenia 4	61.3%	16.1%	22.6%	31	19	5	7
Immunology	*CSF3R*<br>Severe congenital neutropenia 7	61.3%	16.1%	22.6%	31	19	5	7
Immunology	*DOCK8*<br>DOCK8 deficiency	61.3%	9.7%	29.0%	31	19	3	9
Immunology	*UNC13D*<br>Familial hemophagocytic lymphohistiocytosis 3	61.3%	12.9%	25.8%	31	19	4	8
Immunology	*STX11*<br>Familial hemophagocytic lymphohistiocytosis 4	61.3%	12.9%	25.8%	31	19	4	8
Immunology	*RMRP*<br>Cartilage-hair hypoplasia	61.3%	16.1%	22.6%	31	19	5	7
Immunology	*LIG4*<br>LIG4 syndrome	61.3%	6.5%	32.3%	31	19	2	10
Immunology	*PNP*<br>Purine nucleoside phosphorylase deficiency	61.3%	12.9%	25.8%	31	19	4	8
Hematology	*GGCX*<br>Combined deficiency of vitamin K-dependent clotting factors 1	61.1%	16.7%	22.2%	36	22	6	8

© 2023 Gold NB et al. *JAMA Network Open.*

Clinical Category	Gene-disease pairs	Yes (%)	No (%)	Unsure (%)	n	Yes (n)	No (n)	Unsure (n)
Metabolism	*SLC5A1*<br>Glucose-galactose malabsorption	61.0%	11.9%	27.1%	59	36	7	16
Nephrology	*SLC4A4*<br>SLC4A4 associated proximal renal tubular acidosis	60.7%	21.4%	17.9%	28	17	6	5
Metabolism	*GNPTA*<br>I-Cell Disease	60.7%	32.8%	6.6%	61	37	20	4
Metabolism	*GALC*<br>Krabbe disease	60.7%	29.5%	9.8%	61	37	18	6
Endocrinology	*HSD11B2*<br>Apparent mineralocorticoid excess	60.5%	15.8%	23.7%	38	23	6	9
Endocrinology	*RNPC3*<br>RNPC3 associated growth hormone deficiency	60.5%	23.7%	15.8%	38	23	9	6
Endocrinology	*LEP*<br>Leptin deficiency	60.5%	23.7%	15.8%	38	23	9	6
Endocrinology	*LEPR*<br>Leptin receptor deficiency	60.5%	23.7%	15.8%	38	23	9	6
Hematology	*SLX4*<br>Fanconi anemia, complementation group P	60.5%	18.4%	21.1%	38	23	7	8
Hematology	*ERCC4*<br>Fanconi anemia, complementation group Q	60.5%	18.4%	21.1%	38	23	7	8
Hematology	*UBE2T*<br>Fanconi anemia, complementation group T	60.5%	18.4%	21.1%	38	23	7	8
Hematology	*MAD2L2*<br>Fanconi anemia, complementation group V	60.5%	18.4%	21.1%	38	23	7	8

© 2023 Gold NB et al. *JAMA Network Open.*

Clinical Category	Gene-disease pairs	Yes (%)	No (%)	Unsure (%)	n	Yes (n)	No (n)	Unsure (n)
Hematology	*RFWD3* Fanconi anemia, complementation group W	60.5%	18.4%	21.1%	38	23	7	8
Hematology	*RTEL1* Dyskeratosis congenita	60.5%	15.8%	23.7%	38	23	6	9
Endocrinology	*MNX1* MNX1 associated neonatal diabetes mellitus	60.5%	16.3%	23.3%	43	26	7	10
Endocrinology	*AVPR2* X-linked nephrogenic diabetes insipidus	60.0%	22.5%	17.5%	40	24	9	7
Immunology	*SP110* Hepatic venoocclusive disease with immunodeficiency	60.0%	13.3%	26.7%	30	18	4	8
Immunology	*ITGB2* Leukocyte adhesion deficiency, type I	60.0%	10.0%	30.0%	30	18	3	9
Immunology	*STXBP2* Familial hemophagocytic lymphohistiocytosis 5	60.0%	13.3%	26.7%	30	18	4	8
Nephrology	*PMM2* Polycystic kidney disease with hyperinsulinemic hypoglycemia	60.0%	20.0%	20.0%	30	18	6	6
Neurology	*ARSA* Metachromatic leukodystrophy	60.0%	22.0%	18.0%	50	30	11	9
Hematology	*SLC19A1* Folate dependent megaloblastic anemia	59.5%	18.9%	21.6%	37	22	7	8
Immunology	*IKBKB* Immunodeficiency 15, 15B	59.4%	12.5%	28.1%	32	19	4	9
Endocrinology	*CYP11B2* Aldosterone synthase deficiency	59.0%	12.8%	28.2%	39	23	5	11
Endocrinology	*CACNA1D* Primary aldosteronism with seizures and neurologic abnormalities	59.0%	15.4%	25.6%	39	23	6	10

© 2023 Gold NB et al. *JAMA Network Open.*

Clinical Category	Gene-disease pairs	Yes (%)	No (%)	Unsure (%)	n	Yes (n)	No (n)	Unsure (n)
Oncology	*ALK*<br>Neuroblastoma	58.8%	23.5%	17.6%	51	30	12	9
Immunology	*STK4*<br>STK4 associated T-cell immunodeficiency, recurrent infections, autoimmunity, and cardiac malformations	58.6%	10.3%	31.0%	29	17	3	9
Nephrology	*FOXI1*<br>FOXI1 associated distal renal tubular acidosis	58.6%	20.7%	20.7%	29	17	6	6
Nephrology	*COL4A4*<br>Alport syndrome 2	58.6%	31.0%	10.3%	29	17	9	3
Nephrology	*COL4A3*<br>Alport syndrome 3	58.6%	31.0%	10.3%	29	17	9	3
Gastroenterology	*HSD3B7*<br>Congenital bile acid synthesis defect type 1	58.3%	13.9%	27.8%	36	21	5	10
Gastroenterology	*AKR1D1*<br>Congenital bile acid synthesis defect type 2	58.3%	13.9%	27.8%	36	21	5	10
Gastroenterology	*TRMU*<br>Transient infantile liver failure	58.3%	11.1%	30.6%	36	21	4	11
Metabolism	*UMPS*<br>Orotic aciduria	58.3%	25.0%	16.7%	60	35	15	10
Cardiovascular	*ENPP1*<br>Generalized arterial calcification of infancy 1	58.1%	17.7%	24.2%	62	36	11	15
Immunology	*GATA2*<br>Immunodeficiency 21	58.1%	12.9%	29.0%	31	18	4	9
Immunology	*PRKDC*<br>Immunodeficiency 26	58.1%	9.7%	32.3%	31	18	3	10
Immunology	*CD40*<br>Immunodeficiency with hyper-IgM, type 3	58.1%	12.9%	29.0%	31	18	4	9

© 2023 Gold NB et al. *JAMA Network Open.*

Clinical Category	Gene-disease pairs	Yes (%)	No (%)	Unsure (%)	n	Yes (n)	No (n)	Unsure (n)
Immunology	*PIK3R1*<br>Agammaglobulinemia 7	58.1%	12.9%	29.0%	31	18	4	9
Immunology	*TCF3*<br>Agammaglobulinemia 8	58.1%	9.7%	32.3%	31	18	3	10
Immunology	*ICOS*<br>Common variable immune deficiency 1	58.1%	29.0%	12.9%	31	18	9	4
Immunology	*IRF2BP2*<br>Common variable immune deficiency 14	58.1%	29.0%	12.9%	31	18	9	4
Immunology	*SH2D1A*<br>X-linked lymphoproliferative syndrome 1	58.1%	12.9%	29.0%	31	18	4	9
Immunology	*GFI1*<br>Severe congenital neutropenia 2	58.1%	16.1%	25.8%	31	18	5	8
Immunology	*HAX1*<br>Severe congenital neutropenia 3	58.1%	12.9%	29.0%	31	18	4	9
Immunology	*JAGN1*<br>Severe congenital neutropenia 6	58.1%	16.1%	25.8%	31	18	5	8
Immunology	*DOCK2*<br>DOCK2 defeciency	58.1%	9.7%	32.3%	31	18	3	10
Immunology	*FERMT3*<br>Leukocyte adhesion deficiency, type III	58.1%	12.9%	29.0%	31	18	4	9
Immunology	*PRF1*<br>Familial hemophagocytic lymphohistiocytosis 2	58.1%	16.1%	25.8%	31	18	5	8
Immunology	*MVK*<br>Hyper-IgD syndrome / mevalonate kinase deficiciency	58.1%	22.6%	19.4%	31	18	7	6
Immunology	*C1NH*<br>Hereditary angioedema	58.1%	22.6%	19.4%	31	18	7	6
Neurology	*ATM*<br>Ataxia-telangiectasia	58.0%	22.0%	20.0%	50	29	11	10
Neurology	*TSC1*<br>Tuberous sclerosis 1	58.0%	28.0%	14.0%	50	29	14	7

© 2023 Gold NB et al. *JAMA Network Open.*

Clinical Category	Gene-disease pairs	Yes (%)	No (%)	Unsure (%)	n	Yes (n)	No (n)	Unsure (n)
Neurology	*TSC2*<br>Tuberous sclerosis 2	58.0%	28.0%	14.0%	50	29	14	7
Endocrinology	*SOX3*<br>X-linked panhypopituitarism	57.9%	18.4%	23.7%	38	22	7	9
Hematology	*DNAJC21*<br>Bone marrow failure syndrome 3	57.9%	18.4%	23.7%	38	22	7	9
Hematology	*MYSM1*<br>Bone marrow failure syndrome 4	57.9%	18.4%	23.7%	38	22	7	9
Hematology	*MPL*<br>Congenital amegakaryocytic thrombocytopenia	57.9%	21.1%	21.1%	38	22	8	8
Metabolism	*COQ5*<br>Coenzyme Q5 methyltransferase deficiency	57.9%	14.0%	28.1%	57	33	8	16
Metabolism	*PDP1*<br>Pyruvate dehydrogenase phosphatase deficiency	57.9%	19.3%	22.8%	57	33	11	13
Oncology	*PHOX2B*<br>Neuroblastoma	57.7%	23.1%	19.2%	52	30	12	10
Metabolism	*ACAD9*<br>Mitochondrial complex I deficiency nuclear type 20	57.6%	28.8%	13.6%	59	34	17	8
Metabolism	*ALDH5A1*<br>Succinic semialdehyde dehydrogenase deficiency	57.6%	20.3%	22.0%	59	34	12	13
Metabolism	*HEXB*<br>Sandhoff disease, infantile, juvenile, and adult forms	57.4%	27.9%	14.8%	61	35	17	9
Metabolism	*DHCR7*<br>7-dehydrocholesterol reductase deficiency	57.4%	23.0%	19.7%	61	35	14	12
Neurology	*CHRNA1*<br>Congenital myasthenic syndrome 1	57.1%	24.5%	18.4%	49	28	12	9

© 2023 Gold NB et al. *JAMA Network Open.*

Clinical Category	Gene-disease pairs	Yes (%)	No (%)	Unsure (%)	n	Yes (n)	No (n)	Unsure (n)
Metabolism	*ACAT1*<br>Mitochondrial acetoacetyl-CoA thiolase deficiency	56.9%	25.9%	17.2%	58	33	15	10
Metabolism	*AMT*<br>Glycine encephalopathy due to aminomethyltransferase (AMT)	56.9%	25.9%	17.2%	58	33	15	10
Metabolism	*SLC39A4*<br>Acrodermatitis enteropathica	56.9%	19.0%	24.1%	58	33	11	14
Metabolism	*SI*<br>Congenital sucrase-isomaltase deficiency	56.9%	13.8%	29.3%	58	33	8	17
Hematology	*NBN*<br>Nijmegen breakage syndrome	56.8%	13.5%	29.7%	37	21	5	11
Endocrinology	*GATA6*<br>Pancreatic agenesis and congenital heart defects	56.4%	20.5%	23.1%	39	22	8	9
Endocrinology	*TCIRG1*<br>Osteopetrosis type 1	56.4%	20.5%	23.1%	39	22	8	9
Endocrinology	*PCSK1*<br>Obesity with impaired prohormone processing	56.4%	25.6%	17.9%	39	22	10	7
Immunology	*IL2RA*<br>Immunodeficiency 41 with lymphoproliferation and autoimmunity	56.3%	6.3%	37.5%	32	18	2	12
Neurology	*DPAGT1*<br>Congenital myasthenic syndrome 13	56.3%	20.8%	22.9%	48	27	10	11
Pulmonology	*SFTPC*<br>Pulmonary surfactant metabolism dysfunction 2	56.3%	15.6%	28.1%	32	18	5	9
Cardiovascular	*ABCC6*<br>Generalized arterial calcification of infancy 2	55.6%	19.0%	25.4%	63	35	12	16

© 2023 Gold NB et al. *JAMA Network Open.*

Clinical Category	Gene-disease pairs	Yes (%)	No (%)	Unsure (%)	n	Yes (n)	No (n)	Unsure (n)
Gastroenterology	*CYP7B1*<br>Congenital bile acid synthesis defect type 3	55.6%	16.7%	27.8%	36	20	6	10
Metabolism	*CP*<br>Aceruloplasminemia	55.4%	14.3%	30.4%	56	31	8	17
Metabolism	*PKLR*<br>Pyruvate kinase deficiency	55.2%	24.1%	20.7%	58	32	14	12
Neurology	*COLQ*<br>Congenital myasthenic syndrome 5	55.1%	22.4%	22.4%	49	27	11	11
Neurology	*CHAT*<br>Congenital myasthenic syndrome 6	55.1%	22.4%	22.4%	49	27	11	11
Neurology	*AGRN*<br>Congenital myasthenic syndrome 8	55.1%	22.4%	22.4%	49	27	11	11
Neurology	*DOK7*<br>Congenital myasthenic syndrome 10	55.1%	22.4%	22.4%	49	27	11	11
Neurology	*ALG2*<br>Congenital myasthenic syndrome 14	55.1%	22.4%	22.4%	49	27	11	11
Neurology	*SCN4A*<br>Congenital myasthenic syndrome 16	55.1%	22.4%	22.4%	49	27	11	11
Neurology	*SLC5A7*<br>Congenital myasthenic syndrome 20	55.1%	22.4%	22.4%	49	27	11	11
Neurology	*PREPL*<br>Congenital myasthenic syndrome 22	55.1%	22.4%	22.4%	49	27	11	11
Neurology	*SLC25A1*<br>Congenital myasthenic syndrome 23	55.1%	22.4%	22.4%	49	27	11	11
Neurology	*MYO9A*<br>Congenital myasthenic syndrome 24	55.1%	22.4%	22.4%	49	27	11	11
Endocrinology	*HNF1A*<br>HNF1A associated hyperinsulinism	55.0%	17.5%	27.5%	40	22	7	11
Endocrinology	*HNF4A*<br>HNF4A associated hyperinsulinism	55.0%	17.5%	27.5%	40	22	7	11

© 2023 Gold NB et al. *JAMA Network Open.*

Clinical Category	Gene-disease pairs	Yes (%)	No (%)	Unsure (%)	n	Yes (n)	No (n)	Unsure (n)
Immunology	*IFNGR1*<br>Immunodeficiency 27B	54.8%	9.7%	35.5%	31	17	3	11
Immunology	*STAT1*<br>Immunodeficiency 31B	54.8%	16.1%	29.0%	31	17	5	9
Immunology	*LIG1*<br>LIG1 associated immunodeficiency	54.8%	9.7%	35.5%	31	17	3	11
Immunology	*CD81*<br>Common variable immune deficiency 6	54.8%	29.0%	16.1%	31	17	9	5
Immunology	*CIITA*<br>Bare lymphocyte syndrome, type II, complementation group A	54.8%	6.5%	38.7%	31	17	2	12
Immunology	*RFX5*<br>Bare lymphocyte syndrome, type II, complementation group C and group E	54.8%	6.5%	38.7%	31	17	2	12
Immunology	*RFXAP*<br>Bare lymphocyte syndrome, type II, complementation group D	54.8%	6.5%	38.7%	31	17	2	12
Immunology	*CARD9*<br>Candidiasis, familial	54.8%	22.6%	22.6%	31	17	7	7
Nephrology	*PKD1*<br>Polycystic kidney disease 1	54.8%	32.3%	12.9%	31	17	10	4
Nephrology	*PKD2*<br>Polycystic kidney disease 2	54.8%	32.3%	12.9%	31	17	10	4
Endocrinology	*HNF1B*<br>Renal cysts and diabetes syndrome	54.8%	26.2%	19.0%	42	23	11	8
Cardiovascular	*LMNA*<br>Hutchinson-Gilford progeria syndrome	54.7%	32.8%	12.5%	64	35	21	8
Oncology	*DICER1*<br>Pleuropulmonary blastoma	54.5%	25.5%	20.0%	55	30	14	11
Oncology	*SMARCB1*<br>Rhabdoid tumors	54.5%	29.1%	16.4%	55	30	16	9

© 2023 Gold NB et al. *JAMA Network Open.*

Clinical Category	Gene-disease pairs	Yes (%)	No (%)	Unsure (%)	n	Yes (n)	No (n)	Unsure (n)
Pulmonology	*SERPINA1*<br>Alpha-1-antitrypsin deficiency	54.5%	24.2%	21.2%	33	18	8	7
Hematology	*HOXA11*<br>Radioulnar synostosis with amegakaryocytic thrombocytopenia 1	54.1%	21.6%	24.3%	37	20	8	9
Hematology	*MECOM*<br>Radioulnar synostosis with amegakaryocytic thrombocytopenia 2	54.1%	21.6%	24.3%	37	20	8	9
Hematology	*AP3B1*<br>Hermansky-Pudlak syndrome 2	54.1%	16.2%	29.7%	37	20	6	11
Endocrinology	*CA2*<br>Osteopetrosis with renal tubular acidosis	53.8%	20.5%	25.6%	39	21	8	10
Endocrinology	*TNFRSF11A*<br>Osteopetrosis type 7	53.8%	23.1%	23.1%	39	21	9	9
Oncology	*SUFU*<br>Medulloblastoma	53.8%	26.9%	19.2%	52	28	14	10
Endocrinology	*SLC16A1*<br>Familial hyperinsulinemic hypoglycemia 7	53.7%	22.0%	24.4%	41	22	9	10
Immunology	*IFNGR2*<br>Immunodeficiency 27A	53.3%	10.0%	36.7%	30	16	3	11
Immunology	*UNG*<br>Immunodeficiency with hyper IgM, type 5	53.3%	16.7%	30.0%	30	16	5	9
Immunology	*MS4A1*<br>Common variable immune deficiency 5	53.3%	30.0%	16.7%	30	16	9	5
Immunology	*CORO1A*<br>Immunodeficiency 8	53.1%	12.5%	34.4%	32	17	4	11
Immunology	*PIK3CD*<br>Immunodeficiency 14	53.1%	12.5%	34.4%	32	17	4	11
Neurology	*CHRNB1*<br>Congenital myasthenic syndrome 2	53.1%	24.5%	22.4%	49	26	12	11
Neurology	*CHRND*<br>Congenital myasthenic syndrome 3	53.1%	24.5%	22.4%	49	26	12	11

© 2023 Gold NB et al. *JAMA Network Open.*

Clinical Category	Gene-disease pairs	Yes (%)	No (%)	Unsure (%)	n	Yes (n)	No (n)	Unsure (n)
Neurology	*CHRNE*<br>Congenital myasthenic syndrome 4	53.1%	24.5%	22.4%	49	26	12	11
Neurology	*SYT2*<br>Congenital myasthenic syndrome 7	53.1%	24.5%	22.4%	49	26	12	11
Neurology	*MUSK*<br>Congenital myasthenic syndrome 9	53.1%	24.5%	22.4%	49	26	12	11
Neurology	*RAPSN*<br>Congenital myasthenic syndrome 11	53.1%	24.5%	22.4%	49	26	12	11
Neurology	*GFPT1*<br>Congenital myasthenic syndrome 12	53.1%	24.5%	22.4%	49	26	12	11
Neurology	*ALG14*<br>Congenital myasthenic syndrome 15	53.1%	24.5%	22.4%	49	26	12	11
Neurology	*LRP4*<br>Congenital myasthenic syndrome 17	53.1%	24.5%	22.4%	49	26	12	11
Neurology	*SNAP25*<br>Congenital myasthenic syndrome 18	53.1%	24.5%	22.4%	49	26	12	11
Neurology	*COL13A1*<br>Congenital myasthenic syndrome 19	53.1%	24.5%	22.4%	49	26	12	11
Neurology	*SLC18A3*<br>Congenital myasthenic syndrome 21	53.1%	24.5%	22.4%	49	26	12	11
Oncology	*PTCH1*<br>Medulloblastoma	52.8%	26.4%	20.8%	53	28	14	11
Gastroenterology	*MTTP*<br>Abetalipoproteinemia	52.8%	22.2%	25.0%	36	19	8	9
Endocrinology	*CLCN2*<br>Familial hyperaldosteronism, Type II	52.6%	23.7%	23.7%	38	20	9	9
Endocrinology	*KCNJ5*<br>Familial hyperaldosteronism, Type III	52.6%	23.7%	23.7%	38	20	9	9
Endocrinology	*AAAS*<br>Achalasia-addisonianism-alacrimia syndrome	52.6%	15.8%	31.6%	38	20	6	12

© 2023 Gold NB et al. *JAMA Network Open.*

Clinical Category	Gene-disease pairs	Yes (%)	No (%)	Unsure (%)	n	Yes (n)	No (n)	Unsure (n)
Metabolism	*SLC35A2*<br>Congenital disorder of glycosylation, type IIm	52.6%	22.8%	24.6%	57	30	13	14
Metabolism	*GLDC*<br>Glycine decarboxylase (GLDC) deficiency	52.6%	26.3%	21.1%	57	30	15	12
Metabolism	*PPT1*<br>Ceroid lipofuscinosis, neuronal, 1	52.5%	23.7%	23.7%	59	31	14	14
Endocrinology	*HK1*<br>HK1 associated hyperinsulinism	52.5%	22.5%	25.0%	40	21	9	10
Hematology	*ALAS2*<br>X-linked erythropoietic protoporphyria	52.5%	12.5%	35.0%	40	21	5	14
Metabolism	*MTHFR*<br>Methylenetetrahydrofolate reductase deficiency	52.5%	32.8%	14.8%	61	32	20	9
Neurology	*FLAD1*<br>Lipid storage myopathy due to flavin adenine dinucleotide synthetase deficiency	52.1%	14.6%	33.3%	48	25	7	16
Neurology	*SLC52A3*<br>Brown-Vialetto-Van Laere syndrome 1	52.0%	20.0%	28.0%	50	26	10	14
Neurology	*SLC52A2*<br>Brown-Vialetto-Van Laere syndrome 2	52.0%	20.0%	28.0%	50	26	10	14
Neurology	*CACNA1S*<br>Hypokalemic periodic paralysis type 1	52.0%	24.0%	24.0%	50	26	12	12
Metabolism	*SLC39A8*<br>Congenital disorder of glycosylation, type IIn	51.7%	20.7%	27.6%	58	30	12	16
Metabolism	*MTHFS*<br>5,10-Methenyltetrahydrofolate synthetase deficiency	51.7%	24.1%	24.1%	58	30	14	14
Immunology	*CD3D*<br>Immunodeficiency 19	51.6%	9.7%	38.7%	31	16	3	12

© 2023 Gold NB et al. *JAMA Network Open.*

Clinical Category	Gene-disease pairs	Yes (%)	No (%)	Unsure (%)	n	Yes (n)	No (n)	Unsure (n)
Immunology	*IL2RB* Immunodeficiency 63 with lymphoproliferation and autoimmunity	51.6%	12.9%	35.5%	31	16	4	11
Immunology	*TNFRSF13C* Common variable immune deficiency 4	51.6%	35.5%	12.9%	31	16	11	4
Immunology	*CR2* Common variable immune deficiency 7	51.6%	29.0%	19.4%	31	16	9	6
Immunology	*ITK* Lymphoproliferative syndrome 1	51.6%	19.4%	29.0%	31	16	6	9
Immunology	*CD27* Lymphoproliferative syndrome 2	51.6%	19.4%	29.0%	31	16	6	9
Immunology	*CD70* Lymphoproliferative syndrome 3	51.6%	19.4%	29.0%	31	16	6	9
Immunology	*RAB27A* Griscelli syndrome, type 2	51.6%	19.4%	29.0%	31	16	6	9
Immunology	*RFXANK* MHC class II deficiency, complementation group B	51.6%	12.9%	35.5%	31	16	4	11
Immunology	*CXCR4* WHIM syndrome	51.6%	12.9%	35.5%	31	16	4	11
Hematology	*CBLIF* Intrinsic factor deficiency	51.4%	20.0%	28.6%	35	18	7	10
Endocrinology	*PTF1A* Pancreatic agenesis 2	51.3%	20.5%	28.2%	39	20	8	11
Endocrinology	*SNX10* Osteopetrosis type 8	51.3%	25.6%	23.1%	39	20	10	9
Endocrinology	*FOXA2* FOXA2 associated hyperinsulinism	51.2%	22.0%	26.8%	41	21	9	11
Metabolism	*GALM* Galactose mutarotase deficiency	50.9%	14.0%	35.1%	57	29	8	20

© 2023 Gold NB et al. *JAMA Network Open.*

Clinical Category	Gene-disease pairs	Yes (%)	No (%)	Unsure (%)	n	Yes (n)	No (n)	Unsure (n)
Metabolism	*MT-TL1*<br>MELAS (Myopathy, Encephalopathy, Lactic Acidosis, and Stroke-like episodes)	50.8%	37.3%	11.9%	59	30	22	7
Metabolism	*ECHS1*<br>Mitochondrial short-chain enoyl-CoA hydratase-1 deficiency	50.8%	28.8%	20.3%	59	30	17	12
Endocrinology	*GATA4*<br>GATA4 associated diabetes	50.0%	28.6%	21.4%	42	21	12	9
Endocrinology	*CACNA1H*<br>Familial hyperaldosteronism, Type IV	50.0%	26.3%	23.7%	38	19	10	9
Gastroenterology	*LARS1*<br>LARS1 associated Infantile liver failure syndrome 1	50.0%	13.9%	36.1%	36	18	5	13
Hematology	*FECH*<br>Erythropoietic protoporphyria 1	50.0%	15.0%	35.0%	40	20	6	14
Hematology	*FGA*<br>FGA associated afibrinogenemia	50.0%	19.4%	30.6%	36	18	7	11
Hematology	*FGB*<br>FGB associate afibrinogenemia	50.0%	19.4%	30.6%	36	18	7	11
Hematology	*FGG*<br>FGG associated afibrinogenemia	50.0%	19.4%	30.6%	36	18	7	11
Hematology	*PIK3CA*<br>PIK3CA related overgrowth spectrum	50.0%	31.6%	18.4%	38	19	12	7
Immunology	*CD3E*<br>Immunodeficiency 18	50.0%	9.4%	40.6%	32	16	3	13
Immunology	*CD247*<br>Immunodeficiency 25	50.0%	6.7%	43.3%	30	15	2	13
Immunology	*DNMT3B*<br>Immunodeficiency-centromeric instability-facial anomalies syndrome 1	50.0%	13.3%	36.7%	30	15	4	11

© 2023 Gold NB et al. *JAMA Network Open.*

Clinical Category	Gene-disease pairs	Yes (%)	No (%)	Unsure (%)	n	Yes (n)	No (n)	Unsure (n)
Immunology	*TRNT1*<br>Sideroblastic anemia with B-cell immunodeficiency, periodic fevers, and developmental delay	50.0%	16.7%	33.3%	30	15	5	10
Metabolism	*FUCA1*<br>Fucosidosis	50.0%	33.3%	16.7%	60	30	20	10
Metabolism	*TMEM165*<br>Congenital disorder of glycosylation, type IIk	50.0%	22.4%	27.6%	58	29	13	16
Metabolism	*SLC30A10*<br>Hypermagnesemia with dystonia 1	50.0%	17.2%	32.8%	58	29	10	19
Metabolism	*SLC39A14*<br>Hypermagnesemia with dystonia 2	50.0%	19.0%	31.0%	58	29	11	18
Metabolism	*APRT*<br>Adenine phosphoribosyltransferase deficiency	50.0%	14.3%	35.7%	56	28	8	20
Metabolism	*GLUL*<br>Glutamine synthetase deficiency	50.0%	20.7%	29.3%	58	29	12	17
Nephrology	*SGPL1*<br>Nephrotic syndrome, type 14	50.0%	28.6%	21.4%	28	14	8	6
Metabolism	*HIBCH*<br>3-hydroxyisobutryl-CoA hydrolase deficiency	49.1%	24.6%	26.3%	57	28	14	15
Metabolism	*PHGDH*<br>Phosphoglycerate dehydrogenase deficiency	49.1%	21.1%	29.8%	57	28	12	17
Neurology	*KCNQ2*<br>Early infantile epileptic encephalopathy 7	49.0%	28.6%	22.4%	49	24	14	11
Endocrinology	*AKT2*<br>Hypoinsulinemic hypoglycemia	48.8%	24.4%	26.8%	41	20	10	11
Endocrinology	*UCP2*<br>UCP2 associated hyperinsulinism	48.7%	23.1%	28.2%	39	19	9	11

© 2023 Gold NB et al. *JAMA Network Open.*

Clinical Category	Gene-disease pairs	Yes (%)	No (%)	Unsure (%)	n	Yes (n)	No (n)	Unsure (n)
Endocrinology	*AGPAT2*<br>Congenital generalized lipodystrophy type 1	48.7%	28.2%	23.1%	39	19	11	9
Endocrinology	*BSCL2*<br>Congenital generalized lipodystrophy type 2	48.7%	25.6%	25.6%	39	19	10	10
Hematology	*UROD*<br>Porphyria cutanea tarda	48.7%	17.9%	33.3%	39	19	7	13
Hematology	*HMBS*<br>Acute intermittent porphyria	48.7%	17.9%	33.3%	39	19	7	13
Endocrinology	*SCNN1B*<br>SCNN1B associated pseudohypoaldosteronism, type I	48.6%	21.6%	29.7%	37	18	8	11
Immunology	*LCK*<br>Immunodeficiency 22	48.4%	12.9%	38.7%	31	15	4	12
Immunology	*PGM3*<br>Immunodeficiency 23	48.4%	12.9%	38.7%	31	15	4	12
Immunology	*TYK2*<br>Immunodeficiency 35	48.4%	22.6%	29.0%	31	15	7	9
Immunology	*RELB*<br>Immunodeficiency 53	48.4%	12.9%	38.7%	31	15	4	12
Immunology	*ZBTB24*<br>Immunodeficiency-centromeric instability-facial anomalies syndrome 2	48.4%	16.1%	35.5%	31	15	5	11
Immunology	*LAMTOR2*<br>MAPBP-interacting protein associated immunodeficiency	48.4%	22.6%	29.0%	31	15	7	9
Immunology	*MAP3K14*<br>MAP3K14 associated immunodeficiency	48.4%	16.1%	35.5%	31	15	5	11

© 2023 Gold NB et al. *JAMA Network Open.*

Clinical Category	Gene-disease pairs	Yes (%)	No (%)	Unsure (%)	n	Yes (n)	No (n)	Unsure (n)
Immunology	*MTHFD1*<br>Combined immunodeficiency and megaloblastic anemia with or without hyperhomocysteinemia	48.4%	12.9%	38.7%	31	15	4	12
Immunology	*TNFRSF13B*<br>Common variable immune deficiency 2	48.4%	38.7%	12.9%	31	15	12	4
Immunology	*PRKCD*<br>Autoimmune lymphoproliferative syndrome, type III	48.4%	25.8%	25.8%	31	15	8	8
Immunology	*KRT14*<br>Epidermolysis bullosa	48.4%	29.0%	22.6%	31	15	9	7
Immunology	*KRT5*<br>Epidermolysis bullosa	48.4%	29.0%	22.6%	31	15	9	7
Immunology	*IL10*<br>Interleukin-10 deficiency	48.4%	16.1%	35.5%	31	15	5	11
Immunology	*NLRC4*<br>NLRC4 associated familial cold inflammatory syndrome	48.4%	19.4%	32.3%	31	15	6	10
Immunology	*IRAK4*<br>IRAK4 deficiency	48.4%	12.9%	38.7%	31	15	4	12
Immunology	*MYD88*<br>MYD88 deficiency	48.4%	22.6%	29.0%	31	15	7	9
Immunology	*PAX1*<br>Otofaciocervical syndrome 2	48.4%	19.4%	32.3%	31	15	6	10
Immunology	*NFE2L2*<br>NRF2 superactivity (immunodeficiency, developmental delay, and hypohomocysteinemia)	48.3%	20.7%	31.0%	29	14	6	9
Immunology	*AMN*<br>Imerslund-Grasbeck syndrome 2	48.3%	13.8%	37.9%	29	14	4	11
Metabolism	*MFSD8*<br>Ceroid lipofuscinosis, neuronal, 7	48.3%	25.9%	25.9%	58	28	15	15

© 2023 Gold NB et al. *JAMA Network Open.*

Clinical Category	Gene-disease pairs	Yes (%)	No (%)	Unsure (%)	n	Yes (n)	No (n)	Unsure (n)
Metabolism	*ETHE1*<br>Mitochondrial sulfur dioxygenase deficiency	48.3%	25.9%	25.9%	58	28	15	15
Metabolism	*TRPM6*<br>TRPM6 associated hypomagnesemia	48.3%	15.5%	36.2%	58	28	9	21
Metabolism	*PIGA*<br>PIGA-CDG	48.3%	32.8%	19.0%	58	28	19	11
Metabolism	*PIGM*<br>PIGM-CDG	48.3%	32.8%	19.0%	58	28	19	11
Neurology	*SCN1A*<br>Early infantile epileptic encephalopathy 6	47.9%	31.3%	20.8%	48	23	15	10
Metabolism	*MT-ND1*<br>MELAS (Myopathy, Encephalopathy, Lactic Acidosis, and Stroke-like episodes)	47.5%	40.7%	11.9%	59	28	24	7
Metabolism	*MT-ND4*<br>MELAS (Myopathy, Encephalopathy, Lactic Acidosis, and Stroke-like episodes)	47.5%	40.7%	11.9%	59	28	24	7
Metabolism	*MT-ND5*<br>MELAS (Myopathy, Encephalopathy, Lactic Acidosis, and Stroke-like episodes)	47.5%	40.7%	11.9%	59	28	24	7
Metabolism	*MT-ND6*<br>MELAS (Myopathy, Encephalopathy, Lactic Acidosis, and Stroke-like episodes)	47.5%	40.7%	11.9%	59	28	24	7
Metabolism	*MT-TF*<br>MELAS (Myopathy, Encephalopathy, Lactic Acidosis, and Stroke-like episodes)	47.5%	40.7%	11.9%	59	28	24	7
Metabolism	*MT-TH*<br>MELAS (Myopathy, Encephalopathy, Lactic Acidosis, and Stroke-like episodes)	47.5%	40.7%	11.9%	59	28	24	7
Metabolism	*MT-TQ*<br>MELAS (Myopathy, Encephalopathy, Lactic Acidosis, and Stroke-like episodes)	47.5%	40.7%	11.9%	59	28	24	7

© 2023 Gold NB et al. *JAMA Network Open.*

Clinical Category	Gene-disease pairs	Yes (%)	No (%)	Unsure (%)	n	Yes (n)	No (n)	Unsure (n)
Metabolism	*MT-TS1* MELAS (Myopathy, Encephalopathy, Lactic Acidosis, and Stroke-like episodes)	47.5%	40.7%	11.9%	59	28	24	7
Metabolism	*MT-TS2* MELAS (Myopathy, Encephalopathy, Lactic Acidosis, and Stroke-like episodes)	47.5%	40.7%	11.9%	59	28	24	7
Metabolism	*MT-TW* MELAS (Myopathy, Encephalopathy, Lactic Acidosis, and Stroke-like episodes)	47.5%	40.7%	11.9%	59	28	24	7
Endocrinology	*WNK4* Pseudohypoaldosteronism, type IIB	47.4%	23.7%	28.9%	38	18	9	11
Endocrinology	*WNK1* Pseudohypoaldosteronism, type IIC	47.4%	23.7%	28.9%	38	18	9	11
Endocrinology	*KLHL3* Pseudohypoaldosteronism, type IID	47.4%	23.7%	28.9%	38	18	9	11
Endocrinology	*CUL3* Pseudohypoaldosteronism, type IIE	47.4%	23.7%	28.9%	38	18	9	11
Endocrinology	*NR3C2* NR3C2 associated pseudohypoaldosteronism, type I	47.4%	23.7%	28.9%	38	18	9	11
Endocrinology	*SCNN1A* SCNN1A associated pseudohypoaldosteronism, type I	47.4%	23.7%	28.9%	38	18	9	11
Endocrinology	*SCNN1G* SCNN1G associated pseudohypoaldosteronism, type I	47.4%	23.7%	28.9%	38	18	9	11
Endocrinology	*GPR101* Growth hormone-secreting pituitary adenoma 2	47.4%	26.3%	26.3%	38	18	10	10
Metabolism	*FXYD2* Hypomagnesemia, type 2	47.4%	19.3%	33.3%	57	27	11	19

© 2023 Gold NB et al. *JAMA Network Open.*

Clinical Category	Gene-disease pairs	Yes (%)	No (%)	Unsure (%)	n	Yes (n)	No (n)	Unsure (n)
Metabolism	*SORD*<br>Sorbitol dehydrogenase deficiency with peripheral neuropathy	47.4%	19.3%	33.3%	57	27	11	19
Hematology	*SLC25A38*<br>Pyridoxine-refractory sideroblastic anemia 2	47.2%	22.2%	30.6%	36	17	8	11
Neurology	*SCN8A*<br>Early infantile epileptic encephalopathy 13	46.9%	30.6%	22.4%	49	23	15	11
Neurology	*KCNT1*<br>Early infantile epileptic encephalopathy 14	46.9%	30.6%	22.4%	49	23	15	11
Immunology	*STIM1*<br>Immunodeficiency 10	46.9%	12.5%	40.6%	32	15	4	13
Immunology	*RASGRP1*<br>Immunodeficiency 64	46.7%	16.7%	36.7%	30	14	5	11
Metabolism	*MT-CO3*<br>MELAS (Myopathy, Encephalopathy, Lactic Acidosis, and Stroke-like episodes)	46.6%	39.7%	13.8%	58	27	23	8
Metabolism	*GOT2*<br>Glutamic-oxaloacetic transaminase 2 deficiency	46.4%	17.9%	35.7%	56	26	10	20
Endocrinology	*CAV1*<br>Congenital generalized lipodystrophy type 3	46.2%	25.6%	28.2%	39	18	10	11
Hematology	*ALAD*<br>Aminolevulinic acid dehydratase deficiency porphyria	46.2%	12.8%	41.0%	39	18	5	16
Hematology	*CPOX*<br>Coproporphyria	46.2%	17.9%	35.9%	39	18	7	14
Hematology	*PPOX*<br>Variegate porphyria	45.9%	18.9%	35.1%	37	17	7	13

© 2023 Gold NB et al. *JAMA Network Open.*

Clinical Category	Gene-disease pairs	Yes (%)	No (%)	Unsure (%)	n	Yes (n)	No (n)	Unsure (n)
Metabolism	*MT-CO1*<br>MELAS (Myopathy, Encephalopathy, Lactic Acidosis, and Stroke-like episodes)	45.8%	40.7%	13.6%	59	27	24	8
Metabolism	*MT-CPO2*<br>MELAS (Myopathy, Encephalopathy, Lactic Acidosis, and Stroke-like episodes)	45.8%	40.7%	13.6%	59	27	24	8
Metabolism	*PIGO*<br>PIGO-CDG	45.6%	35.1%	19.3%	57	26	20	11
Metabolism	*SLC30A2*<br>Transient neonatal zinc deficiency	45.6%	22.8%	31.6%	57	26	13	18
Metabolism	*ALDH4A1*<br>Hyperprolinemia, type II	45.6%	24.6%	29.8%	57	26	14	17
Metabolism	*POR*<br>Cytochrome P450 oxidoreductase deficiency	45.6%	26.3%	28.1%	57	26	15	16
Immunology	*CTPS1*<br>Immunodeficiency 24	45.2%	9.7%	45.2%	31	14	3	14
Immunology	*IRF8*<br>Immunodeficiency 32B	45.2%	16.1%	38.7%	31	14	5	12
Immunology	*IL21R*<br>Immunodeficiency 56	45.2%	16.1%	38.7%	31	14	5	12
Immunology	*CUBN*<br>Imerslund-Grasbeck syndrome 1	45.2%	16.1%	38.7%	31	14	5	12
Immunology	*CFHR1*<br>CFHR1 associated susceptibility to atypical hemolytic uremic syndrome	45.2%	22.6%	32.3%	31	14	7	10
Immunology	*COL7A1*<br>Epidermolysis bullosa	45.2%	29.0%	25.8%	31	14	9	8
Immunology	*NLRP12*<br>Familial cold autoinflammatory syndrome 2	45.2%	22.6%	32.3%	31	14	7	10

© 2023 Gold NB et al. *JAMA Network Open.*

Clinical Category	Gene-disease pairs	Yes (%)	No (%)	Unsure (%)	n	Yes (n)	No (n)	Unsure (n)
Immunology	*PSTPIP1*<br>PSTPIP1 associated inflammatory disease	45.2%	25.8%	29.0%	31	14	8	9
Immunology	*SMARCD2*<br>Specific granule deficiency 2	45.2%	16.1%	38.7%	31	14	5	12
Immunology	*TNFRSF1A*<br>Tumor necrosis factor receptor associated periodic syndrome	45.2%	22.6%	32.3%	31	14	7	10
Immunology	*ARPC1B*<br>Platelet abnormalities with eosinophilia and immune-mediated inflammatory disease	45.2%	22.6%	32.3%	31	14	7	10
Immunology	*MEFV*<br>Familial Mediterranean fever	45.2%	29.0%	25.8%	31	14	9	8
Immunology	*PARN*<br>Dyskeratosis congenita, autosomal recessive 6	45.2%	29.0%	25.8%	31	14	9	8
Neurology	*SCN2A*<br>Early infantile epileptic encephalopathy 11	44.9%	32.7%	22.4%	49	22	16	11
Neurology	*SLC13A5*<br>Early infantile epileptic encephalopathy 25	44.9%	28.6%	26.5%	49	22	14	13
Nephrology	*CA12*<br>Isolated hyperchlorhidrosis	44.8%	20.7%	34.5%	29	13	6	10
Endocrinology	*CAVIN1*<br>Congenital generalized lipodystrophy type 4	44.7%	26.3%	28.9%	38	17	10	11
Endocrinology	*PAPPA2*<br>PAPPA2 associated short stature	44.7%	23.7%	31.6%	38	17	9	12
Endocrinology	*FOXE1*<br>Bamforth-Lazarus syndrome	44.7%	23.7%	31.6%	38	17	9	12
Hematology	*TF*<br>Atransferrinemia	44.4%	25.0%	30.6%	36	16	9	11

© 2023 Gold NB et al. *JAMA Network Open.*

Clinical Category	Gene-disease pairs	Yes (%)	No (%)	Unsure (%)	n	Yes (n)	No (n)	Unsure (n)
Endocrinology	*PDX1*<br>Maturity-onset diabetes of the young, type 4	44.2%	37.2%	18.6%	43	19	16	8
Neurology	*NF1*<br>Neurofibromatosis type 1	44.0%	40.0%	16.0%	50	22	20	8
Metabolism	*PSAT1*<br>Phosphoserine aminotransferase deficiency	43.9%	15.8%	40.4%	57	25	9	23
Immunology	*ORAI1*<br>Immunodeficiency 9	43.8%	12.5%	43.8%	32	14	4	14
Immunology	*MALT1*<br>Immunodeficiency 12	43.8%	21.9%	34.4%	32	14	7	11
Neurology	*CLCN1*<br>Myotonia congenita	43.8%	35.4%	20.8%	48	21	17	10
Endocrinology	*RFX6*<br>Mitchell-Riley syndrome	43.6%	23.1%	33.3%	39	17	9	13
Endocrinology	*FAM111A*<br>Kenny-Caffey syndrome, type 2	43.6%	23.1%	33.3%	39	17	9	13
Immunology	*MCM4*<br>Immunodeficiency 54	43.3%	20.0%	36.7%	30	13	6	11
Immunology	*CDCA7*<br>Immunodeficiency-centromeric instability-facial anomalies syndrome 3	43.3%	13.3%	43.3%	30	13	4	13
Immunology	*HELLS*<br>Immunodeficiency-centromeric instability-facial anomalies syndrome 4	43.3%	16.7%	40.0%	30	13	5	12
Metabolism	*IARS1*<br>Isoleucyl-tRNA synthetase deficiency	43.1%	20.7%	36.2%	58	25	12	21
Endocrinology	*NEUROD1*<br>Maturity-onset diabetes of the young, type 6	42.9%	40.5%	16.7%	42	18	17	7

© 2023 Gold NB et al. *JAMA Network Open.*

Clinical Category	Gene-disease pairs	Yes (%)	No (%)	Unsure (%)	n	Yes (n)	No (n)	Unsure (n)
Neurology	*CAD*<br>Early infantile epileptic encephalopathy 50	42.9%	32.7%	24.5%	49	21	16	12
Cardiovascular	*SMAD4*<br>Myhre syndrome	42.6%	39.3%	18.0%	61	26	24	11
Cardiovascular	*TTR*<br>Transthyretin associated hereditary amyloidosis	42.6%	36.1%	21.3%	61	26	22	13
Neurology	*CLCN7*<br>Osteopetrosis type 4	42.6%	34.0%	23.4%	47	20	16	11
Metabolism	*PSPH*<br>Phosphoserine phosphatase deficiency	42.1%	15.8%	42.1%	57	24	9	24
Neurology	*RNASEH2A*<br>Aicardi-Goutieres syndrome 4	42.0%	32.0%	26.0%	50	21	16	13
Immunology	*IL17RA*<br>Immunodeficiency 30	41.9%	25.8%	32.3%	31	13	8	10
Immunology	*C5*<br>C5 deficiency	41.9%	22.6%	35.5%	31	13	7	11
Immunology	*USP18*<br>Pseudo-TORCH syndrome 2	41.9%	19.4%	38.7%	31	13	6	12
Immunology	*ACP5*<br>Spondyloenchondrodysplasia with ACP5 immune dysregulation	41.9%	22.6%	35.5%	31	13	7	11
Immunology	*CARD14*<br>Pityriasis rubra pilaris	41.9%	25.8%	32.3%	31	13	8	10
Immunology	*LPIN2*<br>Majeed syndrome	41.9%	16.1%	41.9%	31	13	5	13
Immunology	*NLRP3*<br>Cryopyrin associated periodic fever syndrome	41.9%	32.3%	25.8%	31	13	10	8

© 2023 Gold NB et al. *JAMA Network Open.*

Clinical Category	Gene-disease pairs	Yes (%)	No (%)	Unsure (%)	n	Yes (n)	No (n)	Unsure (n)
Immunology	*PLCG2*<br>Autoinflammation and PLCG2 associated antibody deficiency and immune dysregulation (APLAID)	41.9%	25.8%	32.3%	31	13	8	10
Endocrinology	*INS*<br>Maturity-onset diabetes of the young, type 10	41.9%	41.9%	16.3%	43	18	18	7
Oncology	*BMPR1A*<br>BMPR1A associated juvenile polyposis syndrome	41.5%	37.7%	20.8%	53	22	20	11
Ophthalmology	*VAMP1*<br>Congenital myasthenic syndrome 25	41.2%	11.8%	47.1%	34	14	4	16
Endocrinology	ABCC9<br>ABCC9 associated hypertrichotic osteochondrodysplasia	41.0%	23.1%	35.9%	39	16	9	14
Endocrinology	*KCNJ8*<br>KCNJ8 associated hypertrichotic osteochondrodysplasia	41.0%	23.1%	35.9%	39	16	9	14
Neurology	*LSM11*<br>Aicardi-Goutieres syndrome 8	40.8%	34.7%	24.5%	49	20	17	12
Hematology	*HAMP*<br>Hemochromatosis, type 2B	40.5%	24.3%	35.1%	37	15	9	13
Metabolism	*AGA*<br>Aspartylglucosaminidase deficiency	40.4%	26.3%	33.3%	57	23	15	19
Gastroenterology	*IL10RA*<br>Inflammatory bowel disease 25	40.0%	22.9%	37.1%	35	14	8	13
Gastroenterology	*IL10RB*<br>Inflammatory bowel disease 28	40.0%	22.9%	37.1%	35	14	8	13
Gastroenterology	*GPIHBP1*<br>Glycosylphosphatidylinositol-anchored high-density lipoprotein-binding protein 1 (GPIHBP1) deficiency	40.0%	14.3%	45.7%	35	14	5	16

© 2023 Gold NB et al. *JAMA Network Open.*

Clinical Category	Gene-disease pairs	Yes (%)	No (%)	Unsure (%)	n	Yes (n)	No (n)	Unsure (n)
Immunology	*CDKN1C*<br>IMAGE syndrome	40.0%	13.3%	46.7%	30	12	4	14
Immunology	*MARS1*<br>MARS1 associated interstitial lung and liver disease	40.0%	23.3%	36.7%	30	12	7	11
Neurology	*TREX1*<br>Aicardi-Goutieres syndrome 1	40.0%	34.0%	26.0%	50	20	17	13
Neurology	*RNASEH2B*<br>Aicardi-Goutieres syndrome 2	40.0%	34.0%	26.0%	50	20	17	13
Neurology	*RNASEH2C*<br>Aicardi-Goutieres syndrome 3	40.0%	34.0%	26.0%	50	20	17	13
Neurology	*ADAR*<br>Aicardi-Goutieres syndrome 6	40.0%	34.0%	26.0%	50	20	17	13
Neurology	*IFIH1*<br>Aicardi-Goutieres syndrome 7	40.0%	34.0%	26.0%	50	20	17	13
Neurology	*RNU7-1*<br>Aicardi-Goutieres syndrome 9	40.0%	34.0%	26.0%	50	20	17	13
Endocrinology	*IGFALS*<br>Acid-labile subunit deficiency	39.5%	26.3%	34.2%	38	15	10	13
Neurology	*SAMHD1*<br>Aicardi-Goutieres syndrome 5	38.8%	34.7%	26.5%	49	19	17	13
Immunology	*C3*<br>C3 deficiency	38.7%	25.8%	35.5%	31	12	8	11
Immunology	*IL1RN*<br>Interleukin 1 receptor antagonist deficiency	38.7%	25.8%	35.5%	31	12	8	11
Immunology	*TNFAIP3*<br>TNFAIP3 associated autoinflammatory syndrome	38.7%	22.6%	38.7%	31	12	7	12
Immunology	*CFP*<br>X-linked properdin deficiency	38.7%	19.4%	41.9%	31	12	6	13

© 2023 Gold NB et al. *JAMA Network Open.*

Clinical Category	Gene-disease pairs	Yes (%)	No (%)	Unsure (%)	n	Yes (n)	No (n)	Unsure (n)
Immunology	*NIPAL4*<br>Ichthyosis, congenital, autosomal recessive 6	38.7%	29.0%	32.3%	31	12	9	10
Neurology	*GLRB*<br>Hyperekplexia 2	38.0%	30.0%	32.0%	50	19	15	16
Hematology	*HJV*<br>Hemochromatosis, type 2A	37.8%	24.3%	37.8%	37	14	9	14
Neurology	*CHD7*<br>CHARGE syndrome	37.5%	43.8%	18.8%	48	18	21	9
Gastroenterology	*SAR1B*<br>Chylomicron retention disease	37.1%	14.3%	48.6%	35	13	5	17
Endocrinology	*EIF2AK3*<br>Wolcott-Rallison syndrome	36.8%	23.7%	39.5%	38	14	9	15
Neurology	*SCARB2*<br>Progressive myoclonic epilepsy 4	36.7%	32.7%	30.6%	49	18	16	15
Neurology	*SLC25A12*<br>Mitochondrial aspartate-glutamate carrier isoform 1 deficiency (aralar deficiency)	36.7%	30.6%	32.7%	49	18	15	16
Immunology	*NOD2*<br>Blau syndrome	36.7%	33.3%	30.0%	30	11	10	9
Immunology	*OTULIN*<br>OTULIN deficiency	36.7%	30.0%	33.3%	30	11	9	10
Neurology	*FARSB*<br>Autosomal recessive aminoacyl transfer	36.2%	25.5%	38.3%	47	17	12	18
Neurology	*CACNA1A*<br>Episodic ataxia, type 2	36.0%	30.0%	34.0%	50	18	15	17
Neurology	*GLRA1*<br>Hyperekplexia 1	36.0%	32.0%	32.0%	50	18	16	16
Endocrinology	*PAX4*<br>Maturity-onset diabetes of the young, type 9	35.7%	47.6%	16.7%	42	15	20	7

© 2023 Gold NB et al. *JAMA Network Open.*

Clinical Category	Gene-disease pairs	Yes (%)	No (%)	Unsure (%)	n	Yes (n)	No (n)	Unsure (n)
Metabolism	*NAXE*<br>NAD(P)HX epimerase deficiency	35.7%	21.4%	42.9%	56	20	12	24
Immunology	*C1QA*<br>C1QA associated C1q deficiency	35.5%	29.0%	35.5%	31	11	9	11
Immunology	*C1QC*<br>C1QC associated C1q deficiency	35.5%	29.0%	35.5%	31	11	9	11
Immunology	*C2*<br>C2 deficiency	35.5%	29.0%	35.5%	31	11	9	11
Immunology	*C6*<br>C6 deficiency	35.5%	29.0%	35.5%	31	11	9	11
Immunology	*C7*<br>C7 deficiency	35.5%	29.0%	35.5%	31	11	9	11
Immunology	*C8A*<br>C8 deficiency, type I	35.5%	29.0%	35.5%	31	11	9	11
Immunology	*C8B*<br>C8 deficiency, type II	35.5%	29.0%	35.5%	31	11	9	11
Immunology	*C9*<br>C9 deficiency	35.5%	25.8%	38.7%	31	11	8	12
Immunology	*CD46*<br>Susceptibility to atypical hemolytic uremic syndrome 2	35.5%	29.0%	35.5%	31	11	9	11
Immunology	*THBD*<br>Susceptibility to atypical hemolytic uremic syndrome 6	35.5%	29.0%	35.5%	31	11	9	11
Immunology	*CFB*<br>Complement factor B deficiency	35.5%	29.0%	35.5%	31	11	9	11
Immunology	*CFD*<br>Complement factor D deficiency	35.5%	29.0%	35.5%	31	11	9	11
Immunology	*CFH*<br>Complement factor H deficiency	35.5%	29.0%	35.5%	31	11	9	11
Immunology	*CFI*<br>Complement factor I deficiency	35.5%	29.0%	35.5%	31	11	9	11

© 2023 Gold NB et al. *JAMA Network Open.*

Clinical Category	Gene-disease pairs	Yes (%)	No (%)	Unsure (%)	n	Yes (n)	No (n)	Unsure (n)
Ophthalmology	*SLC6A6*<br>Taurine transporter deficiency	35.3%	5.9%	58.8%	34	12	2	20
Metabolism	*ABCG5*<br>Sitosterolemia 1	35.1%	26.3%	38.6%	57	20	15	22
Metabolism	*ABCG8*<br>Sitosterolemia 2	35.1%	26.3%	38.6%	57	20	15	22
Endocrinology	*KLF11*<br>Maturity-onset diabetes of the young, type 7	34.9%	48.8%	16.3%	43	15	21	7
Endocrinology	*CEL*<br>Maturity-onset diabetes of the young, type 8	34.9%	46.5%	18.6%	43	15	20	8
Neurology	*SLC6A5*<br>Hyperekplexia 3	34.7%	32.7%	32.7%	49	17	16	16
Neurology	*GRIN1*<br>Ionotropic glutamate receptor NMDA type subunit 1 dysregulation	34.7%	36.7%	28.6%	49	17	18	14
Neurology	*GRIN2D*<br>Ionotropic glutamate receptor NMDA type subunit 2D superactivity	34.7%	36.7%	28.6%	49	17	18	14
Neurology	*SARS1*<br>SARS1 associated neurodevelopmental disorder with microcephaly, ataxia, and seizures	34.7%	24.5%	40.8%	49	17	12	20
Oncology	*MUTYH*<br>Familial adenomatous polyposis 2	34.5%	49.1%	16.4%	55	19	27	9
Endocrinology	*APPL1*<br>Maturity-onset diabetes of the young, type 14	33.3%	45.2%	21.4%	42	14	19	9
Immunology	*C1QB*<br>C1QB associated C1q deficiency	33.3%	30.0%	36.7%	30	10	9	11

© 2023 Gold NB et al. *JAMA Network Open.*

Clinical Category	Gene-disease pairs	Yes (%)	No (%)	Unsure (%)	n	Yes (n)	No (n)	Unsure (n)
Immunology	*KDSR* Erythrokeratodermia variabilis et progressiva 4	33.3%	23.3%	43.3%	30	10	7	13
Metabolism	*AP1S1* MEDNIK syndrome	32.7%	23.6%	43.6%	55	18	13	24
Neurology	*SCN3A* Familial focal epilepsy with variable foci 4	32.7%	36.7%	30.6%	49	16	18	15
Neurology	*GRIN2A* Ionotropic glutamate receptor NMDA type subunit 2A dysregulation	32.7%	36.7%	30.6%	49	16	18	15
Neurology	*GRIN2B* Ionotropic glutamate receptor NMDA type subunit 2B dysregulation	32.7%	36.7%	30.6%	49	16	18	15
Neurology	*TMLHE* Epsilon-N-trimethyllysine hydroxylase deficiency	32.7%	30.6%	36.7%	49	16	15	18
Hematology	*TFR2* Hemochromatosis, type 3	32.4%	27.0%	40.5%	37	12	10	15
Hematology	*SLC40A1* Hemochromatosis, type 4	32.4%	27.0%	40.5%	37	12	10	15
Immunology	*IL36RN* Pustular psoriasis 14	32.3%	25.8%	41.9%	31	10	8	13
Neurology	*SLC1A3* Episodic ataxia, type 6	32.0%	32.0%	36.0%	50	16	16	18
Neurology	*PRRT2* Episodic kinesigenic dyskinesia 1	32.0%	30.0%	38.0%	50	16	15	19
Neurology	*PDGFRB* PDGFRB activating spectrum disorder	30.6%	30.6%	38.8%	49	15	15	19
Neurology	*PRPS1* Arts syndrome	30.6%	30.6%	38.8%	49	15	15	19
Hematology	*HFE* Hemochromatosis type 1	30.0%	30.0%	40.0%	40	12	12	16

© 2023 Gold NB et al. *JAMA Network Open.*

Clinical Category	Gene-disease pairs	Yes (%)	No (%)	Unsure (%)	n	Yes (n)	No (n)	Unsure (n)
Neurology	*KCNA1* Episodic ataxia/myokymia syndrome	30.0%	34.0%	36.0%	50	15	17	18
Ophthalmology	*PLG* Plasminogen deficiency, type I	29.4%	17.6%	52.9%	34	10	6	18
Neurology	*SLC18A2* Infantile parkinsonism-dystonia 2	29.2%	31.3%	39.6%	48	14	15	19
Oncology	*MSH2* Hereditary nonpolyposis colorectal cancer 1	27.8%	50.0%	22.2%	54	15	27	12
Oncology	*MLH1* Hereditary nonpolyposis colorectal cancer 2	27.8%	50.0%	22.2%	54	15	27	12
Oncology	*PMS2* Hereditary nonpolyposis colorectal cancer 4	27.8%	50.0%	22.2%	54	15	27	12
Oncology	*MSH6* Hereditary nonpolyposis colorectal cancer 5	27.8%	50.0%	22.2%	54	15	27	12
Cardiovascular	*APOC2* Apolipoprotein C-II (apoC-II) deficiency	26.2%	41.0%	32.8%	61	16	25	20
Gastroenterology	*IL12RB1* Inflammatory bowel disease 25, early onset, autosomal recessive	25.7%	28.6%	45.7%	35	9	10	16
Neurology	*GNE* GNE myopathy	25.0%	39.6%	35.4%	48	12	19	17
Cardiovascular	*APOA5* Apolipoprotein A-V deficiency	24.6%	42.6%	32.8%	61	15	26	20
Cardiovascular	*LMF1* Lipase maturation factor 1 (LMF1) deficiency	24.6%	34.4%	41.0%	61	15	21	25
Cardiovascular	*APOE* Apolipoprotein (apo) E	21.3%	59.0%	19.7%	61	13	36	12

© 2023 Gold NB et al. *JAMA Network Open.*

Clinical Category	Gene-disease pairs	Yes (%)	No (%)	Unsure (%)	n	Yes (n)	No (n)	Unsure (n)
Oncology	*EPCAM*<br>Hereditary nonpolyposis colorectal cancer 8	20.8%	52.8%	26.4%	53	11	28	14
Neurology	*SPTLC1*<br>Hereditary sensory neuropathy type IA	18.4%	36.7%	44.9%	49	9	18	22
Neurology	*SPTLC2*<br>Hereditary sensory neuropathy type IC	18.4%	36.7%	44.9%	49	9	18	22
Cardiovascular	*DBH*<br>Orthostatic hypotension 1	17.5%	49.2%	33.3%	63	11	31	21
Cardiovascular	*CYB561*<br>Orthostatic hypotension 2	17.5%	49.2%	33.3%	63	11	31	21

Excluded gene-disease pairs because of incorrect annotation in survey:

Clinical Category	Gene-disease pairs
Immunology	*SLC19A3*<br>Thiamine metabolism dysfunction syndrome 2
Immunology	*SLC35C1*<br>Congenital disorder of glycosylation type IIc (CDG2C)

© 2023 Gold NB et al. *JAMA Network Open.*

eTable 3. Description of Characteristics of Genes Included in Survey

Cardiovascular (17 genes)

Gene	Disease name	System	Inheritance	On RUSP? (Y/N)	Prevalence - disease frequency per 100,000 (Rx genes, if listed)	Age of Onset	Disease symptoms	Orthogonal test? (Y/N)	If yes, orthogonal test	Is orthogonal test expected to be abnormal in infancy?	Intervention Considered (Free Text)	Category of Intervention	Age of Intervention Implementation	MD leading intervention	Comments	Link to ref 1	Link to ref 2	Link to ref 3
APOA5	Apolipoprotein A-V deficiency	cardiovascular	AR	N	0.55	Childhood	Severe hypertriglyceridemia, episodes of abdominal pain, recurrent acute pancreatitis, eruptive cutaneous xanthomata, hepatosplenomegaly	Y	triglycerides		restriction of total dietary fat	diet	childhood	pediatric cardiology, nutrition				
APOC2	Apolipoprotein C-II (apoC-II) deficiency	cardiovascular	AR	N	0.55	Childhood or adolescence	Severe chylomicronemia	Y	triglycerides		restriction of total dietary fat, volanesorsen	diet medication	childhood	pediatric cardiology, nutrition		https://pubmed.ncbi.nlm.nih.gov/31390500/		
APOE	Apolipoprotein (apo) E (familial dysbetalipoproteinemia type III)	cardiovascular	AD	N		Adulthood	Cutaneous xanthomas, coronary artery disease, peripheral artery disease	Y	LDL, IDL -range AUC/LDL-range AUC ratio of > 0.5		exercise and diet, statins, ezetimibe	diet medication		pediatric cardiology		https://pubmed.ncbi.nlm.nih.gov/30731287/	https://www-ncbi-nlm-nih-gov.ezp-prod1.hul.harvard.edu/books/NBK567738/	
DBH	Orthostatic hypotension 1	cardiovascular	AR	N		Neonatal	Vomiting, dehydration, hypotension, hypothermia, hypoglycemia requiring repeated hospitalization, reduced exercise capacity, profound orthostatic hypotension, ptosis of the eyelids, nasal congestion	Y	Plasma norepinephrine, epinephrine, dopamine		droxidopa	medication	infancy	pediatric cardiology				
CYB561	Orthostatic hypotension 2	cardiovascular	AR	N		Infancy or early childhood	Severe symptomatic orthostatic hypotension without compensatory tachycardia, impaired renal function, mild anemia, episodic hypoglycemia	Y	Plasma norepinephrine, epinephrine, dopamine		droxidopa	medication	infancy	pediatric cardiology				
ENPP1	Generalized arterial calcification of infancy 1	cardiovascular	AR	N	0.5	Infancy	Widespread arterial calcification and/or narrowing of large and medium-sized vessels, cardiovascular findings including heart failure, respiratory distress, edema, cyanosis, hypertension (and/or cardiomegaly), skin and retinal manifestations of pseudoxanthoma elasticum, periarticular calcifications, development of rickets after infancy, cervical spine fusion, hearing loss	Y	CT scan	Y	bisphosphonates, calcitriol, phosphate supplements	medication	infancy	pediatric cardiology				
ABCC6	Generalized arterial calcification of infancy 2	cardiovascular	AR	N		Infancy	Widespread arterial calcification and/or narrowing of large and medium-sized vessels, cardiovascular findings (including heart failure, respiratory distress, edema, cyanosis, hypertension, and/or cardiomegaly), skin and retinal manifestations of pseudoxanthoma elasticum, periarticular calcifications, development of rickets after infancy, cervical spine fusion, hearing loss	Y	CT scan	Y	bisphosphonates (It remains unclear whether bisphosphonates (etidronate in particular) are associated with improved survival), calcitriol, phosphate supplements	medication	infancy	pediatric cardiology				
LDLR	Familial hypercholesterolemia 1	cardiovascular	AD, AR	N	416.5	Childhood	Elevated LDL, plaque deposition in the coronary arteries and proximal aorta at an early age	Y	LDL		diet and exercise, statins (8 years)	diet medication	childhood	pediatric cardiology, nutrition				
APOB	Hypobetalipoproteinemia/Familial hypercholesterolemia 2	cardiovascular	AD, AR	N	416.5	Childhood	Elevated LDL, plaque deposition in the coronary arteries and proximal aorta at an early age	Y	LDL		diet and exercise, statins (8 years)	diet medication	childhood	pediatric cardiology, nutrition				
PCSK9	Familial hypercholesterolemia 3	cardiovascular	AD	N	416.5	Childhood	Elevated LDL, plaque deposition in the coronary arteries and proximal aorta at an early age	Y	LDL		diet and exercise, statins (8 years)	diet medication	childhood	pediatric cardiology, nutrition				
LDLRAP1	Familial hypercholesterolemia 4	cardiovascular	AR	N		Childhood	Elevated LDL, plaque deposition in the coronary arteries and proximal aorta at an early age	Y	LDL		diet and exercise, statins (8 years)	diet medication	childhood	pediatric cardiology, nutrition				
LMF1	Lipase maturation factor 1 (LMF1) deficiency (familial chylomicronemia syndrome)	cardiovascular	AR	N	0.55	Late adulthood	Pancreatitis	Y	triglyeride level		dietary fat restriction, volanesorsen (most approved therapies for hypertriglyceridemia have very limited efficacy in FCS)	diet medication	childhood	pediatric cardiology, nutrition		https://pubmed.ncbi.nlm.nih.gov/31390500/	https://pubmed-ncbi-nlm-nih-gov.ezp-prod1.hul.harvard.edu/35246399/	
LMNA	Hutchinson-Gilford progeria syndrome	cardiovascular	AD	N	0.015	Infancy	Growth deficiency, characteristic face, hair, nails, sclerodermatous skin changes, joint dislocations, hearing loss, severe atherosclerosis	N			lonafarnib	medication	childhood	pediatric cardiology				
LPL	Lipoprotein lipase deficiency	cardiovascular	AR	N	0.55	Infancy	Hepatomegaly, splenomegaly, lipemia retinalis, pancreatitis, hypertriglyceridemia	Y	triglyeride level, lipoprotein enzyme level		dietary fat restriction, volanesorsen	diet medication	childhood	pediatric cardiology		https://pubmed.ncbi.nlm.nih.gov/31390500/	https://pubmed.ncbi.nlm.nih.gov/32472350/	https://pubmed.ncbi.nlm.nih.gov/20301485/
SMAD4	Myhre syndrome	cardiovascular	AD	N	3.625	Childhood	Structural heart disease, restrictive cardiomyopathy, hypertension, airway stenosis, pyloric stenosis, thickened skin, intellectual disability, characteristic facial features	N			losartan	medication	childhood	pediatric cardiology		https://pubmed.ncbi.nlm.nih.gov/33369056/		
TAFAZZIN	Barth Syndrome	cardiovascular	XLR	N	0.43	Infancy	Cardiomyopathy, neutropenia, skeletal myopathy, growth delay, characteristic facial features	Y	monolysocard-iolipin/cardio-lipin(MLCL:L4-CL) ratio testing, urine organic acids		elamipretide, cornstarch, GCSF, cardiac transplant	medication OT	infancy, childhood	pediatric cardiology, pediatric hematology		https://pubmed.ncbi.nlm.nih.gov/33077895/		
TTR	Transthyretin associated hereditary amyloidosis	cardiovascular	AD	N	1	Adulthood	Progressive neuropathy, cardiomyopathy, nephropathy, vitreous opacities, CNS amyloidosis	Y	radionucleotide scan		Patisiran, Tegsedi, Tafamidis, NTLA-2001	medication	adulthood	adult cardiology				

© 2023 Gold NB et al. *JAMA Network Open.*

Endocrinology (95 genes)

Gene	Disease name	System	Inheritance	On RUSP? (Y/N)	Prevalence - disease frequency per 100,000 (Rx genes, if listed)	Age of Onset	Disease symptoms	Orthogonal test? (Y/N)	If yes, orthogonal test	Is orthogonal test expected to be abnormal in infancy?	Intervention Considered (Free Text)	Category of Intervention	Age of Intervention Implementation	MD leading intervention	Comments	Link to ref 1	Link to ref 2	Link to ref 3
PDX1	Maturity-onset diabetes of the young, type 4	endocrinology	AD	N		Adolescence, young adulthood	Pacreatic developmental anomalies, pancreatic dysgenesis, exocrine dysfunction	Y	Fecal elastase and pancreatic enzymes (pancreatic amylase and lipase) , imaging using abdominal ultrasonography, computed tomography, or	N	Oral antidiabetic drugs (OADs), insulin	medication	Adolescence	Endocrinologist		https://www.ncbi.nlm.nih.gov/books/NBK500456/	https://pubmed.ncbi.nlm.nih.gov/28436541/	
NEUROD1	Maturity-onset diabetes of the young, type 6	endocrinology	AD, AR	N		Adolescence, young adulthood	Chronic Hyperglycemia, progression of microangiopathy, neurological abnormalities	Y	Glucose tolerance test, hemoglobin A1C, insulin level, glucose level	N	Oral antidiabetic drugs (OADs), insulin	medication	Adolescence	Endocrinologist		https://www.ncbi.nlm.nih.gov/books/NBK500456/	https://pubmed.ncbi.nlm.nih.gov/30793219//	
KLF11	Maturity-onset diabetes of the young, type 7	endocrinology	AD	N		Adolescence, young adulthood	Pancreatic anomalies, absence of islet autoimmunity	Y	Glucose tolerance test, hemoglobin A1C, insulin level, glucose level	N	Oral antidiabetic drugs (OADs), insulin	medication	Adolescence	Endocrinologist		https://www.ncbi.nlm.nih.gov/books/NBK500456/	https://pubmed.ncbi.nlm.nih.gov/32528556/	
CEL	Maturity-onset diabetes of the young, type 8	endocrinology	AD	N		Adolescence, young adulthood	Pancreatic anomalies, absence of islet autoimmunity	Y	Glucose tolerance test, hemoglobin A1C, insulin level, glucose level	N	Oral antidiabetic drugs (OADs), insulin	medication	Adolescence	Endocrinologist		https://www.ncbi.nlm.nih.gov/books/NBK500456/	https://pubmed.ncbi.nlm.nih.gov/32528556/	
PAX4	Maturity-onset diabetes of the young, type 9	endocrinology	AD	N		Adolescence, young adulthood	Pancreatic anomalies, absence of islet autoimmunity	Y	Glucose tolerance test, hemoglobin A1C, insulin level, glucose level	N	Oral antidiabetic drugs (OADs), insulin	medication	Adolescence	Endocrinologist		https://www.ncbi.nlm.nih.gov/books/NBK500456/	https://pubmed.ncbi.nlm.nih.gov/32528556/	
INS	Maturity-onset diabetes of the young, type 10	endocrinology	AD, AR	N	0.87 (per 100,000)	Adolescence, young adulthood	Pancreatic anomalies, absence of islet autoimmunity	Y	Glucose tolerance test, hemoglobin A1C, insulin level, glucose level	N	Oral antidiabetic drugs (OADs), insulin	medication	Adolescence	Endocrinologist		https://www.ncbi.nlm.nih.gov/books/NBK500456/	https://pubmed.ncbi.nlm.nih.gov/32528556/	
APPL1	Maturity-onset diabetes of the young, type 14	endocrinology	AD	N		Adolescence, young adulthood	Pancreatic anomalies, absence of islet autoimmunity	Y	Glucose tolerance test, hemoglobin A1C, insulin level, glucose level	N	Oral antidiabetic drugs (OADs), insulin	medication	Adolescence	Endocrinologist		https://www.ncbi.nlm.nih.gov/books/NBK500456/	https://pubmed.ncbi.nlm.nih.gov/32528556/	
GATA4	GATA4 associated diabetes	endocrinology	AD	N	0.87 (per 100,000) See INS per RXGenes	Neonatal, childhood	Endothelial cell dysfunction, enhanced inflammation, atherosclerosis development	Y	Fecal elastase and pancreatic enzymes (pancreatic amylase and lipase) , imaging using abdominal ultrasonography, computed tomography, or magnetic resonance imaging, glucose tolerance test, hemoglobin A1C, insulin level, glucose level		Insulin, oral pancreatic enzymes	medication	Neonatal	Endocrinologist		https://www.ncbi.nlm.nih.gov/pmc/articles/PMC6850908/	https://pubmed.ncbi.nlm.nih.gov/30809744/	
HNF1B	Renal cysts and diabetes syndrome	endocrinology	AD	N		Adolescence, young adulthood	Kidney abnormalities, early-onset diabetes, abnormal liver function, pancreatic hypoplasia, & genital tract malformations	Y	Glucose tolerance test, hemoglobin A1C, insulin level, glucose level, imaging using abdominal ultrasonography, computed tomography, or magnetic resonance imaging, uric acid level, fecal elastase		Oral antidiabetic drugs (OADs), insulin	medication	Adolescence	Endocrinologist		https://www.omim.org/entry/137920#description	https://pubmed.ncbi.nlm.nih.gov/30778115/	
SLC19A2	Thiamine-responsive megaloblastic anemia syndrome with diabetes mellitus and sensorineural deafness	endocrinology	AR	N	0.87 (per 100,000) See INS per RXGenes	Infancy, adolescence	Prompt reticulocytosis and a rise in hemoglobin concentration, latent diabetes mellitus, sensorineural deafness	Y	Glucose tolerance test, hemoglobin A1C, insulin level, glucose levels, complete blood count with MCV, B12 and folate levels, hearing test		B1 (thiamine), insulin	medication	Childhood	Endocrinologist		https://omim.org/entry/249270	https://pubmed.ncbi.nlm.nih.gov/671156/	
MNX1	MNX1 associated neonatal diabetes mellitus	endocrinology	AD	N	0.87 (per 100,000) See INS per RXGenes	Neonatal	Promotion of cancer, cervical cancer	Y	Glucose tolerance test, hemoglobin A1C, insulin level, glucose leve		Insulin	medication	Childhood	Endocrinologist		https://pubmed.ncbi.nlm.nih.gov/32850410/		
NEUROG3	NEUROG3 associated neonatal diabetes mellitus	endocrinology	AR	N	0.87 (per 100,000) See INS per RXGenes	Neonatal Infancy, childhood	Abnormalities of the intrahepatic biliary tract, thyroid gland & central nervous system	Y	Glucose tolerance test, hemoglobin A1C, insulin level, glucose level		Insulin	medication	Childhood	Endocrinologist		https://pubmed.ncbi.nlm.nih.gov/28940958/	https://pubmed.ncbi.nlm.nih.gov/28940958/	
NKX2-2	NKX2-2 associated neonatal diabetes mellitus	endocrinology	AR	N	0.87 (per 100,000) See INS per RXGenes	Neonatal, infancy	Severe NDM associated with very low birth weight, childhood obesity, and developmental delay, associated with postprandial paradoxical ghrelin secretion	Y	Glucose tolerance test, hemoglobin A1C, insulin level, glucose level		Insulin	medication	Childhood	Endocrinologist		https://pubmed.ncbi.nlm.nih.gov/32818257/		
ABCC8	Familial hyperinsulinemic hypoglycemia-1; ABCC8 associated permanent neonatal diabetes mellitus	endocrinology	AD, AR	N	2.4 ((per 100,000) See GLUD1 per RXGenes	Infancy	Presence of low plasma glucose levels, severe and persistent hypoglycaemia in neonates and children, suppressed ketone body formation	Y	Glucose, insulin, free fatty acid levels		Diazoxide, somatostatin analogs, nifedipine, glucagon, IGF-1, glucocorticoids, growth hormone, pancreatic resection, mTOR inhibitors, GLP-1 receptor antagonists, sirolimus	medication surgery	Infancy	Endocrinologist		https://www.omim.org/entry/256450?search=abcc8&highlight=abcc8#phenotypeMap	https://pubmed.ncbi.nlm.nih.gov/29280746/	
KCNJ11	Familial hyperinsulinemic hypoglycemia-2; KCNJ11 associated permanent neonatal diabetes mellitus	endocrinology	AD	N	2.4 ((per 100,000) See GLUD1 per RXGenes	Neonatal, childhood	Presence of low plasma glucose levels, severe and persistent hypoglycaemia in neonates and children, suppressed ketone body formation	Y	Glucose, insulin, free fatty acid levels		Diazoxide, somatostatin analogs, nifedipine, glucagon, IGF-1, glucocorticoids, growth hormone, pancreatic resection, mTOR inhibitors, GLP-1 receptor antagonists, sirolimus	medication surgery	Infancy	Endocrinologist		https://www.ncbi.nlm.nih.gov/books/NBK1375/	https://pubmed.ncbi.nlm.nih.gov/29280746/	
GCK	Familial hyperinsulinemic hypoglycemia 3	endocrinology	AR	N	2.4 ((per 100,000) See GLUD1 per RXGenes	Neonatal,childhood	Presence of low plasma glucose levels, severe and persistent hypoglycaemia in neonates and children,suppressed ketone body formation	Y	Glucose, insulin, free fatty acid levels		Diazoxide, somatostatin analogs, nifedipine, glucagon, IGF-1, glucocorticoids, growth hormone, pancreatic resection, mTOR inhibitors, GLP-1 receptor antagonists, sirolimus	medication surgery	Childhood	Endocrinologist		https://www.ncbi.nlm.nih.gov/books/NBK1375/	https://pubmed.ncbi.nlm.nih.gov/29280746/	
SLC16A1	Familial hyperinsulinemic hypoglycemia 7	endocrinology	AD	N	2.4 ((per 100,000) See GLUD1 per RXGenes	Neonatal, childhood	Presence of low plasma glucose levels, severe and persistent hypoglycaemia in neonates and children,suppressed ketone body formation	Y	Glucose, insulin, free fatty acid levels		Diazoxide, somatostatin analogs, nifedipine, glucagon, IGF-1, glucocorticoids, growth hormone, pancreatic resection, mTOR inhibitors, GLP-1 receptor antagonists, sirolimus	medication surgery	Childhood	Endocrinologist		https://pubmed.ncbi.nlm.nih.gov/29280746/ https://pubmed.ncbi.nlm.nih.gov/29329447/		
AKT2	Hypoinsulinemic hypoglycemia	endocrinology	AD	N		Neonatal,childhood	Presence of low plasma glucose levels, severe and persistent hypoglycaemia in neonates and children,suppressed ketone body formation	N			Sirolimus	medication	Childhood	Endocrinologist		https://pubmed.ncbi.nlm.nih.gov/29280746/		

© 2023 Gold NB et al. *JAMA Network Open.*

Endocrinology (95 genes)

Gene	Disease name	System	Inheritance	On RUSP? (Y/N)	Prevalence - disease frequency per 100,000 (Rx genes, if listed)	Age of Onset	Disease symptoms	Orthogonal test? (Y/N)	If yes, orthogonal test	Is orthogonal test expected to be abnormal in infancy?	Intervention Considered (Free Text)	Category of Intervention	Age of Intervention Implementation	MD leading intervention	Comments	Link to ref 1	Link to ref 2	Link to ref 3
FOXA2	FOXA2 associated hyperinsulinism	endocrinology	AD	N	2.4 ((per 100,000) See GLUD1 per RXGenes	Infancy,neonatal	Uncontrolled goblet cell hyperplasia & metaplasia, mucus hypersecretion, impaired mucociliary clearance of pathogens, infection of pulmonary functions	Y	Glucose, insulin, free fatty acid levels, TSH		Diazoxide, somatostatin analogs, nifedipine, glucagon, IGF-1, glucocorticoids, growth hormone, pancreatic resection, mTOR inhibitors, GLP-1 receptor antagonists, sirolimus, levothyroxine	medication surgery	Childhood	Endocrinologist		https://pubmed.ncbi.nlm.nih.gov/32269574/	https://pubmed.ncbi.nlm.nih.gov/32269574/	
HK1	HK1 associated hyperinsulinism	endocrinology	AD	N	2.4 ((per 100,000) See GLUD1 per RXGenes	Infancy, neonatal	Birth asphyxia, small for gestational age birthweight, infant of diabetic mother Beckwith-Wiedemann, Kabuki, & Turner syndromes	Y	Glucose, insulin, free fatty acid levels		Diazoxide, somatostatin analogs, nifedipine, glucagon, IGF-1, glucocorticoids, growth hormone, pancreatic resection, mTOR inhibitors, GLP-1 receptor antagonists, sirolimus	medication surgery	Childhood	Endocrinologist		https://pubmed.ncbi.nlm.nih.gov/26908106/		
HNF1A	HNF1A associated hyperinsulinism	endocrinology	AD	N	2.4 ((per 100,000) See GLUD1 per RXGenes	Infancy, childhood	Birth asphyxia, small for gestational age birthweight, infant of diabetic mother Beckwith-Wiedemann, Kabuki, & Turner syndromes	Y	Glucose, insulin, free fatty acid levels		Diazoxide, somatostatin analogs, nifedipine, glucagon, IGF-1, glucocorticoids, growth hormone, pancreatic resection, mTOR inhibitors, GLP-1 receptor antagonists, sirolimus	medication surgery	Childhood	Endocrinologist		https://medlineplus.gov/genetics/condition/congenital-hyperinsulinism/	https://pubmed.ncbi.nlm.nih.gov/26908106/	
HNF4A	HNF4A associated hyperinsulinism	endocrinology	AD	N	2.4 ((per 100,000) See GLUD1 per RXGenes	Infancy, childhood	Birth asphyxia, small for gestational age birthweight, infant of diabetic mother Beckwit& Turner syndromesh-Wiedemann, Kabuki,	Y	Glucose, insulin, free fatty acid levels		Diazoxide, somatostatin analogs, nifedipine, glucagon, IGF-1, glucocorticoids, growth hormone, pancreatic resection, mTOR inhibitors, GLP-1 receptor antagonists, sirolimus	medication surgery	Childhood	Endocrinologist				
UCP2	UCP2 associated hyperinsulinism	endocrinology	AD	N	2.4 ((per 100,000) See GLUD1 per RXGenes	Infancy, childhood		Y	Glucose, insulin, free fatty acid levels		Diazoxide, somatostatin analogs, nifedipine, glucagon, IGF-1, glucocorticoids, growth hormone, pancreatic resection, mTOR inhibitors, GLP-1 receptor antagonists, sirolimus	medication surgery	Childhood	Endocrinologist				
EIF2AK3	Wolcott-Rallison syndrome	endocrinology	AR	N	0.87 (per 100,000) See INS per RXGenes	Neonatal, early-onset childhood	Neonatal/early-onset non-autoimmune insulin -requiring diabetes associated with skeletal dysplasia and growth retardation	Y	Glucose tolerance test, hemoglobin A1C, insulin level, glucose level, fecal elastase, complete blood count, TSH level		Insulin, oral pancreatic enzymes, levothyroxine	medication	Childhood	Endocrinologist				
RFX6	Mitchell-Riley syndrome	endocrinology	AR	N	0.87 (per 100,000) See INS per RXGenes	Neonatal, infancy	Neonatal diabetes, intestinal atresia, progressive cholestasis	Y	Glucose tolerance test, hemoglobin A1C, insulin level, glucose level		Insulin	medication	Infancy	Endocrinologist				
GATA6	Pancreatic agenesis and congenital heart defects	endocrinology	AD	N	0.87 (per 100,000) See INS per RXGenes	Neonatal	Triggers a junctional zone and sebaceous differentiation program whilst limiting lipid production and cell proliferation	Y	Fecal elastase and pancreatic enzymes (pancreatic amylase and lipase) , imaging using abdominal ultrasonography, computed tomography, or magnetic resonance imaging		Insulin, oral pancreatic enzymes	medication	Infancy	Endocrinologist		https://www.ncbi.nlm.nih.gov/pmc/articles/PMC4338276/	https://pubmed.ncbi.nlm.nih.gov/33082341/	
PTF1A	Pancreatic agenesis 2	endocrinology	AR	N	0.87 (per 100,000) See INS per RXGenes	Neonatal	Neonatal diabetes and impaired food digestion due to exocrine pancreatic insufficiency	Y	Fecal elastase and pancreatic enzymes (pancreatic amylase and lipase) , imaging using abdominal ultrasonography, computed tomography, or magnetic resonance imaging		Insulin, oral pancreatic enzymes	medication	Infancy	Endocrinologist		https://www.ncbi.nlm.nih.gov/pmc/articles/PMC4338276/	https://pubmed.ncbi.nlm.nih.gov/34125483/	
AQP2	Nephrogenic diabetes insipidus	endocrinology	AD, AR	N	0.87 (per 100,000) See INS per RXGenes	Neonatal, infancy	Growth retardation, vomiting or feeding concerns, polyuria plus polydipsia, and dehydration, constipation, urological complication, megacystis, trabeculated bladder, hydroureter, hydronephrosis, and mental retardation	Y	Fecal elastase and pancreatic enzymes (pancreatic amylase and lipase) , imaging using abdominal ultrasonography, computed tomography, or magnetic resonance imaging		Insulin, oral pancreatic enzymes	medication	Infancy	Endocrinologist		https://www.orpha.net/consor/cgi-bin/OC_Exp.php?Expert=223&lng=EN	https://pubmed.ncbi.nlm.nih.gov/34055061/	
AVPR2	X-linked nephrogenic diabetes insipidus	endocrinology	XLR	N	0.64	Infancy, early childhood	Growth retardation, vomiting or feeding concerns, polyuria plus polydipsia, and dehydration, constipation, urological complication, megacystis, trabeculated bladder, hydroureter, hydronephrosis, and mental retardation	Y	Serum elecrolytes, urine osmolality		Adequate hydration, low-salt, low-protein diet, thiazide diuretics, amiloride, indomethacin	diet medication	Childhood	Endocrinologist		https://www.ncbi.nlm.nih.gov/pmc/articles/PMC6280332/	https://pubmed.ncbi.nlm.nih.gov/34055061/	
AVP	Neurohypophyseal diabetes insipidus	endocrinology	AD	N	0.64	Neonatal,infancy	Germinoma/craniopharyngioma, Langerhans cell histiocytosis (LCH), local inflammatory, autoimmune or vascular diseases, trauma resulting from surgery or an accident, sarcoidosis, metastases and midline cerebral and cranial malformations, genetic defects in vasopressin synthesis	Y	Water deprivation test and desmopressin (DDAVP) trial		Adequate hydration, low-salt, low-protein diet, thiazide diuretics, amiloride, indomethacin	diet medication		Endocrinologist		https://pubmed.ncbi.nlm.nih.gov/19897608/	https://pubmed.ncbi.nlm.nih.gov/22433947/	
AGPAT2	Congenital generalized lipodystrophy type 1	endocrinology	AR	N	0.023	Early onset childhood to adulthood	Insulin resistance, hypertriglyceridemia, hepatic steatosis (metabolic complications)	Y	Leptin level		Metreleptin	medication		Endocrinologist		https://rarediseases.org/rare-diseases/congenital-generalized-lipodystrophy/	https://pubmed.ncbi.nlm.nih.gov/27823605/	
BSCL2	Congenital generalized lipodystrophy type 2	endocrinology	AR	N	0.023	Early onset childhood to adulthood	Insulin resistance, hypertriglyceridemia, hepatic steatosis (metabolic complications)	Y	Leptin level		Metreleptin	medication		Endocrinologist		https://rarediseases.org/rare-diseases/congenital-generalized-lipodystrophy/	https://pubmed.ncbi.nlm.nih.gov/27823605/	
CAV1	Congenital generalized lipodystrophy type 3	endocrinology	AR	N	0.023	Early onset childhood to adulthood	Insulin resistance, hypertriglyceridemia, hepatic steatosis (metabolic complications)	Y	Leptin level		Metreleptin	medication	Childhood	Endocrinologist		https://rarediseases.org/rare-diseases/congenital-generalized-lipodystrophy/	https://pubmed.ncbi.nlm.nih.gov/27823605/	
CAVIN1	Congenital generalized lipodystrophy type 4	endocrinology	AR	N	0.023	Early onset childhood to adulthood	Insulin resistance, hypertriglyceridemia, hepatic steatosis (metabolic complications)	Y	Leptin level		Metreleptin	medication	Childhood	Endocrinologist		https://rarediseases.org/rare-diseases/congenital-generalized-lipodystrophy/	https://pubmed.ncbi.nlm.nih.gov/27823605/	

© 2023 Gold NB et al. *JAMA Network Open.*

Endocrinology (95 genes)

Gene	Disease name	System	Inheritance	On RUSP? (Y/N)	Prevalence - disease frequency per 100,000 (Rx genes, if listed)	Age of Onset	Disease symptoms	Orthogonal test? (Y/N)	If yes, orthogonal test	Is orthogonal test expected to be abnormal in infancy?	Intervention Considered (Free Text)	Category of Intervention	Age of Intervention Implementation	MD leading intervention	Comments	Link to ref 1	Link to ref 2	Link to ref 3
ABCC9	ABCC9 associated hypertrichotic osteochondrodysplasia	endocrinology	AD	N		Neonatal	Cantu syndrome, congenital hypertrichosis, osteochondrodysplasia, extensive cardiovascular abnormalities and distinctive facial anomalies including a broad nasal bridge, long philtrum, epicanthal folds, & prominent lips	N			Glibenclamide	medication	Infancy,childhood	Endocrinologist		https://www.orpha.net/consor/cgi-bin/OC_Exp.php?lng=EN&Expert=1517	https://pubmed.ncbi.nlm.nih.gov/32100467/	
KCNJ8	KCNJ8 associated hypertrichotic osteochondrodysplasia	endocrinology	AD	N		Neonatal	Cantu syndrome, congenital hypertrichosis, osteochondrodysplasia, extensive cardiovascular abnormalities and distinctive facial anomalies including a broad nasal bridge, long philtrum, epicanthal folds, & prominent lips	N			Glibenclamide	medication	Infancy,childhood	Endocrinologist		https://www.orpha.net/consor/cgi-bin/OC_Exp.php?lng=EN&Expert=1517	https://pubmed.ncbi.nlm.nih.gov/32100467/	
CA2	Osteopetrosis with renal tubular acidosis	endocrinology	AD	N	0.4 (See CLCN7 per RxGenes)	Infancy, neonatal	Cerebral calcification, renal tubular acidosis (often combined proximal and distal),mental retardation, growth failure,complications of osteopetrosis	Y	Skeletal survey, serum potassium, bicarbonate and anion gap, urinary pH		Sodium bicarbonate, potassium citrate, calcium and vitamin D	medication	Infancy	Endocrinologist		https://www.orpha.net/consor/cgi-bin/OC_Exp.php?lng=en&Expert=2785	https://pubmed.ncbi.nlm.nih.gov/23640632/	
TCIRG1	Osteopetrosis type 1	endocrinology	AR	N		Neonatal,infancy	Osteoclast-rich ARO, inability to resorb bone and mineralized cartilage	Y	Skeletal survey	Y	Bone marrow transplant (hematopoietic stem cell transplantation (HSCT))	HSCT	Infancy	Endocrinologist		https://pubmed.ncbi.nlm.nih.gov/23877423/		
TNFRSF11A	Osteopetrosis type 7	endocrinology	AR	N		Neonatal,infancy	Paget's disease of bone, familial expansile osteolysis, and expansile skeletal hyperphosphatasia, dysosteosclerosis	Y	Skeletal survey	Y	Bone marrow transplant (hematopoietic stem cell transplantation (HSCT))	HSCT	Infancy	Endocrinologist		https://pubmed.ncbi.nlm.nih.gov/23877423/		
SNX10	Osteopetrosis type 8	endocrinology	AR	N	0.4 (See CLCN7 per RxGenes)	Neonatal, infancy	Osteoclast-rich ARO, inability to resorb bone and mineralized cartilage	Y	Skeletal survey	Maybe	Bone marrow transplant (hematopoietic stem cell transplantation (HSCT))	HSCT	Infancy	Endocrinologist		https://pubmed.ncbi.nlm.nih.gov/23877423/		
FAM111A	Kenny-Caffey syndrome, type 2	endocrinology	AD	N		Neonatal	Hypoparathyroidism,cortical thickening, medullary stenosis of tubular long bones, delayed closure of anterior fontanel and eye abnormalities,short stature	Y	Serum calcium, parathyroid hormone level, calcitonin level		Magnesium, calcium, calcitriol or alfacalcidol	medication	Infancy	Endocrinologist		https://rarediseases.org/rare-diseases/kenny-caffey-syndrome/	https://pubmed.ncbi.nlm.nih.gov/32428224/	
CYP11B2	Aldosterone synthase deficiency	endocrinology	AR	N		Neonatal, infancy	Hypotension, hyponatremia, hyperkalemia, acidosis, primary adrenal insufficiency & congenital adrenal hypoplasia	Y	Sodium, potassium, aldosterone, renin levels		Fludrocortisone	medication	Infancy	Endocrinologist		https://www.ncbi.nlm.nih.gov/pmc/articles/PMC8272273/	https://pubmed.ncbi.nlm.nih.gov/25905305/	
CACNA1D	Primary aldosteronism with seizures and neurologic abnormalities	endocrinology	AD	N		Infancy,neonatal	Seizures, neurologic abnormalities, global developmental delay with primary hyperaldosteronism	Y	Blood pressure measurement and aldosterone, renin, potassium levels		Calcium channel blocker, clonidine, spironolactone	medication	Infancy	Endocrinologist		https://www.orpha.net/consor/cgi-bin/OC_Exp.php?lng=en&Expert=369929	https://pubmed.ncbi.nlm.nih.gov/30698561/	
CLCN2	Familial hyperaldosteronism, Type II	endocrinology	AD	N		Adulthood	Early-onset hypertension, severe target organ damage, secondary hypertension	Y	Blood pressure measurement and potassium, aldosterone, renin levels		Antihypertensive medication	medication	Adulthood	Endocrinologist		https://www.orpha.net/consor/cgi-bin/Disease_Search.php?lng=EN&data_id=8738&Disease_Disease_Search_diseaseGroup=Familial-hyperaldosteronism&Disease_Disease_Search_diseaseType=Pat&Disease(s)/group%20of%20diseases=Familial-hyperaldosteronism-type-II&title=Familial%20hyperaldosteronism%20type%20II&search=Disease_Search_Simple	https://pubmed.ncbi.nlm.nih.gov/20131203/	
KCNJ5	Familial hyperaldosteronism, Type III	endocrinology	AD	N		Infancy to adolescence	Early-onset hypertension, severe target organ damage, secondary hypertension	Y	Blood pressure measurement and aldosterone, 18-hydroxycortisol, 18-oxocortisol, potassium, renin levels		Bilateral adrenalectomy	surgery	Childhood	Endocrinologist		https://www.orpha.net/consor/cgi-bin/Disease_Search.php?lng=EN&data_id=19618&Disease_Disease_Search_diseaseGroup=Familial-hyperaldosteronism&Disease_Disease_Search_diseaseType=Pat&Disease(s)/group%20of%20diseases=Familial-hyperaldosteronism-type-III&title=Familial%20hyperaldosteronism%20type%20III&search=Disease_Search_Simple	https://pubmed.ncbi.nlm.nih.gov/20131203/	
CACNA1H	Familial hyperaldosteronism, Type IV	endocrinology	AD	N		Hypertension by age 10 years	Early-onset hypertension, severe target organ damage, secondary hypertension	Y	Blood pressure measurement and potassium, aldosterone, renin levels		Calcium channel blocker	medication		Endocrinologist		https://www.omim.org/entry/617027		
WNK4	Pseudohypoaldosteronism, type IIB	endocrinology	AD	N		Childhood to young adulthood	Overt dehydration, hyponatremia, insufficient weight gain, resistance of kidney & other tissues to mineralocorticoids,highly variable plasma aldosterone concentrations, suppressed plasma renin activity, various degrees of hyperchloremia and metabolic acidosis	Y	Serum potassium, chloride and anion gap and blood pressure measurement		Thiazide diuretics	medication	Childhood	Endocrinologist		https://www.ncbi.nlm.nih.gov/books/NBK65707/	https://pubmed.ncbi.nlm.nih.gov/23392097/	
WNK1	Pseudohypoaldosteronism, type IIC	endocrinology	AD	N		Childhood to young adulthood	Overt dehydration, hyponatremia, insufficient weight gain, resistance of kidney & other tissues to mineralocorticoids,highly variable plasma aldosterone concentrations, suppressed plasma renin activity, various degrees of hyperchloremia and metabolic acidosis	Y	Serum potassium, chloride and anion gap and blood pressure measurement		Thiazide diuretics	medication	Childhood	Endocrinologist		https://www.ncbi.nlm.nih.gov/books/NBK65707/		
KLHL3	Pseudohypoaldosteronism, type IID	endocrinology	AD, AR	N		Childhood to young adulthood	Overt dehydration, hyponatremia, insufficient weight gain, resistance of kidney & other tissues to mineralocorticoids,highly variable plasma aldosterone concentrations, suppressed plasma renin activity, various degrees of hyperchloremia and metabolic acidosis	Y	Serum potassium, chloride and anion gap and blood pressure measurement		Thiazide diuretics	medication	Childhood	Endocrinologist		https://www.ncbi.nlm.nih.gov/books/NBK65707/		
CUL3	Pseudohypoaldosteronism, type IIE	endocrinology	AD	N		Childhood to young adulthood	Overt dehydration, hyponatremia, insufficient weight gain, resistance of kidney & other tissues to mineralocorticoids,highly variable plasma aldosterone concentrations, suppressed plasma renin activity, various degrees of hyperchloremia and metabolic acidosis	Y	Serum potassium, chloride and anion gap and blood pressure measurement		Thiazide diuretics	medication	Childhood	Endocrinologist		https://www.ncbi.nlm.nih.gov/books/NBK65707/		

© 2023 Gold NB et al. *JAMA Network Open.*

Endocrinology (95 genes)

Gene	Disease name	System	Inheritance	On RUSP? (Y/N)	Prevalence - disease frequency per 100,000 (Rx genes, if listed)	Age of Onset	Disease symptoms	Orthogonal test? (Y/N)	If yes, orthogonal test	Is orthogonal test expected to be abnormal in infancy?	Intervention Considered (Free Text)	Category of Intervention	Age of Intervention Implementation	MD leading intervention	Comments	Link to ref 1	Link to ref 2	Link to ref 3
NR3C2	NR3C2 associated pseudohypoaldosteronism, type I	endocrinology	AD	N		Neonatal, infancy	Overt dehydration, hyponatremia, insufficient weight gain, resistance of kidney & other tissues to mineralocorticoids,highly variable plasma aldosterone concentrations, suppressed plasma renin activity, various degrees of hyperchloremia and metabolic acidosis	Y	Blood pressure measurement and sodium, potassium, aldosterone, renin levels		Sodium chloride (NaCl) replacement	medication	Infancy	Endocrinologist		https://pubmed.ncbi.nlm.nih.gov/23392097/		
SCNN1A	SCNN1A associated pseudohypoaldosteronism, type I	endocrinology	AR	N	3.595	Neonatal, infancy	Overt dehydration, hyponatremia, insufficient weight gain, resistance of kidney & other tissues to mineralocorticoids,highly variable plasma aldosterone concentrations, suppressed plasma renin activity, various degrees of hyperchloremia and metabolic acidosis	Y	Blood pressure measurement and sodium, potassium, aldosterone, renin levels		Sodium chloride (NaCl) replacement	medication	Infancy	Endocrinologist		https://pubmed.ncbi.nlm.nih.gov/23392097/		
SCNN1B	SCNN1B associated pseudohypoaldosteronism, type I	endocrinology	AR	N	3.595	Neonatal, infancy	Overt dehydration, hyponatremia, insufficient weight gain, resistance of kidney & other tissues to mineralocorticoids,highly variable plasma aldosterone concentrations, suppressed plasma renin activity, various degrees of hyperchloremia and metabolic acidosis	Y	Blood pressure measurement and sodium, potassium, aldosterone, renin levels		Sodium chloride (NaCl) replacement	medication	Infancy	Endocrinologist		https://pubmed.ncbi.nlm.nih.gov/23392097/		
SCNN1G	SCNN1G associated pseudohypoaldosteronism, type I	endocrinology	AR	N	3.595	Neonatal, infancy	Overt dehydration, hyponatremia, insufficient weight gain, resistance of kidney & other tissues to mineralocorticoids,highly variable plasma aldosterone concentrations, suppressed plasma renin activity, various degrees of hyperchloremia and metabolic acidosis	Y	Blood pressure measurement and sodium, potassium, aldosterone, renin levels		Sodium chloride (NaCl) replacement	medication	Infancy	Endocrinologist		https://pubmed.ncbi.nlm.nih.gov/23392097/		
CYP11A1	Adrenal insufficiency, congenital, with 46XY sex reversal, partial or complete	endocrinology	AR	N		Early childhood, infancy	Adrenal insufficiency, variable degrees of disorder of sex development (DSD), hyperpigmentation, failure of cortisol to respond to short synacthen test, penoscrotal penoscrotal hypospadias,hypospadias, & raised ACTH	Y	Serum cortisol, aldosterone and adrenocorticotropic hormone (ACTH) levels	Sometimes	Hydrocortisone, fludrohydrocortisone	medication	Infancy, childhood	Endocrinologist		https://www.ncbi.nlm.nih.gov/pmc/articles/PMC3494866/		
POMC	Obesity, adrenal insufficiency, and red hair due to POMC deficiency	endocrinology	AR	N		Infancy, Early onset childhood	Adrenal insufficiency, obesity and red hair	Y	Serum cortisol and adrenocorticotropic hormone (ACTH) levels	Y	Hydrocortisone, setmelanotide	medication	Infancy,childhood	Endocrinologist		https://rarediseases.info.nih.gov/diseases/10823/proopiomelanocortin-deficiency	https://pubmed.ncbi.nlm.nih.gov/21860632/	
CYP17A1	17-alpha-hydroxylase/17,20-lyase deficiency	endocrinology	AR	N		Infancy to adolescence	Low blood levels of estrogens, androgens and cortisol, increase in adrenocorticotropic hormone levels, hypertension, hypokalemia, primary amenorrhea and sexual infantilism	Y	Blood pressure measurement, serum potassium, cortisol and adrenocorticotropic hormone (ACTH) levels	Sometimes	Spironolactone, hydrocortisone	medication	Infancy, childhood	Endocrinologist		https://www.ncbi.nlm.nih.gov/pmc/articles/PMC5355729/		
CYP11B1	Congenital adrenal hyperplasia due to 11-beta-hydroxylase deficiency	endocrinology	AR	N	0.75	Infancy, neonatal	Ambiguous external genitalia with normal internal reproductive organs (in females), early development of their secondary sexual characteristics (precocious puberty), early growth spurt, short stature in adulthood, high blood pressure, excessive body hair growth & irregular menstruation	Y	Serum 11-deoxycortisol and 11-deoxycorticosterone levels	Sometimes	Hydrocortisone	medication	Infancy	Endocrinologist		https://rarediseases.info.nih.gov/diseases/5658/11-beta-hydroxylase-deficiency		
HSD3B2	Adrenal hyperplasia, congenital, due to 3-beta-hydroxysteroid dehydrogenase 2 deficiency	endocrinology	AR	N		Infancy, neonatal	Severe salt-wasting to the non-salt-wasting forms, premature pubarche, ambigious genitalia, dehydration, poor feeding, vomiting, infertility	Y	Serum cortisol, aldosterone and adrenocorticotropic hormone (ACTH) levels	Y	Hydrocortisone, 9_-fludrohydrocortisone, oral supplements of sodium chloride	medication	Infancy	Endocrinologist		https://pubmed.ncbi.nlm.nih.gov/15585552/	https://rarediseases.info.nih.gov/diseases/9152/3-beta-hydroxysteroid-dehydrogenase-deficiency	
STAR	Lipoid adrenal hyperplasia	endocrinology	AR	N		Infancy, neonatal	Severe adrenal failure in early infancy, adrenal insufficiency, salt wasting, develop female external genitalia in both human karyotypes	Y	Serum cortisol, aldosterone and adrenocorticotropic hormone (ACTH) levels	Y	Hydrocortisone, 9_-fludrohydrocortisone	medication	Infancy	Endocrinologist		https://www.ncbi.nlm.nih.gov/pmc/articles/PMC3415193/	https://pubmed.ncbi.nlm.nih.gov/25654062/	
NR5A1	NR5A1 associated adrenocortical insufficiency	endocrinology	AD	N		Infancy, neonatal	Detoriation of organ development, anomalies of adrenal or testis development, ovarian insufficiency, 46, XY disorders of sex development (DSD), hypospadias, anorchia, male factor infertility	Y	Serum LH, FSH, testosterone, inhibin, cortisol and adrenocorticotropic hormone (ACTH) levels	Sometimes	Hydrocortisone	medication	Infancy	Endocrinologist		https://www.ncbi.nlm.nih.gov/pmc/articles/PMC3057017/	https://pubmed.ncbi.nlm.nih.gov/19849982/	
SAMD9	MIRAGE syndrome	endocrinology	AD	N		Infancy, neonatal	Myelodysplasia, infection, restriction of growth, adrenal hypoplasia, genital phenotypes, and enteropathy	N			Hematopoietic stem cell transplantation (HSCT) - bone marrow transplant	HSCT	Infancy	Endocrinologist				
MC2R	Glucocorticoid deficiency due to ACTH unresponsiveness	endocrinology	AR	N		Infancy, early onset childhood	Hyperpigmentation, recurrent hypoglycemia, chronic asthenia and failure to thrive within the first 2 years of life. Typically, they have deficient production of cortisol and adrenal androgens in the presence of markedly elevated ACTH levels, while renin and aldosterone levels are usually normal and responsive to activation of the renin-angiotensin axis	Y	Serum cortisol and adrenocorticotropic hormone (ACTH) levels		Hydrocortisone	medication	Infancy, childhood	Endocrinologist				
MRAP	Glucocorticoid deficiency 2	endocrinology	AR	N		Infancy, early onset childhood	Hyperpigmentation, recurrent hypoglycemia, chronic asthenia and failure to thrive within the first 2 years of life. Typically, they have deficient production of cortisol and adrenal androgens in the presence of markedly elevated ACTH levels, while renin and aldosterone levels are usually normal and responsive to activation of the renin-angiotensin axis	Y	Serum cortisol and adrenocorticotropic hormone (ACTH) levels		Hydrocortisone	medication	Infancy, childhood	Endocrinologist		https://rarediseases.info.nih.gov/diseases/2498/familial-glucocorticoid-deficiency		
NNT	Glucocorticoid deficiency 4, with or without mineralocorticoid deficiency	endocrinology	AR	N		Infancy, early onset childhood	Hyperpigmentation, recurrent hypoglycemia, chronic asthenia and failure to thrive within the first 2 years of life. Typically, they have deficient production of cortisol and adrenal androgens in the presence of markedly elevated ACTH levels, while renin and aldosterone levels are usually normal and responsive to activation of the renin-angiotensin axis	Y	Serum cortisol and adrenocorticotropic hormone (ACTH) levels		Hydrocortisone	medication	Infancy, childhood	Endocrinologist		https://rarediseases.info.nih.gov/diseases/2498/familial-glucocorticoid-deficiency		
HSD11B2	Apparent mineralocorticoid excess		AR	N		Infancy, neonatal	Insulin resistance, hypertriglyceridemia, hepatic steatosis (metabolic complications)		Blood pressure measurement and aldosterone, renin, potassium levels		Amiloride, triamterene, spironolactone, eplerenone, dexamethasone	medication	Infancy	Endocrinologist				
TBX19	Adrenocorticotropic hormone deficiency	endocrinology	AR	N		Neonatal	Thyroiditis, hypophysitis,immune checkpoint inhibitors, such as nivolumab, pembrolizumab, and atezolizumab	Y	Serum cortisol and adrenocorticotropic hormone (ACTH) levels		Hydrocortisone	medication	Infancy	Endocrinologist		https://rarediseases.info.nih.gov/diseases/5727/isolated-acth-deficiency		

© 2023 Gold NB et al. *JAMA Network Open.*

Endocrinology (95 genes)

Gene	Disease name	System	Inheritance	On RUSP? (Y/N)	Prevalence - disease frequency per 100,000 (Rx genes, if listed)	Age of Onset	Disease symptoms	Orthogonal test? (Y/N)	If yes, orthogonal test	Is orthogonal test expected to be abnormal in infancy?	Intervention Considered (Free Text)	Category of Intervention	Age of Intervention Implementation	MD leading intervention	Comments	Link to ref 1	Link to ref 2	Link to ref 3
CYP27B1	Vitamin D-dependent rickets, type IA	endocrinology	AR	N	0.3	Infancy, early onset childhood	Hypotonia, irritability, tetany or seizures, and failure to thrive, typical skeletal features of rickets (e.g., frontal bossing, long-bone deformities, and rib cage abnormalities) as well as impaired growth,hypocalcemia, hypophosphatemia, and elevated serum levels of alkaline phosphatase and parathyroid hormone	Y	Serum calcium, parathyroid hormone, 1,25-dihydroxy vitamin D levels	N	Vitamin D as calcitriol	medication	Infancy, childhood	Endocrinologist		https://www.ncbi.nlm.nih.gov/pmc/articles/PMC7303887/		
CYP2R1	Vitamin D-dependent rickets, type IB	endocrinology	AR	N		Infancy,early childhood	Rickets, ovarian cancer, and multiple sclerosis	Y	Serum calcium, parathyroid hormone, 25-hydroxy vitamin D levels	N	Calcifediol (25_OH_D3)	medication	Infancy,childhood	Endocrinologist				
VDR	Vitamin D-dependent rickets, type 2A	endocrinology	AR	N		Infancy, adolescence	Early onset of severe rickets and associated alopecia	Y	Serum calcium, parathyroid hormone, phosphate, 1,25-dihydroxy vitamin D levels	N	Calcitriol, oral calcium, intravenous calcium, cinacalcet	medication	Infancy. childhood	Endocrinologist		https://pubmed.ncbi.nlm.nih.gov/18711299/		
PHEX	X-linked dominant hypophosphatemic rickets	endocrinology	XLD	N	5	Infancy, Childhood	Rickets and osteomalacia, severe deformities of the lower limbs, bone and muscular pain, stunted growth, & reduced quality of life	Y	Serum phosphate concentration, tubular resorption of phosphate corrected for glomerular filtration rate	N	Burosumab	medication	Infancy, childhood	Endocrinologist		https://rarediseases.info.nih.gov/diseases/12943/x-linked-hypophosphatemia		
SLC34A3	Hypophosphatemic rickets with hypercalciuria	endocrinology	AR	N		Early childhood	Development of urinary phosphate (Pi) wasting and hypophosphatemic rickets, bowing, and short stature, as well as appropriately elevated 1,25 (OH)2D levels	N		N	Phosphate supplementation	medication	Childhood	Endocrinologist		https://www.omim.org/entry/241530		
ALPL	Hypophosphatasia	endocrinology	AR	N	1	Early childhood	Severely impaired bone mineralization, seizures, and hypercalcemia, to young adults with premature exfoliation of their teeth without any other symptom, low ALP levels	Y	Alkaline phosphatase	Y (if severe type)	Tissue-nonspecific alkaline phosphatase (TNSALP) enzyme replacement therapy - asfotase alfa, avoid bisphosphonates	ERT	Childhood	Endocrinologist		https://www.ncbi.nlm.nih.gov/pmc/articles/PMC5726215/		
GH1	Isolated growth hormone deficiency type 1A, type 1B, type 2	endocrinology	AD, AR	N		Infancy to mid-childhood	Short stature, delayed growth velocity, and delayed skeletal maturation	Y	Growth hormone stimulation test		Growth hormone	medication	Infancy, childhood	Endocrinologist		https://medlineplus.gov/genetics/condition/isolated-growth-hormone-deficiency/		
GHRHR	Isolated growth hormone deficiency type 4	endocrinology	AR	N		Early childhood	Short stature, delayed growth velocity, and delayed skeletal maturation	Y	Growth hormone stimulation test		Growth hormone	medication	Childhood	Endocrinologist		https://medlineplus.gov/genetics/condition/isolated-growth-hormone-deficiency/		
RNPC3	RNPC3 associated growth hormone deficiency	endocrinology	AR	N		Mid-childhood - late childhood	Severe isolated growth hormone deficiency and pituitary hypoplasia, severe postnatal growth retardation, developmental delay	Y	Growth hormone stimulation test, insulin-like growth factor, IGF binding protein-3 levels		Growth hormone	medication	Childhood	Endocrinologist				
GHR	Growth hormone receptor deficiency	endocrinology	AR	N		Infancy, neonatal	Idiopathic short stature (ISS)	Y	Growth hormone stimulation test	Probably	Growth hormone	medication	Infancy	Endocrinologist		https://www.orpha.net/consor/cgi-bin/Disease_Search.php?lng=EN&data_id=3250&Disease_Disease_Search_diseaseType=ORPHA&Disease_Disease_Search_diseaseGroup=633&Disease(s)/group%20of%20diseases=Growth-hormone-receptor-deficiency&title=Growth-hormone-receptor-deficiency&search=Disease_Search_Simple		
IGF1	Insulin-like growth factor I deficiency	endocrinology	AR	N		Infancy, neonatal	Growth failure, dysmorphic and metabolic abnormalities	Y	IGF-1 level and growth hormone level	Probably	Recombinant human IGF-1	medication	Infancy	Endocrinologist		https://rarediseases.info.nih.gov/diseases/10627/insulin-like-growth-factor-i-deficiency		
GPR101	Growth hormone-secreting pituitary adenoma 2	endocrinology	XLD	N		Infancy to adulthood	High expressionin the arcuate nucleus & the occurrence of increased circulating GHRH levels in some patients with XLAG, increased hypothalamic GHRH secretion, gigantism	Y	Growth hormone, prolactin, IGF-1 levels		Transsphenoidal surgery, GH receptor antagonist	medication surgery	Infancy	Endocrinologist		https://www.orpha.net/consor/cgi-bin/Disease_Search.php?lng=EN&data_id=408&Disease_Disease_Search_diseaseGroup=gpr101&Disease_Disease_Search_diseaseType=Gen&Disease(s)/group%20of%20diseases=Acromegaly&title=Acromegaly&search=Disease_Search_Simple		
IGFALS	Acid-labile subunit deficiency	endocrinology	AR	N		Infancy, neonatal	Severely reduced serum IGF-I and IGFBP-3 concentrations that is incongruent with the associated mild growth retardation, insulin insensitivity	Y	Acid labile subunit level, IGF-1 and IGFBP-3		Growth hormone	medication	Infancy	Endocrinologist		https://www.orpha.net/consor/cgi-bin/OC_Exp.php?lng=en&Expert=140941		
PAPPA2	PAPPA2 associated short stature	endocrinology	AR	N		Early/late childhood	Short stature, Laron syndrome	Y	Free IGF1, PAPPA2, IGF1, IGFBP3, IGFALS, stimulated GH levels		Recombinant human IGF-1	medication	Childhood, adulthood	Endocrinologist		https://www.ncbi.nlm.nih.gov/pmc/articles/PMC4818753/		
POU1F1	Combined pituitary hormone deficiency 1	endocrinology	AD, AR	N	2.31	Early childhood onset to adulthoood	Organic etiology, H-P abnormalities (in particular pituitary stalk abnormalities, empty sella and ectopic posterior pituitary), midline brain (corpus callosum) and optic nerves abnormalities, genetic defects and longer duration of follow-up	Y	Growth hormone, thyroid-stimulating hormone, prolactin levels	Probably not	Growth hormone, levothyroxine	medication	Childhood, adulthood	Endocrinologist				
PROP1	Combined pituitary hormone deficiency 2	endocrinology	AR	N	2.31	Early childhood onset to adulthoood	Deficiencies of: growth hormone (GH); thyroid-stimulating hormone (TSH); the two gonadotropins, luteinizing hormone (LH) & follicle-stimulating hormone (FSH); prolactin (PrL)	Y	Growth hormone, thyroid-stimulating hormone, luteinizing hormone, follicle-stimulating hormone, prolactin, adrenocorticotropic hormone levels	N	Growth hormone, levothyroxine, hydrocortisone, testosterone for males, estrogen for females	medication	Childhood, adulthood	Endocrinologist				
LHX3	Combined pituitary hormone deficiency 3	endocrinology	AR	N	2.31	Early childhood onset to adulthoood	Organic etiology, H-P abnormalities (in particular pituitary stalk abnormalities, empty sella and ectopic posterior pituitary), midline brain (corpus callosum) and optic nerves abnormalities, genetic defects and longer duration of follow-up	Y	Growth hormone, thyroid-stimulating hormone, luteinizing hormone, follicle-stimulating hormone, prolactin levels	Probably	Growth hormone, levothyroxine, hydrocortisone	medication	Childhood, adulthood	Endocrinologist				
LHX4	Combined pituitary hormone deficiency 4	endocrinology	AD	N	2.31	Early childhood onset to adulthoood	Organic etiology, H-P abnormalities (in particular pituitary stalk abnormalities, empty sella and ectopic posterior pituitary), midline brain (corpus callosum) and optic nerves	Y	Growth hormone, thyroid-stimulating hormone, adrenocorticotropic hormone levels	Maybe	Growth hormone, levothyroxine, hydrocortisone	medication	Childhood, adulthood	Endocrinologist				
HESX1	Combined pituitary hormone deficiency 5	endocrinology	AD, AR	N	2.31	Early childhood onset to adulthoood	Organic etiology, H-P abnormalities (in particular pituitary stalk abnormalities, empty sella and ectopic posterior pituitary), midline brain (corpus callosum) and optic nerves abnormalities, genetic defects and longer duration of follow-up	Y	Growth hormone, thyroid-stimulating hormone, luteinizing hormone, follicle-stimulating hormone, prolactin, adrenocorticotropic hormone levels		Growth hormone, levothyroxine, hydrocortisone	medication	Childhood, adulthood	Endocrinologist				
LEP	Leptin deficiency	endocrinology	AR	N		Early childhood onset	Reduction in energy balance, body weight, metabolism, & endocrine function	Y	Leptin level		Metreleptin	medication	Childhood	Endocrinologist				

© 2023 Gold NB et al. *JAMA Network Open.*

Endocrinology (95 genes)

Gene	Disease name	System	Inheritance	On RUSP? (Y/N)	Prevalence - disease frequency per 100,000 (Rx genes, if listed)	Age of Onset	Disease symptoms	Orthogonal test? (Y/N)	If yes, orthogonal test	Is orthogonal test expected to be abnormal in infancy?	Intervention Considered (Free Text)	Category of Intervention	Age of Intervention Implementation	MD leading intervention	Comments	Link to ref 1	Link to ref 2	Link to ref 3
LEPR	Leptin receptor deficiency	endocrinology	AR	N		Infancy	Early-onset severe obesity, hyperphagia and pituitary hormone deficiencies	N			Setmelanotide	medication	Infancy	Endocrinologist		https://pubmed.ncbi.nlm.nih.gov/31658438/		
GNAS	GNAS associated Pseudohypoparathyroidism	endocrinology	AD	N	0.7	Neonatal to adolesecence	Albright hereditary osteodystrophy, resistance toward PTH, TSH, & additional hormones	Y	Calcium, phosphate, parathyroid hormone, thyroid-stimulating hormone, growth hormone, IGF1, IGFBP3, methylation studies		Calcium, calcitriol, levothyroxine, growth hormone	medication	Infancy	Endocrinologist				
FOXE1	Bamforth-Lazarus syndrome	endocrinology	AR	N		Prenatal, neonatal	Congenital hypothyroidism (CH) with thyroid dysgenesis (usually athyreosis), cleft palate, spiky hair, with or without choanal atresia, and bifid epiglottis	Y	TSH, T4		Levolevothyroxine	medication	Infancy	Endocrinologist				
SOX3	X-linked panhypopituitarism	endocrinology	XLR	N		Infancy to adulthood (variable)	X-linked mental retardation X-linked retardation and isolated growth hormone deficiency or X-linked panhypopituitarism	Y	Growth hormone, levothyroxine, hydrocortisone		Growth hormone, levothyroxine, hydrocortisone	medication	Childhood, adulthood	Endocrinologist		https://rarediseases.info.nih.gov/diseases/414/bamforth-syndrome		
WFS1	Wolfram syndrome 1	endocrinology	AD, AR	N	0.87 (per 100,000) See INS per RXGenes	Early childhood onset	Juvenile-onset diabetes mellitus, diabetes insipidus, optic nerve atrophy, hearing loss, & neurodegeneration	Y	Glucose tolerance test, hemoglobin A1C, insulin level, glucose, TSH levels, hearing test, eye exam		Insulin, DDAVP, levothyroxine	medication	Childhood	Endocrinologist		https://rarediseases.org/rare-diseases/wolfram-syndrome/		
AAAS	Achalasia-addisonianism-alacrimia syndrome	endocrinology	AR	N		Infancy to adulthood	Esophageal achalasia, adrenocorticotropic hormone refractoriness, and alacrima	N			Hydrocortisone	medication	Infancy - adulthood	Endocrinologist		https://www.orpha.net/consor/cgi-bin/OC_Exp.php?Expert=869&lng=EN	https://pubmed.ncbi.nlm.nih.gov/29383495/	
AIRE	Autoimmune polyendocrinopathy syndrome, type I, with or without reversible metaphyseal dysplasia	endocrinology	AD, AR	N	0.805	Early onset childhood to young adulthood	Hypoparathyroidism, mucocutaneous candidiasis & Addison's disease	N			Prednisone, prednisolone, azathioprine, 6-mercaptopurine, mycophenolate mofetil, rituximab, oral swish/swallow suspension of amphotericin B, calcium, recombinant PTH, cyclosporine ophthalmic solution, mTOR inhibitor	medication	Childhood, Adulthood	Endocrinologist		https://rarediseases.org/rare-diseases/autoimmune-polyglandular-syndrome-type-1/		
PCSK1	Obesity with impaired prohormone processing	endocrinology	AR	N	32.262	Early-onset childhood	Obesity, early-onset, diarrhea, villous atrophy, hypoglycemia, impaired processing of proopiomelanocortin, hypocortisolemia, increased plasma proinsulin, decreased or normal plasma insulin, increased plasma progastrin, increased plasma proglucagon	N			Setmelanotide	medication	Childhood	Endocrinologist		https://www.omim.org/entry/600955		

© 2023 Gold NB et al. *JAMA Network Open.*

Gastroenterology (14 genes)

Gene	Disease name	System	Inheritance	On RUSP? (Y/N)	Prevalence - disease frequency per 100,000 (Rx genes, if listed)	Age of Onset	Disease symptoms	Orthogonal test? (Y/N)	If yes, orthogonal test	Is orthogonal test expected to be abnormal in infancy?	Intervention Considered (Free Text)	Category of Intervention	Age of Intervention Implementation	MD leading intervention	Comments	Link to ref 1	Link to ref 2	Link to ref 3
HSD3B7	Congenital bile acid synthesis defect type 1	gastroenterology	AR	N		Infancy	Cholestasis, malabsorption	Y	urine (FAB-MS) analysis	yes	cholic acid	medication	Infancy	pediatric GI				
AKR1D1	Congenital bile acid synthesis defect type 2	gastroenterology	AR	N		Infancy	Cholestasis, malabsorption	Y	urine (FAB-MS) analysis	yes	cholic acid	medication	Infancy	pediatric GI				
CYP7B1	Congenital bile acid synthesis defect type 3	gastroenterology	AR	N		Infancy	Cholestasis, malabsorption	Y	urine (FAB-MS) analysis	yes	cholic acid	medication	Infancy	pediatric GI				
LARS1	LARS1 associated Infantile liver failure syndrome 1	gastroenterology	AR	N		Infancy	Liver steatosis, fibrosis	Y	LFTs. liver biopsy	yes	leucine supplementation, increase protein intake during illness	diet medication	Infancy	pediatric metabolism				
TRMU	Transient infantile liver failure	gastroenterology	AR	N		Infancy	Transient infantile liver failure	Y	LFTs, lactate	yes	NAC	medication	Infancy	pediatric metabolism				
MTTP	Abetalipoproteinemia	gastroenterology	AR	N		Infancy	Poor weight gain, malabsorption	Y	lipid panel, apoB levels	yes	low-fat diet, fat-soluble vitamins	diet medication	Infancy	pediatric GI				
IL10RA	Inflammatory bowel disease 25	gastroenterology	AR	N		Infancy	Bloody diarrhea, rectal abscess	Y	flow cytometry	yes	HSCT	HSCT	Infancy	pediatric GI, pediatric heme/onc				
IL10RB	Inflammatory bowel disease 28	gastroenterology	AR	N		Infancy	Bloody diarrhea, rectal abscess	Y	flow cytometry	yes	HSCT	HSCT	Infancy	pediatric GI, pediatric heme/onc				
IL12RB1	Inflammatory bowel disease 25, early onset, autosomal recessive	gastroenterology	AR	N		Infancy	Diarrhea, inflammation		flow cytometry	yes	HSCT	HSCT	Infancy	pediatric GI, pediatric heme/onc				
SLC26A3	Congenital secretory chloride diarrhea	gastroenterology	AR	N		Infancy	Watery diarrhea, malabsorption	Y	fecal chloride content	yes	omeprazole, chloride, sodium, potassium	medication	Infancy	pediatric GI				
SLC9A3	Congenital secretory sodium diarrhea	gastroenterology	AR	N		Infancy	Watery diarrhea, malabsorption	Y	fecal sodium content	yes	sodium, bicarbonate	medication	Infancy	pediatric GI				
DGAT1	Diarrhea 7, protein-losing enteropathy type	gastroenterology	AR	N		Infancy	Diarrhea, protein losing enteropathy	Y	fecal alpha-1-antitrypsin	yes	low-fat diet	diet	Infancy	pediatric GI				
GPIHBP1	Glycosylphosphatidylinositol-anchored high-density lipoprotein-binding protein 1 (GPIHBP1) deficiency	gastroenterology	AR	N		Infancy	Fat malabsorption, pancreatitis, diarrhea	Y	triglyceride level	yes	volanesorsen, fat-restricted diet	diet medication	Infancy	pediatric GI				
SAR1B	Chylomicron retention disease	gastroenterology	AR	N		Infancy	Fat malabsorption	Y	lipid panel	yes	low-fat diet. fat-soluble vitamins	diet medication	Infancy	pediatric GI				

© 2023 Gold NB et al. *JAMA Network Open.*

Hematology (90 genes)

Gene	Disease name	System	Inheritance	On RUSP? (Y/N)	Prevalence - disease frequency per 100,000 (Rx genes, if listed)	Age of Onset	Disease symptoms	Orthogonal test? (Y/N)	If yes, orthogonal test	Is orthogonal test expected to be abnormal in infancy?	Intervention Considered (Free Text)	Category of Intervention	Age of Intervention Implementation	MD leading intervention	Comments	Link to ref 1	Link to ref 2	Link to ref 3
ALAS2	X-linked erythropoietic protoporphyria	hematology	XLR	N		Childhood	Sideroblastic anemia	Y	Complete blood count, ferritin, bone marrow aspiration, biospy		pyridoxine, folate, phlebotomy	medication procedure	Childhood	Hematologist				
UROD	Porphyria cutanea tarda	hematology	AD	N	7.5	Childhood/Adult	Porphyria	Y	Urine porphyrins		Phlebotomy chloroquine, hydroxychloroquine	medication procedure	Childhood	Hematologist				
ALAD	Aminolevulinic acid dehydratase deficiency porphyria	hematology	AR	N		Childhood	Porphyria, anemia	Y	Urine total porphyrins, Delta-aminolevulnic acid level		Hemin	medication	Childhood	Hematologist				
CPOX	Coproporphyria	hematology	AD	N		Childhood	Porphyria, anemia	Y	Urinary total porphobilinogen, coproporphyrin		Hemin	medication	Childhood	Hematologist				
FECH	Erythropoietic protoporphyria 1	hematology	AR	N	0.35	Childhood	Porphyria	Y	Free protoporphyrin		Afamelanotide	medication	Childhood	Hematologist				
HMBS	Acute intermittent porphyria	hematology	AD	N	1.4	Childhood/Adult	Porphyria	Y	Erythrocyte porphobilinogen deaminase activity		Givosiran, Hemin	medication	Childhood	Hematologist				
PPOX	Variegate porphyria	hematology	AD	N	0.9	Childhood/Adult	Porphyria	Y	Plasma porphyrin fluorescence		Hemin	medication	Childhood	Hematologist				
DNAJC21	Bone marrow failure syndrome 3	hematology	AR	N	1.3	Childhood	Neutropenia, pancytopenia, bone marrow failure	N			Oral pancreatic enzymes, fat-soluble vitamins, blood and/or platelet transfusions, granulocyte-colony stimulation factor, Hematopoietic Stem Cell Transplantation(HSCT), bone marrow transplant	medication transfusion HSCT	Childhood	Hematologist				
MYSM1	Bone marrow failure syndrome 4	hematology	AR	N		Childhood	Pancytopenia, bone marrow failure	Y	WBC & RBC counts, Immunoglobulin level, T & B Lymphocyte & natural killer cell profile		Hematopoietic stem cell transplantation (HSCT), bone marrow transplant	HSCT	Childhood	Hematologist				
GATA1	GATA1 associated X-Linked Cytopenia	hematology	XLR	N	0.75	Childhood	Anemia, thrombocytopenia, leukemia predisposition	Y	Complete blood count with reticulocyte count, erythrocyte adenosine deaminase activity, fetal hemoglobin, bone marrow aspiration & biopsy		Corticosteroids, red blood cell transfusion, Hematopoietic stem cell transplantation, bone marrow transplant(HCST), preventive measures for bleeding (DDAVP)	medication transfusion HSCT	Childhood	Hematologist				
SAMD9L	Ataxia-pancytopenia syndrome	hematology	AD	N		Childhood	Bone marrow failure, early onset myelodysplastic syndrome	Y	Bone marrow biopsy, aspirate		Hematopoietic stem cell transplantation (HSCT), bone marrow transplant	HSCT	Childhood	Hematologist				
LYST	Chediak-Higashi Syndrome	hematology	AR	N		Childhood	Recurrent pyogenic infections, albinism, peripheral neuropathy	Y	Peripheral blood smear		Hematopoietic Stem Cell Transplantation (HSCT), bone marrow transplant	HSCT	Childhood	Hematologist				
SBDS	Shwachman-Diamond syndrome	hematology	AR	N	1.3	Childhood	Neutropenia, pancytopenia, bone marrow failure	Y	Bone marrow biopsy, aspirate, pancreatic function analysis		Oral pancreatic enzymes/ Fat-soluble vitamins/ Blood and/or platelet transfusions/ Granulocyte-colony stimulation factor/ Hematopoietic Stem Cell Transplantation(HSCT) Bone Marrow Transplant	medication transfusion HSCT	Childhood	Hematologist				
EFL1	Shwachman-Diamond syndrome 2	hematology	AR	N	1.3	Childhood	Neutropenia, pancytopenia, bone marrow failure	Y	Bone marrow biopsy/aspirate		Oral pancreatic enzymes, fat-soluble vitamins, blood and/or platet transfusions, granulocyte-colony stimulation factor, Hematopoietic Stem Cell Transplantation(HSCT), bone marrow transplant	medication transfusion HSCT	Childhood	Hematologist				
SRP54	SRP54 associated Shwachman-Diamond syndrome	hematology	AD	N	1.3	Childhood	Neutropenia, pancytopenia, bone marrow failure	Y	Bone marrow biopsy/aspirate		Oral pancreatic enzymes, fat-soluble vitamins, blood and/or platelet transfusions, granulocyte-colony stimulation factor, Hematopoietic Stem Cell Transplantation(HSCT), bone marrow transplant	medication transfusion HSCT	Childhood	Hematologist				
FANCA	Fanconi anemia, complementation group A	hematology	AR	N	0.76	Childhood	Pancytopenia,bone marrow failure, cancer predisposition	Y	MMC and/or DEB induced chromosome breakage analysis		Hematopoiectic stem cell transplantation (HCST), bone marrow transplantation	HSCT	Childhood	Hematologist				
FANCB	Fanconi anemia, complementation group B	hematology	AR	N	0.76	Childhood	Pancytopenia,bone marrow failure, cancer predisposition	Y	MMC and/or DEB induced chromosome breakage analysis		Hematopoiectic stem cell transplantation (HCST), bone marrow transplantation	HSCT	Childhood	Hematologist				
FANCC	Fanconi anemia, complementation group C	hematology	AR	N	0.76	Childhood	Pancytopenia,bone marrow failure, cancer predisposition	Y	MMC and/or DEB induced chromosome breakage analysis		Hematopoiectic stem cell transplantation (HCST), bone marrow transplantation	HSCT	Childhood	Hematologist				
BRCA2	Fanconi anemia, complementation group D1	hematology	AR	N	0.76	Childhood	Pancytopenia,bone marrow failure, cancer predisposition	Y	MMC and/or DEB induced chromosome breakage analysis		Hematopoiectic stem cell transplantation (HCST), bone marrow transplantation	HSCT	Childhood	Hematologist				
FANCD2	Fanconi anemia, complementation group D2	hematology	AR	N	0.76	Childhood	Pancytopenia,bone marrow failure, cancer predisposition	Y	MMC and/or DEB induced chromosome breakage analysis		Hematopoiectic stem cell transplantation (HCST), bone marrow transplantation	HSCT	Childhood	Hematologist				
FANCE	Fanconi anemia, complementation group E	hematology	AR	N	0.76	Childhood	Pancytopenia,bone marrow failure, cancer predisposition	Y	MMC and/or DEB induced chromosome breakage analysis		Hematopoiectic stem cell transplantation (HCST), bone marrow transplantation	HSCT	Childhood	Hematologist				

© 2023 Gold NB et al. *JAMA Network Open.*

Hematology (90 genes)

Gene	Disease name	System	Inheritance	On RUSP? (Y/N)	Prevalence - disease frequency per 100,000 (Rx genes, if listed)	Age of Onset	Disease symptoms	Orthogonal test? (Y/N)	If yes, orthogonal test	Is orthogonal test expected to be abnormal in infancy?	Intervention Considered (Free Text)	Category of Intervention	Age of Intervention Implementation	MD leading intervention	Comments	Link to ref 1	Link to ref 2	Link to ref 3
FANCF	Fanconi anemia, complementation group F	hematology	AR	N	0.76	Childhood	Pancytopenia,bone marrow failure, cancer predisposition	Y	MMC and/or DEB induced chromosome breakage analysis		Hematopoiectic stem cell transplantation (HCST), bone marrow transplantation	HSCT	Childhood	Hematologist				
FANCG	Fanconi anemia, complementation group G	hematology	AR	N	0.76	Childhood	Pancytopenia,bone marrow failure, cancer predisposition	Y	MMC and/or DEB induced chromosome breakage analysis		Hematopoiectic stem cell transplantation (HCST), bone marrow transplantation	HSCT	Childhood	Hematologist				
FANCI	Fanconi anemia, complementation group I	hematology	AR	N	0.76	Childhood	Pancytopenia,bone marrow failure, cancer predisposition	Y	MMC and/or DEB induced chromosome breakage analysis		Hematopoiectic stem cell transplantation (HCST), bone marrow transplantation	HSCT	Childhood	Hematologist				
BRIP1	Fanconi anemia, complementation group J	hematology	AR	N	0.76	Childhood	Pancytopenia,bone marrow failure, cancer predisposition	Y	MMC and/or DEB induced chromosome breakage analysis		Hematopoiectic stem cell transplantation (HCST), bone marrow transplantation	HSCT	Childhood	Hematologist				
FANCL	Fanconi anemia, complementation group L	hematology	AR	N	0.76	Childhood	Pancytopenia,bone marrow failure, cancer predisposition	Y	MMC and/or DEB induced chromosome breakage analysis		Hematopoiectic stem cell transplantation (HCST), bone marrow transplantation	HSCT	Childhood	Hematologist				
PALB2	Fanconi anemia, complementation group N	hematology	AR	N	187	Childhood	Pancytopenia,bone marrow failure, cancer predisposition	N			Prophylactic mastectomy	surgery	Childhood	Hematologist				
RAD51C	Fanconi anemia, complementation group O	hematology	AR	N		Childhood	Pancytopenia,bone marrow failure, cancer predisposition	N					Childhood	Hematologist				
SLX4	Fanconi anemia, complementation group P	hematology	AR	N	0.76	Childhood	Pancytopenia,bone marrow failure, cancer predisposition	Y	MMC and/or DEB induced chromosome breakage analysis		Hematopoiectic stem cell transplantation (HCST), bone marrow transplantation	HSCT	Childhood	Hematologist				
ERCC4	Fanconi anemia, complementation group Q	hematology	AR	N	0.76	Childhood	Pancytopenia,bone marrow failure, cancer predisposition	Y	MMC and/or DEB induced chromosome breakage analysis		Hematopoiectic stem cell transplantation (HCST), bone marrow transplantation	HSCT	Childhood	Hematologist				
BRCA1	Fanconi anemia, complementation group S	hematology	AR	N	370	Childhood	Pancytopenia,bone marrow failure, cancer predisposition	N			Prophylactic mastectomy, prophylactic oophorectomy, chemoprevention	medication surgery	Childhood	Hematologist				
UBE2T	Fanconi anemia, complementation group T	hematology	AR	N	0.76	Childhood	Pancytopenia,bone marrow failure, cancer predisposition	Y	MMC and/or DEB induced chromosome breakage analysis		Hematopoiectic stem cell transplantation (HCST,)bone marrow transplantation	HSCT	Childhood	Hematologist				
MAD2L2	Fanconi anemia, complementation group V	hematology	AR	N	0.76	Childhood	Pancytopenia,bone marrow failure, cancer predisposition	Y	MMC and/or DEB induced chromosome breakage analysis		Hematopoiectic stem cell transplantation (HCST), bone marrow transplantation	HSCT	Childhood	Hematologist				
RFWD3	Fanconi anemia, complementation group W	hematology	AR	N	0.76	Childhood	Pancytopenia,bone marrow failure, cancer predisposition	Y	MMC and/or DEB induced chromosome breakage analysis		Hematopoiectic stem cell transplantation (HCST), bone marrow transplantation	HSCT	Childhood	Hematologist				
RPS19	Diamond-Blackfan anemia 1	hematology	AD	N	0.75	Childhood	Hypoplastic anemia, reticulocytopenia, craniofacial and limb defects in some patients	Y	Complete blood count with reticulocyte count, erythrocyte adenosine deaminase activity, fetal hemoglobin, bone marrow aspiration & biopsy		Corticosteroids, red blood cell transfusion, Hematopoietic Stem Cell Transplantation (HSCT), bone marrow transplant	medication transfusion HSCT	Childhood	Hematologist				
RPS24	Diamond-Blackfan anemia 3	hematology	AD	N	0.75	Childhood	Hypoplastic anemia, reticulocytopenia, craniofacial and limb defects in some patients	Y	Complete blood count with reticulocyte count, erythrocyte adenosine deaminase activity, fetal hemoglobin, bone marrow aspiration & biopsy		Corticosteroids, red blood cell transfusion, Hematopoietic Stem Cell Transplantation (HSCT), bone marrow transplant	medication transfusion HSCT	Childhood	Hematologist				
RPS17	Diamond-Blackfan anemia 4	hematology	AD	N	0.75	Childhood	Hypoplastic anemia, reticulocytopenia, craniofacial and limb defects in some patients	Y	Complete blood count with reticulocyte count, erythrocyte adenosine deaminase activity, fetal hemoglobin, bone marrow aspiration & biopsy		Corticosteroids, red blood cell transfusion, Hematopoietic Stem Cell Transplantation (HSCT), bone marrow transplant	medication transfusion HSCT	Childhood	Hematologist				
RPL35A	Diamond-Blackfan anemia 5	hematology	AD	N	0.75	Childhood	Hypoplastic anemia, reticulocytopenia, craniofacial and limb defects in some patients	Y	Complete blood count with reticulocyte count, erythrocyte adenosine deaminase activity, fetal hemoglobin, bone marrow aspiration & biopsy		Corticosteroids, red blood cell transfusion, Hematopoietic Stem Cell Transplantation (HSCT), bone marrow transplant	medication transfusion HSCT	Childhood	Hematologist				
RPL5	Diamond-Blackfan anemia 6	hematology	AD	N	0.75	Childhood	Hypoplastic anemia, reticulocytopenia, craniofacial and limb defects in some patients	Y	Complete blood count with reticulocyte count, erythrocyte adenosine deaminase activity, fetal hemoglobin, bone marrow aspiration & biopsy		Corticosteroids, red blood cell transfusion, Hematopoietic Stem Cell Transplantation (HSCT), bone marrow transplant	medication transfusion HSCT	Childhood	Hematologist				

© 2023 Gold NB et al. *JAMA Network Open.*

Hematology (90 genes)

Gene	Disease name	System	Inheritance	On RUSP? (Y/N)	Prevalence - disease frequency per 100,000 (Rx genes, if listed)	Age of Onset	Disease symptoms	Orthogonal test? (Y/N)	If yes, orthogonal test	Is orthogonal test expected to be abnormal in infancy?	Intervention Considered (Free Text)	Category of Intervention	Age of Intervention Implementation	MD leading intervention	Comments	Link to ref 1	Link to ref 2	Link to ref 3
RPL11	Diamond-Blackfan anemia 7	hematology	AD	N	0.75	Childhood	Hypoplastic anemia, reticulocytopenia, craniofacial and limb defects in some patients	Y	Complete blood count with reticulocyte count, erythrocyte adenosine deaminase activity, fetal hemoglobin, bone marrow aspiration & biopsy		Corticosteroids, red blood cell transfusion, Hematopoietic Stem Cell Transplantation (HSCT), bone marrow transplant	medication transfusion HSCT	Childhood	Hematologist				
RPS7	Diamond-Blackfan anemia 8	hematology	AD	N	0.75	Childhood	Hypoplastic anemia, reticulocytopenia, craniofacial and limb defects in some patients	Y	Complete blood count with reticulocyte count, erythrocyte adenosine deaminase activity, fetal hemoglobin, bone marrow aspiration & biopsy		Corticosteroids, red blood cell transfusion, Hematopoietic Stem Cell Transplantation (HSCT), bone marrow transplant	medication transfusion HSCT	Childhood	Hematologist				
RPS10	Diamond-Blackfan anemia 9	hematology	AD	N	0.75	Childhood	Hypoplastic anemia, reticulocytopenia, craniofacial and limb defects in some patients	Y	Complete blood count with reticulocyte count, erythrocyte adenosine deaminase activity, fetal hemoglobin, bone marrow aspiration & biopsy		Corticosteroids, red blood cell transfusion, Hematopoietic Stem Cell Transplantation (HSCT), bone marrow transplant	medication transfusion HSCT	Childhood	Hematologist				
RPS26	Diamond-Blackfan anemia 10	hematology	AD	N	0.75	Childhood	Hypoplastic anemia, reticulocytopenia, craniofacial and limb defects in some patients	Y	Complete blood count with reticulocyte count, erythrocyte adenosine deaminase activity, fetal hemoglobin, bone marrow aspiration & biopsy		Corticosteroids, red blood cell transfusion, Hematopoietic Stem Cell Transplantation (HSCT), bone marrow transplant	medication transfusion HSCT	Childhood	Hematologist				
RPL26	Diamond-Blackfan anemia 11	hematology	AD	N	0.75	Childhood	Hypoplastic anemia, reticulocytopenia, craniofacial and limb defects in some patients	Y	Complete blood count with reticulocyte count, erythrocyte adenosine deaminase activity, fetal hemoglobin, bone marrow aspiration & biopsy		Corticosteroids, red blood cell transfusion, Hematopoietic Stem Cell Transplantation (HSCT), bone marrow transplant	medication transfusion HSCT	Childhood	Hematologist				
RPL15	Diamond-Blackfan anemia 12	hematology	AD	N	0.75	Childhood	Hypoplastic anemia, reticulocytopenia, craniofacial and limb defects in some patients	Y	Complete blood count with reticulocyte count, erythrocyte adenosine deaminase activity, fetal hemoglobin, bone marrow aspiration & biopsy		Corticosteroids, red blood cell transfusion, Hematopoietic Stem Cell Transplantation (HSCT), bone marrow transplant	medication transfusion HSCT	Childhood	Hematologist				
RPS29	Diamond-Blackfan anemia 13	hematology	AD	N	0.75	Childhood	Hypoplastic anemia, reticulocytopenia, craniofacial and limb defects in some patients	Y	Complete blood count with reticulocyte count, erythrocyte adenosine deaminase activity, fetal hemoglobin, bone marrow aspiration & biopsy		Corticosteroids, red blood cell transfusion, Hematopoietic Stem Cell Transplantation (HSCT), bone marrow transplant	medication transfusion HSCT	Childhood	Hematologist				
TSR2	Diamond-Blackfan anemia 14 with mandibulofacial dysostosis	hematology	XLR	N	0.75	Childhood	Hypoplastic anemia, reticulocytopenia, craniofacial and limb defects in some patients	Y	Complete blood count with reticulocyte count, erythrocyte adenosine deaminase activity, fetal hemoglobin, bone marrow aspiration & biopsy		Corticosteroids, red blood cell transfusion, Hematopoietic Stem Cell Transplantation (HSCT), bone marrow transplant	medication transfusion HSCT	Childhood	Hematologist				
RPS28	Diamond Blackfan anemia 15 with mandibulofacial dysostosis	hematology	AD	N	0.75	Childhood	Hypoplastic anemia, reticulocytopenia, craniofacial and limb defects in some patients	Y	Complete blood count with reticulocyte count, erythrocyte adenosine deaminase activity, fetal hemoglobin, bone marrow aspiration & biopsy		Corticosteroids, red blood cell transfusion, Hematopoietic Stem Cell Transplantation (HSCT), bone marrow transplant	medication transfusion HSCT	Childhood	Hematologist				
RPL27	Diamond-Blackfan anemia 16	hematology	AD	N	0.75	Childhood	Hypoplastic anemia, reticulocytopenia, craniofacial and limb defects in some patients	Y	Complete blood count with reticulocyte count, erythrocyte adenosine deaminase activity, fetal hemoglobin, bone marrow aspiration & biopsy		Corticosteroids, red blood cell transfusion, Hematopoietic Stem Cell Transplantation (HSCT), bone marrow transplant	medication transfusion HSCT	Childhood	Hematologist				

© 2023 Gold NB et al. *JAMA Network Open.*

Hematology (90 genes)

Gene	Disease name	System	Inheritance	On RUSP? (Y/N)	Prevalence - disease frequency per 100,000 (Rx genes, if listed)	Age of Onset	Disease symptoms	Orthogonal test? (Y/N)	If yes, orthogonal test	Is orthogonal test expected to be abnormal in infancy?	Intervention Considered (Free Text)	Category of Intervention	Age of Intervention Implementation	MD leading intervention	Comments	Link to ref 1	Link to ref 2	Link to ref 3
RPS27	Diamond-Blackfan anemia 17	hematology	AD	N	0.75	Childhood	Hypoplastic anemia, reticulocytopenia, craniofacial and limb defects in some patients	Y	Complete blood count with reticulocyte count, erythrocyte adenosine deaminase activity, fetal hemoglobin, bone marrow aspiration & biopsy		Corticosteroids, red blood cell transfusion, Hematopoietic Stem Cell Transplantation (HSCT), bone marrow transplant	medication transfusion HSCT	Childhood	Hematologist				
RPL18	Diamond-Blackfan anemia 18	hematology	AD	N	0.75	Childhood	Hypoplastic anemia, reticulocytopenia, craniofacial and limb defects in some patients	Y	Complete blood count with reticulocyte count, erythrocyte adenosine deaminase activity, fetal hemoglobin, bone marrow aspiration & biopsy		Corticosteroids, red blood cell transfusion, Hematopoietic Stem Cell Transplantation (HSCT), bone marrow transplant	medication transfusion HSCT	Childhood	Hematologist				
RPL35	Diamond-Blackfan anemia 19	hematology	AD	N	0.75	Childhood	Hypoplastic anemia, reticulocytopenia, craniofacial and limb defects in some patients	Y	Complete blood count with reticulocyte count, erythrocyte adenosine deaminase activity, fetal hemoglobin, bone marrow aspiration & biopsy		Corticosteroids, red blood cell transfusion, Hematopoietic Stem Cell Transplantation (HSCT), bone marrow transplant	medication transfusion HSCT	Childhood	Hematologist				
RPS15A	Diamond-Blackfan anemia 20	hematology	AD	N	0.75	Childhood	Hypoplastic anemia, reticulocytopenia, craniofacial and limb defects in some patients	Y	Complete blood count with reticulocyte count, erythrocyte adenosine deaminase activity, fetal hemoglobin, bone marrow aspiration & biopsy		Corticosteroids, red blood cell transfusion, Hematopoietic Stem Cell Transplantation (HSCT), bone marrow transplant	medication transfusion HSCT	Childhood	Hematologist				
RPL31	RPL31 associated Diamond-Blackfan anemia	hematology	AD	N	0.75	Childhood	Hypoplastic anemia, reticulocytopenia, craniofacial and limb defects in some patients	Y	Complete blood count with reticulocyte count, erythrocyte adenosine deaminase activity, fetal hemoglobin, bone marrow aspiration & biopsy		Corticosteroids, red blood cell transfusion, Hematopoietic Stem Cell Transplantation (HSCT), bone marrow transplant	medication transfusion HSCT	Childhood	Hematologist				
G6PD	Hemolytic anemia due to G6PD deficiency	hematology	XLD	N		Childhood	Variable anemia, severe cases can also have recurrent pyogenic infections due to neutrophil dysfunction	Y	Functional testing of G6PD activity (quantitative and qualitative tests available, blood smear analysis		Nutritional restriction (oxidants), in several cases can consider intermittent RBC transfusion, splenectomy	diet transfusion surgery	Childhood	Hematologist				
SLC19A1	Folate dependent megaloblastic anemia	hematology	AR	N		Childhood	Anemia	Y	Plasma amino acids for homocysteine and sarcosine levels & urine 5-amino-4-imidazolecarboxamide riboside		Folic acid	medication	Childhood	Hematologist				
SLC46A1	Hereditary folate malabsorption	hematology	AR	N	1.72	Childhood	Anemia	Y	CSF & serum folate levels		5-formyltetrahydrofolate (5-formylTHF, folinic acid, Leucovorin) or the active isomer of 5-formylTHF (Isovorin or Fusilev) Parenteral (intra-muscular) or high-dose oral	medication	Childhood	Hematologist				
TF	Atransferrinemia	hematology	AR	N		Childhood	Anemia, hemosiderosis	Y	Serum transferrin level		Red cell transfusions, deferoxamine	medication transfusion	Childhood	Hematologist				
SLC25A38	Pyridoxine-refractory sideroblastic anemia 2	hematology	AR	N		Childhood	Anemia	Y	Complete blood count, ferritin, bone marrow aspiration and biopsy		Bone marrow transplantation, Hematopoietic Stem Cell Transplantation (HSCT)	HSCT	Childhood	Hematologist				
NBN	Nijmegen breakage syndrome	hematology	AR	N	1.72	Childhood	Progressive microcephaly, recurrent sinopulmonary infections	N			Hematopoietic stem cell transplantation (HSCT) , bone marrow transplant	HSCT	Childhood	Hematologist		https://www.ncbi.nlm.nih.gov/books/NBK1176/		
CBLIF	Intrinsic factor deficiency	hematology	AR	N		Most frequent in adults > 60 years old	Fatigue, weakness, incontinence	Y	Vitamin B12 level		Cobalamin	medication	Childhood	Hematologist		https://www.ncbi.nlm.nih.gov/books/NBK540989/		
RTEL1	Dyskeratosis congenita	hematology	AR	N		Childhood/Adult	Pancytopenia, bone marrow failure, pulmonary fibrosis	Y	Telomere length testing (flow cytometry or other modalities)		Hematopoietic stem cell transplantation (HSCT), bone marrow transplant	HSCT	Childhood	Hematologist				
TERC	Dyskeratosis congenita, autosomal dominant 1	hematology	AD	N		Childhood/Adult	Pancytopenia, bone marrow failure, pulmonary fibrosis	Y	Telomere length testing (flow cytometry or other modalities)		Hematopoietic stem cell transplantation (HSCT), bone marrow transplant	HSCT	Childhood	Hematologist				
TINF2	Dyskeratosis congenita, autosomal dominant 3	hematology	AD	N		Childhood/Adult	Pancytopenia, bone marrow failure, pulmonary fibrosis	Y	Telomere length testing (flow cytometry or other modalities)		Hematopoietic stem cell transplantation (HSCT), bone marrow transplant	HSCT	Childhood	Hematologist				
DKC1	Dyskeratosis congenita, X-linked	hematology	XLR	N		Childhood/Adult	Pancytopenia, bone marrow failure, pulmonary fibrosis	Y	Telomere length testing (flow cytometry or other modalities)		Hematopoietic stem cell transplantation (HSCT), bone marrow transplant	HSCT	Childhood	Hematologist				
ELANE	ELANE associated neutropenia 1	hematology	AD	N		Childhood	Severe neutropenia, recurrent infections	Y	Complete blood count, bone marrow aspiration & biopsy		Granulocyte colony-stimulating factor (G-CSF), bone marrow transplant (hematopoietic stem cell transplantation (HSCT))	medication HSCT	Childhood	Hematologist				

© 2023 Gold NB et al. *JAMA Network Open.*

Hematology (90 genes)

Gene	Disease name	System	Inheritance	On RUSP? (Y/N)	Prevalence - disease frequency per 100,000 (Rx genes, if listed)	Age of Onset	Disease symptoms	Orthogonal test? (Y/N)	If yes, orthogonal test	Is orthogonal test expected to be abnormal in infancy?	Intervention Considered (Free Text)	Category of Intervention	Age of Intervention Implementation	MD leading intervention	Comments	Link to ref 1	Link to ref 2	Link to ref 3
VPS45	Severe congenital neutropenia 5	hematology	AR	N		Childhood	Severe neutropenia, recurrent infections, myelodyplastic syndrome predisposition	Y	Complete blood count, bone marrow aspiration & biopsy		Bone marrow transplant (hematopoietic stem cell transplantation (HSCT)	HSCT	Childhood	Hematologist				
F8	Hemophilia A	hematology	XLR	N	7.5	Childhood	Bleeding	Y	Factor 8 level		Factor 8	medication	Childhood	Hematologist				
F9	Hemophilia B	hematology	XLR	N	1.335	Childhood	Bleeding	Y	Factor 9 level		Factor 9	medication	Childhood	Hematologist				
F13A1	Factor XIIIA deficiency	hematology	AR	N	0.065	Childhood	Bleeding	Y	Factor XIII activity		Tretten (coagulation Factor XIII A-Subunit (Recombinant)), fresh-frozen plasma (FFP), cryoprecipitate, or factor (F)XIII concentrates	medication transfusion	Childhood	Hematologist				
F13B	Factor XIIIB deficiency	hematology	AR	N	0.065	Childhood	Bleeding	Y	Immunoglobulin levels		Replacement immunoglobulin treatment	medication	Childhood	Hematologist				
GGCX	Combined deficiency of vitamin K-dependent clotting factors 1	hematology	AR	N		Childhood	Bleeding	Y	Quantitation of vitamin K dependent blood coagulation factors		Vitamin K	medication	Childhood	Hematologist				
VKORC1	Combined deficiency of vitamin K-dependent clotting factors 2	hematology	AR	N		Childhood	Bleeding	Y	Quantitation of vitamin K dependent blood coagulation factors		Vitamin K	medication	Childhood	Hematologist				
FGA	FGA associated afibrinogenemia	hematology	AR	N	0.1	Childhood	Bleeding	Y	Fibrinogen activity & antigen levels		Fibrinogen concentrate	transfusion	Childhood	Hematologist				
FGB	FGB associate afibrinogenemia	hematology	AR	N	0.1	Childhood	Bleeding	Y	Fibrinogen activity & antigen levels		Fibrinogen concentrate	transfusion	Childhood	Hematologist				
FGG	FGG associated afibrinogenemia	hematology	AR	N	0.1	Childhood	Bleeding	Y	Fibrinogen activity & antigen levels		Fibrinogen concentrate	transfusion	Childhood	Hematologist				
HOXA11	Radioulnar synostosis with amegakaryocytic thrombocytopenia 1	hematology	AD	N		Childhood	Thrombocytopenia, bone marrow failure, pancytopenia	Y	Complete blood count, bone marrow aspiration & biopsy		Hematopoietic stem cell transplantation (HSCT), bone marrow transplant	HSCT	Childhood	Hematologist				
MECOM	Radioulnar synostosis with amegakaryocytic thrombocytopenia 2	hematology	AD	N		Childhood	Thrombocytopenia, bone marrow failure, pancytopenia	Y	Complete blood count, bone marrow aspiration & biopsy		Hematopoietic stem cell transplantation (HSCT), bone marrow transplant	HSCT	Childhood	Hematologist				
MPL	Congenital amegakaryocytic thrombocytopenia	hematology	AR	N		Childhood	Thrombocytopenia, bone marrow failure, pancytopenia	Y	Complete blood count, bone marrow aspiration & biopsy		Hematopoietic stem cell transplantation (HSCT), bone marrow transplant	HSCT	Childhood	Hematologist				
WDR1	Periodic fever, immunodeficiency, and thrombocytopenia syndrome	hematology	AR	N		Childhood	Periodic fevers with immunodeficiency and low platelets	Y	T and B Lymphocyte and Natural Killer Cell Profile/ Peripheral smear		Hematopoietic stem cell transplantation (HSCT), bone marrow transplant	HSCT	Childhood	Hematologist				
ADAMTS13	Familial thrombotic thrombocytopenic purpura	hematology	AR	N		Childhood/Adult	Recurrent microangiopathy with infections	Y	ADAMTS13 activity		Plasma infusion or exchange	transfusion	Childhood	Hematologist				
AP3B1	Hermansky-Pudlak syndrome 2	hematology	AR	N		Childhood	Bleeding, albinism, visual impairment	Y	Natural killer cell activity/ Cytotoxic T lymphocyte activity		Hematopoietic Stem Cell Transplantation (HSCT), bone marrow transplant, granulocyte-colony stimulating factor (G-CSF)	medication HSCT	Childhood	Hematologist				
HFE	Hemochromatosis type 1	hematology	AR	N	458.335	Adult	Iron overload	N			Phlebotomy	procedure	Childhood	Hematologist				
HJV	Hemochromatosis, type 2A	hematology	AR	N		Childhood	Iron overload	Y	Transferrin saturation/ ferritin levels		Therapeutic phlebotomy	procedure	Childhood	Hematologist				
HAMP	Hemochromatosis, type 2B	hematology	AR	N		Adult	Iron overload	Y	Transferrin saturation/ ferritin levels		Therapeutic phlebotomy	procedure	Childhood	Hematologist				
TFR2	Hemochromatosis, type 3	hematology	AR	N		Adult	Iron overload	Y	Transferrin saturation/ ferritin levels		Therapeutic phlebotomy	procedure	Childhood	Hematologist				
SLC40A1	Hemochromatosis, type 4	hematology	AD	N		Adult	Iron overload	Y	Transferrin saturation/ ferritin levels		Therapeutic phlebotomy	procedure	Childhood	Hematologist				
HBA1	Alpha-thalassemia	hematology	AR	N	5.05	Childhood	Anemia	Y	Complete blood count, qualitative and quantitative hemoglobin analysis		Rarely (HbH disease/ Bart's Hydrops), red cell transfusions, bone marrow transplantation (Hematopoietic Stem Cell Transplantation (HSCT)	transfusion HSCT	Childhood	Hematologist				
HBA2	Alpha-thalassemia	hematology	AR	N	5.05	Childhood	Anemia	Y	Complete blood count, qualitative and quantitative hemoglobin analysis		Rarely (HbH disease/ Bart's Hydrops), red cell transfusions, bone marrow Transplantation (Hematopoietic Stem Cell Transplantation (HSCT)	transfusion HSCT	Childhood	Hematologist				
PIK3CA	PIK3CA related overgrowth spectrum	hematology	AD	N		Childhood	Regional overgrowth, vascular anomalies	N			Alpelisib, miransertib	medication	Childhood	Hematologist (Oncology at some centers)				

© 2023 Gold NB et al. *JAMA Network Open.*

Immunology (167 genes)

Gene	Disease name	System	Inheritance	On RUSP? (Y/N)	Prevalence - disease frequency per 100,000 (Rx genes, if listed)	Age of Onset	Disease symptoms	Orthogonal test? (Y/N)	If yes, orthogonal test	Is orthogonal test expected to be abnormal in infancy?	Intervention Considered (Free Text)	Category of Intervention	Age of Intervention Implementation	MD leading intervention	Comments	Link to ref 1	Link to ref 2	Link to ref 3
CORO1A	Immunodeficiency 8	immunology	AR	N	1.72		recurrent infections	Y	T and B Lymphocyte and Natural Killer Cell Profile	Y	Hematopoietic stem cell transplantation (HSCT) - bone marrow transplant	HSCT						
ORAI1	Immunodeficiency 9	immunology	AR	N	1.72		recurrent infections	Y	T cell proliferation assay	Y	Hematopoietic stem cell transplantation (HSCT) - bone marrow transplant	HSCT						
STIM1	Immunodeficiency 10	immunology	AR	N	1.72		recurrent infections	N	N/A	Y	Hematopoietic stem cell transplantation (HSCT) - bone marrow transplant	HSCT						
MALT1	Immunodeficiency 12	immunology	AR	N	ultrarare		recurrent infections	N	T cell proliferation assay	Y	prophalactic antibiotics, replacement immunoglobulin treatment, Bone marrow transplant (hematopoietic stem cell transplantation (HSCT))	medication transfusion HSCT						
PIK3CD	Immunodeficiency 14	immunology	AD, AR	N	0.4		recurrent infections, lymphoproliferat ion	Y	immunoglobulin levels, T and B Lymphocyte and Natural Killer Cell Profile	N	Replacement immunoglobulin treatment, rapamycin, leniolisib, Bone marrow transplant (hematopoietic stem cell transplantation (HSCT)) - treatment for AR and AD forms differ	medication transfusion HSCT						
IKBKB	Immunodeficiency 15, 15B	immunology	AR	N	ultrarare		recurrent infections	Y	Treg, gamma/delta T cells	Y	Hematopoietic stem cell transplantation (HSCT) - bone marrow transplant	HSCT						
CD3E	Immunodeficiency 18	immunology	AR	N	1.72		recurrent infections	Y	T and B Lymphocyte and Natural Killer Cell Profile	Y	Hematopoietic stem cell transplantation (HSCT) - bone marrow transplant	HSCT						
CD3D	Immunodeficiency 19	immunology	AR	N	1.72		recurrent infections	Y	T and B Lymphocyte and Natural Killer Cell Profile	Y	Hematopoietic stem cell transplantation (HSCT) - bone marrow transplant	HSCT						
GATA2	Immunodeficiency 21	immunology	AD	N	unk		recurrent infections, mielodysplasia	N	N/A	N	Hematopoietic stem cell transplantation (HSCT) - bone marrow transplant	HSCT						
LCK	Immunodeficiency 22	immunology	AR	N	1.72		recurrent infections	Y	T and B Lymphocyte and Natural Killer Cell Profile	Y	Hematopoietic stem cell transplantation (HSCT) - bone marrow transplant	HSCT						
PGM3	Immunodeficiency 23	immunology	AR	N	1.72		recurrent infections, atopy	Y	T and B Lymphocyte and Natural Killer Cell Profile	Y	immunoglobulin replacement, Hematopoietic stem cell transplantation (HSCT) - bone marrow transplant	transfusion HSCT						
CTPS1	Immunodeficiency 24	immunology	AR	N	ultrarare		recurrent infections, autoimmunity	Y	T and B Lymphocyte and Natural Killer Cell Profile	Y	Hematopoietic stem cell transplantation (HSCT) - bone marrow transplant	HSCT						
CD247	Immunodeficiency 25	immunology	AR	N	1.72		recurrent infections	Y	T and B Lymphocyte and Natural Killer Cell Profile	Y	Hematopoietic stem cell transplantation (HSCT) - bone marrow transplant	HSCT						
PRKDC	Immunodeficiency 26	immunology	AR	N	1.72		recurrent infections	Y	T and B Lymphocyte and Natural Killer Cell Profile	Y	Hematopoietic stem cell transplantation (HSCT) - bone marrow transplant	HSCT						
IFNGR2	Immunodeficiency 27A	immunology	AR	N	ultrarare		recurrent infections	Y	flow cytometric analysis	Y	Hematopoietic stem cell transplantation (HSCT) - bone marrow transplant	HSCT						
IFNGR1	Immunodeficiency 27B	immunology	AD, AR	N	ultrarare		recurrent infections	Y	flow cytometric analysis	Y	Hematopoietic stem cell transplantation (HSCT) - bone marrow transplant	HSCT						
IL17RA	Immunodeficiency 30	immunology	AR	N	ultrarare		recurrent infections	Y	flow cytometric analysis	Y	Hematopoietic stem cell transplantation (HSCT) - bone marrow transplant	HSCT						
STAT1GOF	Immunodeficiency 31B	immunology	AR	N			recurrent infections	Y	N/A	N	Bone marrow transplant (hematopoietic stem cell transplantation (HSCT)), Ruxolitinib	medication HSCT						
IRF8	Immunodeficiency 32B	immunology	AR	N	ultrarare		recurrent infections	Y	Monocyte and dentritic cell quantitation	Y	Bone marrow transplant (hematopoietic stem cell transplantation (HSCT)), Ruxolitinib	HSCT						
TYK2	Immunodeficiency 35	immunology	AR	N	ultrarare		recurrent infections				Hematopoietic stem cell transplantation (HSCT) - bone marrow transplant	HSCT						
IL2RA	Immunodeficiency 41 with lymphoproliferation and autoimmunity	immunology	AR	N	ultrarare		recurrent infections	Y	flow cytometric analysis	Y	Rapamycin, hematopoietic stem cell transplantation (HSCT) - bone marrow transplant	medication HSCT						
ZAP70	Immunodeficiency 48	immunology	AR	N	1.72		recurrent infections	Y	T and B Lymphocyte and Natural Killer Cell Profile	Y	Hematopoietic stem cell transplantation (HSCT) - bone marrow transplant	HSCT						
RELB	Immunodeficiency 53	immunology	AR	N	unk		recurrent infections	Y	T and B Lymphocyte and Natural Killer Cell Profile	Y	Hematopoietic stem cell transplantation (HSCT) - bone marrow transplant, immunoglobulin replacement	transfusion HSCT						
MCM4	Immunodeficiency 54	immunology	AR	N	unk		recurrent infections	Y	serum cortisol and adrenocorticotrop ic hormone (ACTH) levels, T and B Lymphocyte and Natural Killer Cell Profile	Y	Hydrocortisone	medication						
IL21R	Immunodeficiency 56	immunology	AR	N	unk		recurrent infections	Y	immunoglobulin levels	Y	Bone marrow transplant (hematopoietic stem cell transplantation (HSCT))	HSCT						
IL2RB	Immunodeficiency 63 with lymphoproliferation and autoimmunity	immunology	AR	N	unk		recurrent infections	Y	flow cytometry	Y	Hematopoietic stem cell transplantation (HSCT) - bone marrow transplant	HSCT						
RASGRP1	Immunodeficiency 64	immunology	AR	N	unk		recurrent infections, malignancy	Y	T and B Lymphocy	Y	Bone marrow transplant (hematopoietic stem cell transplantation (HSCT))	HSCT						
CD40LG	X-linked immunodeficiency with hyper-IgM type 1	immunology	XLR	N	0.1		recurrent infections	Y	immunoglobulin levels, flow cytometric analysis	Y	Hematopoietic Stem Cell Transplantation (HSCT) Bone marrow transplant. Prophylaxis for pneumonia secondary to Pneumocystis jiroveci. immunoglobulin replacement	medication transfusion HSCT						

© 2023 Gold NB et al. *JAMA Network Open.*

Immunology (167 genes)

Gene	Disease name	System	Inheritance	On RUSP? (Y/N)	Prevalence - disease frequency per 100,000 (Rx genes, if listed)	Age of Onset	Disease symptoms	Orthogonal test? (Y/N)	If yes, orthogonal test	Is orthogonal test expected to be abnormal in infancy?	Intervention Considered (Free Text)	Category of Intervention	Age of Intervention Implementation	MD leading intervention	Comments	Link to ref 1	Link to ref 2	Link to ref 3
AICDA	Immunodeficiency with hyper-IgM, type 2	immunology	AR	N	unk		recurrent infections	Y	immunoglobulin levels	Y	replacement immunoglobulin treatment	transfusion						
CD40	Immunodeficiency with hyper-IgM, type 3	immunology	AR	N	unk		recurrent infections	Y	immunoglobulin levels, flow cytometric analysis	Y	Bone marrow transplant (hematopoietic stem cell transplantation (HSCT))	HSCT						
UNG	Immunodeficiency with hyper IgM, type 5	immunology	AR	N	unk		recurrent infections	Y	immunoglobulin levels	Y	immunoglobulin replacement	transfusion						
DNMT3B	Immunodeficiency-centromeric instability-facial anomalies syndrome 1	immunology	AR	N	unk		recurrent infections	Y	immunoglobulin levels, cytogenetic analysis for centromeric instability, DNA methylation studies	Y	replacement immunoglobulin treatment, Bone marrow transplant (hematopoietic stem cell transplantation (HSCT))	transfusion HSCT						
ZBTB24	Immunodeficiency-centromeric instability-facial anomalies syndrome 2	immunology	AR	N	unk		recurrent infections	Y	immunoglobulin levels, cytogenetic analysis for centromeric instability, DNA methylation studies	Y	replacement immunoglobulin treatment, Bone marrow transplant (hematopoietic stem cell transplantation (HSCT))	transfusion HSCT						
CDCA7	Immunodeficiency-centromeric instability-facial anomalies syndrome 3	immunology	AR	N	unk		recurrent infections, facial anomalie	Y	immunoglobulin levels, cytogenetic analysis for centromeric instability, DNA methylation studies	Y	replacement immunoglobulin treatment, Bone marrow transplant (hematopoietic stem cell transplantation (HSCT))	transfusion HSCT						
HELLS	Immunodeficiency-centromeric instability-facial anomalies syndrome 4	immunology	AR	N	unk		recurrent infections, facial anomalies	Y	immunoglobulin levels, cytogenetic analysis for centromeric instability, DNA methylation studies	Y	replacement immunoglobulin treatment, Bone marrow transplant (hematopoietic stem cell transplantation (HSCT))	transfusion HSCT						
DCLRE1C	Omenn syndrome/Severe combined immunodeficiency, Athabascan type	immunology	AR	N	unk		recurrent infections	Y	T and B Lymphocyte and Natural Killer Cell Profile	Y	Hematopoietic stem cell transplantation (HSCT) - bone marrow transplant	transfusion HSCT						
FOXN1	T-cell immunodeficiency with congenital alopecia and nail dystrophy	immunology	AR, AD	N	unk		recurrent infections, alopecia, nail distrophy	Y	T and B Lymphocyte and Natural Killer Cell Profile	Y	thymus transplantation	OT						
LAMTOR2	MAPBP-interacting protein associated immunodeficiency	immunology	AR	N	unk		recurrent infections	Y	complete blood count, bone marrow aspiration and biopsy, immunoglobulin level	Y	Granulocyte colony-stimulating factor (G-CSF)	medication						
LIG1	LIG1 associated immunodeficiency	immunology	AR	N	unk		recurrent infections	Y	immunoglobulin levels, T and B Lymphocyte and Natural Killer Cell Profile, complete blood count	Y	replacement immunoglobulin treatment, Bone marrow transplant (hematopoietic stem cell transplantation (HSCT))	transfusion HSCT						
MAGT1	X-linked Immunodeficiency with magnesium defect, Epstein-Barr virus infecti	immunology	XLR	N	unk		recurrent infections	Y	immunoglobulin levels, T and B Lymphocyte and Natural Killer Cell Profile, Carbohydrate deficient glycosylation profile	Y	magnesium, Bone marrow transplant (hematopoietic stem cell transplantation (HSCT)), replacement immunoglobulin treatment	medication transfusion HSCT						
MAP3K14	MAP3K14 associated immunodeficiency	immunology	AR	N	unk		recurrent infections	Y	immunoglobulin levels, T and B Lymphocyte and Natural Killer Cell Profile	Y	Bone marrow transplant (hematopoietic stem cell transplantation (HSCT))	HSCT						
MTHFD1	Combined immunodeficiency and megaloblastic anemia with or without hype	immunology	AR	N	1.72		recurrent infections	Y	T and B Lymphocyte and Natural Killer Cell Profile, complete blood count with MCV, plasma homocysteine and methylmalonic acid levels, CSF 5_methyltetrahyd rofolate level	Y	hydroxocobalamin, folinic acid and betaine	medication						
NFE2L2	NRF2 superactivity (immunodeficiency, developmental delay, and hypohomo	immunology	AD	N	unk		recurrent infections	N/A										
NFKBIA	Ectodermal dysplasia and immunodeficiency 2	immunology	AD	N	0.5		recurrent infections	N	N/A		Hematopoietic stem cell transplantation (HSCT) - bone marrow transplant	HSCT						
RAG2	RAG2 associated T cell-negative, B cell-negative, severe combined immunod	immunology	AR	N	1.72		recurrent infections	Y	T and B Lymphocyte and Natural Killer Cell Profile	Y	Hematopoietic stem cell transplantation (HSCT) - bone marrow transplant	HSCT						
SP110	Hepatic venoocclusive disease with immunodeficiency	immunology	AR	N	1.72		recurrent infections, VODI	Y	T and B Lymphocyte and Natural Killer Cell Profile, immunoglobulin levels	Y	Hematopoietic stem cell transplantation (HSCT) - bone marrow transplant, immunoglobulin replacement	transfusion HSCT						
STAT5B	Growth hormone insensitivity with immunodeficiency	immunology	AR	N	ultrarare		recurrent infections											
STK4	STK4 associated T-cell immunodeficiency, recurrent infections, autoimmunity	immunology	AR	N	unk		recurrent infections	Y	T and B Lymphocyte and Natural Killer Cell Profile	Y	Bone marrow transplant (hematopoietic stem cell transplantation (HSCT))	HSCT						

© 2023 Gold NB et al. *JAMA Network Open.*

Immunology (167 genes)

Gene	Disease name	System	Inheritance	On RUSP? (Y/N)	Prevalence - disease frequency per 100,000 (Rx genes, if listed)	Age of Onset	Disease symptoms	Orthogonal test? (Y/N)	If yes, orthogonal test	Is orthogonal test expected to be abnormal in infancy?	Intervention Considered (Free Text)	Category of Intervention	Age of Intervention Implementation	MD leading intervention	Comments	Link to ref 1	Link to ref 2	Link to ref 3
TRNT1	Sideroblastic anemia with B-cell immunodeficiency, periodic fevers, and deve	immunology	AR	N	unk		recurrent infections	Y	immunoglobulin levels, T and B Lymphocyte and Natural Killer Cell Profile	Y	Bone marrow transplant (hematopoietic stem cell transplantation (HSCT)), replacement immunoglobulin treatment	transfusion HSCT						
TTC7A	Gastrointestinal defects and immunodeficiency syndrome	immunology	AR	N	1.72		recurrent infections, intestinal atresia	Y	T and B Lymphocyte and Natural Killer Cell Profile, immunoglobulin levels	Y	leflunomide, immunoglobulin replacement, hematopoietic stem cell transplantation (HSCT) - bone marrow transplant	medication transfusion HSCT						
CARD11	B-cell expansion with NKFB and T-cell anergy/Immunodeficiency 11B with at	immunology	AD	N	unk		recurrent infections	Y	complete blood count, immunoglobulin levels		replacement immunoglobulin treatment, Bone marrow transplant (hematopoietic stem cell transplantation (HSCT))	transfusion HSCT						
CUBN	Imerslund-Grasbeck syndrome 1	immunology	AR	N	unk		recurrent infections	Y	vitamin B12 level		cobalamin	medication						
AMN	Imerslund-Grasbeck syndrome 2	immunology	AR	N	unk		recurrent infections	Y	vitamin B12 level		cobalamin	medication						
IGHM	Agammaglobulinemia 1	immunology	AR	N	0.75		recurrent infections	Y	immunoglobulin levels, T and B Lymphocyte and Natural Killer Cell Profile	Y	replacement immunoglobulin treatment, Bone marrow transplant (hematopoietic stem cell transplantation (HSCT))	transfusion HSCT						
IGLL1	Agammaglobulinemia 2	immunology	AR	N	0.75		recurrent infections	Y	immunoglobulin levels, T and B Lymphocyte and Natural Killer Cell Profile		replacement immunoglobulin treatment	transfusion						
CD79A	Agammaglobulinemia 3	immunology	AR	N	0.75		recurrent infections	Y	immunoglobulin levels, T and B Lymphocyte and Natural Killer Cell Profile		replacement immunoglobulin treatment	transfusion						
BLNK	Agammaglobulinemia 4	immunology	AR	N	0.75		recurrent infections	Y	immunoglobulin levels, T and B Lymphocyte and Natural Killer Cell Profile, complete blood count	Y	replacement immunoglobulin treatment	transfusion						
CD79B	Agammaglobulinemia 6	immunology	AR	N	0.75		recurrent infections	Y	immunoglobulin levels, T and B Lymphocyte and Natural Killer Cell Profile		replacement immunoglobulin treatment	transfusion						
PIK3R1	Agammaglobulinemia 7	immunology	AD, AR	N	0.75		recurrent infections, lymphproliferati on	Y	immunoglobulin levels, T and B Lymphocyte	Y	~~Plasma infusion or exchange~~, Bone marrow transplantation Hematopoietic Stem Cell Transplantation (HSCT)	HSCT						
TCF3	Agammaglobulinemia 8	immunology	AD, AR	N	0.75		recurrent infections	Y	immunoglobulin levels, T and B Lymphocyte and Natural Killer Cell Profile		replacement immunoglobulin treatment	transfusion						
BTK	X-linked agammaglobulinemia	immunology	XLR	N	0.75		recurrent infections	Y	immunoglobulin levels, T and B Lymphocyte and Natural Killer Cell Profile, complete blood count	Y	replacement immunoglobulin treatment/Hematopoietic Stem Cell Transplantation (HSCT)	transfusion HSCT						
C1QA	C1QA associated C1q deficiency	immunology	AR	N	unk		recurrent infections	Y	classical pathway and alternative pathway of the complement system functional activity tests	Y	Plasma infusion or exchange, Bone marrow transplantation Hematopoietic Stem Cell Transplantation (HSCT)	transfusion HSCT						
C1QB	C1QB associated C1q deficiency	immunology	AR	N	unk		recurrent infections	Y	classical pathway and alternative pathway of the complement system functional activity tests	Y	Plasma infusion or exchange, Bone marrow transplantation Hematopoietic Stem Cell Transplantation (HSCT)	transfusion HSCT						
C1QC	C1QC associated C1q deficiency	immunology	AR	N	unk		recurrent infections	Y	classical pathway and alternative pathway of the complement system functional activity tests	Y	Plasma infusion or exchange, Bone marrow transplantation Hematopoietic Stem Cell Transplantation (HSCT)	transfusion HSCT						
C2	C2 deficiency	immunology	AR	N	5		recurrent infections	Y	classical pathway and alternative pathway of the complement system functional activity tests	Y	pneumococcal, meningococcal, haemophilus influenzae vaccines	vaccination						
C3	C3 deficiency	immunology	AR	N	0.2		recurrent infections	Y	classical pathway and alternative pathway of the complement system functional activity tests	Y	pneumococcal, meningococcal, haemophilus influenzae vaccines	vaccination						
C5	C5 deficiency	immunology	AR	N	unk		recurrent infections	Y	classical pathway and alternative pathway of the complement system functional activity tests	Y	pneumococcal, meningococcal, haemophilus influenzae vaccines	vaccination						
C6	C6 deficiency	immunology	AR	N	unk		recurrent infections	Y	classical pathway and alternative pathway of the complement system functional activity tests	Y	pneumococcal, meningococcal, haemophilus influenzae vaccines	vaccination						
C7	C7 deficiency	immunology	AR	N	unk		recurrent infections	Y	classical pathway and alternative pathway of the complement system functional activity tests	Y	pneumococcal, meningococcal, haemophilus influenzae vaccines	vaccination						

© 2023 Gold NB et al. *JAMA Network Open.*

Immunology (167 genes)

Gene	Disease name	System	Inheritance	On RUSP? (Y/N)	Prevalence - disease frequency per 100,000 (Rx genes, if listed)	Age of Onset	Disease symptoms	Orthogonal test? (Y/N)	If yes, orthogonal test	Is orthogonal test expected to be abnormal in infancy?	Intervention Considered (Free Text)	Category of Intervention	Age of Intervention Implementation	MD leading intervention	Comments	Link to ref 1	Link to ref 2	Link to ref 3
C8A	C8 deficiency, type I	immunology	AR	N	unk		recurrent infections	Y	classical pathway and alternative pathway of the complement system functional activity tests	Y	pneumococcal, meningococcal, haemophilus influenzae vaccines	vaccination						
C8B	C8 deficiency, type II	immunology	AR	N	unk		recurrent infections	Y	classical pathway and alternative pathway of the complement system functional activity tests	Y	pneumococcal, meningococcal, haemophilus influenzae vaccines	vaccination						
C9	C9 deficiency	immunology	AR	N	unk		recurrent infections	Y	classical pathway and alternative pathway of the complement system functional activity tests	Y	pneumococcal, meningococcal, haemophilus influenzae vaccines	vaccination						
ICOS	Common variable immune deficiency 1	immunology	AR	N	0.4		recurrent infections	Y	immunoglobulin levels	Y	replacement immunoglobulin treatment, Bone marrow transplant (hematopoietic stem cell transplantation (HSCT))	transfusion HSCT						
TNFRSF13B	Common variable immune deficiency 2	immunology	AD	N	0.4		recurrent infections	Y	immunoglobulin levels	N	replacement immunoglobulin treatment	transfusion						
CD19	Common variable immune deficiency 3	immunology	AR	N	0.4		recurrent infections	Y	immunoglobulin levels	Y	replacement immunoglobulin treatment	transfusion						
TNFRSF13C	Common variable immune deficiency 4	immunology	AR	N	0.4		recurrent infections	Y	immunoglobulin levels		replacement immunoglobulin treatment	transfusion						
MS4A1	Common variable immune deficiency 5	immunology	AR	N	0.4		recurrent infections	Y	immunoglobulin levels		replacement immunoglobulin treatment	transfusion						
CD81	Common variable immune deficiency 6	immunology	AR	N	0.4		recurrent infections	Y	immunoglobulin levels		replacement immunoglobulin treatment	transfusion						
CR2	Common variable immune deficiency 7	immunology	AR	N	0.4		recurrent infections	Y	immunoglobulin levels		replacement immunoglobulin treatment	transfusion						
LRBA	Common variable immune deficiency 8	immunology	AR	N	0.4		recurrent infections	Y	immunoglobulin levels	Y	Abatacept, Bone marrow transplant (hematopoietic stem cell transplantation (HSCT))	medication HSCT						
NFKB2	Common variable immune deficiency 10	immunology	AD	N	0.4		recurrent infections, autoimmunity	Y	immunoglobulin levels		replacement immunoglobulin treatment, cortisol	medication transfusion						
IL21	Common variable immune deficiency 11	immunology	AR	N	0.4		recurrent infections	Y	immunoglobulin levels	Y	replacement immunoglobulin treatment	transfusion						
NFKB1	Common variable immune deficiency 12	immunology	AD	N	0.4		recurrent infections	Y	immunoglobulin levels	N	replacement immunoglobulin treatment	transfusion						
IKZF1	Common variable immune deficiency 13	immunology	AD	N	0.4		recurrent infections	Y	immunoglobulin levels	Y	replacement immunoglobulin treatment, Bone marrow transplant (hematopoietic stem cell transplantation (HSCT))	transfusion HSCT						
IRF2BP2	Common variable immune deficiency 14	immunology	AD	N	0.4		recurrent infections	Y	immunoglobulin levels	Y	replacement immunoglobulin treatment	transfusion						
ITK	Lymphoproliferative syndrome 1	immunology	AR	N	0.5		recurrent infections	Y	immunoglobulin levels, T and B Lymphocyte and Natural Killer Cell Profile	Y	Bone marrow transplant (hematopoietic stem cell transplantation (HSCT))	HSCT						
CD27	Lymphoproliferative syndrome 2	immunology	AR	N	unk		recurrent infections	Y	immunoglobulin levels	Y	replacement immunoglobulin treatment, rituximab, Bone marrow transplant (hematopoietic stem cell transplantation (HSCT))	medication transfusion HSCT						
CD70	Lymphoproliferative syndrome 3	immunology	AR	N	ultrarare		recurrent infections	Y	immunoglobulin levels, T and B Lymphocyte and Natural Killer Cell Profile	Y	Bone marrow transplant (hematopoietic stem cell transplantation (HSCT))	HSCT						
PRKCD	Autoimmune lymphoproliferative syndrome, type III	immunology	AR	N	ultrarare		recurrent infections	N	N/A	Y	rituximab, ofatumumab	medication						
CTLA4	Autoimmune lymphoproliferative syndrome, type V	immunology	AD	N	ultrarare		recurrent infections, autoimmunity	Y	immunoglobulin levels, T and B Lymphocyte and Natural Killer Cell Profile	N	Abatacept, Bone marrow transplant (hematopoietic stem cell transplantation (HSCT))	medication HSCT						
SH2D1A	X-linked lymphoproliferative syndrome 1	immunology	XLR	N	0.1		recurrent infections	Y	~~invariant natural killer T-cell quantitation~~	Y	Emapalumab, Hematopoietic stem cell transplantation (HSCT) - bone marrow transplant	medication HSCT						
XIAP	X-linked lymphoproliferative syndrome 2	immunology	XLR	N	0.1		recurrent infections	N	invariant natural killer T-cell quantitation	Y	Emapalumab, IL18BP, Hematopoietic stem cell transplantation (HSCT) - bone marrow transplant	medication HSCT						
CFHR1	CFHR1 associated susceptibility to atypical hemolytic uremic syndrome	immunology	AD, AR	N	0.2			N	N/A		Eculizumab, Ravulizumab, Plasma infusion or exchange	medication transfusion						
CD46	Susceptibility to atypical hemolytic uremic syndrome 2	immunology	AD, AR	N	0.2			N	N/A		Eculizumab, Ravulizumab, Plasma infusion or exchange	medication transfusion						
THBD	Susceptibility to atypical hemolytic uremic syndrome 6	immunology	AD	N	0.2			N	N/A		Eculizumab, Ravulizumab, Plasma infusion or exchange	medication transfusion						
CFB	Complement factor B deficiency	immunology	AD	N	0.2			N	N/A		Eculizumab, Ravulizumab, Plasma infusion or exchange	medication transfusion						
CFD	Complement factor D deficiency	immunology	AR	N	unk			Y	classical pathway and alternative pathway of the complement system functional activity tests	Y	pneumococcal, meningococcal, haemophilus influenzae vaccines	vaccination						
CFH	Complement factor H deficiency	immunology	AD, AR	N	0.2			N	N/A		Eculizumab, Ravulizumab, Plasma infusion or exchange	medication transfusion						
CFI	Complement factor I deficiency	immunology	AD	N	0.2		recurrent infections	N	N/A		Eculizumab, Ravulizumab, Plasma infusion or exchange	medication transfusion						
CIITA	Bare lymphocyte syndrome, type II, complementation group A	immunology	AR	N	1.72		recurrent infections	Y	flow cytometric analysis of HLA-DR expression on monocytes and B cells	Y	Hematopoietic stem cell transplantation (HSCT) - bone marrow transplant	HSCT						
RFX5	Bare lymphocyte syndrome, type II, complementation group C and group E	immunology	AR	N	1.72		recurrent infections	Y	flow cytometric analysis of HLA-DR expression on monocytes and B cells	Y	Hematopoietic stem cell transplantation (HSCT) - bone marrow transplant	HSCT						

© 2023 Gold NB et al. *JAMA Network Open.*

Immunology (167 genes)

Gene	Disease name	System	Inheritance	On RUSP? (Y/N)	Prevalence - disease frequency per 100,000 (Rx genes, if listed)	Age of Onset	Disease symptoms	Orthogonal test? (Y/N)	If yes, orthogonal test	Is orthogonal test expected to be abnormal in infancy?	Intervention Considered (Free Text)	Category of Intervention	Age of Intervention Implementation	MD leading intervention	Comments	Link to ref 1	Link to ref 2	Link to ref 3
RFXAP	Bare lymphocyte syndrome, type II, complementation group D	immunology	AR	N	1.72		recurrent infections	Y	flow cytometric analysis of HLA-DR expression on monocytes and B cells	Y	Hematopoietic stem cell transplantation (HSCT) - bone marrow transplant	HSCT						
COL7A1	Epidermolysis bullosa	immunology	AR	N	unk		recurrent infections											
KRT14	Epidermolysis bullosa	immunology	AD	N	unk													
KRT5	Epidermolysis bullosa	immunology	AD	N	unk													
GFI1	Severe congenital neutropenia 2	immunology	AD	N	unk			Y	complete blood count	Y	granulocyte colony-stimulating factor (G-CSF), Bone marrow transplant (hematopoietic stem cell transplantation (HSCT))	medication HSCT						
HAX1	Severe congenital neutropenia 3	immunology	AR	N	unk			Y	complete blood count, bone marrow aspiration and biopsy	Y	granulocyte colony-stimulating factor (G-CSF), Bone marrow transplant (hematopoietic stem cell transplantation (HSCT))	medication HSCT						
G6PC3	Severe congenital neutropenia 4	immunology	AR	N	0.04			Y	complete blood count, bone marrow aspiration and biopsy	Y	granulocyte colony-stimulating factor (G-CSF), Bone marrow transplant (hematopoietic stem cell transplantation (HSCT))	medication HSCT						
JAGN1	Severe congenital neutropenia 6	immunology	AR	N	unk		recurrent infections	Y	complete blood count, bone marrow aspiration and biopsy	Y	granulocyte colony-stimulating factor (G-CSF), Bone marrow transplant (hematopoietic stem cell transplantation (HSCT))	medication HSCT						
CSF3R	Severe congenital neutropenia 7	immunology	AR	N	unk		recurrent infections	Y	complete blood count, bone marrow aspiration and biopsy	Y	Neutropenia does not respond to granulocyte-colony stimulating factor (G-CSF), but does respond to granulocyte-macrophage colony-stimulating factor (GM-CSF)	medication						
CYBA	CYBA associated chronic granulomatous disease	immunology	AR	N	0.675		recurrent infections	Y	dihydrorhodamine assay	Y	Antibacterial prophylaxis, antifungal prophalaxis, Interferon gamma, Hematopoietic stem cell transplantation (HSCT) - bone marrow transplant, ACTIMMUNE	medication HSCT						
CYBB	X-linked chronic granulomatous disease	immunology	XLR	N	0.675		recurrent infections	Y	dihydrorhodamine assay	Y	Antibacterial prophylaxis, antifungal prophalaxis, Interferon gamma, Hematopoietic stem cell transplantation (HSCT) - bone marrow transplant	medication HSCT						
CYBC1	CYBC1 associated chronic granulomatous disease	immunology	AR	N	0.675		recurrent infections	Y	dihydrorhodamine assay	Y	Bone marrow transplant (hematopoietic stem cell transplantation (HSCT))	HSCT						
NCF1	NCF1 associated chronic granulomatous disease	immunology	AR	N	0.675		recurrent infections	Y	dihydrorhodamine assay	Y	Antibacterial prophylaxis, antifungal prophalaxis, Interferon gamma, Hematopoietic stem cell transplantation (HSCT) - bone marrow transplant, ACTIMMUNE	medication HSCT						
NCF2	NCF2 associated chronic granulomatous disease	immunology	AR	N	0.675		recurrent infections	Y	dihydrorhodamine assay	Y	Antibacterial prophylaxis, antifungal prophalaxis, Interferon gamma, Hematopoietic stem cell transplantation (HSCT) - bone marrow transplant, ACTIMMUNE	medication HSCT						
NCF4	NCF4 associated chronic granulomatous disease	immunology	AR	N	0.675		recurrent infections	Y		Y	Antibacterial prophylaxis, antifungal prophalaxis, Interferon gamma, Hematopoietic stem cell transplantation (HSCT) - bone marrow transplant, ACTIMMUNE	medication HSCT						
DOCK2	DOCK2 defeciency	immunology	AR	N	1.72		recurrent infections	Y	T and B Lymphocyte and Natural Killer Cell Profile	Y	Hematopoietic stem cell transplantation (HSCT) - bone marrow transplant	HSCT						
DOCK8	DOCK8 deficiency	immunology	AR	N	1.72		recurrent infections	Y	serum IgE levels, T and B Lymphocyte and Natural Killer Cell Profile	Y	Hematopoietic stem cell transplantation (HSCT) - bone marrow transplant	HSCT						
ITGB2	Leukocyte adhesion deficiency, type I	immunology	AR	N	1		recurrent infections	Y	neutrophil chemotaxis	Y	Bone marrow transplant (hematopoietic stem cell transplantation (HSCT))	HSCT						
FERMT3	Leukocyte adhesion deficiency, type III	immunology	AR	N	1		recurrent infections	Y	neutrophil chemotaxis	Y	Bone marrow transplant (hematopoietic stem cell transplantation (HSCT))	HSCT						
IL10RB	Interleukin-10 deficiency	immunology	AR	N	ultrarare		recurrent infections	Y	flow cytometry	Y	Hematopoietic stem cell transplantation (HSCT) - bone marrow transplant	HSCT						
IL1RN	Interleukin 1 receptor antagonist deficiency	immunology	AR	N	ultrarare		hyperinflammation	N	N/A		anakinra, etanercept, methotrexate, corticosteroid	medication						
NLRC4	NLRC4 associated familial cold inflammatory syndrome	immunology	AD	N	ultrarare		hyperinflammation	Y	IL-18 serum levels	Y	tadekinig alfa (human recombinant interleukin-18 binding protein)	medication						
NLRP12	Familial cold autoinflammatory syndrome 2	immunology	AD	N	ultrarare		hyperinflammation	N	N/A		Corticosteroids, anakinra, rilonacept and canakinumab	medication						
PRF1	Familial hemophagocytic lymphohistiocytosis 2	immunology	AR	N	2		HLH	Y	natural killer cell activity, cytotoxic T lymphocyte activity	Y	Emapalumab, Hematopoietic stem cell transplantation (HSCT) - bone marrow transplant	medication HSCT						
UNC13D	Familial hemophagocytic lymphohistiocytosis 3	immunology	AR	N	2		HLH	Y	natural killer cell activity, cytotoxic T lymphocyte activity	Y	Emapalumab, Hematopoietic stem cell transplantation (HSCT) - bone marrow transplant	medication HSCT						
STX11	Familial hemophagocytic lymphohistiocytosis 4	immunology	AR	N	2		HLH	Y	natural killer cell activity, cytotoxic T lymphocyte activity	Y	Emapalumab, Hematopoietic stem cell transplantation (HSCT) - bone marrow transplant	medication HSCT						
STXBP2	Familial hemophagocytic lymphohistiocytosis 5	immunology	AR	N	2		HLH	Y	natural killer cell activity, cytotoxic T lymphocyte activity	Y	Emapalumab, Hematopoietic stem cell transplantation (HSCT) - bone marrow transplant	medication HSCT						
MVK	Hyper-IgD syndrome / mevalonate kinase deficiciency	immunology	AR	N	4		recurrent fevers	Y	serum immunoglobulin levels, urine organic acids	Y	anakinra, canakinumab, tocilizumab, etanercept	medication						

© 2023 Gold NB et al. *JAMA Network Open.*

Immunology (167 genes)

Gene	Disease name	System	Inheritance	On RUSP? (Y/N)	Prevalence - disease frequency per 100,000 (Rx genes, if listed)	Age of Onset	Disease symptoms	Orthogonal test? (Y/N)	If yes, orthogonal test	Is orthogonal test expected to be abnormal in infancy?	Intervention Considered (Free Text)	Category of Intervention	Age of Intervention Implementation	MD leading intervention	Comments	Link to ref 1	Link to ref 2	Link to ref 3
STAT3	Hyper-IgE recurrent infection syndrome	immunology	AD	N	2		recurrent infections	N			replacement immunoglobulin treatment, Bone marrow transplant (hematopoietic stem cell transplantation (HSCT))	transfusion HSCT						
PSTPIP1	PSTPIP1 associated inflammatory disease	immunology	AD	N	unk		immunedysregu lation	N	N/A		adalimumab and tacrolimus, NSAIDs, corticosteroids, Bone marrow transplant (hematopoietic stem cell transplantation (HSCT))	medication HSCT						
RAB27A	Griscelli syndrome, type 2	immunology	AR	N	2		HLH	Y	natural killer cell activity, cytotoxic T lymphocyte activity		Emapalumab, Hematopoietic stem cell transplantation (HSCT) - bone marrow transplant	medication HSCT						
RFXANK	MHC class II deficiency, complementation group B	immunology	AR	N	1.72		recurrent infections	Y	flow cytometric analysis of HLA-DR expression on monocytes and B cells		Hematopoietic stem cell transplantation (HSCT) - bone marrow transplant	HSCT						
RMRP	Cartilage-hair hypoplasia	immunology	AR	N	1.72		recurrent infections	N	N/A		Hematopoietic stem cell transplantation (HSCT) - bone marrow transplant	HSCT						
SMARCD2	Specific granule deficiency 2	immunology	AR	N	unk			Y	complete blood count, bone marrow aspiration and biopsy	Y	Bone marrow transplant (hematopoietic stem cell transplantation (HSCT))	HSCT						
TNFAIP3	TNFAIP3 associated autoinflammatory syndrome	immunology	AD	N	unk		recurrent fevers	N	N/A		Colchicine, glucocorticoid, mesalazine, cyclosporine, methotrexate, azathioprine, anakinra, rituximab, tocilizumab, infliximab	medication						
TNFRSF1A	Tumor necrosis factor receptor associated periodic syndrome	immunology	AD	N	0.056		recurrent fevers	N	N/A		NSAIDs, corticosteroids, Etanercept , anakinra, canakinumab, tocilizumab	medication						
USP18	Pseudo-TORCH syndrome 2	immunology	AR	N			immunedysregu lation	Y	Interferon signature		Ruxolitinib	medication						
WAS	WAS associated disorder	immunology	XLR	N	0.175		recurrent infections, malignancy, atopy	Y	PLT, natural killer c	Y	granulocyte colony-stimulating factor (G-CSF), Bone marrow transplant (hematopoietic stem cell transplantation (HSCT)), Antibiotic prophylaxis, immunoglobulin replacement, gene therapy	medication transfusion HSCT gene therapy						
WIPF1	Wiskott-Aldrich syndrome 2	immunology	AR	N	ultrarare		recurrent infections	N	PLT, natural killer c	Y	Bone marrow transplant (hematopoietic stem cell transplantation (HSCT))	HSCT						
ADA2	Vasculitis, autoinflammation, immunodeficiency, and hematologic defects syn	immunology	AR	N	0.5		recurrent infections, fevers, strokes	Y	plasma ADA2 enzyme activity	Y	TNF inhibitor, HSCT	medication, HSCT						
AK2	Reticular dysgenesis	immunology	AR	N	1.72		recurrent infections	Y	ANC, T and B Lymphocyte and Natural Killer Cell Profile	Y	Hematopoietic stem cell transplantation (HSCT) - bone marrow transplant	HSCT						
ACP5	Spondyloenchondrodysplasia with ACP5 immune dysregulation	immunology	AR	N	unk		recurrent infections, CNS involvement	N	N/A		Bone marrow transplant (hematopoietic stem cell transplantation (HSCT))	HSCT						
ARPC1B	Platelet abnormalities with eosinophilia and immune-mediated inflammatory	immunology	AR	N	unk		recurrent infections	Y	high IgA and IgE	Y	Bone marrow transplant (hematopoietic stem cell transplantation (HSCT))	HSCT						
C1NH	Hereditary angioedema	immunology	AD, AR	N	unk		angioedema	Y	C1-INH, C4									
CARD14	Pityriasis rubra pilaris	immunology	AD	N	unk		recurrent infections	N	N/A		ustekinumab	medication						
CARD9	Candidiasis, familial	immunology	AR	N	unk		recurrent infections		N/A									
CDKN1C	IMAGE syndrome	immunology	AD	N	unk			Y	serum cortisol and adrenocorticotrop ic hormone (ACTH) levels		Hydrocortisone, 9_-fludrohydrocortisone, oral supplements of sodium chloride	medication						
CFP	X-linked properdin deficiency	immunology	XLR	N	0.5		recurrent infections	Y	classical pathway and alternative pathway of the complement system functional activity tests		pneumococcal, meningococcal, haemophilus influenzae vaccines	vaccination						
CXCR4	WHIM syndrome	immunology	AD	N	0.023		recurrent infections, myelokathexis	Y	complete blood count, bone marrow aspiration and biopsy	Y	prophylactic antibiotics, granulocyte colony-stimulating factor (G-CSF), replacement immunoglobulin treatment, Plerixafor, Bone marrow transplant (hematopoietic stem cell transplantation (HSCT))	medication transfusion HSCT						
FOXP3	X-linked immunodysregulation, polyendocrinopathy, and enteropathy	immunology	XLR	N	unk		recurrent infections	Y	Flow Cytometry, T reg	Y	Rapamycin , hematopoietic stem cell transplantation (HSCT) - bone marrow transplant	medication HSCT						
IL36RN	Pustular psoriasis 14	immunology	AR	N	unk		immunedysregu lation, hyperinflammati on	N			ustekinumab, secukinumab, etanercept	medication						
IRAK4	IRAK4 deficiency	immunology	AR	N	unk		recurrent infections	Y	toll-like receptor function	y	Prophylactic antibiotic treatment, pneumococcal, meningococcal, haemophilus influenzae vaccines, and immunoglobulin replacement	medication vaccination transfusion						
KDSR	Erythrokeratodermia variabilis et progressiva 4	immunology	AR	N	unk		recurrent infections	N	N/A		isotretinoin	medication						
LIG4	LIG4 syndrome	immunology	AR	N	1.72		recurrent infections	Y	T and B Lymphocyte and Natural Killer Cell Profile	Y	Hematopoietic stem cell transplantation (HSCT) - bone marrow transplant	HSCT						
LPIN2	Majeed syndrome	immunology	AR	N	unk		recurrent infections	N	N/A		anakinra, canakinumab	medication						
MARS1	MARS1 associated interstitial lung and liver disease	immunology	AR	N	unk		recurrent infections	N	N/A		methionine supplementation, protein fortification, increased protein fortification during illness	diet medication						
MEFV	Familial Mediterranean fever	immunology	AR	N	unk		recurrent fevers	N	N/A		Colchicine, Canakinumab	medication						
MYD88	MYD88 deficiency	immunology	AR	N	unk		recurrent infections	Y	toll-like receptor function	Y	Prophylactic antibiotic treatment, pneumococcal, meningococcal, haemophilus influenzae vaccines, and immunoglobulin replacement	medication vaccination transfusion						

© 2023 Gold NB et al. *JAMA Network Open.*

Immunology (167 genes)

Gene	Disease name	System	Inheritance	On RUSP? (Y/N)	Prevalence - disease frequency per 100,000 (Rx genes, if listed)	Age of Onset	Disease symptoms	Orthogonal test? (Y/N)	If yes, orthogonal test	Is orthogonal test expected to be abnormal in infancy?	Intervention Considered (Free Text)	Category of Intervention	Age of Intervention Implementation	MD leading intervention	Comments	Link to ref 1	Link to ref 2	Link to ref 3
NIPAL4	Ichthyosis, congenital, autosomal recessive 6	immunology	AR	N	unk		recurrent infections	N	N/A		ustekinumab	medication						
NLRP3	Cryopyrin associated periodic fever syndrome	immunology	AD	N	0.19		hyperinflammati on	N	N/A		Corticosteroids, anakinra, rilonacept and canakinumab	medication						
NOD2	Blau syndrome	immunology	AD	N	1.72		hyperinflammati on	N	N/A		adalimumab, infliximab, golimumab, etanercept, methotrexate, prednisolone	medication						
OTULIN	OTULIN deficiency	immunology	AR	N	unk		hyperinfalmmati on	N	N/A		inflixiimab, anakinra, etanercept, corticosteroids	medication						
PARN	Dyskeratosis congenita, autosomal recessive 6	immunology	AR	N	unk		recurrent infections, lung fibrosis											
PAX1	Otofaciocervical syndrome 2	immunology	AR	N	1.72		recurrent infections, dysmorphism	Y	T and B Lymphocyte and Natural Killer Cell Profile	Y	thymus transplantation	OT						
PLCG2	Autoinflammation and PLCG2 associated antibody deficiency and immune dy	immunology	AD	N	0.02		recurrent infections, hyperinflammati on	Y	immunoglobulin levels, T and B Lymphocyte and Natural Killer Cell Profile	Y	replacement immunoglobulin treatment	transfusion						
PNP	Purine nucleoside phosphorylase deficiency	immunology	AR	N	1.72		recurrent infections	Y	T and B Lymphocyte and Natural Killer Cell Profile, erythrocyte purine nucleoside phosphorylase activity	Y	Hematopoietic stem cell transplantation (HSCT) - bone marrow transplant	HSCT						

© 2023 Gold NB et al. *JAMA Network Open.*

Metabolism (135 genes)

Gene	Disease name	System	Inheritance	On RUSP? (Y/N)	Prevalence - disease frequency per 100,000 (Rx genes, if listed)	Age of Onset	Disease symptoms	Orthogonal test? (Y/N)	If yes, orthogonal test	If yes, orthogonal test	Is orthogonal test expected to be abnormal in infancy?	Intervention Considered (Free Text)	Category of Intervention	If pharma, what company or companies are making treatment	Age of Intervention Implementation	MD leading intervention	Comments	Link to ref 1	Link to ref 2	Link to ref 3
ABCG5	Sitosterolemia 1	metabolism	AR	N	2	Childhood/Adult	artherosclerosis, xathomas, hypercholesterolemia, hemolytic anemmia, thrombocytopenia	Y	Plasma plant sterol concentrations	Plasma plant sterol concentrations	N	diet low in shellfish sterols and plant sterols, ezetimibe, cholestryramine	diet medication	generic	at diagnosis	cardio		https://www.ncbi.nlm.nih.gov/books/NBK131810/#stsl		
ABCG8	Sitosterolemia 2	metabolism	AR	N	2	Childhood/Adult	artherosclerosis, xathomas, hypercholesterolemia, hemolytic anemmia, thrombocytopenia	Y	Plasma plant sterol concentrations	Plasma plant sterol concentrations	N	diet low in shellfish sterols and plant sterols, ezetimibe, cholestryramine	diet medication	generic	at diagnosis	cardio		https://www.ncbi.nlm.nih.gov/books/NBK131810/#stsl		
G6PC	Glycogen storage disease Ia	metabolism	AR	N	0.04	Neonatal	hypoglycemia, lactic acidosis, hepatomegaly	Y	glucose, lactate, uric acid, free fatty acids	glucose, lactate, uric acid, free fatty acids	Y	corn starch, nighttime intragastric continuous glucose infusion, low carb/high protein diet	diet		at diagnosis	endo or metabolism				
SLC37A4	Glycogen storage disease Ib	metabolism	AR	N	0.04	Neonatal	hypoglycemia, lactic acidosis, hepatomegaly, IBD-like intestinal symptoms, neutropenia, other autoimmune symptoms	Y	glucose, lactate, uric acid, free fatty acids, complete blood count, bone marrow aspiration and biopsy	glucose, lactate, uric acid, free fatty acids, complete blood count, bone marrow aspiration and biopsy	Y	corn starch, nighttime intragastric continuous glucose infusion, allopurinol, statin, granulocyte-colony stimulating factor (G-CSF), immunomodulators, low carb/high protein diet	diet medication	generic	at diagnosis	endo or metabolism				
AGL	Glycogen storage disease III	metabolism	AR	N	1	Infancy	hypoglycemia, lactic acidosis, hepatomegaly	Y	glucose, liver biopsy with PAS-D	glucose, liver biopsy with PAS-D	N	high-protein diet with cornstarch supplementation	diet		at diagnosis	endo or metabolism				
PHKA2	Glycogen storage disease, type IXa	metabolism	XLR	N	0.75	Early childhood	hepatomegaly, liver dysfunction, growth restriction, hyperketotic hypoglycemia	Y	liver biopsy with glycogen accumulation, low PhK enzymology in liver, RBC, WBC	liver biopsy with glycogen accumulation, low PhK enzymology in liver, RBC, WBC	Y (enzyme)	high-protein diet with cornstarch supplementation	diet		diagnosis	endo or metabolism		https://www.ncbi.nlm.nih.gov/books/NBK55061/		
PHKB	Glycogen storage disease, type IXb	metabolism	AR	N	0.1	Early childhood	hepatomegaly, liver dysfunction, growth restriction, hyperketotic hypoglycemia, exercise intolerance, weakness, rhabdomyolsis	y	liver biopsy with glycogen accumulation, low PhK enzymology in liver, RBC, WBC	liver biopsy with glycogen accumulation, low PhK enzymology in liver, RBC, WBC	Y (enzyme)	high-protein diet with cornstarch supplementation	diet		diagnosis	endo or metabolism		https://www.ncbi.nlm.nih.gov/books/NBK55061/		
PHKG2	Glycogen storage disease, type IXc	metabolism	AR	N	0.1	Early childhood	hepatomegaly, liver dysfunction, growth restriction, hyperketotic hypoglycemia	y	liver biopsy with glycogen accumulation, low PhK enzymology in liver, RBC, WBC	liver biopsy with glycogen accumulation, low PhK enzymology in liver, RBC, WBC	Y (enzyme)	high-protein diet with cornstarch supplementation	diet		diagnosis	endo or metabolism		https://www.ncbi.nlm.nih.gov/books/NBK55061/		
PHKA1	Glycogen storage disease, type IXd	metabolism	XLR	N	<1	Childhood/Adult	exercise intolerance, weakness, rhabdomyolsis	Y	excessive amounts of subsarcolemmal glycogen; low PhK in muscle	excessive amounts of subsarcolemmal glycogen; low PhK in muscle	Y (enzyme)	none	n/a		n/a	n/a		https://www.ncbi.nlm.nih.gov/books/NBK55061/		
PYGL	Glycogen storage disease VI	metabolism	AR	N	1.36	Infancy/childhood	hepatomegaly & growth restriction +/- hypoglycemia	y	glucose, liver biopsy with PAS-D, liver enzymology	glucose, liver biopsy with PAS-D, liver enzymology	N	high-protein, low simple carbohydrate diet with cornstarch supplementation	diet		diagnosis	endo or metabolism		https://www.ncbi.nlm.nih.gov/books/NBK5941		

© 2023 Gold NB et al. *JAMA Network Open.*

Metabolism (135 genes)

Gene	Disease name	System	Inheritance	On RUSP? (Y/N)	Prevalence - disease frequency per 100,000 (Rx genes, if listed)	Age of Onset	Disease symptoms	Orthogonal test? (Y/N)	If yes, orthogonal test	If yes, orthogonal test	Is orthogonal test expected to be abnormal in infancy?	Intervention Considered (Free Text)	Category of Intervention	If pharma, what company or companies are making treatment	Age of Intervention Implementation	MD leading intervention	Comments	Link to ref 1	Link to ref 2	Link to ref 3
IDS	Mucopolysaccharidosis II	metabolism	XLR	N	0.795	Early childhood	coarse facial features, recurrent ear infections, sleep apnea, carpal tunnel syndrome, intellectual disability, dysostosis multiplex, hepato/splenomegaly, inguinal hernia, chronic diarrhea, behavioral problems, hydrocephalus	Y	iduronate 2-sulfatase (I2S) enzyme activity in white cells, fibroblasts, or plasma, urine glucosaminoglycans	iduronate 2-sulfatase (I2S) enzyme activity in white cells, fibroblasts, or plasma, urine glucosaminoglycans	Y	idursulfase Elaprase enzyme replacement, Bone marrow transplantation Hematopoietic Stem Cell Transplantation (HSCT)	ERT HSCT	Takeda	before 1 year	metabolism				
SGSH	Mucopolysaccharidosis type IIIA (Sanfilippo A)	metabolism	AR	N	1	1-3 years old	developmental delay, behavior problems, sleep disorder, regression, seizures, +/- subtle coarseness, ear infections, hearing loss, hernias	Y	serum or plasma enzyme activity, urine glucosaminoglycans	serum or plasma enzyme activity, urine glucosaminoglycans	Y	none	n/a	n/a	n/a	n/a				
NAGLU	Mucopolysaccharidosis type IIIB	metabolism	AR	N	0.5	1-3 years old	developmental delay, behavior problems, sleep disorder, regression, seizures, +/- subtle coarseness, ear infections, hearing loss, hernias	Y	Serum or plasma N-acetyl-alpha-D-glucosaminidase enzyme activity, urine glucosaminoglycans	Serum or plasma N-acetyl-alpha-D-glucosaminidase enzyme activity, urine glucosaminoglycans	Y	intraventricular Tralesinidase alfa (BMN 250) enzyme replacement	ERT	Allievex	study was for 1-11 yo	metabolism		https://clinicaltrials.gov/ct2/show/NCT02754076?term=Allievex&draw=2&rank=2		
HGSNAT	Mucopolysaccharidosis type IIIC (Sanfilippo C)	metabolism	AR	N	0.07	1-3 years old	developmental delay, behavior problems, sleep disorder, regression, seizures, +/- subtle coarseness, ear infections, hearing loss, hernias	Y	serum or plasma enzyme activity, urine glucosaminoglycans	serum or plasma enzyme activity, urine glucosaminoglycans	Y	none	n/a	n/a	n/a	n/a				
GALNS	Mucopolysaccharidosis IVA	metabolism	AR	N	0.335	1-3 years old	coarse facial features, skeletal dysplasia, short stature, kyphoscoliosis, joint hypermobility, airway obstruction, atlantoaxial instability	Y	leukocyte N-acetylgalactosamine 6-sulfatase enzyme activity, urine GAGs	leukocyte N-acetylgalactosamine 6-sulfatase enzyme activity, urine GAGs	Y	elosulfase alfa enzyme replacement	ERT	biomarin	at diagnosis	metabolism	late-onset forms exist	https://www.ncbi.nlm.nih.gov/books/NBK148668/		
ARSB	Mucopolysaccharidosis type VI	metabolism	AR	N	0.285	2-3 years old	coarse facial features, recurrent ear infections, sleep apnea, carpal tunnel syndrome, dysostosis multiplex, hepato/splenomegaly, inguinal hernia, hort stature	Y	leukocyte arylsulfatase B enzyme activity	leukocyte arylsulfatase B enzyme activity	Y	galsulfase enzyme replacement, HSCT	ERT HSCT	Biomarin	ASAP (kids who get neonatal or presymptomatic ERT do better)	metabolism	late-onset forms exist w onset as late as 20-30	https://www.ncbi.nlm.nih.gov/pmc/articles/PMC2873242/		

© 2023 Gold NB et al. *JAMA Network Open.*

Metabolism (135 genes)

Gene	Disease name	System	Inheritance	On RUSP? (Y/N)	Prevalence - disease frequency per 100,000 (Rx genes, if listed)	Age of Onset	Disease symptoms	Orthogonal test? (Y/N)	If yes, orthogonal test	If yes, orthogonal test	Is orthogonal test expected to be abnormal in infancy?	Intervention Considered (Free Text)	Category of Intervention	If pharma, what company or companies are making treatment	Age of Intervention Implementation	MD leading intervention	Comments	Link to ref 1	Link to ref 2	Link to ref 3
GUSB	Mucopolysaccharidosis type VII	metabolism	AR	N	0.13	neonatal form; infantile form; adolescent form	coarse facila features, corneal clouding, frequent ear infections, hearing loss, recurrent respiratory infections, sleep apnea, obstructive/restrictive lung disease, cardiomyopathy, cardiac valvulopathy, hydrops, dysostosis muyltiplex, joint contractures, scoliosis, kyphosis , intellectual disability, short stature	Y	Leukocyte beta-glucuronidase enzyme activity	Leukocyte beta-glucuronidase enzyme activity	Y	Vestronidase alfa enzyme replacement, HSCT	ERT HSCT	ultragenyx	at diagnosis	metabolism		https://www.ncbi.nlm.nih.gov/pmc/articles/PMC4893087/		
GNPTA	I-Cell Disease	metabolism	AR	N	0.25	Birth	growth impairment, joint contractures, coarse facial features, recurrent ear infections, airway and lung parenchymal disease, respiratory insufficiency, pulmonary hypertension, cardiac valvulopathy, umbilical hernia, bone deformation, kyphosis, clubfoot, hip dislocation, joint contractures	Y	urine glycosaminoglycans, elevated plasma lysosomal enzymes (multiple)	urine glycosaminoglycans, elevated plasma lysosomal enzymes (multiple)	Y	occupational therapy, dental surveillance, myringotomy tubes, airway precautions	supportive surveillance surgery		diagnosis	metabolism or complex care peds	allelic form ML III not addressed in comments	https://www.ncbi.nlm.nih.gov/books/NBK1828/		
GALC	Krabbe disease	metabolism	AR	N	0.4	Infantile onset; late-onset (1-3 years); (2-4 years); adolescence; adulthood	peripheral neuropathy, regression, leukodystrophy, seizures	Y	Leukocyte enzyme activity, elevated psychosine	Leukocyte enzyme activity, elevated psychosine	enzyme Y; psychosine only in infantile forms	HSCT	HSCT		first 7 weeks of life	hematology	HSCT later in life may be reasonable for later-onset Krabbe disease, but no clear guidelines	https://www.ncbi.nlm.nih.gov/books/NBK1238		
SMPD1	Niemann-Pick disease, type A and type B	metabolism	AR	N	0.4	Early infantile (3 months)- childhood	hepatosplenomegaly, neurologic deterioration, cherry-red spot, interstitial lung disease	Y	Leukocyte acid sphingomyelinase enzyme activity	Leukocyte acid sphingomyelinase enzyme activity	Y	HSCT, recombinant human acid sphingomyelinase enzyme replacement therapy, OLT (late-onset only)	ERT HSCT OT	Sanofi	3-18 for clinical trial	metabolism	no treatment guidelines are definitive	https://www.ncbi.nlm.nih.gov/books/NBK1370	https://clinicaltrials.gov/ct2/show/NCT04877132	
NPC1	Niemann-Pick disease, type C, NPC1	metabolism	AR	N	0.7	Early infantile (<2), late infantile (2-6), juvenile (6-15), adult (>15)	hepatosplenomegaly, jaundice, pulmonay infiltrates, ataxia, seizures, dementia, neurodegeneration	Y	Oxysterol analysis, filippin staining	Oxysterol analysis, filippin staining	unknown	Miglustat	medication	Janssen	symptom onset	metabolism		https://www.ncbi.nlm.nih.gov/books/NBK1296/		
NPC2	Niemann-Pick disease, type C, NPC2	metabolism	AR	N	0.03	Early infantile (<2), late infantile (2-6), juvenile (6-15), adult (>15)	hepatosplenomegaly, jaundice, pulmonay infiltrates, ataxia, seizures, dementia, neurodegeneration	Y	Oxysterol analysis, filippin staining	Oxysterol analysis, filippin staining	unknown	Miglustat	medication	Janssen	symptom onset	metabolism		https://www.ncbi.nlm.nih.gov/books/NBK1296/		
HEXA	Tay-Sachs disease	metabolism	AR	N	0.3	Classic (3-6 mo); subacute (2), late onset (teen-young adult)	visual loss, cherry red spot, seizures, neurodeterioration	Y	Hex A enzyme	Hex A enzyme	Y	supportive	supportive		diagnosis	metabolism or complex care peds		https://www.ncbi.nlm.nih.gov/books/NBK1218		
HEXB	Sandhoff disease, infantile, juvenile, and adult forms	metabolism	AR	N	0.1	Infantile (<6mo), juvenile (2-5y), late (teens-young adult)	neurologic regression, seizures	Y	Hex B Enzyme	Hex B Enzyme	Y	supportive	supportive							

© 2023 Gold NB et al. *JAMA Network Open.*

Metabolism (135 genes)

Gene	Disease name	System	Inheritance	On RUSP? (Y/N)	Prevalence - disease frequency per 100,000 (Rx genes, if listed)	Age of Onset	Disease symptoms	Orthogonal test? (Y/N)	If yes, orthogonal test	If yes, orthogonal test	Is orthogonal test expected to be abnormal in infancy?	Intervention Considered (Free Text)	Category of Intervention	If pharma, what company or companies are making treatment	Age of Intervention Implementation	MD leading intervention	Comments	Link to ref 1	Link to ref 2	Link to ref 3
FUCA1	Fucosidosis	metabolism	AR	N	<0.5	0-5 years old	coarse facial features, growth retardation, recurrent URI, dysostosis multiplex, angiokeratoma, neurodegeneration	Y	Fucosidase activity in serum or plasma	Fucosidase activity in serum or plasma	Y	supportive or Hematopoietic Stem Cell Transplantation (HSCT)	supportive HCST	n/a	presymptomatic	hematology		https://www.ncbi.nlm.nih.gov/pmc/articles/PMC7700486/		
GBA	Gaucher disease, type I	metabolism	AR	N	1.94	10-20 years old	bone marrow failure, hepatosplenomegaly, bone crisis	Y	Glucocerebrosidase enzyme activity in leukocytes, TRAP, ACE	Glucocerebrosidase enzyme activity in leukocytes, TRAP, ACE	Y (enzyme)	Enzyme replacement therapy (ERT): imiglucerase (Cerezyme); velalgl -ucerase alfa (VPRIV); & taliglucerase alfa (Elelyso) or substrate reduction therapy (SRT) miglustat or eliglustat	medication ERT	sanofi, shire, pfizer, actelion, genzyme	symptom onset (typically adolescent-adulthood)	metabolism		https://www.ncbi.nlm.nih.gov/books/NBK1269/	https://www.ncbi.nlm.nih.gov/pmc/articles/PMC5343975/	
GLA	Fabry disease	metabolism	XLR	N	2 (classic form); 11.8 (including atypical forms)	Classic (4-8); atypical (>25 years)	angiokeratoma, acroparesthesias, corenal opacity, cardiomyopathy, cardiac ischemia, stroke, ESRD, proteinuria	Y	Serum globotriaosylsphingosine, a-Gal A enzyme activity	Serum globotriaosylsphingosine, a-Gal A enzyme activity	Y (enzyme)	Agalsidase alfa enzyme replacement	ERT	genzyme	at diagnosis in males and if disease manifestations in females	metabolism		https://www.ncbi.nlm.nih.gov/pmc/articles/PMC7507033/	https://www.ncbi.nlm.nih.gov/books/NBK1292/	
PPT1	Ceroid lipofuscinosis, neuronal, 1	metabolism	AR	N	0.52	6-18 months old	developmental regression, epilepsy, ataxia, dystonia, choreoathetosis, myclonus, progressive vision los	Y	PPT1 enzyme fibroblasts, WBC, amnio, DBS, CVS	PPT1 enzyme fibroblasts, WBC, amnio, DBS, CVS	Y	none						https://pubmed.ncbi.nlm.nih.gov/35628533/		
TPP1	Neuronal ceroid lipofuscinosis 2	metabolism	AR	N	0.465	2-4 years old	delayed development & developmental regression, epilepsy, ataxia, choreathetosis, progressive vision loss	Y	Leukocyte tripeptidyl peptidase enzyme activity	Leukocyte tripeptidyl peptidase enzyme activity	Y	Cerliponase alfa enzyme replacement	ERT	Bio Marin	3	metabolism		https://www.ncbi.nlm.nih.gov/books/NBK544807/	https://pubmed.ncbi.nlm.nih.gov/35628533/	
MFSD8	Ceroid lipofuscinosis, neuronal, 7	metabolism	AR	N	<0.465	1.5-6	cognitive and motor decline, ataxia, myoclonus, epilepsy, progressive vision loss	Y	skin biopsy for storage material	skin biopsy for storage material	N	none						https://pubmed.ncbi.nlm.nih.gov/35628533/		
COQ2	Primary coenzyme Q10 deficiency 1	metabolism	AR	N	<1	Iinfancy; adult-onset phenotype exists	nephrotic syndrome, hypertrophic cardiomyopathy, hearing loss, encephalopathy, seizures, myopathy	Y	leukocyte or muscle CoQ10	leukocyte or muscle CoQ10	Y	CoQ10 supplementation	medication	generic	diagnosis	metabolism		https://www.ncbi.nlm.nih.gov/books/NBK410087		
PDSS1	Primary coenzyme Q10 deficiency 2	metabolism	AR	N	<1	Infancy	optic atrophy, encephalopathy, peripheral neuropathy	Y	leukocyte or muscle CoQ10	leukocyte or muscle CoQ10	Y	CoQ10 supplementation	medication	generic	diagnosis	metabolism		https://www.ncbi.nlm.nih.gov/books/NBK410087		
PDSS2	Primary coenzyme Q10 deficiency 3	metabolism	AR	N	<1	Infancy	nephrotic syndrome, retinopathy, hearing loss, leigh sydrome, ataxia	Y	leukocyte or muscle CoQ10	leukocyte or muscle CoQ10	Y	CoQ10 supplementation	medication	generic	diagnosis	metabolism		https://www.ncbi.nlm.nih.gov/books/NBK410087		
COQ8A	Primary coenzyme Q10 deficiency 4	metabolism	AR	N	<1	Infancy	encephalopathy, ataxia, dystonia, seizures, exercise intolerance	Y	leukocyte or muscle CoQ10	leukocyte or muscle CoQ10	Y	CoQ10 supplementation	medication	generic	diagnosis	metabolism		https://www.ncbi.nlm.nih.gov/books/NBK410087		
COQ9	Primary coenzyme Q10 deficiency 5	metabolism	AR	N	<1	Infancy	renal tubulopathy, hypertrophic cardiomyopathy, encephalopathy, myopathy	Y	leukocyte or muscle CoQ10	leukocyte or muscle CoQ10	Y	CoQ10 supplementation	medication	generic	diagnosis	metabolism		https://www.ncbi.nlm.nih.gov/books/NBK410087		

© 2023 Gold NB et al. *JAMA Network Open.*

Metabolism (135 genes)

Gene	Disease name	System	Inheritance	On RUSP? (Y/N)	Prevalence - disease frequency per 100,000 (Rx genes, if listed)	Age of Onset	Disease symptoms	Orthogonal test? (Y/N)	If yes, orthogonal test	If yes, orthogonal test	Is orthogonal test expected to be abnormal in infancy?	Intervention Considered (Free Text)	Category of Intervention	If pharma, what company or companies are making treatment	Age of Intervention Implementation	MD leading intervention	Comments	Link to ref 1	Link to ref 2	Link to ref 3
COQ6	Primary coenzyme Q10 deficiency 6	metabolism	AR	N	<1	Infancy	nephrotic syndrome, hearing loss, encephalopathy, seizures	Y	leukocyte or muscle CoQ10	leukocyte or muscle CoQ10	Y	CoQ10 supplementation	medication	generic	diagnosis	metabolism		https://www.ncbi.nlm.nih.gov/books/NBK410087		
COQ4	Primary coenzyme Q10 deficiency 7	metabolism	AR	N	<1	Infancy	heart failure; hypertrophic cardiomyopathy, encephalopathy, seizures, myopathy, respiratory insufficiency, cerebellar hypoplasia, neuro deterioration	Y	leukocyte or muscle CoQ10	leukocyte or muscle CoQ10	Y	CoQ10 supplementation	medication	generic	diagnosis	metabolism		https://www.ncbi.nlm.nih.gov/books/NBK410087		
COQ7	Primary coenzyme Q10 deficiency 8	metabolism	AR	N	<1	Infancy	encephalopathy, intellectual disability, peripheral neuropathy	Y	leukocyte or muscle CoQ10	leukocyte or muscle CoQ10	Y	CoQ10 supplementation	medication	generic	diagnosis	metabolism		https://www.ncbi.nlm.nih.gov/books/NBK410087		
COQ5	Coenzyme Q5 methyltransferase deficiency	metabolism	AR	N	<1	Early chidhood	ataxia, encephalopathy, seizures, developmental delay, short stature, myoclonus	Y	leukocyte or muscle CoQ10	leukocyte or muscle CoQ10	Y	CoQ10 supplementation	medication	generic	diagnosis	metabolism		https://pubmed.ncbi.nlm.nih.gov/29044765/		
MT-CO1	MELAS (Myopathy, Encephalopathy, Lactic Acidosis, and Stroke-like episodes)	metabolism	Mt	N	<1	adolesence-early adulthoor	encephalopathy, hearing loss, ataxia, epilepsy, intellectual disability, myoglobinuria	Y	complex IV deficiency in muscle	complex IV deficiency in muscle	N	antioxidants, creatine	medication	generic	diagnosis	metabolism		https://doi.org/10.1016/j.mito.2014.06.003		
MT-CO3	MELAS (Myopathy, Encephalopathy, Lactic Acidosis, and Stroke-like episodes)	metabolism	Mt	N	<0.02	2-40 (most 2-20)	seizures, headaches, stroke-like episodes, weakness, vomiting, short stature	Y	complex IV deficiency in muscle	complex IV deficiency in muscle	N	arginine, citrulline, pyruvate	medication	generic	diagnosis	metabolism		https://www.ncbi.nlm.nih.gov/books/NBK1233/		
MT-CO2	MELAS (Myopathy, Encephalopathy, Lactic Acidosis, and Stroke-like episodes)	metabolism	Mt	N	<0.02	2-40 (most 2-20)	seizures, headaches, stroke-like episodes, weakness, vomiting, short stature	Y	Complex IV deficiency in muscle	Complex IV deficiency in muscle	N	arginine, citrulline, pyruvate	medication	generic	diagnosis	metabolism		https://www.ncbi.nlm.nih.gov/books/NBK1233/		
MT-ND1	MELAS (Myopathy, Encephalopathy, Lactic Acidosis, and Stroke-like episodes)	metabolism	Mt	N	<0.02	2-40 (most 2-20)	seizures, headaches, stroke-like episodes, weakness, vomiting, short stature	Y	Complex I deficiency in muscle	Complex I deficiency in muscle	N	arginine, citrulline, pyruvate	medication	generic	diagnosis	metabolism		https://www.ncbi.nlm.nih.gov/books/NBK1233/		
MT-ND4	MELAS (Myopathy, Encephalopathy, Lactic Acidosis, and Stroke-like episodes)	metabolism	Mt	N	<0.02	2-40 (most 2-20)	seizures, headaches, stroke-like episodes, weakness, vomiting, short stature	Y	Complex I deficiency in muscle	Complex I deficiency in muscle	N	arginine, citrulline, pyruvate	medication	generic	diagnosis	metabolism		https://www.ncbi.nlm.nih.gov/books/NBK1233/		
MT-ND5	MELAS (Myopathy, Encephalopathy, Lactic Acidosis, and Stroke-like episodes)	metabolism	Mt	N	0.02	2-40 (most 2-20)	seizures, headaches, stroke-like episodes, weakness, vomiting, short stature	Y	Complex I deficiency in muscle	Complex I deficiency in muscle	N	arginine, citrulline, pyruvate	medication	generic	diagnosis	metabolism		https://www.ncbi.nlm.nih.gov/books/NBK1233/		
MT-ND6	MELAS (Myopathy, Encephalopathy, Lactic Acidosis, and Stroke-like episodes)	metabolism	Mt	N	<0.02	2-40 (most 2-20)	seizures, headaches, stroke-like episodes, weakness, vomiting, short stature	Y	Complex I deficiency in muscle	Complex I deficiency in muscle	N	arginine, citrulline, pyruvate	medication	generic	diagnosis	metabolism		https://www.ncbi.nlm.nih.gov/books/NBK1233/		
MT-TF	MELAS (Myopathy, Encephalopathy, Lactic Acidosis, and Stroke-like episodes)	metabolism	Mt	N	<0.02	2-40 (most 2-20)	seizures, headaches, stroke-like episodes, weakness, vomiting, short stature	Y	Muscle biopsy may show multi-complex dysfunction; lactate; elevated proline, alanine	Muscle biopsy may show multi-complex dysfunction; lactate; elevated proline, alanine	N	arginine, citrulline, pyruvate	medication	generic	diagnosis	metabolism		https://www.ncbi.nlm.nih.gov/books/NBK1233/		

© 2023 Gold NB et al. *JAMA Network Open.*

Metabolism (135 genes)

Gene	Disease name	System	Inheritance	On RUSP? (Y/N)	Prevalence - disease frequency per 100,000 (Rx genes, if listed)	Age of Onset	Disease symptoms	Orthogonal test? (Y/N)	If yes, orthogonal test	If yes, orthogonal test	Is orthogonal test expected to be abnormal in infancy?	Intervention Considered (Free Text)	Category of Intervention	If pharma, what company or companies are making treatment	Age of Intervention Implementation	MD leading intervention	Comments	Link to ref 1	Link to ref 2	Link to ref 3
MT-TH	MELAS (Myopathy, Encephalopathy, Lactic Acidosis, and Stroke-like episodes)	metabolism	Mt	N	<0.02	2-40 (most 2-20)	seizures, headaches, stroke-like episodes, weakness, vomiting, short stature	Y	Muscle biopsy may show multi-complex dysfunction; lactate; elevated proline, alanine	Muscle biopsy may show multi-complex dysfunction; lactate; elevated proline, alanine	N	arginine, citrulline, pyruvate	medication	generic	diagnosis	metabolism		https://www.ncbi.nlm.nih.gov/books/NBK1233/		
MT-TL1	MELAS (Myopathy, Encephalopathy, Lactic Acidosis, and Stroke-like episodes)	metabolism	Mt	N	0.16	2-40 (most 2-20)	seizures, headaches, stroke-like episodes, weakness, vomiting, short stature	Y	Muscle biopsy may show multi-complex dysfunction; lactate; elevated proline, alanine	Muscle biopsy may show multi-complex dysfunction; lactate; elevated proline, alanine	N	arginine, citrulline, taurine, pyruvate	medication	generic	diagnosis	metabolism		https://www.ncbi.nlm.nih.gov/books/NBK1233/		
MT-TQ	MELAS (Myopathy, Encephalopathy, Lactic Acidosis, and Stroke-like episodes)	metabolism	Mt	N	<0.02	2-40 (most 2-20)	seizures, headaches, stroke-like episodes, weakness, vomiting, short stature	Y	Muscle biopsy may show multi-complex dysfunction; lactate; elevated proline, alanine	Muscle biopsy may show multi-complex dysfunction; lactate; elevated proline, alanine	N	arginine, citrulline, pyruvate	medication	generic	diagnosis	metabolism		https://www.ncbi.nlm.nih.gov/books/NBK1233/		
MT-TS1	MELAS (Myopathy, Encephalopathy, Lactic Acidosis, and Stroke-like episodes)	metabolism	Mt	N	<0.02	2-40 (most 2-20)	seizures, headaches, stroke-like episodes, weakness, vomiting, short stature	Y	Muscle biopsy may show multi-complex dysfunction; lactate; elevated proline, alanine	Muscle biopsy may show multi-complex dysfunction; lactate; elevated proline, alanine	N	arginine, citrulline, pyruvate	medication	generic	diagnosis	metabolism		https://www.ncbi.nlm.nih.gov/books/NBK1233/		
MT-TS2	MELAS (Myopathy, Encephalopathy, Lactic Acidosis, and Stroke-like episodes)	metabolism	Mt	N	<0.02	2-40 (most 2-20)	seizures, headaches, stroke-like episodes, weakness, vomiting, short stature	Y	Muscle biopsy may show multi-complex dysfunction; lactate; elevated proline, alanine	Muscle biopsy may show multi-complex dysfunction; lactate; elevated proline, alanine	N	arginine, citrulline, pyruvate	medication	generic	diagnosis	metabolism		https://www.ncbi.nlm.nih.gov/books/NBK1233/		
MT-TW	MELAS (Myopathy, Encephalopathy, Lactic Acidosis, and Stroke-like episodes)	metabolism	Mt	N	<0.02	2-40 (most 2-20)	seizures, headaches, stroke-like episodes, weakness, vomiting, short stature	Y	Muscle biopsy may show multi-complex dysfunction; lactate; elevated proline, alanine	Muscle biopsy may show multi-complex dysfunction; lactate; elevated proline, alanine	N	arginine, citrulline, pyruvate	medication	generic	diagnosis	metabolism		https://www.ncbi.nlm.nih.gov/books/NBK1233/		
ACAD9	Mitochondrial complex I deficiency nuclear type 20	metabolism	AR	N	unk	~35% neonatal; ~25% infantile; 25% early childhood; 3% adolescent	cardiomyopathy, myopathy, intellectual disability, developmental delay	Y	acylcarnitine profile	acylcarnitine profile	Y	riboflavin, sodium pyruvate, beta-blocker, coenzyme Q10, carnitine	medication	generic	diagnosis	metabolism, cardiology		https://ojrd.biomedcentral.com/articles/10.1186/s13023-018-0784-8		
ACAT1	Mitochondrial acetoacetyl-CoA thiolase deficiency	metabolism	AR	N	0.415	Infancy-early childhood	intermittent ketoacidosis	Y	urine organic acids, plasma acylcarnitine profile	urine organic acids, plasma acylcarnitine profile	Y	avoid fasting, carnitine, riboflavin, protein restricted diet	diet medication		diagnosis	metabolism		https://www.sciencedirect.com/science/article/pii/S1096719217300884?via%3Dihub		
ECHS1	Mitochondrial short-chain enoyl-CoA hydratase-1 deficiency	metabolism	AR	N	unk	Prenatal form; infantile form; childhood-adolescent form	signal abnormalities in basal ganglia, developmental delay, hypotonia, dystonia, epilepsy, encephalopathy, ataxia, choreoathetosis, cardiomyopathy, optic atrophy, hearing loss, apnea, liver steatosis, lactic acidemia	Y	acylcarnitine profile, urine organic acids, PDC enzymology	acylcarnitine profile, urine organic acids, PDC enzymology	Y	valine restricted diet, ketogenic diet	diet		diagnosis	metabolism		https://www.ncbi.nlm.nih.gov/books/NBK542806/		
ETHE1	Mitochondrial sulfur dioxygenase deficiency	metabolism	AR	N	unk	Infancy	developmental delay, hypotonia, epilepsy, purpura, chronic diarrhea	Y	organic acids, acylcarnitine, lactate, thiosulphate (plasma)	organic acids, acylcarnitine, lactate, thiosulphate (plasma)	Y	n-acetylcysteine, metronidazole, low sulfur diet	diet medication	generic	diagnosis	metabolism		https://www.ncbi.nlm.nih.gov/books/NBK453432/		
TK2	Thymidine kinase deficiency	metabolism	AR	N	unk	Infantile (<2) >> juvenile (2-18) = adult (>18)	hypotonia, respiratory difficulties, motor regression, hyporeflexia, lactic acidosis, seizures	Y	muscle biopsy shows ragged red fibers, low or no CIV activity	muscle biopsy shows ragged red fibers, low or no CIV activity	N	deoxycytidine (dC) & deoxythymidine (dT)	medication	https://clinicaltrials.gov/ct2/show/NCT03639701	diagnosis	neurology		https://clinicaltrials.gov/ct2/show/NCT03639701	https://www.ncbi.nlm.nih.gov/books/NBK114628/	
DLAT	Pyruvate dehydrogenase deficiency	metabolism	AR	N	0.02	Infancy	episodic dystonia, globus pallidus lesions	Y	PDH enzyme activity in blood or skin; lactate/pyruvate in blood or CSF	PDH enzyme activity in blood or skin; lactate/pyruvate in blood or CSF	Y	ketogenic diet, thiamine, lipoic acid	diet medication	generic	diagnosis	metabolism, neurology		https://www.ncbi.nlm.nih.gov/books/NBK571223/		

© 2023 Gold NB et al. *JAMA Network Open.*

Metabolism (135 genes)

Gene	Disease name	System	Inheritance	On RUSP? (Y/N)	Prevalence - disease frequency per 100,000 (Rx genes, if listed)	Age of Onset	Disease symptoms	Orthogonal test? (Y/N)	If yes, orthogonal test	If yes, orthogonal test	Is orthogonal test expected to be abnormal in infancy?	Intervention Considered (Free Text)	Category of Intervention	If pharma, what company or companies are making treatment	Age of Intervention Implementation	MD leading intervention	Comments	Link to ref 1	Link to ref 2	Link to ref 3
PDHA1	Pyruvate dehydrogenase deficiency	metabolism	XLR	N	1.52	Infancy	epilepsy, encephalopathy, lacitic acidosis, Leigh syndrome	Y	PDH enzyme activity in blood or skin; lactate/pyruvate in blood or CSF	PDH enzyme activity in blood or skin; lactate/pyruvate in blood or CSF	Y	ketogenic diet, thiamine	diet medication	generic	diagnosis	metabolism, neurology		https://www.ncbi.nlm.nih.gov/books/NBK571223/		
PDHB	Pyruvate dehydrogenase deficiency	metabolism	AR	N	0.08	Neonatal	microcephaly, structural brain anomalies, leigh syndrome, neonatal lactic acidosis, IUGR	Y	PDH enzyme activity in blood or skin; lactate/pyruvate in blood or CSF	PDH enzyme activity in blood or skin; lactate/pyruvate in blood or CSF	Y	ketogenic diet, thiamine	diet medication	generic	diagnosis	metabolism, neurology		https://www.ncbi.nlm.nih.gov/books/NBK571223/		
PDHX	Pyruvate dehydrogenase deficiency	metabolism	AR	N	0.14	Neonatal	lactic acidosis, encephalopathy	Y	PDH enzyme activity in blood or skin; lactate/pyruvate in blood or CSF	PDH enzyme activity in blood or skin; lactate/pyruvate in blood or CSF	Y	ketogenic diet, thiamine	diet medication	generic	diagnosis	metabolism, neurology		https://www.ncbi.nlm.nih.gov/books/NBK571223/		
PDP1	Pyruvate dehydrogenase phosphatase deficiency	metabolism	AR	N	0.02	Neonatal	lactic acidosis, cardiomyopathy	Y	PDH enzyme activity in blood or skin; lactate/pyruvate in blood or CSF	PDH enzyme activity in blood or skin; lactate/pyruvate in blood or CSF	Y	ketogenic diet, thiamine	diet medication	generic	diagnosis	metabolism, neurology		https://www.ncbi.nlm.nih.gov/books/NBK571223/		
PKLR	Pyruvate kinase deficiency	metabolism	AR	N	0.8	Neonatal, juvenile or adult	hemolytic anemia	Y	omplete blood count and red cell pyruvate kinase activity	omplete blood count and red cell pyruvate kinase activity	Y	Mitapivat, red cell transfusion, folic acid	medication transfusion	Agios	folic acid in childhood; mitapivat in adulthood	hematology		https://www.ncbi.nlm.nih.gov/books/NBK560581/		
TRPM6	TRPM6 associated hypomagnesemia	metabolism	AR	N	unk	Neonatal	hypomagnesemia, hypocalcemia	Y	serum magnesium and calcium levels	serum magnesium and calcium levels	Y	magnesium	medication	generic	diagnosis	gastroenterology		https://www-nature-com.proxy.library.upenn.edu/articles/ng901z		
FXYD2	Hypomagnesemia, type 2	metabolism	AD	N	unk	Adulthood > childhood	hypomagnesemia, hypermagnesuria	Y	serum magnesium & fractional excretion of magnesium & calcium	serum magnesium & fractional excretion of magnesium & calcium	unk	magnesium	medication	generic	diagnosis	unk		https://www.zora.uzh.ch/id/eprint/121654/1/gfv014.pdf		
MPI	Congenital disorder of glycosylation, type Ib	metabolism	AR	N	<1	Infancy	diarrhea, hepatomegaly, hypoglycemia, protein-losing enteropathy	Y	transferrin profiling, N-glycan profiling	transferrin profiling, N-glycan profiling	Y	mannose, liver transplant	medication OT	generic	diagnosis (mannose)	metabolism		https://www.ncbi.nlm.nih.gov/pmc/articles/PMC8720509/	https://pubmed.ncbi.nlm.nih.gov/33098580/	
PGM1	Congenital disorder of glycosylation, type It	metabolism	AR	N	<1	Neonatal	cleft palate, hypoglycemia, hepatitis, myopathy, structural cardiac defects, neurologic involvement	Y	transferrin profiling, N-glycan profiling	transferrin profiling, N-glycan profiling	Y	D-galactose	medication	generic	diagnosis	metabolism		https://www.ncbi.nlm.nih.gov/pmc/articles/PMC8720509/	https://www.sciencedirect.com/science/article/pii/S1096719220301852?via%3Dihub	
SLC35A2	Congenital disorder of glycosylation, type IIm	metabolism	XLD	N	<1	Infancy	epileptic encephalopathy, retinopathy, nystagmus, strabismus, failure to thrive, liver dysfunction, hepatosplemegaly, nephrotic syndrome, rhizomelia, craniosynostosis, scoliosis	Y	N-glycan profiling, carbohydrate deficienct transferrin profile	N-glycan profiling, carbohydrate deficienct transferrin profile	Y	D-galactose	medication	generic	diagnosis	metabolism		https://www.ncbi.nlm.nih.gov/pmc/articles/PMC8720509/	https://www.ncbi.nlm.nih.gov/pmc/articles/PMC7275909/	
SLC39A8	Congenital disorder of glycosylation, type IIn	metabolism	AR	N	<1	Infancy	Leigh like changes on MRI, cerebral and cerebellar atrophy, developmental delay	Y	Mn level, N-glycan, carbohydrate deficient transferin	Mn level, N-glycan, carbohydrate deficient transferin	Y	manganese, galactose, uridine	medication	generic	diagnosis	metabolism		https://www.ncbi.nlm.nih.gov/pmc/articles/PMC8720509/	https://www.ncbi.nlm.nih.gov/pmc/articles/PMC8272319/	
TMEM165	Congenital disorder of glycosylation, type IIk	metabolism	AR	N	<1	Neonatal	skeletal dysplasia, neurologic, pulmonary, gastrointestinal, endocrine & hematolgic dysfunction, coagulopathy	Y	carbohydrate deficient transferrin	carbohydrate deficient transferrin	Y	D-galactose	medication	generic	diagnosis	metabolism		https://www.ncbi.nlm.nih.gov/pmc/articles/PMC8720509/	https://www.ncbi.nlm.nih.gov/pmc/articles/PMC6283449/	
PIGA	PIGA-CDG	metabolism	XLR	N	<1	Infancy	cardiac defects, GU anomalies, feeding difficulties, skin abnormalities, scoliosis, severe intellectual disability, epilepsy	Y	GPI anchor flow	GPI anchor flow	Y	pyridoxine (benefit may be limited)	medication	generic	diagnosis	metabolism				

© 2023 Gold NB et al. *JAMA Network Open.*

Gene	Disease name	System	Inheritance	On RUSP? (Y/N)	Prevalence - disease frequency per 100,000 (Rx genes, if listed)	Age of Onset	Disease symptoms	Orthogonal test? (Y/N)	If yes, orthogonal test	If yes, orthogonal test	Is orthogonal test expected to be abnormal in infancy?	Intervention Considered (Free Text)	Category of Intervention	If pharma, what company or companies are making treatment	Age of Intervention Implementation	MD leading intervention	Comments	Link to ref 1	Link to ref 2	Link to ref 3
PIGM	PIGM-CDG	metabolism	AR	N	<1	Early childhood	seizures, thrombosis	Y	GPI anchor flow	GPI anchor flow	Y	pyridoxine (benefit may be limited), anticoagulation	medication	generic	diagnosis	metabolism	very limited papers	https://onlinelibrary.wiley.com/doi/10.1111/epi.16545	https://www.sciencedirect.com/science/article/pii/S0925443909000027?via%3Dihub	
PIGO	PIGO-CDG	metabolism	AR	N	<1	Neonatal	dysmorphic features, hypogonadism, epilepsy, developmental delay, hyperphosphatasia, anorectal anomalies	Y	GPI anchor flow, alkaline phosphotase	GPI anchor flow, alkaline phosphotase	Y	pyridoxine (benefit may be limited)	medication	generic	diagnosis	metabolism		https://onlinelibrary.wiley.com/doi/10.1111/epi.16545	https://pubmed.ncbi.nlm.nih.gov/28545593/	
AMT	Glycine encephalopathy due to aminomethyltransferase (AMT)	metabolism	AR	N	0.36	Neonatal	epileptic encephalopathy	Y	plasma/CSF amino acids	plasma/CSF amino acids	Y	benzoate, NMDA blockade	medication	generic	diagnosis	metabolism, neurology				
OAT	Ornithine aminotransferase deficiency	metabolism	AR	N	0.067	Adolesence-20's	gyrate atrophy	Y	plasma amino acids, OAT enzyme in skin	plasma amino acids, OAT enzyme in skin	Y, but sensitivity unk (AA); Y (enzyme)	pryidoxine, protein restricted diet (arginine-restricted diet)	diet medication	generic	diagnosis	metabolism		https://pubmed.ncbi.nlm.nih.gov/34894815/		
OTC	Ornithine transcarbamylase deficiency	metabolism	XLR	N	1.455	Boys: neonatal-adolesence; girls: neonatal-adulthood	hyperammonemia	Y	plasma amino acids, urine orotic acid	plasma amino acids, urine orotic acid	Y	Protein restriction, citrulline, sodium benzoate, phenylbutyrate, Ravicti, liver transplatation	diet medication OT	Horizon	diagnosis	metabolism				
GLUD1	Hyperinsulinism - hyperammonemia syndrome	metabolism	AD	N	2.4	Neonatal	hyperammonemia and hyperinsulinism	Y	Ammonia, glucose, insulin, free fatty acid levels	Ammonia, glucose, insulin, free fatty acid levels	Y	Diazoxide, somatostatin analogs, nifedipine, glucagon, IGF-1, glucocorticoids, growth hormone, pancreatic resection, mTOR inhibitors, GLP-1 receptor antagonists, sirolimus	medication surgery	many	diagnosis	metabolism or endocrinology		https://pubmed.ncbi.nlm.nih.gov/32229669/		
UMPS	Orotic aciduria	metabolism	AR	N	<1	Childhood	megaloblastic anemia	Y	Urinary orotic acid	Urinary orotic acid	Y	Uridine triacetate	medication	Wellstat	diagnosis	metabolism or hematology				
SLC25A15	Hyperornithinemia-hyperammonemia-homocitrullinuria syndrome	metabolism	AR	N	0.07	Childhood > infantile > neonatal > adolescent-adult	hyperammonemia, liver dysfunction, caogulopathy, hypotonia, motor dysfunction, encephalopathy	Y	Plasma ornithine, urinary homocitrulline	Plasma ornithine, urinary homocitrulline	Y	Protein-restricted diet, citrulline, & nitrogen scavengers	diet medication	Horizon	diagnosis	metabolism		https://www.ncbi.nlm.nih.gov/pmc/articles/PMC6323011/		
SLC25A19	Thiamine metabolism dysfunction syndrome 4	metabolism	AR	N	<0.1	Infancy-childhood	transient encephalopathy, seizures, areflexia, chronic polyneuropathy, skeletal muscle atrophy	N	N/A	N/A		B1 (thiamine)	medication	generic	diagnosis	metabolism		https://pubmed.ncbi.nlm.nih.gov/35102031/		
TPK1	Thiamine metabolism dysfunction syndrome 5	metabolism	AR	N	unk	1.5-4	episodic encephalopathy, developmental delay, hypotonia	Y	Plasma thiamine pyrophosphate level	Plasma thiamine pyrophosphate level		B1 (thiamine)	medication	generic	diagnosis	metabolism		https://www.ncbi.nlm.nih.gov/pmc/articles/PMC5121315/		
SLC2A1	GLUT1 deficiency syndrome 1	metabolism	AD	N	1.7	Infancy (classic) > childhood	epilepsy, movement disorder	Y	Comparison of blood glucose concentration with CSF glucose concentration obtained after 4 hr fast	Comparison of blood glucose concentration with CSF glucose concentration obtained after 4 hr fast	Y	Ketogenic diet and carnitine, avoid barbiturates, methyxanthine (caffeine), valproic acid	diet medication	generic	diagnosis	neurology		https://www.ncbi.nlm.nih.gov/pmc/articles/PMC7469861/		
SLC6A8	Creatine transporter deficiency	metabolism	XLR	N	unk	4-54 mo	developmental delay, speech delay, autism, epilepsy, hypotonia, spasticity	Y	urine creatinine levels, MRS for creatinine	urine creatinine levels, MRS for creatinine	Y	creatine	medication	generic	diagnosis	metabolism	Rx with limited efficacy	https://www.ncbi.nlm.nih.gov/books/NBK3794/		
GAMT	Cerebral creatine deficiency syndrome 2	metabolism	AR	N	0.4	3m-2y	developmental delay, speech delay, autism, seizures, movement disorder	Y	Guanidinoacetate, creatine, & creatinine levels in urine & plasma	Guanidinoacetate, creatine, & creatinine levels in urine & plasma	Y	Creatine monohydrate & ornithine supplementation. Arginine restriction.	diet medication	generic	diagnosis	metabolism		https://www.ncbi.nlm.nih.gov/books/NBK3794/		

© 2023 Gold NB et al. *JAMA Network Open.*

Metabolism (135 genes)

Gene	Disease name	System	Inheritance	On RUSP? (Y/N)	Prevalence - disease frequency per 100,000 (Rx genes, if listed)	Age of Onset	Disease symptoms	Orthogonal test? (Y/N)	If yes, orthogonal test	If yes, orthogonal test	Is orthogonal test expected to be abnormal in infancy?	Intervention Considered (Free Text)	Category of Intervention	If pharma, what company or companies are making treatment	Age of Intervention Implementation	MD leading intervention	Comments	Link to ref 1	Link to ref 2	Link to ref 3
GATM	Cerebral creatine deficiency syndrome 3	metabolism	AR	N	0.01	Childhood	intellectual disability, weakness	Y	Guanidinoacetate, creatine, & creatinine levels in urine & plasma	Guanidinoacetate, creatine, & creatinine levels in urine & plasma		Creatine monohydrate	medication	generic	diagnosis	metabolism		https://www.ncbi.nlm.nih.gov/books/NBK3794/		
ALDH5A1	Succinic semialdehyde dehydrogenase deficiency	metabolism	AR	N	0.1	Infancy	hypotonia, developmental delay, ID, speech delay, ataxia, epilepsy, movement disorder	Y	UOA: 4-hydroxybutyric aciduria	UOA: 4-hydroxybutyric aciduria	Y	vigabatrin	medication	Lundbeck, generic	onset of seizures; some may do at diagnosis; earlist tried is 2.5	neurology	treatment efficacy may be limited	https://www.ncbi.nlm.nih.gov/books/NBK1195/		
SLC30A10	Hypermagnesemia with dystonia 1	metabolism	AR	N	unk	Childhood (2-15 years) > adult	dystonia, movement disorder	Y	Mn level	Mn level	unk	Manganese chelation therapy with EDTA-CaNa2, iron	medication	generic	diagnosis	neurology		https://www.ncbi.nlm.nih.gov/books/NBK100241/		
SLC39A14	Hypermagnesemia with dystonia 2	metabolism	AR	N	unk	6m-3y	motor delay, dystonia, hypotonia, spasticity, parknsonism	Y	Mn level	Mn level	Y	Manganese chelation therapy with EDTA-CaNa2, iron	medication	generic	diagnosis	neurology	evidence presymptomatic or early treamtent is more beneficial	https://www.ncbi.nlm.nih.gov/books/NBK431123/		
ALDOB	Hereditary fructose intolerance	metabolism	AR	N	4.395	6m (or initiation of fructose)	hypoglycemia, lactic acidosis, hypophosphatemia, hyperuricemia, nausea, vomiting, growth restriction, liver failure	Y	carbohydrate deficient transferrin, urine reducing substances, elevated plasma lysosomal enzyme activity, lactic acidemia, hypophosphatemia, hyperuricemia, hypermagnesemia, hyperalaninemia	carbohydrate deficient transferrin, urine reducing substances, elevated plasma lysosomal enzyme activity, lactic acidemia, hypophosphatemia, hyperuricemia, hypermagnesemia, hyperalaninemia	N (unless fructose exposure)	Dietary restriction of fructose, sucrose, and sorbitol	diet		diagnosis	metabolism		https://www.ncbi.nlm.nih.gov/books/NBK333439/		
FBP1	Fructose-1,6-bisphosphatase deficiency	metabolism	AR	N	0.195	50% by day 4; 100% by 1 year	hypoglycemia, lactic acidosis	Y	elevated fasting lactate, alanine, ketosis, pseudohyperglyceridemia, glycerol, g-3p in urine	elevated fasting lactate, alanine, ketosis, pseudohyperglyceridemia, glycerol, g-3p in urine	N	Sucrose & fructose restricted diet especially when ill, uncooked cornstarch to limit overnight fasting	diet medication	generic	diagnosis	metabolism or endocrine				
GALM	Galactose mutarotase deficiency	metabolism	AR	N	0.43	Unk; discovered by newborn screening	cataracts	Y	Plasma galactose level while on a galactose containing diet	Plasma galactose level while on a galactose containing diet	Y	Galactose/lactose-restricted diet	diet		diagnosi	metabolism		https://www.sciencedirect.com/science/article/pii/S1096719218307637?via%3Dihub	https://www.ncbi.nlm.nih.gov/pmc/articles/PMC8628924/	
SLC5A1	Glucose-galactose malabsorption	metabolism	AR	N	unk	2-3 days	watery diarrhea	N	N/A	N/A		Elimination of glucose and galactose from the diet. Use fructose-based formula.	diet							
HIBCH	3-hydroxyisobutryl-CoA hydrolase deficiency	metabolism	AR	N	0.77	0-7 years	Leigh syndrome, hypotonia, developmental delay, epilepsy, dystonia, strabismus	Y	Acylcarnitine profile, urine organic acids	Acylcarnitine profile, urine organic acids	Y	Valine restricted diet, antioxidants, CoQ, thiamine, riboflavin	diet medication	generic	diagnosis	metabolism		https://www.frontiersin.org/articles/10.3389/fphar.2021.605803/full		
HMGCS2	3-hydroxy-3-methylglutaryl-CoA synthase deficiency	metabolism	AR	N	<1	Infancy-early childhood	hypoketotic hypoglycemia	Y	organic acids, acylcarnitine profile	organic acids, acylcarnitine profile	Y	IV glucose during acute episodes, avoid prolonged fasting	diet medication	generic	intercurrent illness	metabolism		https://www.ncbi.nlm.nih.gov/pmc/articles/PMC5979369		
MTHFR	Methylenetetrahydrofolate reductase deficiency	metabolism	AR	N	unk	Infantile-adulthood	neurologic regression, hypotonia, apnea, seizures, microcephaly, psychiatric disturbance, thrombosis	Y	Plasma homocysteine, methionine levels and CSF 5-methyltetrahydrofolate level	Plasma homocysteine, methionine levels and CSF 5-methyltetrahydrofolate level	Y	Betaine, 5-methyltetrahydrofolate	medication	generic	diagnosis	metabolism		https://pubmed.ncbi.nlm.nih.gov/18658082/	https://ojrd.biomedcentral.com/articles/10.1186/s13023-018-0767-9	
MTHFS	5,10-Methenyltetrahydrofolate synthetase deficiency	metabolism	AR	N	unk	Neonatal-infantile	microcephaly, developmental delay, hypertonia, epilepsy	Y	low MTHFS enzyme (fibroblast)	low MTHFS enzyme (fibroblast)	Y	Combination of oral L-5-methyltetrahydrofolate & intramuscular methylcobalamin	medication	generic	diagnosis	metabolism		https://www.ncbi.nlm.nih.gov/pmc/articles/PMC6557438/		

© 2023 Gold NB et al. *JAMA Network Open.*

Metabolism (135 genes)

Gene	Disease name	System	Inheritance	On RUSP? (Y/N)	Prevalence - disease frequency per 100,000 (Rx genes, if listed)	Age of Onset	Disease symptoms	Orthogonal test? (Y/N)	If yes, orthogonal test	If yes, orthogonal test	Is orthogonal test expected to be abnormal in infancy?	Intervention Considered (Free Text)	Category of Intervention	If pharma, what company or companies are making treatment	Age of Intervention Implementation	MD leading intervention	Comments	Link to ref 1	Link to ref 2	Link to ref 3
DDC	Aromatic amino acid decarboxylase deficiency	metabolism	AR	N	0.83	Infantile > neonatal	hypotonia, movement disorder, developmental delay, dysautonomia	Y	CSF neurotransmitters	CSF neurotransmitters	Y	Pyridoxine/pyridoxal phosphate, folinic acid, dopamine agonists, SSRIs, 5-HTP, MAO B inhibitors. Gene therapy (PTC-AADC - clinical trial)	medication gene therapy	generic	diagnosis	metabolism		https://ojrd.biomedcentral.com/articles/10.1186/s13023-016-0522-z		
GLDC	Glycine decarboxylase (GLDC) deficiency	metabolism	AR	N	1.05	Infantile > non-classic late onset	epileptic encephalopathy	Y	plasma amino acids, CSF amino acids	plasma amino acids, CSF amino acids	Y	benzoate, NMDA blockade	medication	generic	diagnosis	metabolism		https://www.ncbi.nlm.nih.gov/books/NBK1357/		
PHGDH	Phosphoglycerate dehydrogenase deficiency	metabolism	AR	N	unknown	Infantile	microcephaly, epilepsy, cataract IUGR	Y	CSF & plasma serine & glycine level	CSF & plasma serine & glycine level	Y	serine, glycine	medication	generic	diagnosis	metabolism		https://pubmed.ncbi.nlm.nih.gov/28440900/		
MLYCD	Malonyl-CoA decarboxylase deficiency	metabolism	AR	N	unknown	Infantile-childhood	cardiomyopathy, developmental delay, seizures, acidosis, hypoglycemia	Y	Plasma acylcarnitine profiles, urine organic acid	Plasma acylcarnitine profiles, urine organic acid	Y	Carnitine, low fat, high MCT, high carbohydrate diet	diet medication	generic	diagnosis	metabolism		https://onlinelibrary.wiley.com/doi/epdf/10.1002/mgg3.1379		
SLC30A2	Transient neonatal zinc deficiency	metabolism	AD	N	unknown	Breastfeeding (presents in offspring)	acrodermatitis in offspring	Y	Zinc levels in breast milk	Zinc levels in breast milk	N	Affected female patients produce breast milk with inadequate zinc. Zn supplementation of their infants until weaning	medication	generic	childbirth	metabolism or GI				
SLC39A4	Acrodermatitis enteropathica	metabolism	AR	N	0.2	Weaning (~6 months)	acrodermatitis, diarrhea	Y	Plasma zinc level	Plasma zinc level		zinc	medication	generic	diagnosis	metabolism				
SLC7A7	Lysinuric protein intolerance	metabolism	AR	N	unknown	Weaning (~6 months)	HLH, hyperammonemia, osteopenia, hepatosplenomegaly	Y	24-hour urinary excretion of cationic amino acids	24-hour urinary excretion of cationic amino acids		Protein restriction, carnitine, citrulline, lysine supplementation, sodium benzoate	diet medication	generic	diagnosis	metabolism				
SORD	Sorbitol dehydrogenase deficiency with peripheral neuropathy	metabolism	AR	N	1	6-17	motor-predominant distal muscle weakness, hyporeflexia, foot deformities	Y	Mass spectrometry analysis of serum sorbitol levels	Mass spectrometry analysis of serum sorbitol levels		Epalrestat (Asia only) and Ranirestat (experimental)	medication	ainippon Sumitomo Pharma and PharmaKyorin	no protocol exists	neurology		https://www.frontiersin.org/articles/10.3389/fneur.2021.733926/full		
TCN2	Transcobalamin II deficiency	metabolism	AR	N	<1	Early infancy	pancytopenia, homocystenemia, methylmalonic acidemia	Y	CBC, Serum amino acids, vitamin B12, & methylmalonic acid levels	CBC, Serum amino acids, vitamin B12, & methylmalonic acid levels	Y	Cobalamin	medication	generic	diagnosis	metabolism		https://pubmed.ncbi.nlm.nih.gov/24305960/		
AGA	Aspartylglucosaminidase deficiency	metabolism	AR	N	unk globally; 1.7 in Finland	Infancy-childhood	hernias, developmental delay, rapid neurologic decline in adulthood	Y	enzyme activity, urine oligosaccharides, vacuolated lymphocytes,	enzyme activity, urine oligosaccharides, vacuolated lymphocytes,	Y	symptomatic; carbamazepine as the primary AED	supportive medication	generic	seizures	neurology	BMT has been shown ineffective	https://ojrd.biomedcentral.com/articles/10.1186/s13023-016-0544-6		
AGXT	Primary hyperoxaluria type I	metabolism	AR	N	1	Infantile-adulthood	kidney stones, renal insufficiency, systemic oxalosis with eye, skin and heart involvement	Y	urinary oxalate, glycolate; AGT enzyme	urinary oxalate, glycolate; AGT enzyme	Y, but requires an age-specific reference range, which is currently not well established	Lumasiran, pyridoxine, drinking large volumes, alkalinzation of urine, pyrophosphate-containing solutions, liver-kidney transplant, dialysis	diet medication procedure OT	Alnylam	hyperhydrosis, pyridoxine and alkalinization at diagnosis, (even if diagnosed in infancy due to an affected sibling)	nephrology		https://www.frontiersin.org/articles/10.3389/fmed.2021.703305/full	https://academic.oup.com/ndt/article/27/5/1729/1844423	
ALDH4A1	Hyperprolinemia, type II	metabolism	AR	N	unk	Childhood	ID, epilepsy	Y	urine P5C, plasma proline	urine P5C, plasma proline	Y	vitamin B6 (pyridoxine)	medication	generic	diagnosis	neurology		https://onlinelibrary.wiley.com/doi/epdf/10.1111/ped.12420	https://adc.bmj.com/content/82/3/236	
APRT	Adenine phosphoribosyltransferase deficiency	metabolism	AR	N	2	Adulthood (50%); any age	crystal nephropathy	Y	Adenine phosphoribosyltransferase enzyme activity	Adenine phosphoribosyltransferase enzyme activity	Y	Allopurinol or Febuxostat, Low purine diet and ample fluid intake	diet medication	generic	diagnosis	nephrology		https://www.ncbi.nlm.nih.gov/books/NBK100238/		

© 2023 Gold NB et al. *JAMA Network Open.*

Metabolism (135 genes)

Gene	Disease name	System	Inheritance	On RUSP? (Y/N)	Prevalence - disease frequency per 100,000 (Rx genes, if listed)	Age of Onset	Disease symptoms	Orthogonal test? (Y/N)	If yes, orthogonal test	If yes, orthogonal test	Is orthogonal test expected to be abnormal in infancy?	Intervention Considered (Free Text)	Category of Intervention	If pharma, what company or companies are making treatment	Age of Intervention Implementation	MD leading intervention	Comments	Link to ref 1	Link to ref 2	Link to ref 3
ATP7A	Menkes disease	metabolism	AR	N	0.28-2.5	2-3 months (classic)	hypotonia, seizures, failure to thrive, vascular tortuosity	Y	Serum ceruloplasmin & copper, plasma catechols	Serum ceruloplasmin & copper, plasma catechols	Y	Subcutaneous injections of copper histidine (Expanded access) or copper chloride, droxidopa (clinical trial)	medication	generic	<4 weeks of age for copper; adulthood for droxidopa	neurology or metabolism		https://www.ncbi.nlm.nih.gov/books/NBK1413/		
CP	Aceruloplasminemia	metabolism	AR	N	0.05	30-70 years	retinal degeneration, diabetes mellitus (DM), and neurologic disease	Y	Serum ceruloplasmin & copper level	Serum ceruloplasmin & copper level	Y	Desferrioxamine, deferaserix, Vitamin E, fresh-frozen plasma	medication transfusion	generic	once Hb> 9	hematology		https://www.ncbi.nlm.nih.gov/books/NBK1493/		
ATP7B	Wilson disease	metabolism	AR	N	3.33	Mean 20-22, but any age possible	hemolytic anemia, liver dysfunction, neuropsychiatric symptoms	Y	Serum ceruloplasmin & copper, urinary copper	Serum ceruloplasmin & copper, urinary copper	Y	Zinc, Trientine, penicillamine, low copper diet	diet medication	Wilson Therapeutics	symptom onset; consider zinc presymptomatically	GI		https://www.ncbi.nlm.nih.gov/books/NBK1512/	https://www.sciencedirect.com/science/article/pii/S0168827811008129	
BCKDK	Branched-chain ketoacid dehydrogenase kinase deficiency	metabolism	AR	N	unk	Neonatal-early childhood	autism, epilepsy, DD, low birth weight	Y	Serum amino acids acids	Serum amino acids acids	Y	High protein diet, BCAA supplement, continuous feeds	diet medication	generic	diagnosis	metabolism		https://pubmed.ncbi.nlm.nih.gov/24449431/		
CA5A	Carbonic anhydrase VA deficiency	metabolism	AR	N	unk	0-20 m	hyperammonemia	Y	elevated glutamine, alanine; low citrulline or gln/cit ratio, organic acids	elevated glutamine, alanine; low citrulline or gln/cit ratio, organic acids	Y	N-carbamylglutamate, IV dextrose when ill, extra calories and limited protein when ill	diet medication	Recordati	sick day precautions when intercurrent illnesses begin; carbaglu if hyperammonemic	metabolism		https://www.ncbi.nlm.nih.gov/books/NBK284774/		
CPS1	Carbamoyl phosphate synthetase I deficiency	metabolism	AR	N	0.08	Neonatal	hyperammonemia	Y	Ammonia, plasma amino acid analysis (glutamine and citrulline), urine orotate	Ammonia, plasma amino acid analysis (glutamine and citrulline), urine orotate	Y	Protein restriction, citrulline, sodium benzoate, phenylbutyrate, liver transplatation, N-carbamylglutamate	diet medication OT	Horizon, Recordati	diagnosis	metabolism		https://www.ncbi.nlm.nih.gov/books/NBK1217/		
CYP27A1	Cerebrotendinous xanthomatosis	metabolism	AR	N	0.48	Neonatal (but may not be recognized until disease progression later)	diarrhea, cataract, xanthoma, cholestasis	Y	Cholestanol level	Cholestanol level	Y	Chenodeoxycholic acid	medication	Travere	diagnosis	metabolism	early treatment is disease-modifying and decreased risk of neurologic disease	https://www.ncbi.nlm.nih.gov/books/NBK1409/		
DHCR7	7-dehydrocholesterol reductase deficiency	metabolism	AR	N	2.5	Congenital	dysmorphic features, undervirilization, developmental delay, microcephaly, adrenal insufficiency, structural brain anomalies	Y	7-dhc level	7-dhc level	Y	cholesterol	medication	generic	diagnosis	metabolism		https://www.ncbi.nlm.nih.gov/books/NBK1143/		
DHFR	Dihydrofolate reductase deficiency	metabolism	AR	N	unk	Childhood	megaloblastic anemia	Y	Complete blood count with MCV & CSF 5-methyltetrahydrofolate level	Complete blood count with MCV & CSF 5-methyltetrahydrofolate level	unk	Folinic acid, 5-F-THF, hydroxycobalamin	medication	generic	development of anemia	hematology		https://www.ncbi.nlm.nih.gov/pmc/articles/PMC3035706/		
DLD	Dihydrolipoamide dehydrogenase deficiency	metabolism	AR	N	unk (2.8 in AJ)	Infancy, childhood	intermittent liver dysfunction, leigh syndrome	Y	plasma amino acids, urine organic acids, DLD enzymology	plasma amino acids, urine organic acids, DLD enzymology	Y (enzyme); severe cases only (metabolites)	low protein diet, riboflavin, N-acetylcysteine, avoidance of alcohol and acetaminophen	diet, medication	generic	diagnosis	metabolism	biochemical abnormalities are intermittent			
GLUL	Glutamine synthetase deficiency	metabolism	AR	N	unk	Prenatal	epileptic encepahalopathy, brain malformations, multiorgan failure, necrolytic skin erythemia, dysmorphic	Y	plasma glutamine	plasma glutamine	Y	supportive care	supportive		diagnosis	neurology		https://www.ncbi.nlm.nih.gov/pmc/articles/PMC5192420/		
GOT2	Glutamic-oxaloacetic transaminase 2 deficiency	metabolism	AR	N	unk	0-1y	microcephaly, failure to thrive, epileptic encephalopathy, developmental delay, intellectual disability	Y	plasma serine	plasma serine	only in severe cases	Vitamin B6 (pyridoxine) and serine	medication	generic	diagnosis	metabolism	biochemical labs have limited sensitivity	https://www.ncbi.nlm.nih.gov/pmc/articles/PMC6732527/		

© 2023 Gold NB et al. *JAMA Network Open.*

Metabolism (135 genes)

Gene	Disease name	System	Inheritance	On RUSP? (Y/N)	Prevalence - disease frequency per 100,000 (Rx genes, if listed)	Age of Onset	Disease symptoms	Orthogonal test? (Y/N)	If yes, orthogonal test	If yes, orthogonal test	Is orthogonal test expected to be abnormal in infancy?	Intervention Considered (Free Text)	Category of Intervention	If pharma, what company or companies are making treatment	Age of Intervention Implementation	MD leading intervention	Comments	Link to ref 1	Link to ref 2	Link to ref 3
IARS1	Isoleucyl-tRNA synthetase deficiency	metabolism	AR	N	unk	Prenatal	steatosis, severe failure to thrive, intellectual disability	Y	zinc levels	zinc levels	unk, but believed Y	Isoleucine supplementation & protein fortification (2.5 mg/kg/day, during illness 3.5 g/kg/day), zinc supplementation	diet medication	generic	diagnosis	metabolism		https://www.ncbi.nlm.nih.gov/pmc/articles/PMC8244667/	https://www.cell.com/ajhg/pdfExtended/S0002-9297(16)30198-7	
LIPA	Lysosomal acid lipase deficiency	metabolism	AR	N	2	Childhood > infancy	hepatomegaly, diarrhea, liver dysfunction, adrenal calcifications, adrenal failure	Y	Leukocytes or whole blood lysosomal acid lipase enzyme activity	Leukocytes or whole blood lysosomal acid lipase enzyme activity	Y	Sebelipase alfa enzyme replacement	ERT	Alexion	symptom onset	metabolism		https://www.ncbi.nlm.nih.gov/books/NBK305870/		
MAN2B1	Alpha-mannosidosis	metabolism	AR	N	0.2	Moderate (early childhood) > mild (adolesence) and severe (congenital)	immune deficiency, dysmorphology, skeletal anomalies, neurodegeneration	Y	Leukocyte acid alpha-mannosidase enzyme activity, urine oligosaccharides	Leukocyte acid alpha-mannosidase enzyme activity, urine oligosaccharides	Y	HSCT, Velmanase enzyme replacement	ERT HSCT	Chiesi	<10y (HSCT)	hematology, metabolism		https://ojrd.biomedcentral.com/articles/10.1186/1750-1172-3-21		
MOCS1	Molybdenum cofactor deficiency A	metabolism	AR	N	0.495	First days of life (classic) > late-onset	epilepsy, lens dislocation, encephalopathy, apnea, feeding difficulties	Y	Urinary xanthine, uric acid, S-sulfocysteine, sulfite, thiosulfate & plasma uric acid	Urinary xanthine, uric acid, S-sulfocysteine, sulfite, thiosulfate & plasma uric acid	Y	Cyclic pyranopterin monophosphate (fosdenopterin), low cysteine diet, thiamine	diet medication	Origin Biosciences	<28 days	metabolism		https://www.ncbi.nlm.nih.gov/books/NBK575630/		
NAGS	N-acetylglutamate synthase deficiency	metabolism	AR	N	0.05	Neonatal	hyperammonemia	Y	Ammonia, plasma amino acid analysis (glutamine and citrulline), urine orotate	Ammonia, plasma amino acid analysis (glutamine and citrulline), urine orotate	Y	N-carbamyl glutamate, low protein diet, nitrogen scavengers, liver transplant	diet medication OT	Horizon, Recordati	diagnosis	metabolism		https://www.ncbi.nlm.nih.gov/books/NBK1217/		
NAXE	NAD(P)HX epimerase deficiency	metabolism	AR	N	unk	8-20 months	ataxia, hypotonia, developmental delay, nystagmus, respiratory failure	Y	fibroblast studies (not CLIA)	fibroblast studies (not CLIA)		nicotinamide (theoretical)	medication	generic	no protocol exists, likely at diagnosis	metabolism	treatment theoretical	https://www.ncbi.nlm.nih.gov/pmc/articles/PMC5065653/pdf/main.pdf		
OXCT1	Succinyl-CoA:3-ketoacid CoA transferase (SCOT) deficiency	metabolism	AR	N	unk	Neonatal-3y	permanent ketosis	Y	urine ketones	urine ketones	Y	IV glucose during acute episodes, avoid prolonged fasting, consider mild fat & protein restriction, bicarbonate	diet medication	generic	symptom onset, bicarbonate just if acidosis	metabolism		https://www.sciencedirect.com/science/article/abs/pii/S0300908421000316	https://www.orpha.net/data/patho/GB/uk-scot.pdf	
PNPO	Pyridoxamine 5-prime-phosphate oxidase deficiency	metabolism	AR	N	unk	Neonatal (most), prenatal, infancy	epilepsy	Y	CSF PLP, enzyme studies	CSF PLP, enzyme studies	Y	pyridoxal-L-phosphate	medication	generic	birth	neurology		https://academic.oup.com/brain/article/137/5/1350/334524		
POR	Cytochrome P450 oxidoreductase deficiency	metabolism	AR	N	unk	Congenital	cortisol deficiency, altered sex steroid synthesis, disorders of sex development (DSD), and skeletal malformations	Y	Serum 17-hydroxyprogesterone, cortisol & adrenocorticotropic hormone (ACTH) levels, pregenelone and progesterone metabolites	Serum 17-hydroxyprogesterone, cortisol & adrenocorticotropic hormone (ACTH) levels, pregenelone and progesterone metabolites	Y	Hydrocortisone, testesterone or estrogen replacement therapy	medication	generic	diagnosis	endocrinology		https://www.ncbi.nlm.nih.gov/books/NBK1419/		
PSAT1	Phosphoserine aminotransferase deficiency	metabolism	AR	N	unk	Neonatal	poor feeding, epilepsy	Y	CSF & plasma serine & glycine level	CSF & plasma serine & glycine level	Y	Serine, glycine	medication	generic	diagnosis	metabolism	disease ultrarare; effects of treatment not know	https://www.ncbi.nlm.nih.gov/pmc/articles/PMC1852735/pdf/AJHGv80p931.pdf		
PSPH	Phosphoserine phosphatase deficiency	metabolism	AR	N	<1	neonatal	dysmorphic features, developmental delay, epilepsy, microcephaly	Y	CSF & plasma serine & glycine level	CSF & plasma serine & glycine level	Y	Serine, glycine	diet, supplement	generic	diagnosis	metabolism		9222972	25080166	
SI	Congenital sucrase-isomaltase deficiency	metabolism	AR	N	110	neonatal-adult onset forms	diarrhea when exposed to glucose	Y	13C-sucrose labeled breath test	13C-sucrose labeled breath test	Y	Avoid sucrose and isomaltose. Oral sacrosidase	diet, medication	QOL Medical	at diagnosis	GI		8576798		

© 2023 Gold NB et al. *JAMA Network Open.*

Metabolism (135 genes)

Gene	Disease name	System	Inheritance	On RUSP? (Y/N)	Prevalence - disease frequency per 100,000 (Rx genes, if listed)	Age of Onset	Disease symptoms	Orthogonal test? (Y/N)	If yes, orthogonal test	If yes, orthogonal test	Is orthogonal test expected to be abnormal in infancy?	Intervention Considered (Free Text)	Category of Intervention	If pharma, what company or companies are making treatment	Age of Intervention Implementation	MD leading intervention	Comments	Link to ref 1	Link to ref 2	Link to ref 3
AP1S1	MEDNIK syndrome	metabolism	AR	N	<1	neonatal-childhood	ID, enteropathy, SNHL neuropathy, ichthyosis, erythroderma, hepatopathy, dysmorphic features, basal ganglia lesions	Y	low cooper, low ceruloplasmin, high free serum copper, elevated VLCFA, elevated bile acids, elevated liver copper	low cooper, low ceruloplasmin, high free serum copper, elevated VLCFA, elevated bile acids, elevated liver copper	Y	zinc acetate	supplement, diet	n/a	at diagnosis	metabolism		30244301	23423674	

© 2023 Gold NB et al. *JAMA Network Open.*

Nephrology (24 genes)

Gene	Disease name	System	Inheritance	On RUSP? (Y/N)	Prevalence - disease frequency per 100,000 (Rx genes, if listed)	Age of Onset	Disease symptoms	Orthogonal test? (Y/N)	If yes, orthogonal test	Is orthogonal test expected to be abnormal in infancy?	Intervention Considered (Free Text)	Category of Intervention	Age of Intervention Implementation	MD leading intervention	Comments	Link to ref 1	Link to ref 2	Link to ref 3
ATP6V0A4	ATP6V0A4 associated distal renal tubular acidosis	nephrology	AR	N	0.046-0.16	infancy to childhood	metabolic acidosis; FTT; emesis, polyuria, polydipsia, fatigue, weakness, rickets, nephrocalcinosis and nephrolithiasis; sensorineural deafness	Y	serum bicarbonate, chloride, potassium, urinary pH and anion gap; hearing screen; renal ultrasound	yes; US may be normal	oral alkali replacement therapy, potassium chloride	medication	infancy	nephrologist				
ATP6V1B1	ATP6V1B1 associated distal renal tubular acidosis	nephrology	AR	N	0.046-0.16	infancy to childhood	metabolic acidosis; FTT; emesis, polyuria, polydipsia, fatigue, weakness, rickets, nephrocalcinosis and nephrolithiasis; sensorineural deafness	Y	serum bicarbonate, chloride, potassium, urinary pH and anion gap; hearing screen; renal ultrasound	yes; US may be normal	oral alkali replacement therapy, potassium chloride	medication	infancy	Nephrologist			https://www.ncbi.nlm.nih.gov/books/NBK547595/	
FOXI1	FOXI1 associated distal renal tubular acidosis	nephrology	AR	N	0.046-0.16	infancy to childhood	metabolic acidosis; FTT; emesis, polyuria, polydipsia, fatigue, weakness, rickets, nephrocalcinosis and nephrolithiasis; sensorineural deafness	Y	serum bicarbonate, chloride, potassium, urinary pH and anion gap; hearing screen; renal ultrasound	yes; US may be normal	oral alkali replacement therapy, potassium chloride	medication	infancy	Nephrologist		https://www.ncbi.nlm.nih.gov/pmc/articles/PMC5827603/	https://www.ncbi.nlm.nih.gov/books/NBK547595/	
SLC4A1	SLC4A1 associated distal renal tubular acidosis	nephrology	AD, AR	N	0.046-0.16	infancy to childhood	similar to other dRTA but can also get hemolytic anemia in certain patients.	Y	serum bicarbonate, chloride, potassium, urinary pH and anion gap; CBC; renal US	yes; US may be normal	oral alkali replacement therapy, potassium chloride	medication		Nephrologist				
WDR72	WDR72 associated distal renal tubular acidosis	nephrology	AR	N	Rare; < 0.1	infancy to childhood	Emesis, polyuria, polydipsia, diarrhea, renal complications like medullary cysts and impaired function	Y	serum bicarbonate, chloride, potassium, urinary pH and anion gap		oral alkali replacement therapy, potassium chloride	medication		Nephrologist		https://www.ncbi.nlm.nih.gov/books/NBK547595/		
SLC4A4	SLC4A4 associated proximal renal tubular acidosis	nephrology	AR	N	Rare; < 0.1	all ages	Severe hypokalemic, hyperchloremic, metabolic acidosis, growth retardation, ocular abnormalities like glaucoma and cataracts	Y	serum bicarbonate, chloride, potassium, urinary pH and anion gap		oral alkali replacement therapy, potassium chloride	medication				https://www.ncbi.nlm.nih.gov/books/NBK519044/	orpha.net/consor/cgi-bin/OC_Exp.php?lng=EN&Expert=47159	
SLC12A1	Bartter syndrome, type 1	nephrology	AR	N	Rare; < 0.1	newborns, later in life	Polyuria, hypoatremia, hypokalemic hypochloremic metabolic alkalosis, hypercalciuria	Y	serum electrolytes, calcium and prostaglandin E2, plasma renin and aldosterone, fractional excretion of potassium, calcium and chloride		sodium chloride, potassium chloride, and indomethacin	medication				https://www.ncbi.nlm.nih.gov/pmc/articles/PMC6755923/	https://omim.org/entry/601678	
KCNJ1	Bartter syndrome, type 2	nephrology	AR	N	Rare; < 0.1	neonatal (typically premature birth)	Deafness, transient hyperkalaemia, severe hypokalaemic hypochloraemic alkalosis	Y	serum electrolytes and prostaglandin E2, plasma renin and aldosterone, fractional excretion of potassium, calcium and chloride		potassium chloride and indomethacin	medication		Nephrologist		https://pubmed.ncbi.nlm.nih.gov/19096086/		
CLCNKB	Bartter syndrome, type 3	nephrology	AR	N	Rare; < 0.1	neonatal, infancy	Growth retartdation, polydipsia, polyuria, constipation, vomiting	Y	serum electrolytes, plasma renin and aldosterone, fractional excretion of potassium and chloride		Indomethacin, spironolactone and potassium chloride	medication		Nephrologist		https://pubmed.ncbi.nlm.nih.gov/31834604/		
BSND	Bartter syndrome, type 4a	nephrology	AR	N	Rare; < 0.1	Antenatal, neonatal, infancy, childhood	sensorineural deafness, increased levels of plasma renin and aldosterone, low to normal blood pressure	Y	serum electrolytes, plasma renin and aldosterone, fractional excretion of sodium, potassium and chloride		potassium and sodium chloride. Indomethacin has limited effects.	medication		Nephrologist		https://www.orpha.net/consor/cgi-bin/OC_Exp.php?lng=EN&Expert=89938#:~:text=A%20form%20of%20Bartter%20syndrome,low%20to%20normal%20blood%20pressure.		
MAGED2	Bartter syndrome, type 5	nephrology	XLR	N	Rare; < 0.1	Antenatal, neonatal, infancy, childhood	Polyuria, hypercalciuria nephrocalcinosis	Y	serum electrolytes and prostaglandin E2, plasma renin and aldosterone, fractional excretion of sodium, potassium, calcium and chloride		sodium chloride, potassium chloride, and indomethacin	medication		Nephrologist		https://omim.org/entry/300971		
COL4A4	Alport syndrome 2	nephrology	AD, AR	N	11	Childhood, kidney disease progressing to failure at about age 40	Proteinuria, microalbuminuria, sensorineural hearing loss in late childhood/adolescence	Y	urinalysis	yes	angiotensin-converting enzyme (ACE) inhibitor	medication		Nephrologist		https://pubmed.ncbi.nlm.nih.gov/33159213/	https://www.ncbi.nlm.nih.gov/books/NBK1207/#alport.Summary	
COL4A3	Alport syndrome 3	nephrology	AD, AR	N	11	Childhood, kidney disease progressing to failure at about age 40	Proteinuria, microalbuminuria, sensorineural hearing loss in late childhood/adolescence	Y	urinalysis	yes	angiotensin-converting enzyme (ACE) inhibitor	medication		Nephrologist		https://pubmed.ncbi.nlm.nih.gov/33159213/	https://www.ncbi.nlm.nih.gov/books/NBK1207/#alport.Summary	
COL4A5	X-linked Alport syndrome 1	nephrology	XLD	N	11	Childhood, kidney disease progressing to failure at about age 40	Proteinuria, microalbuminuria, sensorineural hearing loss in late childhood/adolescence	Y	urinalysis	yes	angiotensin-converting enzyme (ACE) inhibitor	medication		Nephrologist		https://pubmed.ncbi.nlm.nih.gov/33159213/	https://www.ncbi.nlm.nih.gov/books/NBK1207/#alport.Summary	
COQ8B	Nephrotic syndrome, type 9	nephrology	AR	N	1	Neonatal to late-onset (as late as age 70)	Neurodegeneration, steroid-resistant nephrotic syndrome, ESRD, retinopathy or optic atrophy	Y	UA and urine protein/creatinine ratio	yes	CoQ10 supplementation	medication		Nephrologist		https://www.ncbi.nlm.nih.gov/books/NBK410087/		

© 2023 Gold NB et al. *JAMA Network Open.*

Nephrology (24 genes)

Gene	Disease name	System	Inheritance	On RUSP? (Y/N)	Prevalence - disease frequency per 100,000 (Rx genes, if listed)	Age of Onset	Disease symptoms	Orthogonal test? (Y/N)	If yes, orthogonal test	Is orthogonal test expected to be abnormal in infancy?	Intervention Considered (Free Text)	Category of Intervention	Age of Intervention Implementation	MD leading intervention	Comments	Link to ref 1	Link to ref 2	Link to ref 3
SGPL1	Nephrotic syndrome, type 14	nephrology	AR	N	Rare < 0.1	Fetal, adolescent	Adrenal insufficiency, testicular insufficiency, hypothyroidism, ecthyosis, lympohnia/immunodeficiency, neuropathy	Y	urine analysis, serum cortisol and adrenocorticotropic hormone (ACTH) levels	yes	Hydrocortisone, kidney transplant	medication OT		Nephrologist		https://www.ncbi.nlm.nih.gov/books/NBK562988/		
GRHPR	Primary hyperoxaluria type II	nephrology	AR	N	1.7	Infancy/early childhood to sixth decade	Nephrolithiasis, nephrocalcinosis, ESRD	Y	urinary oxalate	yes	pyridoxine, drinking large volumes, alkalinzation of urine, pyrophosphate-containing solutions, liver-kidney transplant	diet medication OT		Nephrologist		https://www.ncbi.nlm.nih.gov/books/NBK1283/		
HOGA1	Primary hyperoxaluria type III	nephrology	AR	N	1.7	Childhood, adolescence	Recurring calcium oxalate stones, nephrocalcinosis, reduced kidney function	Y	urinary oxalate	yes	pyridoxine, drinking large volumes, alkalinzation of urine, pyrophosphate-containing solutions, liver-kidney transplant	diet medication OT		Nephrologist		https://www.ncbi.nlm.nih.gov/books/NBK316514/		
PKD1	Polycystic kidney disease 1	nephrology	AD	N	63.5	Childhood	Hypertension, progressive development and growth of bilateral renal cysts leading to loss of renal function	Y	renal ultrasound	no; cysts may take years to develop	Tolvaptan	medication	variable; medication as an adult	Nephrologist		https://www.ncbi.nlm.nih.gov/pmc/articles/PMC7136168/	https://www.ncbi.nlm.nih.gov/pmc/articles/PMC6716585/	
PKD2	Polycystic kidney disease 2	nephrology	AD	N	63.5	Childhood	Hypertension, progressive development and growth of bilateral renal cysts leading to loss of renal function	Y	renal ultrasound	no; cysts may take years to develop	Tolvaptan	medication	variable; medication as an adult	Nephrologist		https://www.ncbi.nlm.nih.gov/pmc/articles/PMC7136168/	https://www.ncbi.nlm.nih.gov/pmc/articles/PMC6716585/	
PMM2	Polycystic kidney disease with hyperinsulinemic hypoglycemia	nephrology	AR	N	5	Antenatal to adulthood	Developmental delya, severe encephalopathy with axial hypotonia, abnormal eye movements, peripheral neuropathy	Y	phosphomannomutase activity in leukocytes		epalrestat	medication	infancy			https://pubmed.ncbi.nlm.nih.gov/30740725/	https://www.ncbi.nlm.nih.gov/pmc/articles/PMC6899038/	
SLC12A3	Gitelman syndrome	nephrology	AR	N	13.75	Childhood, adolescence, or adulthood	Decreased serum potassium and magnesium levels, muscle weakness, tetany fatigue, palpitations, thyroid dysfunction	Y	serum electrolytes and magnesium, fractional excretion of potassium, sodium, chloride and magnesium	yes	potassium, magnesium and sodium	medication	infancy	Nephrologist		https://pubmed.ncbi.nlm.nih.gov/31578736/	https://www.ncbi.nlm.nih.gov/pmc/articles/PMC6308995/	
CA12	Isolated hyperchlorhidrosis	nephrology	AR	N	Rare < 0.1	Infancy	Visible salt precipitates after sweating, hyponatremic dehydration, poor feeding and slow weight gain at infancy	Y	sweat test	yes	Sodium chloride	medication	infancy	Pulmonologist or Nephrologist		https://www.cell.com/ajhg/fulltext/S0002-9297(10)00524-0	https://pubmed.ncbi.nlm.nih.gov/24714577/	
CTNS	Cystinosis	nephrology	AR	N	0.75	Infancy	Fanconi syndrome, poor growth, hypophosphatemic/calcipenic rickets, imparied glomerular function resulting in complete glomerular failure,	Y	slit-lamp examination of the eye, leukocytes cystine concentration, measurement for renal fanconi syndrome- urine AA, urine phosphorus excretion, glycosuria	yes; slit lamp may not be abnormal until >1 yo but later if treated well from early age	Cysteamine, potassium phosphate, vitamin D, sodium bicarbonate, chlorophyllin to replace copper, carnitine, growth hormone, levothyroxine, insulin, testosterone, renal transplant	medication OT	infancy; birth if known prior	Nephrologist		https://www.ncbi.nlm.nih.gov/books/NBK1400/#ctns.Summary		

© 2023 Gold NB et al. *JAMA Network Open.*

Neurology (83 genes)

Gene	Disease name	System	Inheritance	On RUSP? (Y/N)	Prevalence - disease frequency per 100,000 (Rx genes, if listed)	Age of Onset	Disease symptoms	Orthogonal test? (Y/N)	If yes, orthogonal test	Is orthogonal test expected to be abnormal in infancy?	Intervention Considered (Free Text)	Category of Intervention	Age of Intervention Implementation	MD leading intervention	Comments	Link to ref 1	Link to ref 2	Link to ref 3
TREX1	Aicardi-Goutieres syndrome 1	neurology	AD, AR	N		Infancy, neonatal	Neurologic disability, crying, sleep disturbances, seizures, skin inflammation of the trunk, arms, and legs	Y	Interferon signature	Y*	Baricitinib	medication			*studies identifyi	https://pubmed.ncbi.nlm.nih.gov/32877590/	https://www.orpha.net/consor/cgi-bin/OC_Exp.php?Expert=51&lng=EN	
RNASEH2B	Aicardi-Goutieres syndrome 2	neurology	AR	N		Infancy, neonatal	Neurologic disability, crying, sleep disturbances, seizures, skin inflammation of the trunk, arms, and legs	Y	Interferon signature	Y*	Baricitinib	medication			*studies identifyi	https://pubmed.ncbi.nlm.nih.gov/32877590/	https://www.orpha.net/consor/cgi-bin/OC_Exp.php?Expert=51&lng=EN	
RNASEH2C	Aicardi-Goutieres syndrome 3	neurology	AR	N		Infancy, neonatal	Neurologic disability, crying, sleep disturbances, seizures, skin inflammation of the trunk, arms, and legs	Y	Interferon signature	Y*	Baricitinib	medication			*studies identifyi	https://pubmed.ncbi.nlm.nih.gov/32877590/	https://www.orpha.net/consor/cgi-bin/OC_Exp.php?Expert=51&lng=EN	
RNASEH2A	Aicardi-Goutieres syndrome 4	neurology	AR	N		Infancy, neonatal	Neurologic disability, crying, sleep disturbances, seizures, skin inflammation of the trunk, arms, and legs	Y	Interferon signature	Y*	Baricitinib	medication			*studies identifyi	https://pubmed.ncbi.nlm.nih.gov/32877590/	https://www.orpha.net/consor/cgi-bin/OC_Exp.php?Expert=51&lng=EN	
SAMHD1	Aicardi-Goutieres syndrome 5	neurology	AR	N		Infancy, neonatal	Neurologic disability, crying, sleep disturbances, seizures, skin inflammation of the trunk, arms, and legs	Y	Interferon signature	Y*	Baricitinib	medication			*studies identifyi	https://pubmed.ncbi.nlm.nih.gov/32877590/	https://www.orpha.net/consor/cgi-bin/OC_Exp.php?Expert=51&lng=EN	
ADAR	Aicardi-Goutieres syndrome 6	neurology	AD, AR	N		Infancy, neonatal	Neurologic disability, crying, sleep disturbances, seizures, skin inflammation of the trunk, arms, and legs	Y	Interferon signature	Y*	Baricitinib	medication			*studies identifyi	https://pubmed.ncbi.nlm.nih.gov/32877590/	https://www.orpha.net/consor/cgi-bin/OC_Exp.php?Expert=51&lng=EN	
IFIH1	Aicardi-Goutieres syndrome 7	neurology	AD	N		Infancy, neonatal	Neurologic disability, crying, sleep disturbances, seizures, skin inflammation of the trunk, arms, and legs	Y	Interferon signature	Y*	Baricitinib	medication			*has been report	https://pubmed.ncbi.nlm.nih.gov/32877590/	https://www.orpha.net/consor/cgi-bin/OC_Exp.php?Expert=51&lng=EN	
LSM11	Aicardi-Goutieres syndrome 8	neurology	AR	N		Infancy, neonatal	Neurologic disability, crying, sleep disturbances, seizures, skin inflammation of the trunk, arms, and legs	Y	Interferon signature	U	Baricitinib	medication				https://pubmed.ncbi.nlm.nih.gov/32877590/	https://www.orpha.net/consor/cgi-bin/OC_Exp.php?Expert=51&lng=EN	
RNU7-1	Aicardi-Goutieres syndrome 9	neurology	AR	N		Infancy, neonatal	Neurologic disability, crying, sleep disturbances, seizures, skin inflammation of the trunk, arms, and legs	Y	Interferon signature	Y*	Baricitinib	medication			*has been report	https://pubmed.ncbi.nlm.nih.gov/32877590/	https://www.orpha.net/consor/cgi-bin/OC_Exp.php?Expert=51&lng=EN	
CHRNA1	Congenital myasthenic syndrome 1	neurology	AD, AR	N	0.92	Infancy, neonatal	Fatigable weakness involving ocular, bulbar, and limb muscles	Y	Repetitive nerve stimulation test	Y	Acetylcholine-esterase inhibitors, quinidine, fluoxetine, 3,4-diaminopyridine	medication				https://www.ncbi.nlm.nih.gov/books/NBK1168/#	https://pubmed.ncbi.nlm.nih.gov/30808424/	
CHRNB1	Congenital myasthenic syndrome 2	neurology	AD, AR	N	0.92	Infancy, neonatal	Fatigable weakness involving ocular, bulbar, and limb muscles	Y	Repetitive nerve stimulation test	Y	Fluoxetine. Acetylcholine-esterase inhibitors ineffective	medication				https://www.ncbi.nlm.nih.gov/books/NBK1168/#	https://pubmed.ncbi.nlm.nih.gov/30808424/	
CHRND	Congenital myasthenic syndrome 3	neurology	AD, AR	N	0.92	Infancy, neonatal	Fatigable weakness involving ocular, bulbar, and limb muscles	Y	Repetitive nerve stimulation test	Y	3,4-diaminopyridine, Acetylcholine-esterase inhibitors	medication				https://www.ncbi.nlm.nih.gov/books/NBK1168/#	https://pubmed.ncbi.nlm.nih.gov/30808424/	
CHRNE	Congenital myasthenic syndrome 4	neurology	AD, AR	N	0.92	Infancy, neonatal	Fatigable weakness involving ocular, bulbar, and limb muscles	Y	Repetitive nerve stimulation	Y	Acetylcholine-esterase inhibitors (note: for some patients pyridostigmine and 3,4-diaminopyridine may be ineffective or may worsen the phenotype), albuterol, salbutamol, fluoxetine and drug combinations	medication				https://www.ncbi.nlm.nih.gov/books/NBK1168/#	https://pubmed.ncbi.nlm.nih.gov/30808424/	
COLQ	Congenital myasthenic syndrome 5	neurology	AR	N	0.92	Infancy, neonatal	Fatigable weakness involving ocular, bulbar, and limb muscles	Y	Repetitive nerve stimulation	Y	Ephedrine, 3,4-diaminopyridine, salbutamol	medication				https://www.ncbi.nlm.nih.gov/books/NBK1168/#	https://pubmed.ncbi.nlm.nih.gov/30808424/	
CHAT	Congenital myasthenic syndrome 6	neurology	AR	N	0.92	Infancy, neonatal	Fatigable weakness involving ocular, bulbar, and limb muscles	Y	Repetitive nerve stimulation	Y	Acetylcholine-esterase inhibitors	medication				https://www.ncbi.nlm.nih.gov/books/NBK1168/#	https://pubmed.ncbi.nlm.nih.gov/30808424/	
SYT2	Congenital myasthenic syndrome 7	neurology	AD	N	0.92	Infancy, neonatal	Fatigable weakness involving ocular, bulbar, and limb muscles	Y	Repetitive nerve stimulation	Y	Acetylcholine-esterase inhibitors, 3,4-diaminopyridine	medication				https://www.ncbi.nlm.nih.gov/books/NBK1168/#	https://pubmed.ncbi.nlm.nih.gov/30808424/	
AGRN	Congenital myasthenic syndrome 8	neurology	AR	N	0.92	Infancy, neonatal	Fatigable weakness involving ocular, bulbar, and limb muscles	Y	Repetitive nerve stimulation	Y	Ephedrine, Acetylcholine-esterase inhibitors	medication				https://www.ncbi.nlm.nih.gov/books/NBK1168/#	https://pubmed.ncbi.nlm.nih.gov/30808424/	
MUSK	Congenital myasthenic syndrome 9	neurology	AR	N	0.92	Infancy, neonatal	Fatigable weakness involving ocular, bulbar, and limb muscles	Y	Repetitive nerve stimulation	Y	Salbutamol, 3,4-diaminopyridine, albuterol, Acetylcholine-esterase inhibitors worsen the phenotype	medication				https://www.ncbi.nlm.nih.gov/books/NBK1168/#	https://pubmed.ncbi.nlm.nih.gov/30808424/	
DOK7	Congenital myasthenic syndrome 10	neurology	AR	N	0.92	Infancy, neonatal	Fatigable weakness involving ocular, bulbar, and limb muscles	Y	Repetitive nerve stimulation	Y	Ephedrine, Salbutamol, albuterol, Acetylcholine-esterase inhibitors are usually ineffective and may even worsen clinical manifestations	medication				https://www.ncbi.nlm.nih.gov/books/NBK1168/#	https://pubmed.ncbi.nlm.nih.gov/30808424/	
RAPSN	Congenital myasthenic syndrome 11	neurology	AR	N	0.92	Infancy, neonatal	Fatigable weakness involving ocular, bulbar, and limb muscles	Y	Repetitive nerve stimulation	Y	Acetylcholine-esterase inhibitors, 3,4-diaminopyridine. Fluoxetine may worsen the condition.	medication				https://www.ncbi.nlm.nih.gov/books/NBK1168/#	https://pubmed.ncbi.nlm.nih.gov/30808424/	
GFPT1	Congenital myasthenic syndrome 12	neurology	AR	N	0.92	Infancy, neonatal	Fatigable weakness involving ocular, bulbar, and limb muscles	Y	Repetitive nerve stimulation	Y	Acetylcholine-esterase inhibitors	medication				https://www.ncbi.nlm.nih.gov/books/NBK1168/#	https://pubmed.ncbi.nlm.nih.gov/30808424/	
DPAGT1	Congenital myasthenic syndrome 13	neurology	AR	N	0.92	Infancy, neonatal	Fatigable weakness involving ocular, bulbar, and limb muscles	Y	Repetitive nerve stimulation	Y	Acetylcholine-esterase inhibitors, 3,4-diaminopyridine	medication				https://www.ncbi.nlm.nih.gov/books/NBK1168/#	https://pubmed.ncbi.nlm.nih.gov/30808424/	
ALG2	Congenital myasthenic syndrome 14	neurology	AR	N	0.92	Infancy, neonatal	Fatigable weakness involving ocular, bulbar, and limb muscles	Y	Repetitive nerve stimulation	Y	Acetylcholine-esterase inhibitors	medication				https://www.ncbi.nlm.nih.gov/books/NBK1168/#	https://pubmed.ncbi.nlm.nih.gov/30808424/	
ALG14	Congenital myasthenic syndrome 15	neurology	AR	N	0.92	Infancy, neonatal	Fatigable weakness involving ocular, bulbar, and limb muscles	Y	Repetitive nerve stimulation	Y	Acetylcholine-esterase inhibitors	medication				https://www.ncbi.nlm.nih.gov/books/NBK1168/#	https://pubmed.ncbi.nlm.nih.gov/30808424/	
SCN4A	Congenital myasthenic syndrome 16	neurology	AR	N	0.92	Infancy, neonatal	Fatigable weakness involving ocular, bulbar, and limb muscles	Y	Repetitive nerve stimulation	Y	acetylcholineesterase (AChE) inhibitors	medication				https://www.ncbi.nlm.nih.gov/books/NBK1168/#	https://pubmed.ncbi.nlm.nih.gov/30808424/	
LRP4	Congenital myasthenic syndrome 17	neurology	AR	N	0.92	Infancy, neonatal	Fatigable weakness involving ocular, bulbar, and limb muscles	Y	Repetitive nerve stimulation	Y	Albuterol. Acetylcholine-esterase inhibitors ineffecive.	medication				https://www.ncbi.nlm.nih.gov/books/NBK1168/#	https://pubmed.ncbi.nlm.nih.gov/30808424/	
SNAP25	Congenital myasthenic syndrome 18	neurology	AD	N	0.92	Infancy, neonatal	Fatigable weakness involving ocular, bulbar, and limb muscles	Y	Repetitive nerve stimulation	Y	3,4-diaminopyridine. Acetylcholineesterase (AChE) inhibitors ineffective.	medication				https://www.ncbi.nlm.nih.gov/books/NBK1168/#	https://pubmed.ncbi.nlm.nih.gov/30808424/	
COL13A1	Congenital myasthenic syndrome 19	neurology	AR	N	0.92	Infancy, neonatal	Fatigable weakness involving ocular, bulbar, and limb muscles	Y	Repetitive nerve stimulation	Y	Salbutamol, 3,4-diaminopyridine. Acetylcholine-esterase inhibitors ineffecive.	medication				https://www.ncbi.nlm.nih.gov/books/NBK1168/#	https://pubmed.ncbi.nlm.nih.gov/30808424/	
SLC5A7	Congenital myasthenic syndrome 20	neurology	AR	N	0.92	Infancy, neonatal	Fatigable weakness involving ocular, bulbar, and limb muscles	Y	Repetitive nerve stimulation	Y	Salbutamol, Acetylcholine-esterase inhibitors	medication				https://www.ncbi.nlm.nih.gov/books/NBK1168/#	https://pubmed.ncbi.nlm.nih.gov/30808424/	
SLC18A3	Congenital myasthenic syndrome 21	neurology	AR	N	0.92	Infancy, neonatal	Fatigable weakness involving ocular, bulbar, and limb muscles	Y	Repetitive nerve stimulation	Y	Acetylcholine-esterase inhibitors	medication				https://www.ncbi.nlm.nih.gov/books/NBK1168/#	https://pubmed.ncbi.nlm.nih.gov/30808424/	
PREPL	Congenital myasthenic syndrome 22	neurology	AR	N	0.92	Infancy, neonatal	Fatigable weakness involving ocular, bulbar, and limb muscles	Y	Repetitive nerve stimulation	Y	Acetylcholine-esterase inhibitors, growth hormone	medication				https://www.ncbi.nlm.nih.gov/books/NBK1168/#	https://pubmed.ncbi.nlm.nih.gov/30808424/	

© 2023 Gold NB et al. *JAMA Network Open.*

Neurology (83 genes)

Gene	Disease name	System	Inheritance	On RUSP? (Y/N)	Prevalence - disease frequency per 100,000 (Rx genes, if listed)	Age of Onset	Disease symptoms	Orthogonal test? (Y/N)	If yes, orthogonal test	Is orthogonal test expected to be abnormal in infancy?	Intervention Considered (Free Text)	Category of Intervention	Age of Intervention Implementation	MD leading intervention	Comments	Link to ref 1	Link to ref 2	Link to ref 3
SLC25A1	Congenital myasthenic syndrome 23	neurology	AR	N	0.92	Infancy	Fatigable weakness involving ocular, bulbar, and limb muscles	Y	Repetitive nerve stimulation	Y	3,4-diaminopyridine	medication				https://pubmed.ncbi.nlm.nih.gov/33397003/		
MYO9A	Congenital myasthenic syndrome 24	neurology	AR	N	0.92	Infancy, neonatal	Fatigable weakness involving ocular, bulbar, and limb muscles	Y	Repetitive nerve stimulation	Y	Acetylcholineesterase (AChE) inhibitors, 3,4-diaminopyridine	medication				https://www.ncbi.nlm.nih.gov/books/NBK1168/#	https://pubmed.ncbi.nlm.nih.gov/30808424/	
ALDH7A1	Pyridoxine-dependent epilepsy	neurology	AR	N	2.565	Infancy, childhood	Seizures, intellectual disability (classic presentation)	Y	Urine organic acids	Y	vitamin B6 (pyridoxine), lysine restrict and suppl arginine	diet medication				https://www.ncbi.nlm.nih.gov/books/NBK1486/		
PLPBP	Vitamin B6-dependent epilepsy	neurology	AR	N	2.565	Prenatal, neonatal, postnatal	Recurrent seizures, intellectual disability, behavioural abnormalities, abnormalities in brain structure and myelination	Y	Intravenous pyridoxine trial on EEG	Y	vitamin B6 (pyridoxine), lysine restrict and suppl arginine	diet medication				https://www.ncbi.nlm.nih.gov/pmc/articles/PMC6391652/		
SCARB2	Progressive myoclonic epilepsy 4	neurology	AR	N		Teens or twenties	Tremor, myoclonus, ataxia, epilepsy	Y	Plasma glucosylsphingosine		Miglustat	medication				https://www.ncbi.nlm.nih.gov/pmc/articles/PMC8320328/		
SCN3A	Familial focal epilepsy with variable foci 4	neurology	AD	N		Childhood	Epilepsy, malformation of cortical development	N			antiepileptic medications phenytoin and lacosamide	medication				https://www.ncbi.nlm.nih.gov/pmc/articles/PMC8552104/		
KCNA1	Episodic ataxia/myokymia syndrome	neurology	AD	N		Early childhood	Incoordination, imbalance, myokymia, vertigo	N			oxcarbazepine, carbamazepine, acetazolamide, magnesium	medication				https://omim.org/entry/160120	https://www.ncbi.nlm.nih.gov/books/NBK25442/	
CACNA1A	Episodic ataxia, type 2	neurology	AD	N		Childhood, early adolescence	Ataxia, vertigo, nausea, migraine	N			acetazolamide	medication				https://www.ncbi.nlm.nih.gov/books/NBK1501/		
SLC1A3	Episodic ataxia, type 6	neurology	AD	N		Childhood	Ataxia, slurred speech, headache, hemiplegia	N			acetazolamide	medication				https://www.orpha.net/consor/cgi-bin/OC_Exp.php?Expert=209967&lng=EN#:~:text=Episodic%20ataxia%20type%206%20(EA6,%2C%20headache%2C%20confusion%20and%20hemiplegia.		
ATM	Ataxia-telangiectasia	neurology	AR	N		Childhood (age 1-4)	Progressive ataxia, oculomotor apraxia, choreoathetosis, telangiectasias	Y	Serum alpha fetoprotein	Y	Immunoglobulin, transplantation of embryonic stem cells antioxidants	medication transfusion HSCT				https://www.chop.edu/conditions-diseases/ataxia-telangiectasia		https://pubmed.ncbi.nlm.nih.gov/17586848/
TTPA	Ataxia with vitamin E deficiency	neurology	AR	N	0.205	Late childhood, early teens	Progressive ataxia, clumsiness of the hands, loss of proprioception, areflexi, dysdiadochokinesia, dysarthria, positive Romberg sign, head titubation, decreased visual acuity, & positive Babinski sign	Y	Plasma vitamin E (tocopherol) level	Y	Alpha-tocopherol	medication				https://pubmed.ncbi.nlm.nih.gov/20301419/		
SCN1A	Early infantile epileptic encephalopathy 6	neurology	AD	N	3.595	Infancy	Spectrum of epilepsies ranging from genetic epilepsy with febrile seizures plus (GEFS+), to developmental and epileptic encephalopathies (DEEs), Dravet Syndrome, hemiplegic migrane	N			Avoid sodium channel blockers, stiripentol, perampanel has been effective	medication				https://pubmed.ncbi.nlm.nih.gov/31904117/		
KCNQ2	Early infantile epileptic encephalopathy 7	neurology	AD	N	2.8	Neonatal, infancy, childhood	Chronic early seizures with associated focal motor & autonomic features, apnea, cyanosis	N			Arbamazepine, ezogabine for loss-of function variants, sodium channel blockers	medication				https://pubmed.ncbi.nlm.nih.gov/20437616/		
SCN2A	Early infantile epileptic encephalopathy 11	neurology	AD	N		Neonatal	Developmental and epileptic encephalopathies, benign familial neonatal-infantile seizures, episodic ataxia, and autism spectrum disorder and intellectual disability with & without seizures	N			Phenytoin; high dose carbamazepine	medication				https://pubmed.ncbi.nlm.nih.gov/31924505/		
SCN8A	Early infantile epileptic encephalopathy 13	neurology	AD	N		Infancy	Epilepsy,neurodevelopmental disorders, seizures	N			Phenytoin; high dose carbamazepine\	medication				https://pubmed.ncbi.nlm.nih.gov/25568300/		
KCNT1	Early infantile epileptic encephalopathy 14	neurology	AD	N		Infancy	Epilepsy of infancy with migrating focal seizures(EIMFS), autosomal dominant nocturnal frontal lobe epilepsy (ADNFLE)	N			Quinidine for gain of function variants	medication				https://pubmed.ncbi.nlm.nih.gov/30234941/		
SLC13A5	Early infantile epileptic encephalopathy 25	neurology	AR	N		Infancy	Non-alcoholic fatty liver disease, obesity, insulin resistance, cell proliferation, and early infantile epileptic encephalopathy	Y	Plasma and CSF citrate levels	Y	Ketogenic diet, stiripentol	diet medication				https://pubmed.ncbi.nlm.nih.gov/34677420/		
CAD	Early infantile epileptic encephalopathy 50	neurology	AR	N		Infancy	Seizure, muscular hypotonia, and developmental delay	Y	Complete blood count and peripheral blood smear for anisopoikilocytosis	Y	Uridine	medication				https://pubmed.ncbi.nlm.nih.gov/35277149/		
GLRA1	Hyperekplexia 1	neurology	AD, AR	N		Neonatal	Stiffness, startles, apnoea attacks, delayed development, mild-to severe delay in speech acquistion	N			Clonazepam	medication				https://pubmed.ncbi.nlm.nih.gov/24030948/	https://www.ncbi.nlm.nih.gov/books/NBK1260/	
GLRB	Hyperekplexia 2	neurology	AD, AR	N		Neonatal	Stiffness, startles, apnoea attacks, delayed development, mild-to severe delay in speech acquistion	N			Clonazepam	medication				https://pubmed.ncbi.nlm.nih.gov/24030948/	https://www.ncbi.nlm.nih.gov/books/NBK1260/	
SLC6A5	Hyperekplexia 3	neurology	AD, AR	N		Neonatal	Stiffness, startles, apnoea attacks, delayed development, mild-to severe delay in speech acquistion	N			Clonazepam	medication				https://pubmed.ncbi.nlm.nih.gov/24030948/	https://www.ncbi.nlm.nih.gov/books/NBK1260/	
GRIN1	Ionotropic glutamate receptor NMDA type subunit 1 dysregulation	neurology	AD	N		Infancy	Mild-to-severe developmental delay, intellectual disability, epilepsy, muscular hypotonia, movement disorders, spasticity, feeding difficulties, & behavior issues	N			Seizure medications, vagus nerve stimulation (VNS), ketogenic diet	diet medication surgery				https://pubmed.ncbi.nlm.nih.gov/31219694/	https://www.chop.edu/conditions-diseases/grin1-related-disorders	https://www.chop.edu/conditions-diseases/grin1-related-disorders
GRIN2A	Ionotropic glutamate receptor NMDA type subunit 2A dysregulation	neurology	AD	N		Childhood	Epileptic spectrum, speech or language impairment, intellectual disability/ development delay	N			Memantine, dextromethorphan for gain of function variants; avoid phenytoin, barbiturates and carbamazepine	medication				https://pubmed.ncbi.nlm.nih.gov/35217385/	https://www.ncbi.nlm.nih.gov/books/NBK385627/	
GRIN2B	Ionotropic glutamate receptor NMDA type subunit 2B dysregulation	neurology	AD	N		Infancy, childhood	Neurodevelopmental disorders, developmental delay,autism, attention-deficit/ hyperactivity disorder (ADHD), schizophrenia, Alzheimer's disease associated	N			Seizure medications, vagus nerve stimulation (VNS), ketogenic diet	diet medication surgery				**https://pubmed.ncbi.nlm.nih.gov/35217385/**	https://www.chop.edu/conditions-diseases/grin2b-related-disorders	https://www.chop.edu/conditions-diseases/grin2b-related-disorders
GRIN2D	Ionotropic glutamate receptor NMDA type subunit 2D superactivity	neurology	AD	N		Infancy, childhood	Neurodevelopmental disorders, developmental delay,autism, attention-deficit/ hyperactivity disorder (ADHD), schizophrenia, Alzheimer's disease associated	N			Memantine, dextromethorphan for gain of function variants	medication				https://www.ncbi.nlm.nih.gov/pmc/articles/PMC7365295/		
SLC25A12	Mitochondrial aspartate-glutamate carrier isoform 1 deficiency (aralar deficiency)	neurology	AR	N		Childhood	Severe hypotonia, arrested psychomotor development, seizures & global hypomyelination, epilepsy, cerebral atrophy,	N			Levetiracetam, oxcarbazepine, antiepileptic, topiramate, phenobarbital ketogenic diet	diet medication				https://pubmed.ncbi.nlm.nih.gov/31514314/ https://www.ncbi.nlm.nih.gov/pmc/articles/PMC6788951/	https://www.ncbi.nlm.nih.gov/pmc/articles/PMC6788951/	https://www.ncbi.nlm.nih.gov/pmc/articles/PMC6788951/
SLC18A2	Infantile parkinsonism-dystonia 2	neurology	AR	N		Early infancy	Neuropsychiatric disorders, particularly attention deficit hyperactivity disorder	Y	Whole blood serotonin level		Dopamine receptor agonist (pramipexole)	medication				https://pubmed.ncbi.nlm.nih.gov/19478460/		
SLC52A3	Brown-Vialetto-Van Laere syndrome 1	neurology	AR	N		Infancy to third decade	Development of esophageal squamous cell carcinoma (ESCC)	Y	Plasma acylcarnitine profile, urine organic acids	N* (PMID: 21110	Riboflavin	medication			*Important to not	https://pubmed.ncbi.nlm.nih.gov/29428966/	https://pubmed.ncbi.nlm.nih.gov/18416855/	
SLC52A2	Brown-Vialetto-Van Laere syndrome 2	neurology	AR	N		Infancy to third decade	Early onset of sensorineural hearing loss, bulbar palsy, peripheral neuropathy, & respiratory insufficiency	Y	Plasma acylcarnitine profile, urine organic acids		Riboflavin	medication				https://pubmed.ncbi.nlm.nih.gov/34737166/	https://pubmed.ncbi.nlm.nih.gov/18416855/	

© 2023 Gold NB et al. *JAMA Network Open.*

Neurology (83 genes)

Gene	Disease name	System	Inheritance	On RUSP? (Y/N)	Prevalence - disease frequency per 100,000 (Rx genes, if listed)	Age of Onset	Disease symptoms	Orthogonal test? (Y/N)	If yes, orthogonal test	Is orthogonal test expected to be abnormal in infancy?	Intervention Considered (Free Text)	Category of Intervention	Age of Intervention Implementation	MD leading intervention	Comments	Link to ref 1	Link to ref 2	Link to ref 3
SPR	Dopa-responsive dystonia due to sepiapterin reductase deficiency	neurology	AR	N	0.725	Nonspecific features in infancy, other features develop over time	Motor & language delays, axial hypotonia, dystonia, weakness, oculogyric crises, diurnal fluctuation of symptoms with sleep	Y	CSF neurotransmitter metabolites and pterins		Levodopa combined with a decarboxylase inhibitor, 5-hydroxytryptophan	medication				https://pubmed.ncbi.nlm.nih.gov/22522443/	https://www.ncbi.nlm.nih.gov/books/NBK304122/	
TH	Dopa-responsive dystonia due to tyrosine hydroxylase deficiency	neurology	AR	N	0.725	Infancy, childhood	Progressive infantile encephalopathy dominated by decrease motor use, fluctuating extrapyramidal, ocular & vegetative symptoms, prenatally disturbed brain development, impaired catecholaminergic neurotransmission	Y	CSF neurotransmitter metabolites and pterins		Levodopa combined with a decarboxylase inhibitor	medication				https://pubmed.ncbi.nlm.nih.gov/12891655/	https://www.ncbi.nlm.nih.gov/books/NBK1437/	
TMLHE	Epsilon-N-trimethyllysine hydroxylase deficiency	neurology	XLR	N		Childhood	Cardiomyopathy (with or without generalized skeletal muscle weakness),Reye-like metabolic decompensation, isolated gastrointestinal symptoms, mild developmental delay	N			Carnitine supplementation	medication				https://pubmed.ncbi.nlm.nih.gov/25943046//		
SPTLC1	Hereditary sensory neuropathy type IA	neurology	AD	N		Teens to sixth decade	Early sensoy loss, dysesthesia, shooting pains,distal weakness in muscles	Y	Sphingolipid levels		Serine	medication				https://pubmed.ncbi.nlm.nih.gov/20301564/	https://www.ncbi.nlm.nih.gov/books/NBK1390/	
SPTLC2	Hereditary sensory neuropathy type IC	neurology	AD	N		Teens to sixth decade	Sensory deficits, neuropathic pain, & recurrent ulcerations	Y	Sphingolipid levels		Serine	medication				https://pubmed.ncbi.nlm.nih.gov/34090020/	https://omim.org/entry/613640	
FLAD1	Lipid storage myopathy due to flavin adenine dinucleotide synthetas	neurology	AR	N	0.4	Neonatal, infancy, childhood	Metabolic disorders, multiple acyl-CoA dehydrogenase deficiency	Y	Plasma acylcarnitine profile, Urine organic acid analysis,		Riboflavin, carnitine, glycine, Coenzyme Q10 supplementation, fat restriction, avoidance of fasting, and a diet rich in carbohydrates	diet<br>medication				https://pubmed.ncbi.nlm.nih.gov/31392824/		
GNE	GNE myopathy	neurology	AR	N	0.1	Early adulthood	Bilateral foot drop, skeletal muscle detoriation	Y	Plasma n-acetylneuraminic acid (Neu5Ac) concentration by tandem mass spectrometry		N-acetylmannosamine	medication				https://pubmed.ncbi.nlm.nih.gov/30338442/		
TSC1	Tuberous sclerosis 1	neurology	AD	N	10.205	Various ages (childhood to adulthood)	Somatic loss, associated hamartom seizures, moderate-to-severe mental retardation, facial angiofibroma	N			Vigabatrin for seizures, mTOR inhibitor for rhabdomyoma, Epidiolex (cannabidiol) for seizures	medication				https://pubmed.ncbi.nlm.nih.gov/11112665/	https://www.ncbi.nlm.nih.gov/books/NBK1220/	
TSC2	Tuberous sclerosis 2	neurology	AD	N	10.205	Various ages (childhood to adulthood)	Somatic loss, associated hamartom seizures, moderate-to-severe mental retardation, facial angiofibroma	N			Vigabatrin for seizures, mTOR inhibitor for rhabdomyoma, Epidiolex (cannabidiol) for seizures	medication				https://pubmed.ncbi.nlm.nih.gov/11112665/	https://www.ncbi.nlm.nih.gov/books/NBK1220/	
ARSA	Metachromatic leukodystrophy	neurology	AR	N	1.565	Before age 30 months	Progressive motor and cognitive deficiency	Y	arylsulfatase A enzyme activity in leukocytes, urinary sulfatides		Hematopoietic stem cell transplantation (HSCT) - bone marrow transplant, Skysona (elivaldogene autotemcel, Lenti-D), atidarsagene autotemcel (Libmeldy)	HSCT<br>gene therapy				https://pubmed.ncbi.nlm.nih.gov/33195324/	https://www.ncbi.nlm.nih.gov/books/NBK1130/	
CACNA1S	Hypokalemic periodic paralysis type 1	neurology	AD	N	1	Two to thirty years old	Episodic paralysis - coexisting with permanent weakness, vascular myopathy	N			Potassium supplementation, acetazolamide, dichlorphenamide	medication				https://pubmed.ncbi.nlm.nih.gov/34120654/	https://www.ncbi.nlm.nih.gov/books/NBK1338/	
CHD7	CHARGE syndrome	neurology	AD	N	1.72	Childhood, adolescence, adulthood	Ocular coloboma, cogenital heart defects, choanal atresia, slow growth & development, genital hypoplasia, & ear anomlies associated with deafness	Y	T and B Lymphocyte and Natural Killer Cell Profile		Hematopoietic stem cell transplantation (HSCT) - bone marrow transplant	HSCT				https://pubmed.ncbi.nlm.nih.gov/21378379/	https://www.ncbi.nlm.nih.gov/books/NBK1117/	
CLCN1	Myotonia congenita	neurology	AD, AR	N	1.225	Childhood	Muscle stiffness and/or weakness	Y	Electromyography test		Mexiletine. Lamotrigine. Phenytoin and carbamazepine have been reported to have beneficial effects. Quinine, dantrolene, or acetazolamide may be beneficial in some cases. Avoid certain pharmacologic agents.	medication				https://pubmed.ncbi.nlm.nih.gov/25515836/	https://www.ncbi.nlm.nih.gov/books/NBK1355/	
CLCN7	Osteopetrosis type 4	neurology	AD	N	0.4	Late childhood or adolescence	Osteoclast-rich ARO, inability to resorb bone and mineralized cartilage	Y	Skeletal survey		Bone marrow transplant (hematopoietic stem cell transplantation (HSCT))	HSCT				https://pubmed.ncbi.nlm.nih.gov/23877423/	https://www.ncbi.nlm.nih.gov/books/NBK1127/	
DMD	Duchenne muscular dystrophy and other dystrophinopathies	neurology	XLR	N		Early childhood	Atrophy in skeletal & heart muscle, muscle weakness, musclar dystrophy	Y	Serum creatine kinase (CK) level		Eteplirsen, Casimersen and Golodirsen for exon skipping 51, 45 and 53, respectively. Vitolarsen has also been approved for exon 53 skipping.	medication				https://pubmed.ncbi.nlm.nih.gov/25752877/	https://www.ncbi.nlm.nih.gov/books/NBK1119/	
FARSB	Rajab interstitial lung disease with brain calcifications 1	neurology	AR	N		Infancy (also can be later-onset)	ILD with cholesterol pneumonitis, growth delay with combined brain, liver and lung involvement , hypotonia, brain, calcifications with cysts & liver dysfunction	Y	qPCR or MLPA		No treatment found					https://pubmed.ncbi.nlm.nih.gov/31355908/	https://omim.org/entry/609690	
FOLR1	Cerebral folate transport deficiency	neurology	AR	N		Childhood	Disorders of the mitochondrial oxidative phosphorylation system, serine deficiency, & pyridoxine dependent epilepsy	Y	Cerebrospinal fluid 5-methyltetrahydrofolate level		Folinic acid	medication				https://pubmed.ncbi.nlm.nih.gov/30916789/	https://omim.org/entry/613068	
NF1	Neurofibromatosis type 1	neurology	AD	N		Infancy, childhood, adolescence	Disfiguring tumors, pilocytic astrocytomas, gastrointestinal stromal tumors, pheochromocytomas & juvenile myelomonocytic leukemia, malignant peripheral nerve sheath tumor	N			Selumetinib for plexiform neurofibromas	medication				https://pubmed.ncbi.nlm.nih.gov/25062113/	https://www.ncbi.nlm.nih.gov/books/NBK1109/	
PDGFRB	PDGFRB activating spectrum disorder	neurology	AD	N		Infancy, childhood	Kosaki overgrowth syndrome, solitary myofibromas, infantile myofibromatosis, Penttinen syndrome with premature aging & osteopenia, fusiform aneurysms	N			Imatinib and sunitinib	medication				https://pubmed.ncbi.nlm.nih.gov/32500973/		
PRPS1	Arts syndrome	neurology	XLR	N		Early childhood	Cogenital sensorineual hearing impariment, early-onset hypotonia, delayed motor develop intellectual disability, ataxia, & increased risk of infection	N			S-adenosylmethionine and nicotinamide riboside	medication				https://pubmed.ncbi.nlm.nih.gov/20301738/		
PRRT2	Episodic kinesigenic dyskinesia 1	neurology	AD	N	0.667	Infancy, childhood	Seizures,headache disorders, childhood-onset movement disorders, & intellecual disabilities	N			Oxcarbazepine; carbamazepine	medication				https://pubmed.ncbi.nlm.nih.gov/26598493/	https://www.ncbi.nlm.nih.gov/books/NBK475803/	

© 2023 Gold NB et al. *JAMA Network Open.*

Neurology (83 genes)

Gene	Disease name	System	Inheritance	On RUSP? (Y/N)	Prevalence - disease frequency per 100,000 (Rx genes, if listed)	Age of Onset	Disease symptoms	Orthogonal test? (Y/N)	If yes, orthogonal test	Is orthogonal test expected to be abnormal in infancy?	Intervention Considered (Free Text)	Category of Intervention	Age of Intervention Implementation	MD leading intervention	Comments	Link to ref 1	Link to ref 2	Link to ref 3
SLC5A6	Infantile-onset, biotin-responsive neurodegeneration	neurology	AR	N		Infancy	Microcephaly, cerebral palsy, developmental delay, variable immunodeficiency, acid reflux, osteoporosis, & pathologic bone fractures	N			biotin, pantothenic acid, lipoate	medication				https://pubmed.ncbi.nlm.nih.gov/27904971/		
SARS1	SARS1 associated neurodevelopmental disorder with microcephaly, ataxia, and seizures	neurology	AR	N		Infancy	Microcephal, ataxia, & seizures, arteriovenous malformations, intellectual disability, typhus, spine muscular dystrophy	N			serine supplementation	medication				https://www.genecards.org/cgi-bin/carddisp.pl?gene=SARS1#diseases	https://omim.org/entry/617709	

© 2023 Gold NB et al. *JAMA Network Open.*

Oncology (18 genes)

Gene	Disease name	System	Inheritance	On RUSP? (Y/N)	Prevalence - disease frequency per 100,000 (Rx genes, if listed)	Age of Onset	Disease symptoms	Orthogonal test? (Y/N)	If yes, orthogonal test	Is orthogonal test expected to be abnormal in infancy?	Intervention Considered (Free Text)	Category of Intervention	Age of Intervention Implementation	MD leading intervention	Comments	Link to ref 1	Link to ref 2	Link to ref 3
MSH2	Lynch Syndrome/(CMMRD if biallelic)	oncology	AD/(AR if biallelic)	N	35.2	Adulthood/Childhood if biallelic)	Colorectal, gyn, GI, GU, skin cancers	N			Surveillance	surveillance	Adulthood/Childhood if biallelic)	Pediatric heme/onc				
MLH1	Lynch Syndrome/(CMMRD if biallelic)	oncology	AD/(AR if biallelic)	N	51.4	Adulthood/Childhood if biallelic)	Colorectal, gyn, GI, GU, skin cancers	N			Surveillance	surveillance	Adulthood/Childhood if biallelic)	Pediatric heme/onc				
PMS2	Lynch Syndrome/(CMMRD if biallelic)	oncology	AD/(AR if biallelic)	N	140.1	Adulthood/Childhood if biallelic)	Colorectal, gyn, GI, GU, skin cancers	N			Surveillance	surveillance	Adulthood/Childhood if biallelic)	Pediatric heme/onc				
MSH6	Lynch Syndrome/(CMMRD if biallelic)	oncology	AD/(AR if biallelic)	N	131.9	Adulthood/Childhood if biallelic)	Colorectal, gyn, GI, GU, skin cancers	N			Surveillance	surveillance	Adulthood/Childhood if biallelic)	Pediatric heme/onc				
EPCAM	Lynch Syndrome/(CMMRD if biallelic)	oncology	AD/(AR if biallelic)	N		Adulthood/Childhood if biallelic)	Colorectal, gyn, GI, GU, skin cancers	N			Surveillance	surveillance	Adulthood/Childhood if biallelic)	Pediatric heme/onc				
APC	Familial Adenomatous Polyposis	oncology	AD	N	3.2-14.6	Infancy, Childhood, Adolescence	Colorectal cancer and polyposis, hepatoblastoma, thyroid cancer, desmoid tumors	N			Surveillance	surveillance	Infancy, Childhood, Adolescence	Pediatric heme/onc				
MUTYH	MUTYH-associated Polyposis	oncology	AR	N	1.7-3.5	Adulthood	Colorectal cancer and polyposis	N			Surveillance	surveillance	Adolescence	Pediatric heme/onc				
BMPR1A	BMPR1A-associated Polyposis	oncology	AD	N		Adolescence	Juvenile polyposis, colorectal and GI cancers	N			Surveillance	surveillance	Adolescence	Pediatric heme/onc				
ALK	ALK-Related Neuroblastic Tumor Susceptibility	oncology	AD	N		Infancy, Childhood	Neuroblastoma, ganglioneuroblastoma, ganglioneuroma	N			Surveillance	surveillance	Infancy	Pediatric heme/onc				
PHOX2B	Congenital Central Hypoventilation Syndrome	oncology	AD	N		Infancy, Childhood	Neuroblastoma, ganglioneuroblastoma, ganglioneuroma, hypoventilation, cardiac arrhythmia, neurocristopathy	N			Surveillance	surveillance	Infancy	Pediatric heme/onc				
DICER1	DICER1 Tumor Predisposition	oncology	AD	N	21.7	Infancy, Childhood	Pleuropulmonary blastoma (PPB), thyroid gland neoplasia, ovarian tumors, cystic nephroma, and others	N			Surveillance	surveillance	Infancy	Pediatric heme/onc				
PTCH1	Nevoid Basal Cell Carcinoma Syndrome	oncology	AD	N	3.2	Infancy, Childhood	Medulloblastoma, basal cell carcinoma, jaw keratocysts, macrocephaly, skeletal anomalies, cardiac and ovarian fibromas	N			Surveillance	surveillance	Infancy, Childhood	Pediatric heme/onc				
SUFU	Nevoid Basal Cell Carcinoma Syndrome	oncology	AD	N	3.2	Infancy, Childhood	Medulloblastoma, basal cell carcinoma, jaw keratocysts, macrocephaly, skeletal anomalies, cardiac and ovarian fibromas	N			Surveillance	surveillance	Infancy, Childhood	Pediatric heme/onc				
RET	Multiple Endocrine Neoplasia 2	oncology	AD	N	2.9	Childhood	Medullary thyroid carcinoma, pheochromocytoma, parathyroid adenoma	N			Surveillance	surgery/surveillance	Infancy, Childhood	Pediatric heme/onc				
TP53	Li-Fraumeni Syndrome	oncology	AD	N	18.3-28.1	Infancy, Childhood	Adrenocortical carcinomas, breast cancer, central nervous system tumors, osteosarcomas, soft-tissue sarcomas	N			Surveillance	surveillance	Infancy, Childhood	Pediatric heme/onc				
RB1	Hereditary Retinoblastoma	oncology	AD	N		Infancy, Childhood	Retinoblastoma, pineoblastoma	Y	Eye exams	Yes	Surveillance	surveillance	Infancy, Childhood	Pediatric heme/onc				
SMARCB1	Rhabdoid Tumor Predisposition Syndrome	oncology	AD	N		Infancy, Childhood	Renal or extrarenal malignant rhabdoid tumors syndrome	N			Surveillance	surveillance	Infancy, Childhood	Pediatric heme/onc				
WT1	WT1-Related Predisposition Syndrome	oncology	AD	N		Infancy, Childhood	Wilms tumor, nephrotic syndrome, disorders of testicular development, congenital GU anomalies syndrome	N			Surveillance	surveillance	Infancy, Childhood	Pediatric heme/onc				

© 2023 Gold NB et al. *JAMA Network Open.*

Ophthalmology (4 genes)

Gene	Disease name	System	Inheritance	On RUSP? (Y/N)	Prevalence - disease frequency per 100,000 (Rx genes, if listed)	Age of Onset	Disease symptoms	Orthogonal test? (Y/N)	If yes, orthogonal test	Is orthogonal test expected to be abnormal in infancy?	Intervention Considered (Free Text)	Category of Intervention	Age of Intervention Implementation	MD leading intervention	Comments	Link to ref 1	Link to ref 2	Link to ref 3
PLG	Plasminogen deficiency, type I	ophthalmology	AR	N	0.16	Childhood	Chronic mucosal pseudomembranous lesions, hydrocephalus	Y	Plasminogen activity		Ryplazim	medication	Childhood	pediatric hematology				
RPE65	RPE65 associated Leber congenital amaurosis, early-onset severe retinal dystrophy	ophthalmology	AR	N	0.685	Infancy or later	Retinal dystrophy	Y	Electroretinography and retinal imaging		Luxturna	medication	Infancy, childhood	pediatric ophthalmology				
SLC6A6	Taurine transporter deficiency	ophthalmology	AR	N		Childhood	Retinal degeneration, cardiomyopathy	Y	Plasma amino acids		Taurine	medication	Childhood	pediatric metabolism				
VAMP1	Congenital myasthenic syndrome 25	ophthalmology	AR	N	Gene is misclassified and should be in the neurology section													

© 2023 Gold NB et al. *JAMA Network Open.*

Pulmonology (2 genes)

Gene	Disease name	System	Inheritance	On RUSP? (Y/N)	Prevalence - disease frequency per 100,000 (Rx genes, if listed)	Age of Onset	Disease symptoms	Orthogonal test? (Y/N)	If yes, orthogonal test	Is orthogonal test expected to be abnormal in infancy?	Intervention Considered (Free Text)	Category of Intervention	Age of Intervention Implementation	MD leading intervention	Comments	Link to ref 1	Link to ref 2	Link to ref 3
SERPINA1	Alpha-1-antitrypsin deficiency	pulmonology	AR	N	20	Childhood	Liver dysfunction, COPD (adults)	Y	serum concentration of alpha-1 antitrypsin		Liver transplant, infusion of purified human AAT	tranfusion OT	Infancy, childhood, adulthood	Pediatric GI, adult pulmonology				
SFTPC	Pulmonary surfactant metabolism dysfunction 2	pulmonology	AD	N		Infancy	Respiratory insufficiency/failure	N			Hydroxychloroquinone	medication	Infancy	Pediatric pulmonology		https://pubmed.ncbi.nlm.nih.gov/15647591/		

© 2023 Gold NB et al. *JAMA Network Open.*

eTable 4. Demographic Information of the Respondents

Demographic	N (%)
Mean age (years)	52.6; SD = 12.8 (range 27-93)
**Gender**	
Female	126 (52.9%)
Male	112 (47.1%)
**Race**	
Asian	26 (10.9%)
Native Hawaiian/Pacific Islander	2 (0.8%)
White	141 (59.2%)
Multiracial	4 (1.7%)
Other	5 (2.1%)
Unknown	60 (25.2%)
**Ethnicity**	
Hispanic	7 (2.9)
Non-Hispanic	169 (71.0)
Unknown	62 (26.1)

© 2023 Gold NB et al. *JAMA Network Open.*

eTable 5. Additional genes suggested for inclusion by respondents

Clinical Area	Additional Suggested Genes
Cardiovascular	*ACTA2, ELN, FBN1, KCNE1, KCNQ1, MYBPC3, MYH7, PTPN11, RIT1, SCN5A, TGFBR1, TGFBR2*
Endocrinology	*CASR, PPARG*
Gastroenterology	*ABCB11, ABCB4, ALGS, ATP8B1, LCT, MYO5B, WNT2B*
Hematology	*ANKRD26, CDC42, CEBPA, DDX41, ERCC1, ETV6, F2, F5, PAX5, RUNX1*
Immunology	*BCL10, CASP8, CD28, CD3G, EXTL3, HEM1, ICOSLG, IL7, PDCD1, RIPK1, SASH3, SOCS1, TNFRSF6, TNFSF6*
Metabolism	*GM1, GYS1, GYS2, MT-APT6, PEX1, PEX10, PEX11A, PEX11B, PEX11G, PEX12, PEX13, PEX14, PEX16, PEX19, PEX2, PEX26, PEX3, PEX5, PEX6, PEX7, PPA2, SLC25A26, TKT, TYMP, UROS*
Nephrology	*APOL1, PKHD1, SLC3A1, SLC7A9*
Neurology	*CREBBP, EHMT1, EP300, FMR1, KMT2C, KMT2E, MECP2, NSD1, UBE3A*
Oncology	*BLM, CHEK2, FH, FMTC, LZTR1, NF2, PTEN, SDHA, SDHB, SDHC, SDHD, VHL*
Ophthalmology	*ALMS1, CDH23, CLRN1, CYP1B1, GPR98, LRMDA, MTP, MYO7A, MYOC, OCA2, PCHD15, SLC24A5, SLC45A2, TYR, TYRP1, USH1C, USH1G, USH2A, WHRN*
Pulmonology	*ACVRL1, CSF2RA, CSF2RB, ENG, EPHB4, GDF2, RASA1, TERT*

© 2023 Gold NB et al. *JAMA Network Open.*
