# Supplementary material for: Perspectives of Rare Disease Experts on Newborn Genome Sequencing
Source: JAMA Netw Open. 2023 May 8;6(5):e2312231. doi: 10.1001/jamanetworkopen.2023.12231 (PMC10167563; doi:10.1001/jamanetworkopen.2023.12231)
Supplement: Supplement 2. — Data Sharing Statement [file jamanetwopen-e2312231-s002.pdf]

Data Sharing Statement

Gold. Perspectives of Rare Disease Experts on Newborn Genome Sequencing. *JAMA Netw Open*. Published May 08, 2023. doi:10.1001/jamanetworkopen.2023.12231

Data
Data available: Yes
Data types: Deidentified participant data
How to access data: ngold@mgh.harvard.edu
When available: With publication

Supporting Documents
Document types: None

Additional Information
Who can access the data: Researchers whose proposed use of the data has been approved
Types of analyses: For a specified purpose
Mechanisms of data availability: After approval of a proposal
Any additional restrictions: None
